# Comparison of RNA-Seq and microarray in the prediction of protein expression and survival prediction

**DOI:** 10.3389/fgene.2024.1342021

**Published:** 2024-02-23

**Authors:** Won-Ji Kim, Bo Ram Choi, Joseph J. Noh, Yoo-Young Lee, Tae-Joong Kim, Jeong-Won Lee, Byoung-Gie Kim, Chel Hun Choi

**Affiliations:** ^1^ Department of Obstetrics and Gynecology, Samsung Medical Center, Sungkyunkwan University School of Medicine, Seoul, Republic of Korea; ^2^ Department of Obstetrics and Gynecology, Seoul St. Mary’s Hospital, College of Medicine, The Catholic University of Korea, Seoul, Republic of Korea

**Keywords:** RNA-seq, microarray, TCGA, biomarkers, protein expression, RPPA

## Abstract

Gene expression profiling using RNA-sequencing (RNA-seq) and microarray technologies is widely used in cancer research to identify biomarkers for clinical endpoint prediction. We compared the performance of these two methods in predicting protein expression and clinical endpoints using The Cancer Genome Atlas (TCGA) datasets of lung cancer, colorectal cancer, renal cancer, breast cancer, endometrial cancer, and ovarian cancer. We calculated the correlation coefficients between gene expression measured by RNA-seq or microarray and protein expression measured by reverse phase protein array (RPPA). In addition, after selecting the top 103 survival-related genes, we compared the random forest survival prediction model performance across test platforms and cancer types. Both RNA-seq and microarray data were retrieved from TCGA dataset. Most genes showed similar correlation coefficients between RNA-seq and microarray, but 16 genes exhibited significant differences between the two methods. The BAX gene was recurrently found in colorectal cancer, renal cancer, and ovarian cancer, and the PIK3CA gene belonged to renal cancer and breast cancer. Furthermore, the survival prediction model using microarray was better than the RNA-seq model in colorectal cancer, renal cancer, and lung cancer, but the RNA-seq model was better in ovarian and endometrial cancer. Our results showed good correlation between mRNA levels and protein measured by RPPA. While RNA-seq and microarray performance were similar, some genes showed differences, and further clinical significance should be evaluated. Additionally, our survival prediction model results were controversial.

## Introduction

Proteins play a critical role in a wide range of biological processes, including cell differentiation, metabolism, and signaling. Dysregulation of protein expression can contribute to the development and progression of numerous diseases, including cancer. Therefore, accurate prediction of protein expression levels is crucial for understanding disease mechanisms, identifying potential therapeutic targets, and developing personalized treatment strategies.

RNA-sequencing (RNA-seq) and microarray are two commonly used technologies for measuring gene expression levels. However, to date, a comprehensive comparison of RNA-seq and microarray-based predictive models is lacking. RNA-seq has become a standard tool in biological and medical research, providing advantages over microarrays. This method has been used for a variety of purposes, including estimating gene expression and discovering novel genes, alternative transcript variants, chimeric transcripts, expressed sequence variants, and allele-specific expression (Sultan et al., 2008; Ozsolak and Milos, 2011; Djebali et al., 2012; Ferreira et al., 2014; SEQC/MAQC-III Consortium, 2014). Additionally, RNA-seq data have been used to develop gene expression–based predictive models in cancer research, and the technique may outperform microarray-based models for clinical endpoint prediction due to its ability to decipher global gene expression patterns (Cancer Genome Atlas Research Network, 2013; Volinia and Croce, 2013). However, the effectiveness of these technologies in predicting protein expression has not been thoroughly compared.

Microarray-based gene expression profiling has been applied in cancer research and is widely used due to its lower cost and the availability of large and well-maintained repositories (Beer et al., 2002; Pomeroy et al., 2002; van 't Veer et al., 2002; Glinsky et al., 2004; Glas et al., 2005; Barrett et al., 2013; Kolesnikov et al., 2015). Therefore, while most future gene expression studies will use RNA-Seq platforms, the available microarray datasets represent a significant amount of resources.

In this study, we compared the effectiveness of RNA-seq and microarray-based technologies in predicting protein expression and clinical endpoints across multiple cancer types. To achieve this goal, we analyzed global gene expression profiles from The Cancer Genome Atlas (TCGA), a large cohort of more than 30 human tumors subjected to large-scale genome sequencing and integrated multi-dimensional analyses using both RNA-seq and microarrays. Our specific focus was on cancer samples from six different types. Our findings will provide valuable guidance on the optimal use of these technologies in both research and clinical settings.

## Methods

### Data collection for the prediction model

In this study, we investigated the correlation between gene expression and protein expression across various cancer types. We collected data from The Cancer Genome Atlas (TCGA) Data Portal, encompassing mRNA expression through RNA-seq and microarray, as well as protein expression via reverse phase protein array (RPPA). Information on clinical and molecular data for mRNA and protein expression was collected from 4,747 samples across 14 cancer types (available at TCGA Data Portal, https://tcgadata.nci.nih.gov/docs/publications/tcga/). For microarray data, gene level normalization was performed using the Robust Multi-array Average (RMA) (Irizarry et al., 2003) algorithm on GenePattern (Reich et al., 2006). The RNAseq gene expression level 3 data include reads per kilobase per million mapped reads (RPKM) (Mortazavi et al., 2008), RNAseq by expectation-maximization (RSEM) (Li and Dewey, 2011), and read count. The detailed differences between RPKM and RSEM are described by Li et al. (Li et al., 2010). We used publicly available data, carefully curated and chose RSEM as the preferred method. The gene expression profile was measured experimentally using the Illumina HiSeq 2000 RNA Sequencing platform by the University of North Carolina TCGA genome characterization center. Level 3 data was downloaded from TCGA data coordination center. This dataset shows the gene-level transcription estimates, as in log2 (x+1) transformed RSEM normalized count. Genes are mapped onto the human genome coordinates using UCSC Xena HUGO probeMap. In order to more easily view the differential expression between samples, we set the default view to center each gene or exon to zero by independently subtracting the mean of each gene or exon. The processing protocols are described in detail in TCGA open access FTP download directories. We adhered to TCGA publication guidelines (http://cancergenome.nih.gov/publications/publicationguidelines). Table 1 provide data descriptions of the multilevel genomic datasets, including sample distributions across technologies and cancer types.

**TABLE 1:** TCGA sample description.

Cancer abbreviation	Cancer name	No. of samples		No. Of genes
		RNA seq	Array	
LUSC	Lung squamous cell carcinoma	82	82	103
COAD	Colon adenocarcinoma	211	103	25
KIRC	Kidney renal clear cell carcinoma	44	44	103
BRCA	Breast invasive carcinoma	420	420	103
UCEC	Uterine corpus endometrioid carcinoma	102	49	25
OV	Ovarian serous cystadenocarcinoma	200	200	103

## Comparing the correlation R

First, we measured the Pearson correlation coefficients between gene expression using RNA seq and protein expression using RPPA for each gene in each cancer type. Then, we repeated this process using microarray data instead of RNA seq. Next, we compared the two correlation coefficients for each gene in each cancer type to determine significant differences between the two methods. Furthermore, we conducted additional analysis at the exon level, employing the method ‘Estimate Genewise Dispersions from Exon-Level Count Data.’ with comparative analysis on copy number (CN) such as CN vs protein expression (RPPA), CN vs sequencing data, CN vs array data, and CN mean.

## RandomSurvivalForest

Given the comprehensive transcriptomic information provided by RNA-seq, we hypothesized its superiority in gene expression-based survival prediction compared to microarrays. To evaluate this hypothesis, RNAseq and microarray-based expression data were used to predict survival. After selecting the top 103 genes related to survival through cox univariate analysis, we randomly divided the dataset into training (80%) and test (20%) sets. Next, we developed survival prediction models using the training set through the random survival forest (RSF) algorithm in the R package “RandomSurvivalForest” (Ishwaran and Kogalur, 2010) with the recommended default reference values.

**FIGURE 1:**
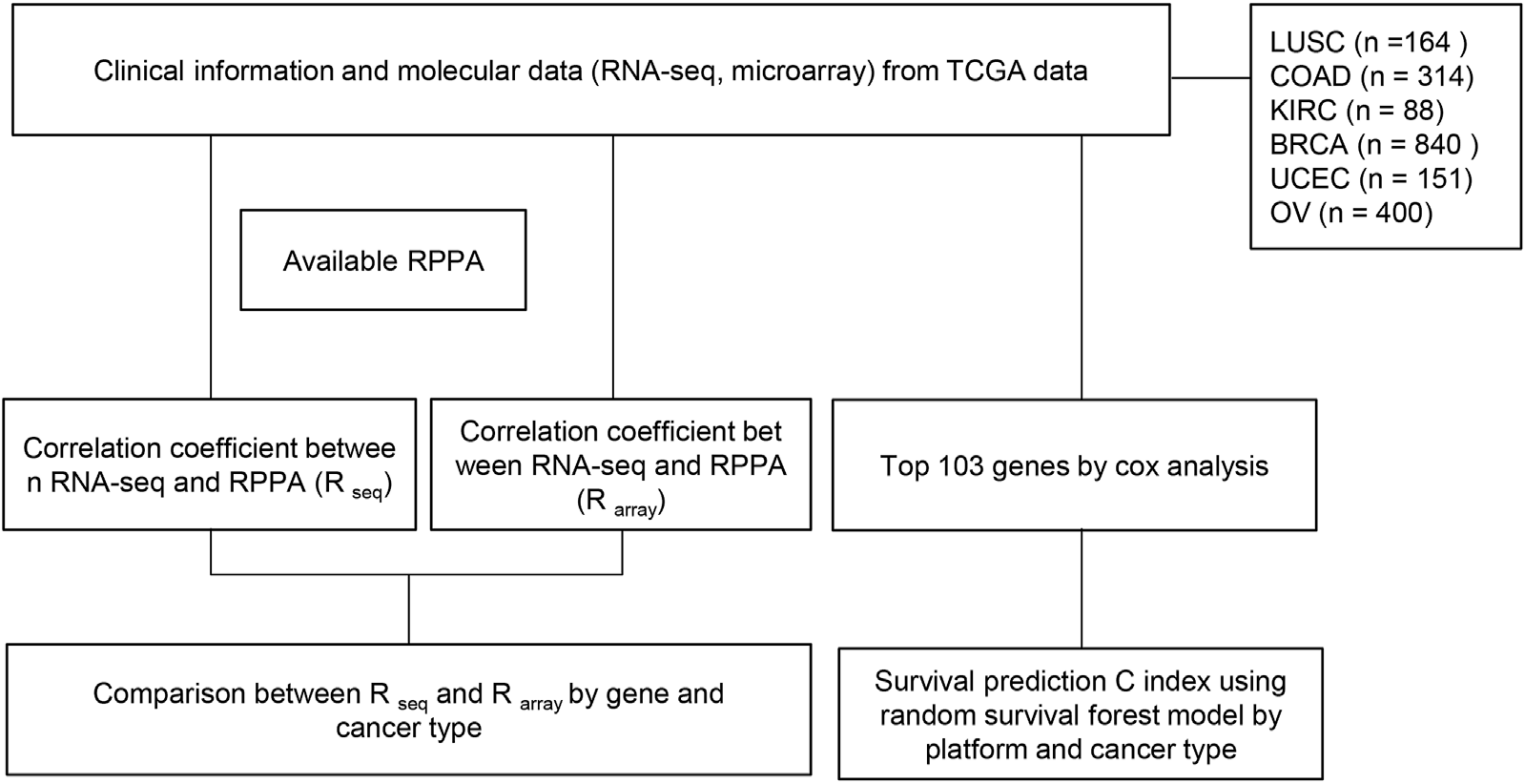
Flow chart of study. RNA-seq and microarray data from TCGA were employed to predict survival. (1) Correlation coefficients between gene expression (RNA seq) and protein expression (RPPA) were measured for each gene in each cancer type. This process was repeated using microarray data instead of RNA seq. Subsequently, a comparison of the two correlation coefficients for each gene in each cancer type was conducted to identify significant differences between the two methods. (2) Focusing on the top 103 genes, the dataset was randomly split into training (80%) and test (20%) sets, and random survival forest (RSF) models were developed. The models were tested on the test set, and C-index was calculated 103 times for each core set to evaluate prognostic performance. The Wilcoxon signed-rank test (*p*-value cutoff of 0.05) was used to compare performance across different data types, and box plots of C-index values were generated for each testing platform and cancer type.

We applied the trained models to the test set for prediction and evaluated the prognostic performance by measuring the C-index. The C-index measures the degree of agreement between the survival time ranking and the selected model. A C-index of 1 means perfect discriminatory power, and a C-index of 0.5 means not better than chance results. To ensure the robustness of the analysis, we repeated the entire process 103 times for each core set and obtained 103 C-index values. To compare the performance of different data types, we used the Wilcoxon signed-rank test with a significance cutoff of 0.05 to assess the *p*-value. Finally, we plotted the box plots of the C-index values for each testing platform and cancer type (Figure 1).

## Results

### TCGA data

To evaluate the accuracy of gene expression-based prediction of clinical endpoints in different cancer types, we compared the gene expression determined by RNA-seq and microarray platforms predicting protein expression in 6 cancer types, using TCGA data. To ensure a fair comparison, we limited our analysis to only genes that were available in RPPA in both platforms.

### Comparing the correlation R

**FIGURE 2:**
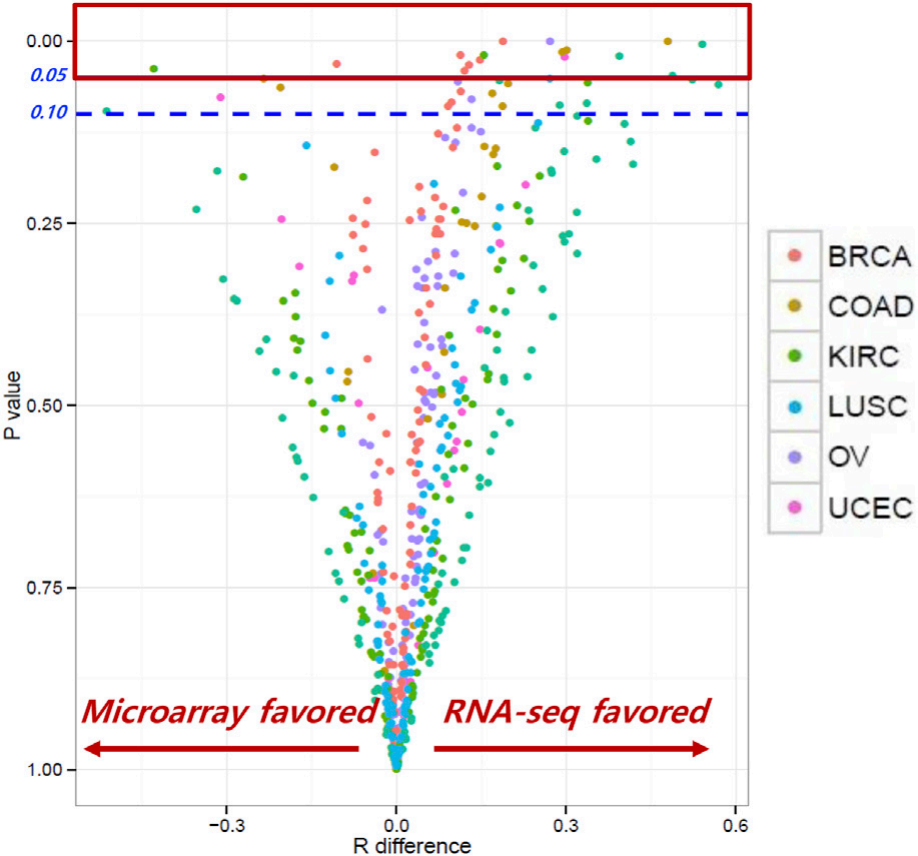
Correlation coefficients between RNA-seq and microarray. Most genes showed similar correlation coefficients between RNA-seq and microarray; 16 genes showed significant differences between the two methods.

The correlation coefficients (R_RNA-seq_ and R_microarray_) between gene expression and RPPA were measured for RNA-seq and microarray data. Our analysis found that, for most genes, the correlation coefficients of gene expression using RNA-seq and microarray were not significantly different, indicating comparable results between the two platforms. R difference plot (R_RNA-seq_–R_microarray_) with *p*-value (Figure 2) identified 16 genes that showed significant differences in correlation between RNA-seq and microarray methods (Table 2). These differences can be attributed to the technological differences in quantifying gene expression between the two platforms. Among the 16 genes, the BAX gene was identified three times in colorectal cancer, renal cancer, and ovarian cancer. The PIK3CA gene was found to have higher correlation in microarray for renal cancer and breast cancer, while other genes showed better results in RNA-seq. Our analysis underscored that the correlation between gene expression and protein expression was stronger when using RNA seq data for certain genes or cancer types, whereas microarray data exhibited stronger correlation in other gene or cancer types. Overall, our findings emphasize the importance of selecting the appropriate method for measuring gene expression when investigating its correlation with protein expression in different cancer types. Our findings also demonstrated concordance at the gene, transcript, and exon levels. In-depth comparative analyses, including copy number (CN) *versus* protein expression (RPPA), CN *versus* sequencing data, CN *versus* array data, and CN mean, indicated that these factors do not seem to have a substantial impact on our results. To ensure a comprehensive understanding, additional investigations are warranted to thoroughly examine the copy numbers of all genes (Supplementary Figures S2–S14).

**TABLE 2:** Genes that showed significant differences in correlation between RNA-seq and microarray methods.

Cancer	Gene	R difference	*p*-value (R difference)
LUSC	*CCNE1*	0.540,395	0.003851
LUSC	*CCNB1*	0.393,845	0.021048
LUSC	*ERRFI1*	0.487,971	0.048153
COAD	*BAX*	0.480,163	0.000002
COAD	*CDH2*	0.300,873	0.012675
COAD	*BIRC2*	0.292,883	0.014629
KIRC	*KIT*	0.154,533	0.019826
KIRC	*PIK3CA*	−0.429,780	0.038242
BRCA	*BAX*	0.147,752	0.025992
BRCA	*SRC*	0.112,956	0.019650
BRCA	*PIK3CA*	−0.106,080	0.031063
BRCA	*BCL2L11*	0.128,137	0.033394
BRCA	*PCNA*	0.120,537	0.040727
BRCA	*MAPK9*	0.188,264	0.000209
UCEC	*ACACA*	0.27515	0.022054
OV	*BAX*	0.271,574	0.000979

Italics were used specifically to denote a gene symbol as a way to differentiate it from the gene product.

### Survival correlation

**FIGURE 3:**
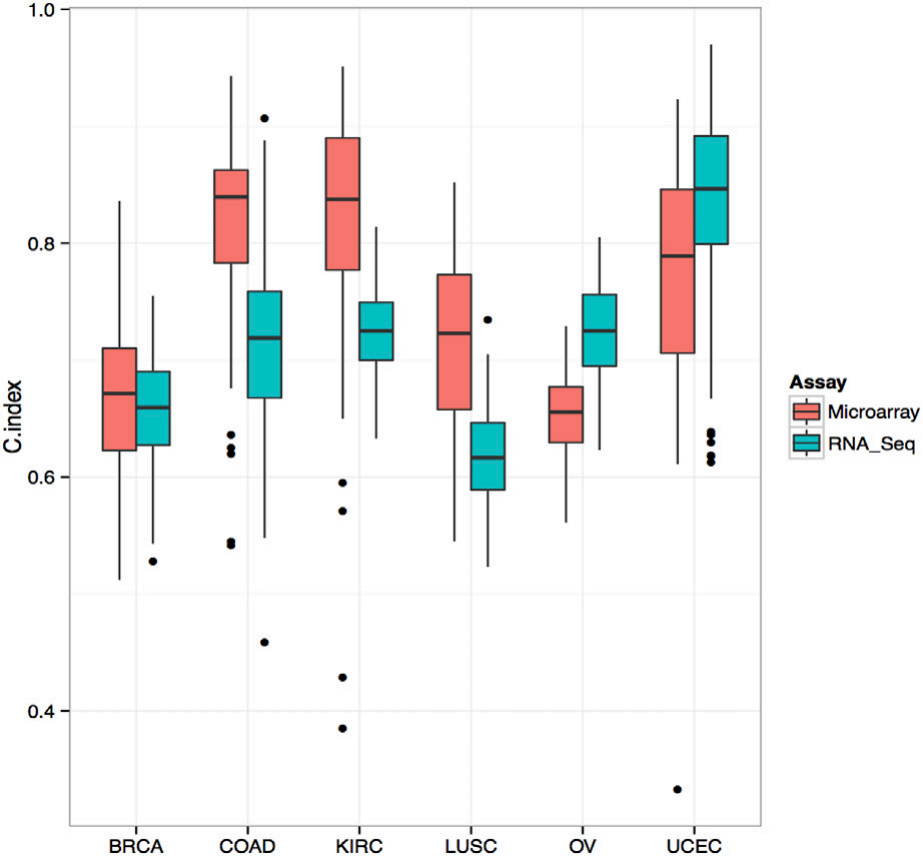
Comparison of C-Index for Survival Prediction between Microarray and RNA-Seq in Various Cancer Types Comparative analysis of Concordance Index (C-Index) for survival prediction between microarray and RNA-Seq data. The top 103 genes associated with survival were selected using Cox univariate analysis from RNA-Seq data. The C-Index was calculated using a random survival forest model. In several cancer types, including colorectal cancer, renal cancer, and small cell lung cancer, the C-Index derived from microarray data surpasses that of RNA-Seq, indicating its superior predictive performance in these contexts.

We selected the top 103 genes predicting survival in each cancer type using RNA-seq and microarray data and evaluated their prognostic performance using the random survival forest model. Our analysis found that, in colorectal cancer, renal cancer, and small cell lung cancer, the C-index of microarray was higher than that of RNA-seq (Figure 3). However, in ovarian and endometrial cancers, RNA-seq-based models performed significantly better than microarray-based models in predicting survival. Overall, our data demonstrate that RNA-seq and microarray models perform similarly well in predicting clinical endpoints. Further studies are needed to validate these results and elucidate the underlying mechanisms of gene expression regulation in cancer progression and survival.

## Discussion

In this study, we compared the ability of RNA-seq and microarrays to predict clinical endpoints. Overall, we observed a strong correlation between mRNA levels and protein expression measured by RPPA and found comparable performance of RNA-seq and microarrays in predicting protein expression. Notably, we identified 14 gene/protein pairs that showed different correlation coefficients between the two methods, with RNA-seq showing a higher correlation in most cases except for PIK3CA gene. The BAX gene had different correlation coefficients in the three tumor types, while the PIK3CA gene had different correlation coefficients in two tumor types. We compared the differences in genetic characteristics such as molecular weight and function for genes that showed different correlation, but no significant differences were found (Supplementary Table S1). The reasons for these discrepancies require further investigation. In terms of survival prediction models, microarrays performed better in COAD, KIRC, and LUSC, while RNA-seq performed better in ovarian and endometrial cancers. Overall, our results do not support the hypothesis that the more extensive transcriptomic information provided by RNA-seq necessarily improves gene expression–based prediction performances in all cases. Further studies are needed to explore the underlying factors that contribute to the observed differences.

Over the past 2 decades, microarrays were the predominant sequencing technology used for investigating transcriptomics. However, the emergence of next-generation sequencing (NGS), particularly RNA sequencing (RNA-Seq), has gradually replaced microarrays in gene expression analysis (Wang et al., 2009). RNA-Seq provides a broader dynamic range than microarrays, rendering it more adept at identifying low-abundance transcripts and facilitating novel transcript detection and analysis (Ozsolak and Milos, 2011; Zhao et al., 2014; Rao et al., 2018). Additionally, RNA-Seq does not necessarily depend on a reference genome, enabling transcriptome analysis in organisms lacking such a reference. These advantages have made RNA-Seq increasingly popular and led to a reduction in its overall cost. In this context, achieving high comparability between data from the two platforms has been crucial for meta-analysis of gene expression across multiple studies. While the preprocessing and analysis steps of microarray data are largely standardized, the establishment of RNA-Seq data analysis methodology and standards is ongoing. Several studies have compared microarray and RNA-Seq technologies for transcriptome profiling, each shedding light on their respective strengths and limitations. L. Chen et al. concentrated on lung squamous cell carcinoma samples from TCGA, simultaneously employing RNA-Seq v2 and three microarray platforms. The analysis revealed a significant correlation (89.8%) between the two methods for 11,120 genes, emphasizing the reproducibility of results (Chen et al., 2017). Another study, focusing on the HrpX regulome in Xanthomonas citri subsp. citri, demonstrated the complementary nature of RNA-Seq and microarray technologies in detecting target genes, thereby advancing understanding of the regulome (Kogenaru et al., 2012). The study, which systematically compared RNA-Seq and microarray-based classifiers for clinical endpoint prediction using neuroblastoma as a model, revealed that RNA-Seq outperformed in determining transcriptomic characteristics. However, both methods performed similarly in prediction models (Zhang et al., 2015). The comparative study using two microarray platform (Affymetrix one-channel microarray and Agilent two-channel microarray) and RNA-seq on TCGA data revealed high correlations (Spearman >0.8) between Affymetrix microarray and RNA-seq, particularly for highly abundant genes. However, Agilent microarray and RNA-seq showed poor correlations (Spearman <0.2), indicating that the correlation between microarray and RNA-seq expression data can be influenced by platform differences and abundance levels. Despite some discrepancies in gene directionality changes between Agilent microarray and RNA-seq, overall, RNA-seq proved comparable to microarrays in expression profiling. In this study, normalization methods RPKM and RSEM showed similar gene-level results with reasonable concordance at the exon level, demonstrating better concordance at the longer exons than shorter ones between the two normalization methods (Guo et al., 2013). A review article demonstrated the evolving landscape, acknowledging that microarrays remain reliable and cost-effective for specific applications, while RNA-seq, though increasingly routine, complements microarrays in certain contexts (Mantione et al., 2014). Another previous showed that background hybridization and probe saturation in microarrays can limit sensitivity in both low and high expression levels, despite the correlation values between microarray and RNA-Seq measurements (Zhao et al., 2014). As RNA-Seq costs continue to decline, this method is expected to fully replace microarrays; nonetheless, the existing microarray data should not be disregarded.

The findings of our study have potential applications in several areas of cancer research. First, the identification of 14 gene/protein pairs that showed different correlation coefficients between RNA-seq and microarray data suggests that caution should be exercised when comparing data from the two platforms. Future studies should explore the underlying reasons for these discrepancies and develop strategies to reconcile the differences. Second, the controversy surrounding the choice of platform for survival prediction modeling in different cancer types highlights the importance of choosing the appropriate technology for each specific research question. Third, the higher dynamic range and accuracy in low-abundance measurements of RNA-seq make it a suitable platform for detecting novel transcripts and analyzing transcriptomics data. This has implications for the identification of new therapeutic targets and biomarkers for cancer diagnosis and treatment. Overall, our study highlights the need for careful consideration of platform choice and data interpretation in cancer research.

In conclusion, our study demonstrated that RNA-seq and microarrays showed similar performance in predicting clinical endpoints, except for a few genes such as PIK3CA and BAX in certain tumor types. These findings have implications for the selection of sequencing technologies in future research and highlight the importance of comparability between platforms to increase the power of gene expression–based prediction models.

## Data Availability

The datasets presented in this study can be found in online repositories. The names of the repository/repositories and accession number(s) can be found below: https://www.ncbi.nlm.nih.gov/gap/, phs000178.v11.p8.

## References

[B1] Barrett T. Wilhite S. E. Ledoux P. Evangelista C. Kim I. F. Tomashevsky M. (2013). NCBI GEO: archive for functional genomics data sets--update. Nucleic Acids Res. 41, D991–D995. 10.1093/nar/gks1193 23193258 PMC3531084

[B2] Beer D. G. Kardia S. L. Huang C. C. Giordano T. J. Levin A. M. Misek D. E. (2002). Gene-expression profiles predict survival of patients with lung adenocarcinoma. Nat. Med. 8, 816–824. 10.1038/nm733 12118244

[B3] Cancer Genome Atlas Research Network (2013). Comprehensive molecular characterization of clear cell renal cell carcinoma. Nature 499, 43–49. 10.1038/nature12222 23792563 PMC3771322

[B4] Chen L. Sun F. Yang X. Jin Y. Shi M. Wang L. (2017). Correlation between RNA-Seq and microarrays results using TCGA data. Gene 628, 200–204. 10.1016/j.gene.2017.07.056 28734892

[B5] Djebali S. Davis C. A. Merkel A. Dobin A. Lassmann T. Mortazavi A. (2012). Landscape of transcription in human cells. Nature 489, 101–108. 10.1038/nature11233 22955620 PMC3684276

[B6] Ferreira P. G. Jares P. Rico D. Gómez-López G. Martínez-Trillos A. Villamor N. (2014). Transcriptome characterization by RNA sequencing identifies a major molecular and clinical subdivision in chronic lymphocytic leukemia. Genome Res. 24, 212–226. 10.1101/gr.152132.112 24265505 PMC3912412

[B7] Glas A. M. Kersten M. J. Delahaye L. J. Witteveen A. T. Kibbelaar R. E. Velds A. (2005). Gene expression profiling in follicular lymphoma to assess clinical aggressiveness and to guide the choice of treatment. Blood 105, 301–307. 10.1182/blood-2004-06-2298 15345589

[B8] Glinsky G. V. Glinskii A. B. Stephenson A. J. Hoffman R. M. Gerald W. L. (2004). Gene expression profiling predicts clinical outcome of prostate cancer. J. Clin. Invest. 113, 913–923. 10.1172/jci20032 15067324 PMC362118

[B9] Guo Y. Sheng Q. Li J. Ye F. Samuels D. C. Shyr Y. (2013). Large scale comparison of gene expression levels by microarrays and RNAseq using TCGA data. PLoS One 8, e71462. 10.1371/journal.pone.0071462 23977046 PMC3748065

[B10] Irizarry R. A. Hobbs B. Collin F. Beazer-Barclay Y. D. Antonellis K. J. Scherf U. (2003). Exploration, normalization, and summaries of high density oligonucleotide array probe level data. Biostatistics 4, 249–264. 10.1093/biostatistics/4.2.249 12925520

[B11] Ishwaran H. Kogalur U. B. (2010). Consistency of random survival forests. Stat. Probab. Lett. 80, 1056–1064. 10.1016/j.spl.2010.02.020 20582150 PMC2889677

[B12] Kogenaru S. Qing Y. Guo Y. Wang N. (2012). RNA-seq and microarray complement each other in transcriptome profiling. BMC Genomics 13, 629. 10.1186/1471-2164-13-629 23153100 PMC3534599

[B13] Kolesnikov N. Hastings E. Keays M. Melnichuk O. Tang Y. A. Williams E. (2015). ArrayExpress update--simplifying data submissions. Nucleic Acids Res. 43, D1113–D1116. 10.1093/nar/gku1057 25361974 PMC4383899

[B14] Li B. Dewey C. N. (2011). RSEM: accurate transcript quantification from RNA-Seq data with or without a reference genome. BMC Bioinforma. 12, 323. 10.1186/1471-2105-12-323 PMC3163565 21816040

[B15] Li B. Ruotti V. Stewart R. M. Thomson J. A. Dewey C. N. (2010). RNA-Seq gene expression estimation with read mapping uncertainty. Bioinformatics 26, 493–500. 10.1093/bioinformatics/btp692 20022975 PMC2820677

[B16] Mantione K. J. Kream R. M. Kuzelova H. Ptacek R. Raboch J. Samuel J. M. (2014). Comparing bioinformatic gene expression profiling methods: microarray and RNA-Seq. Med. Sci. Monit. Basic Res. 20, 138–142. 10.12659/msmbr.892101 25149683 PMC4152252

[B17] Mortazavi A. Williams B. A. McCue K. Schaeffer L. Wold B. (2008). Mapping and quantifying mammalian transcriptomes by RNA-Seq. Nat. Methods 5, 621–628. 10.1038/nmeth.1226 18516045 PMC13303166

[B18] Ozsolak F. Milos P. M. (2011). RNA sequencing: advances, challenges and opportunities. Nat. Rev. Genet. 12, 87–98. 10.1038/nrg2934 21191423 PMC3031867

[B19] Pomeroy S. L. Tamayo P. Gaasenbeek M. Sturla L. M. Angelo M. McLaughlin M. E. (2002). Prediction of central nervous system embryonal tumour outcome based on gene expression. Nature 415, 436–442. 10.1038/415436a 11807556

[B20] Rao M. S. Van Vleet T. R. Ciurlionis R. Buck W. R. Mittelstadt S. W. Blomme E. A. G. (2018). Comparison of RNA-seq and microarray gene expression platforms for the toxicogenomic evaluation of liver from short-term rat toxicity studies. Front. Genet. 9, 636. 10.3389/fgene.2018.00636 30723492 PMC6349826

[B21] Reich M. Liefeld T. Gould J. Lerner J. Tamayo P. Mesirov J. P. (2006). GenePattern 2.0. Nat. Genet. 38, 500–501. 10.1038/ng0506-500 16642009

[B22] SEQC/MAQC-III Consortium (2014). A comprehensive assessment of RNA-seq accuracy, reproducibility and information content by the Sequencing Quality Control Consortium. Nat. Biotechnol. 32, 903–914. 10.1038/nbt.2957 25150838 PMC4321899

[B23] Sultan M. Schulz M. H. Richard H. Magen A. Klingenhoff A. Scherf M. (2008). A global view of gene activity and alternative splicing by deep sequencing of the human transcriptome. Science 321, 956–960. 10.1126/science.1160342 18599741

[B24] van 't Veer L. J. Dai H. van de Vijver M. J. He Y. D. Hart A. A. Mao M. (2002). Gene expression profiling predicts clinical outcome of breast cancer. Nature 415, 530–536. 10.1038/415530a 11823860

[B25] Volinia S. Croce C. M. (2013). Prognostic microRNA/mRNA signature from the integrated analysis of patients with invasive breast cancer. Proc. Natl. Acad. Sci. U. S. A. 110, 7413–7417. 10.1073/pnas.1304977110 23589849 PMC3645522

[B26] Wang Z. Gerstein M. Snyder M. (2009). RNA-Seq: a revolutionary tool for transcriptomics. Nat. Rev. Genet. 10, 57–63. 10.1038/nrg2484 19015660 PMC2949280

[B27] Zhang W. Yu Y. Hertwig F. Thierry-Mieg J. Zhang W. Thierry-Mieg D. (2015). Comparison of RNA-seq and microarray-based models for clinical endpoint prediction. Genome Biol. 16, 133. 10.1186/s13059-015-0694-1 26109056 PMC4506430

[B28] Zhao S. Fung-Leung W. P. Bittner A. Ngo K. Liu X. (2014). Comparison of RNA-Seq and microarray in transcriptome profiling of activated T cells. PLoS One 9, e78644. 10.1371/journal.pone.0078644 24454679 PMC3894192

