# Supplementary figures and images for: Comparison of RNA-Seq and microarray in the prediction of protein expression and survival prediction
Source: Front Genet. 2024 Feb 23;15:1342021. doi: 10.3389/fgene.2024.1342021 (PMC10920353; doi:10.3389/fgene.2024.1342021)

Supplementary Fig.1 Densitogram of R value

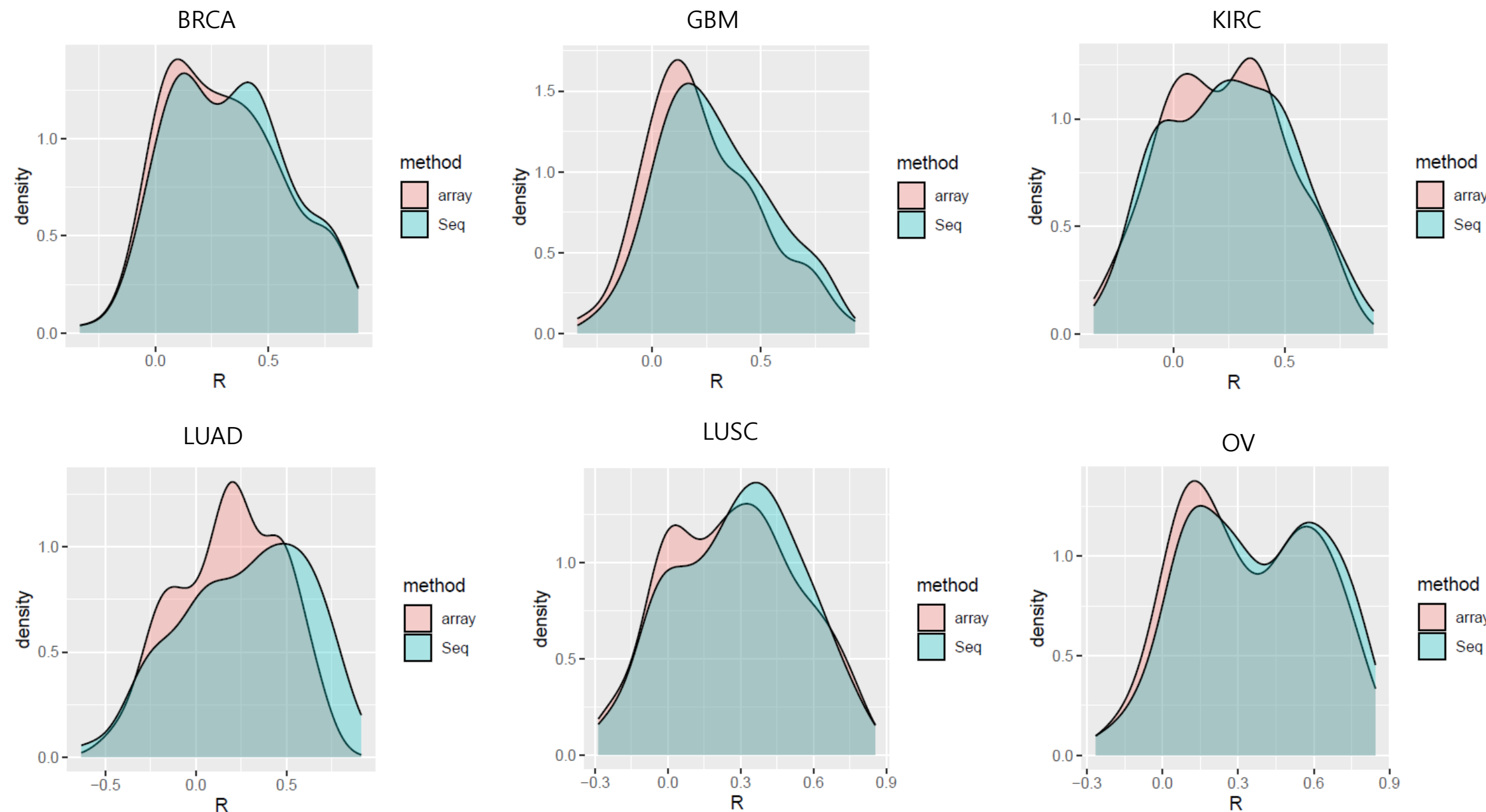

Supplement: Supplementary file 1 [file DataSheet7.PDF]

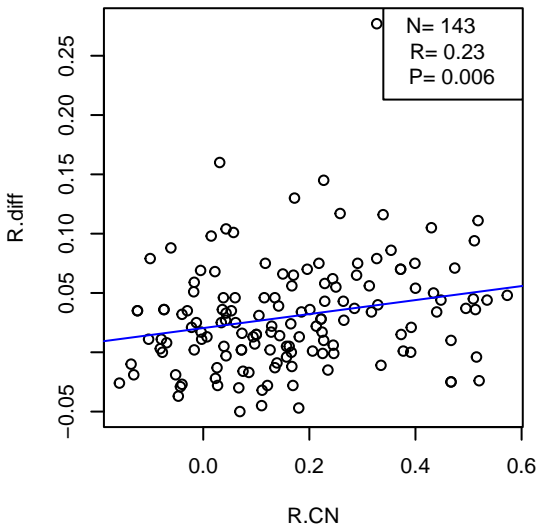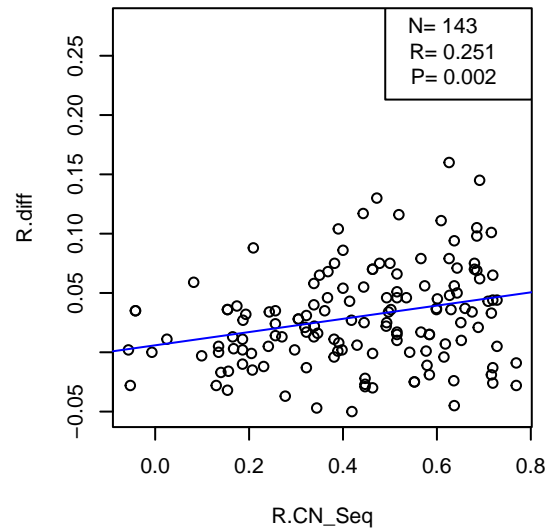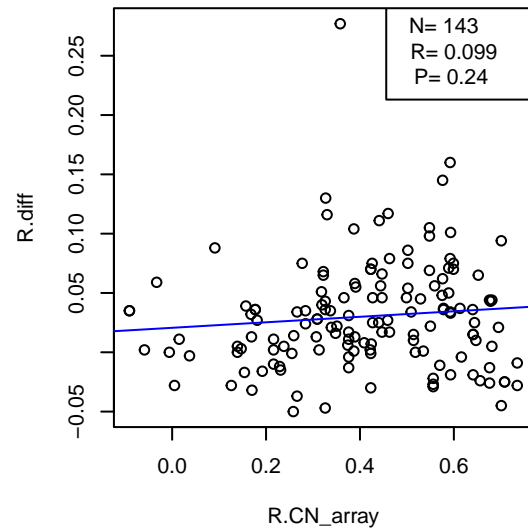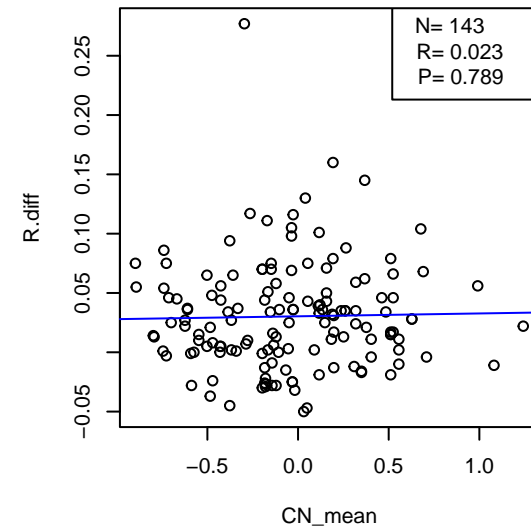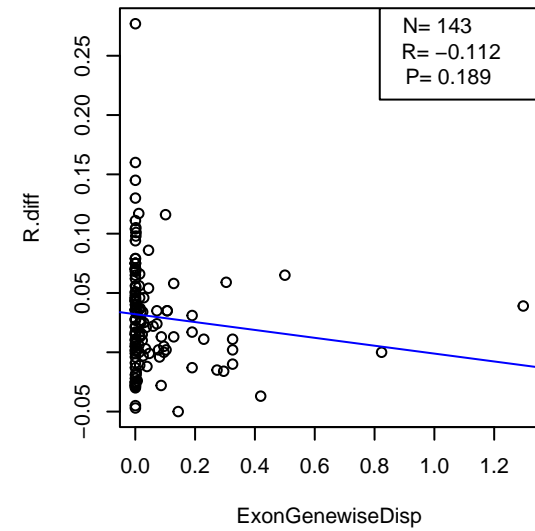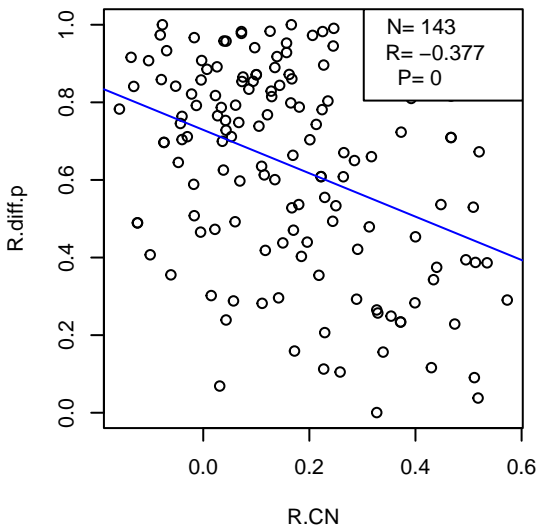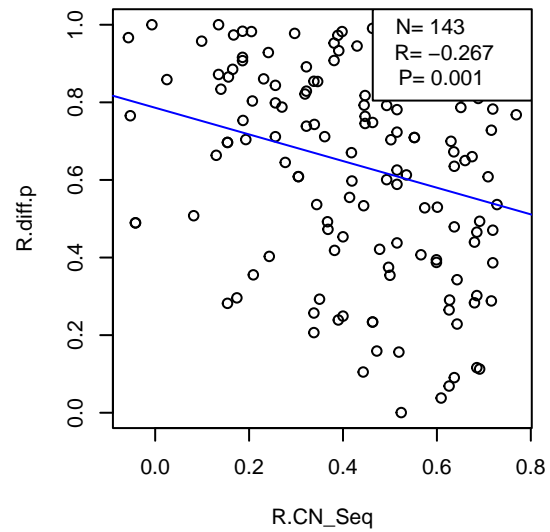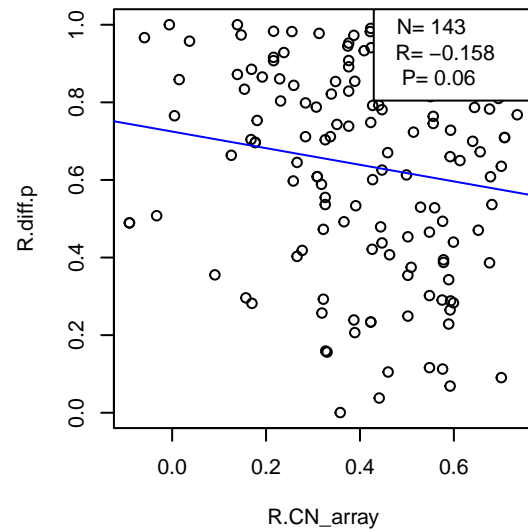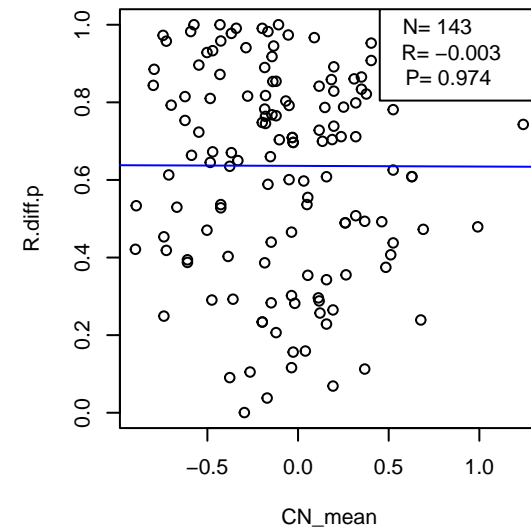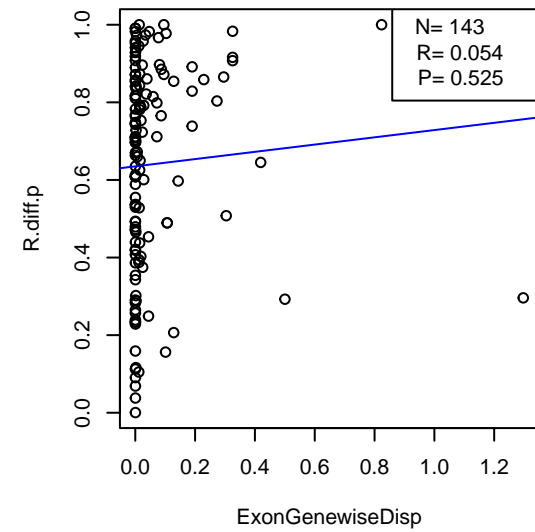

Supplement: Supplementary file 2 [file DataSheet13.PDF]

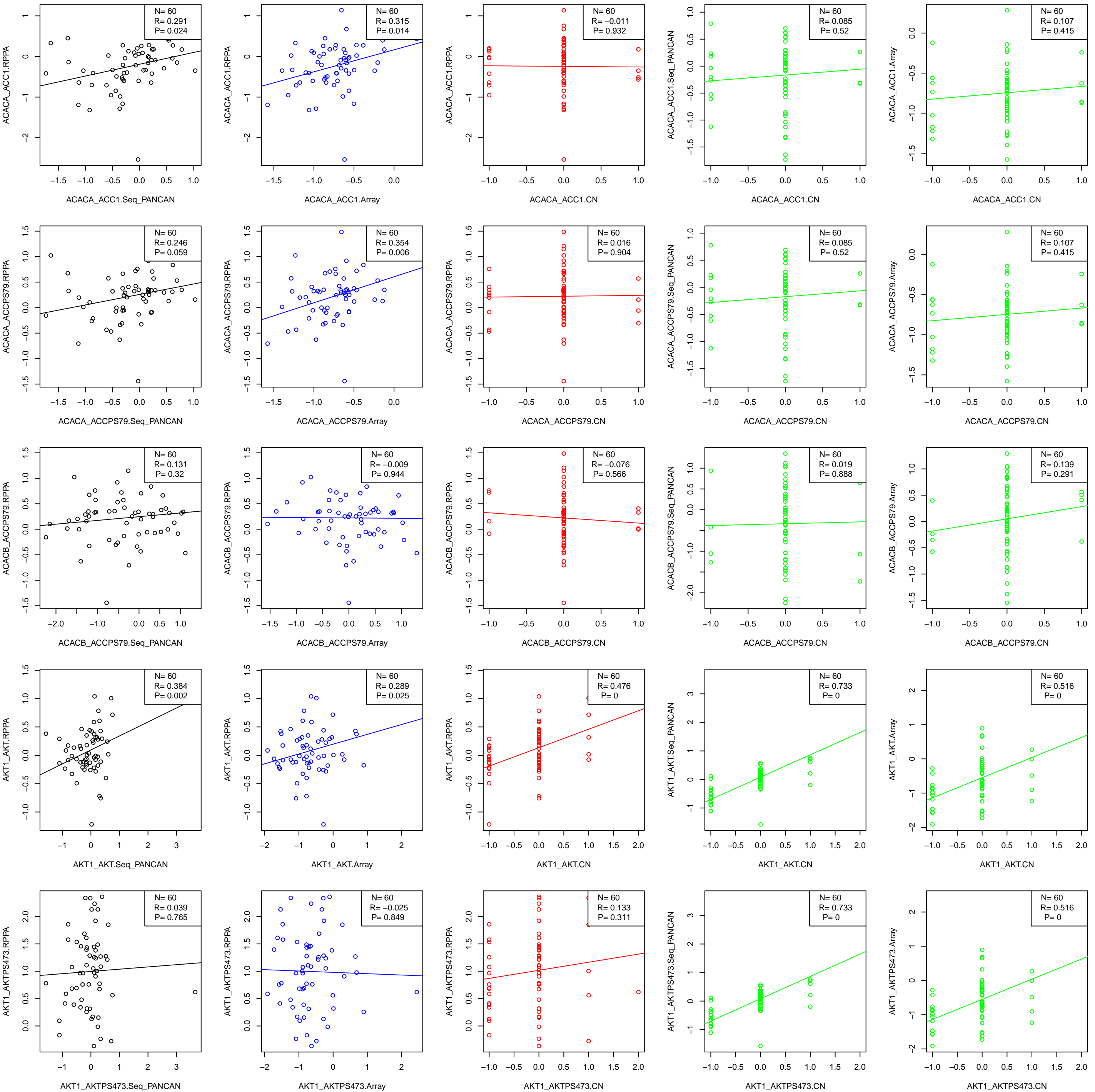

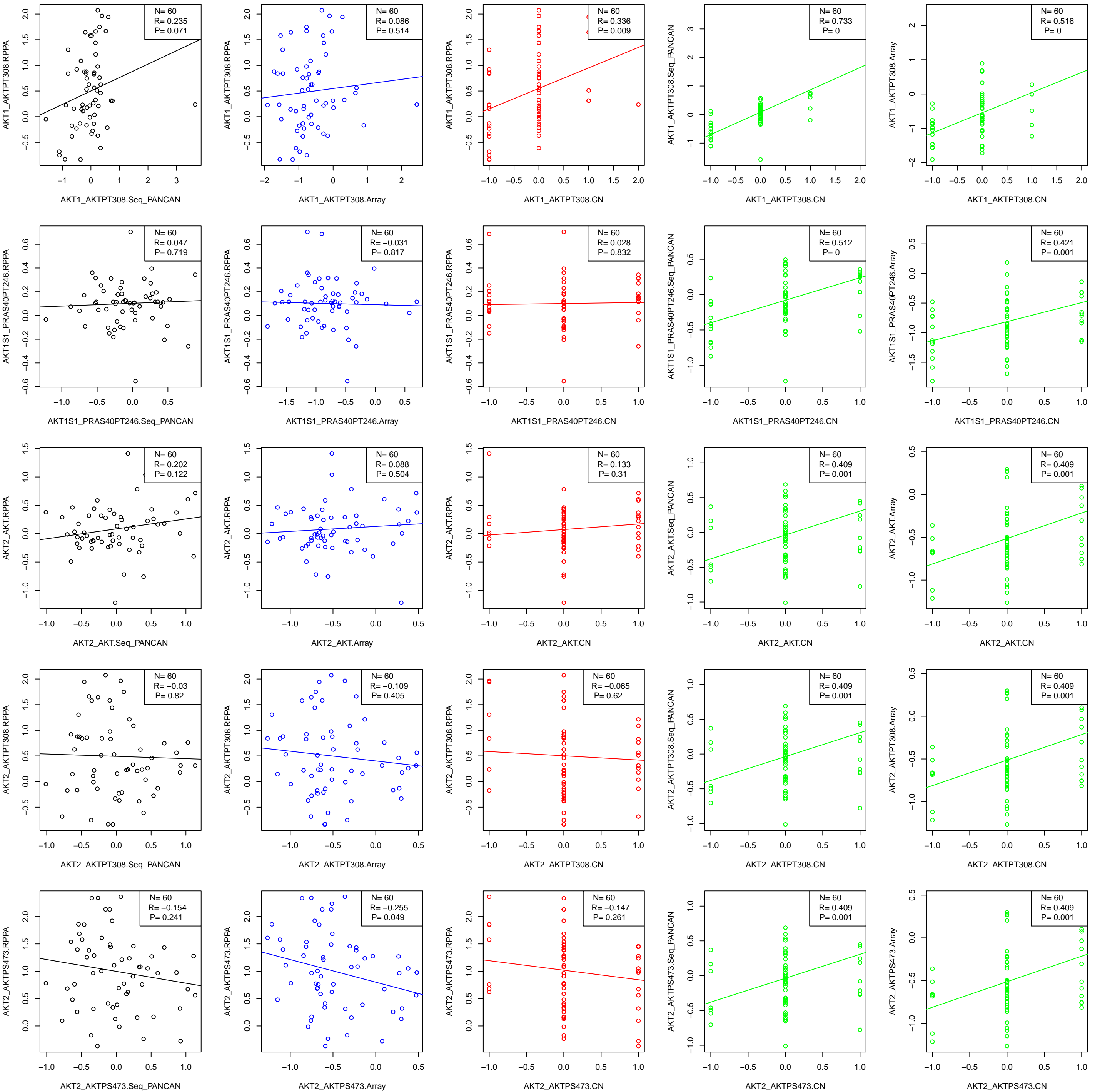

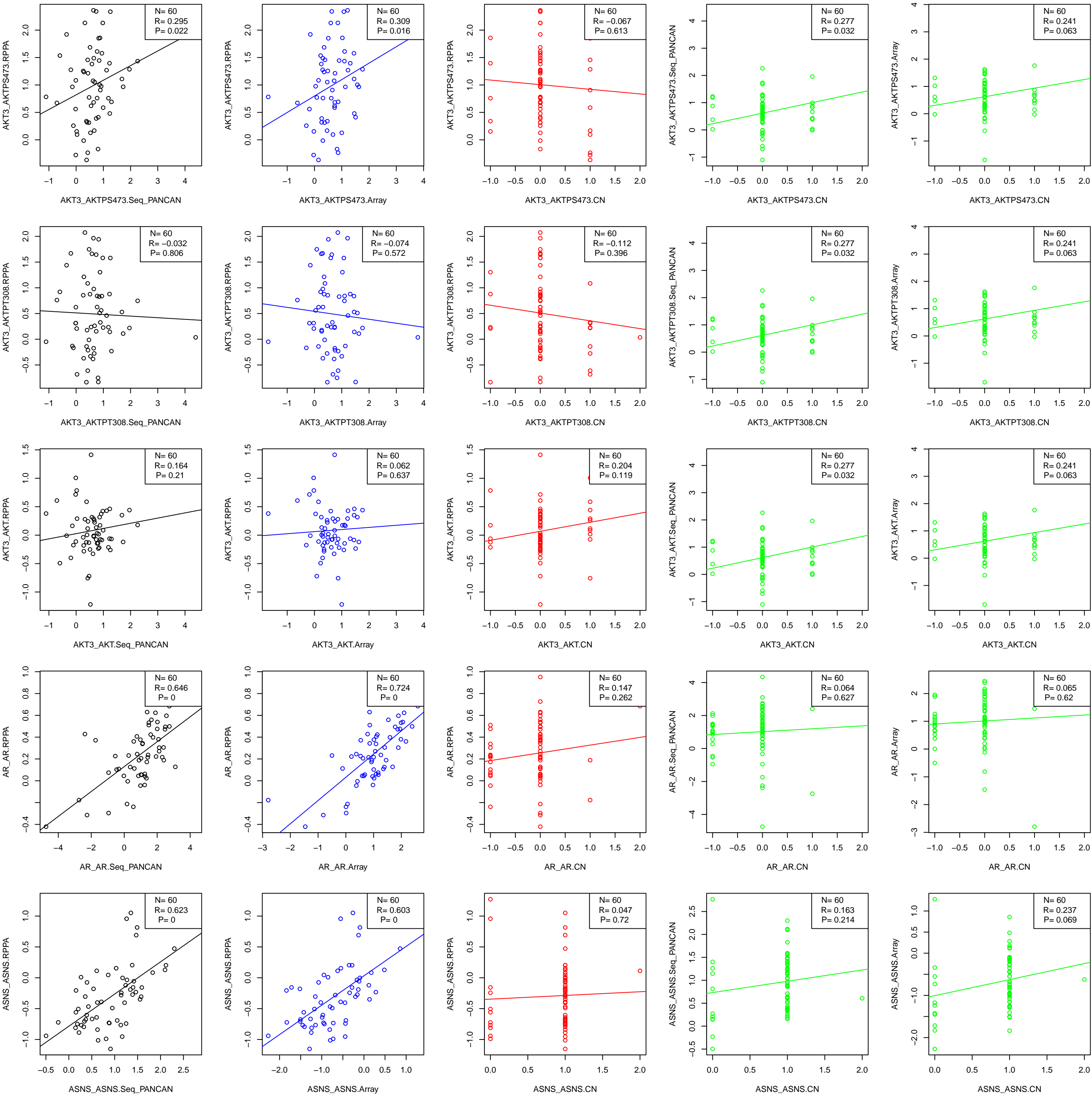

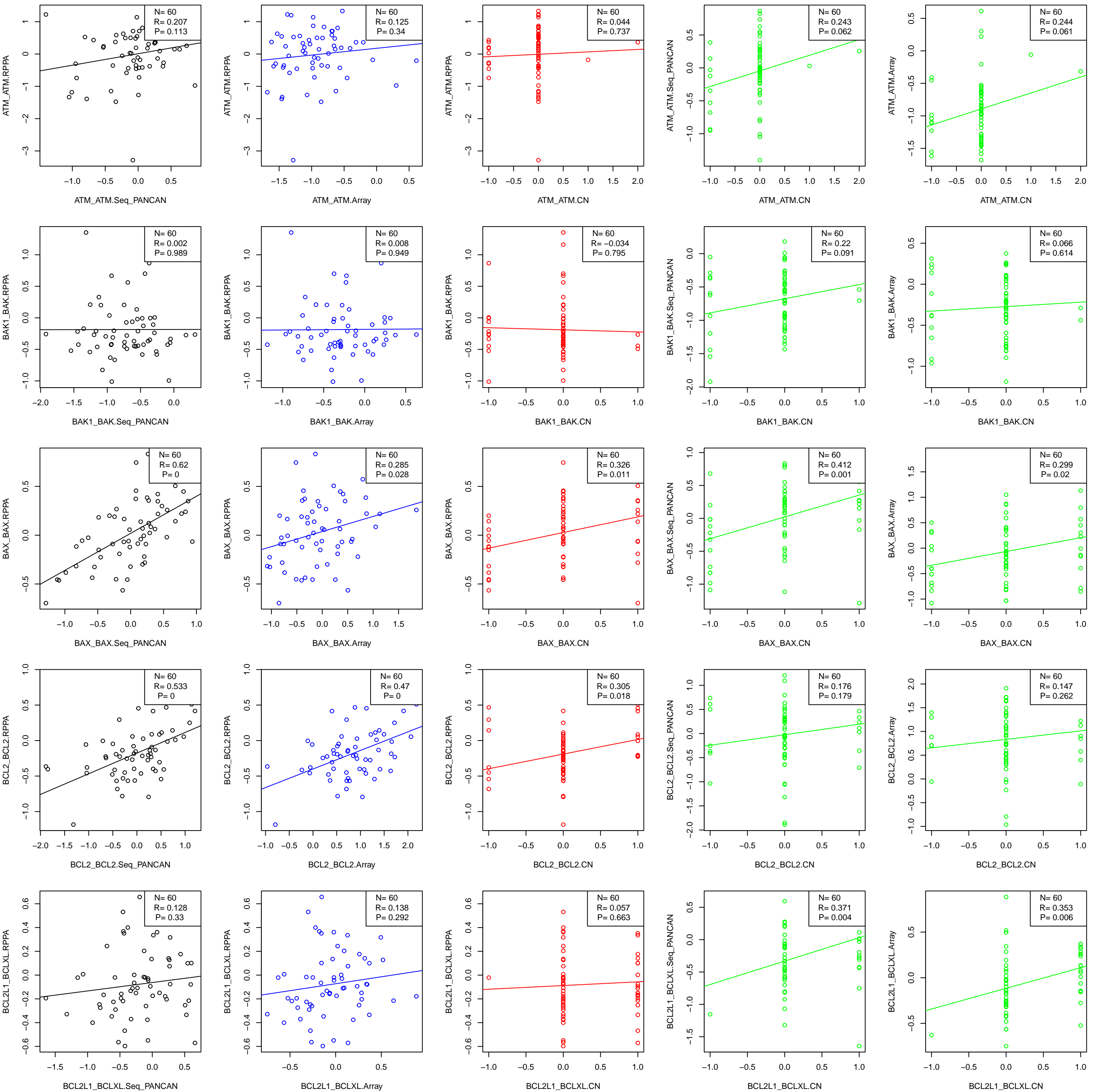



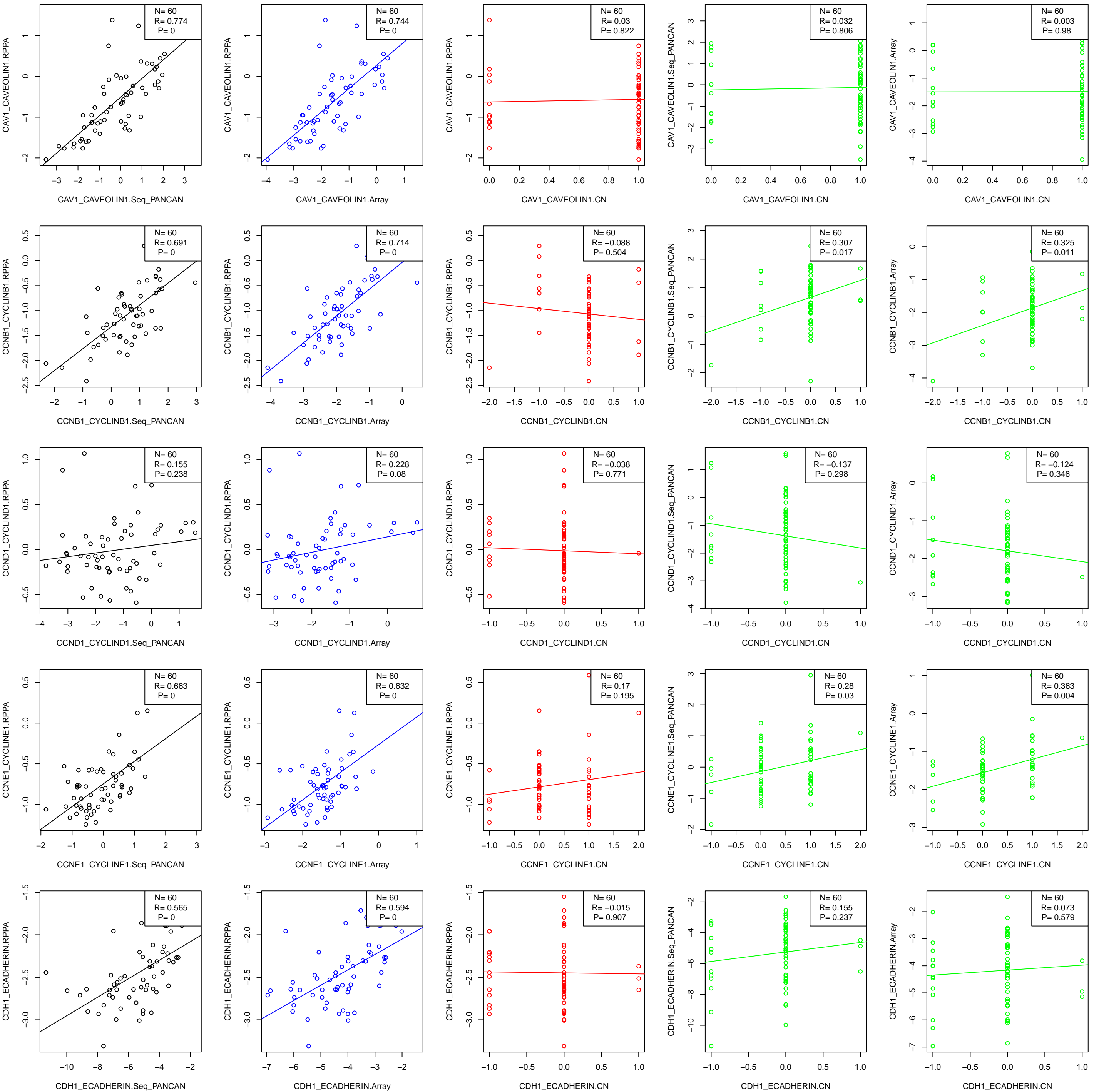

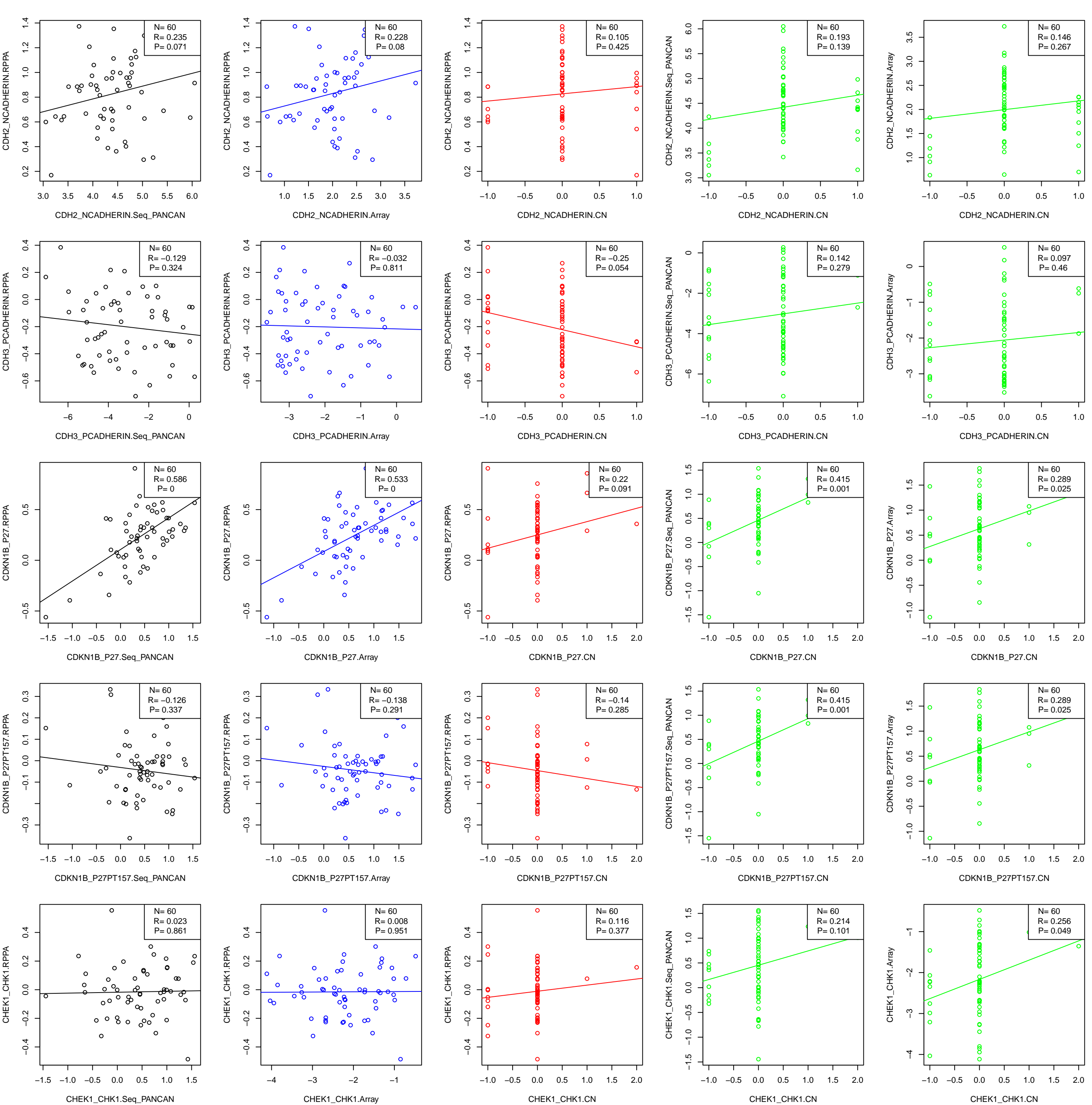

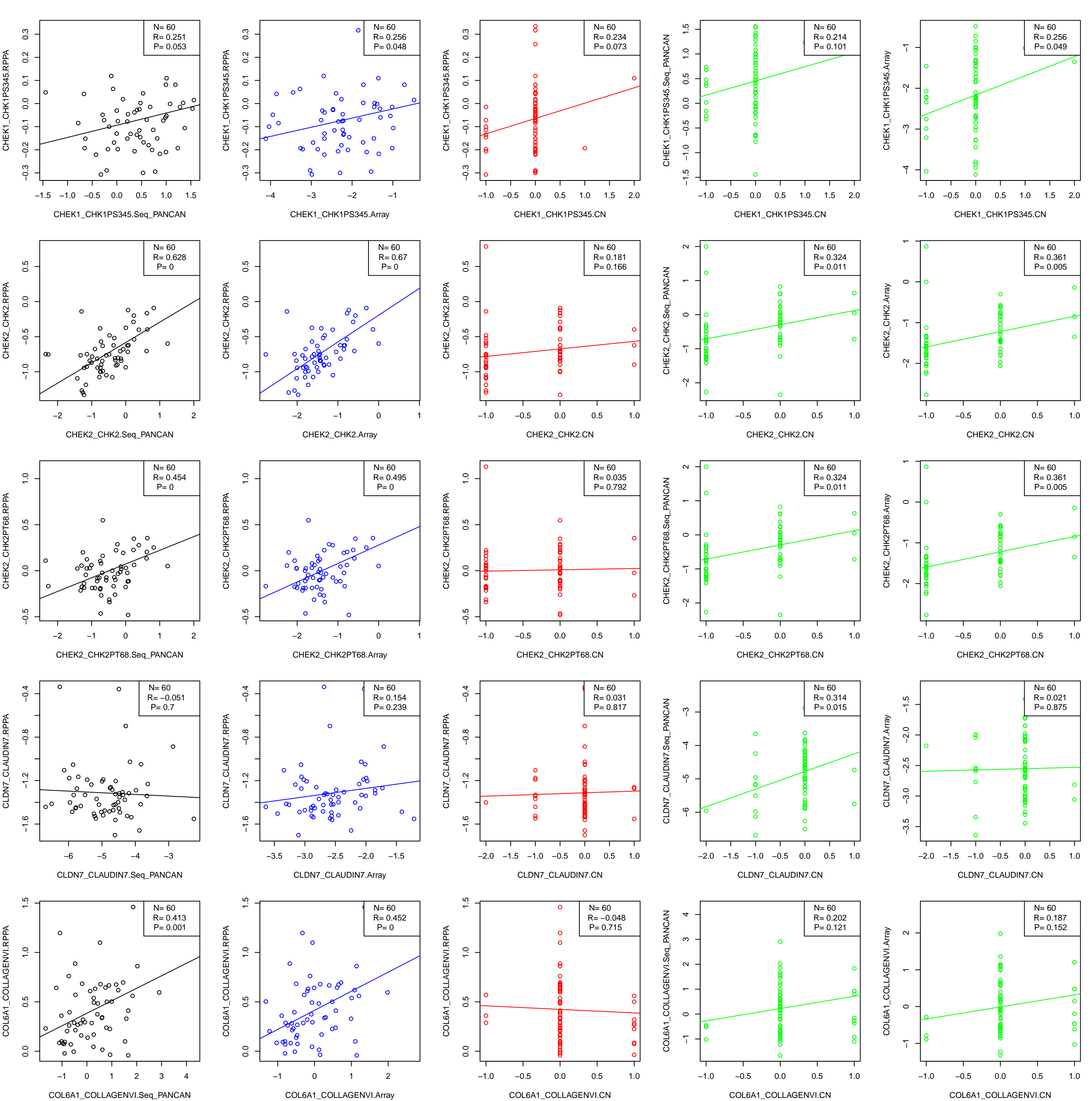

Supplement: Supplementary file 3 [file DataSheet2.PDF]

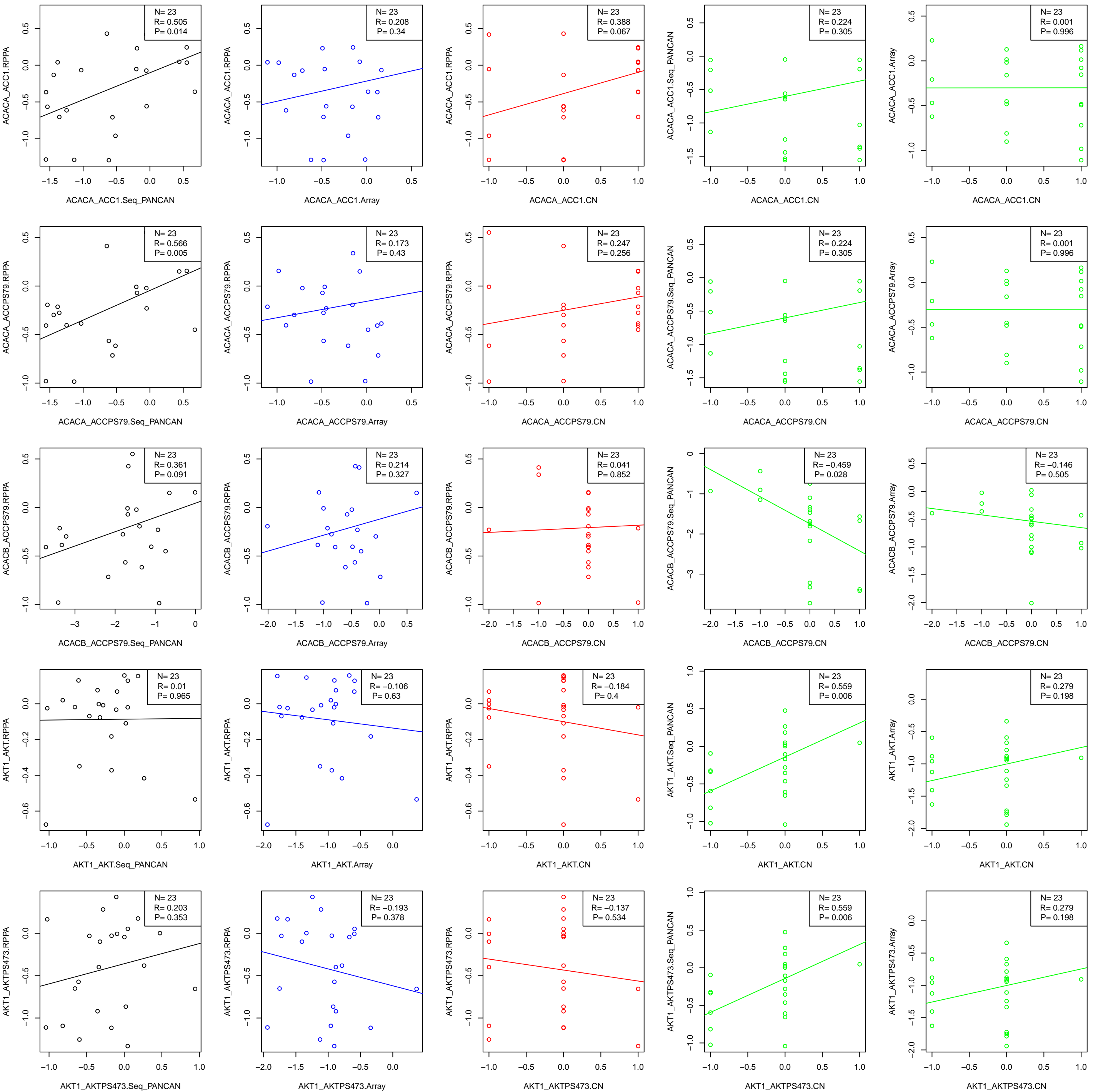

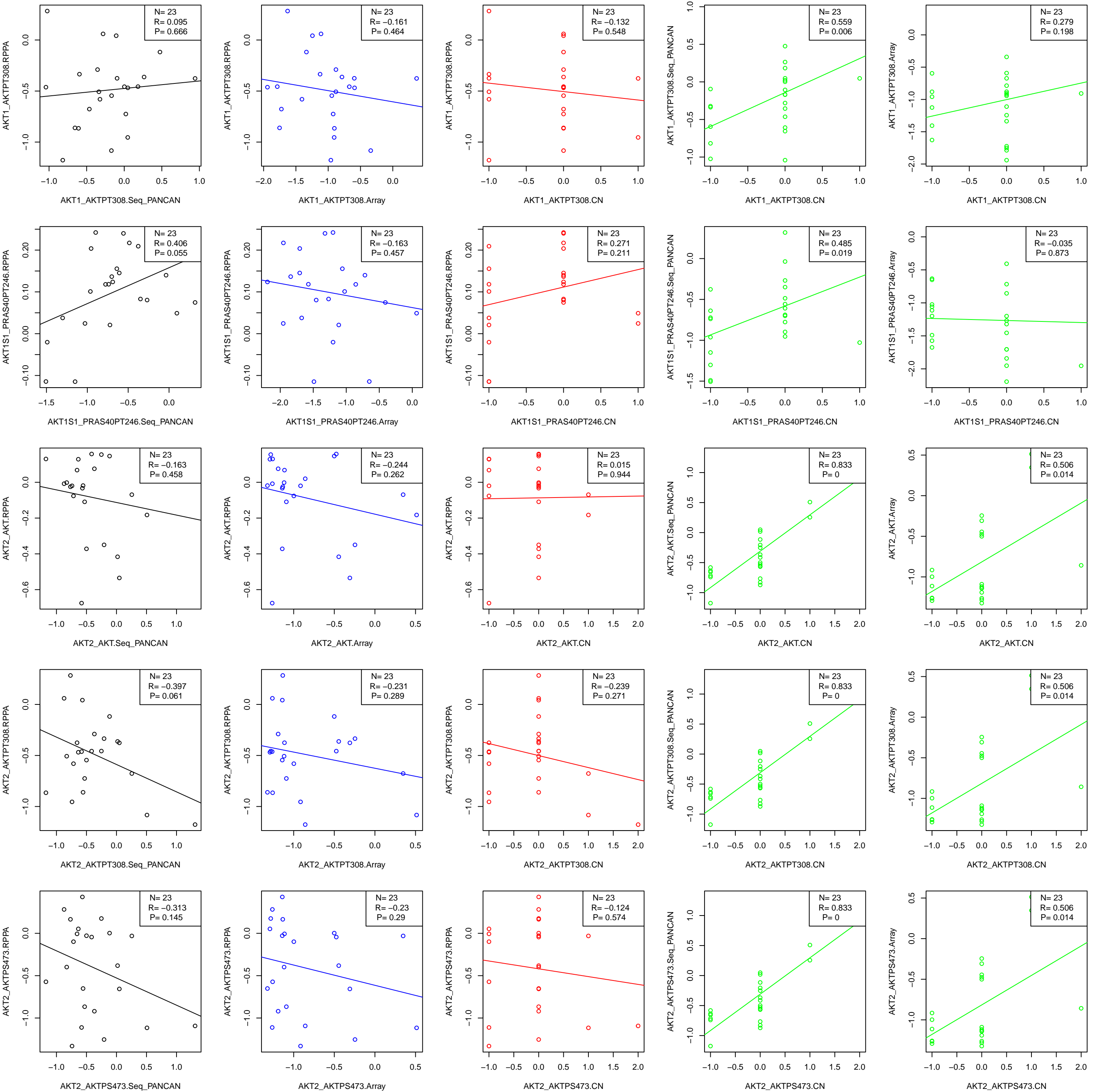

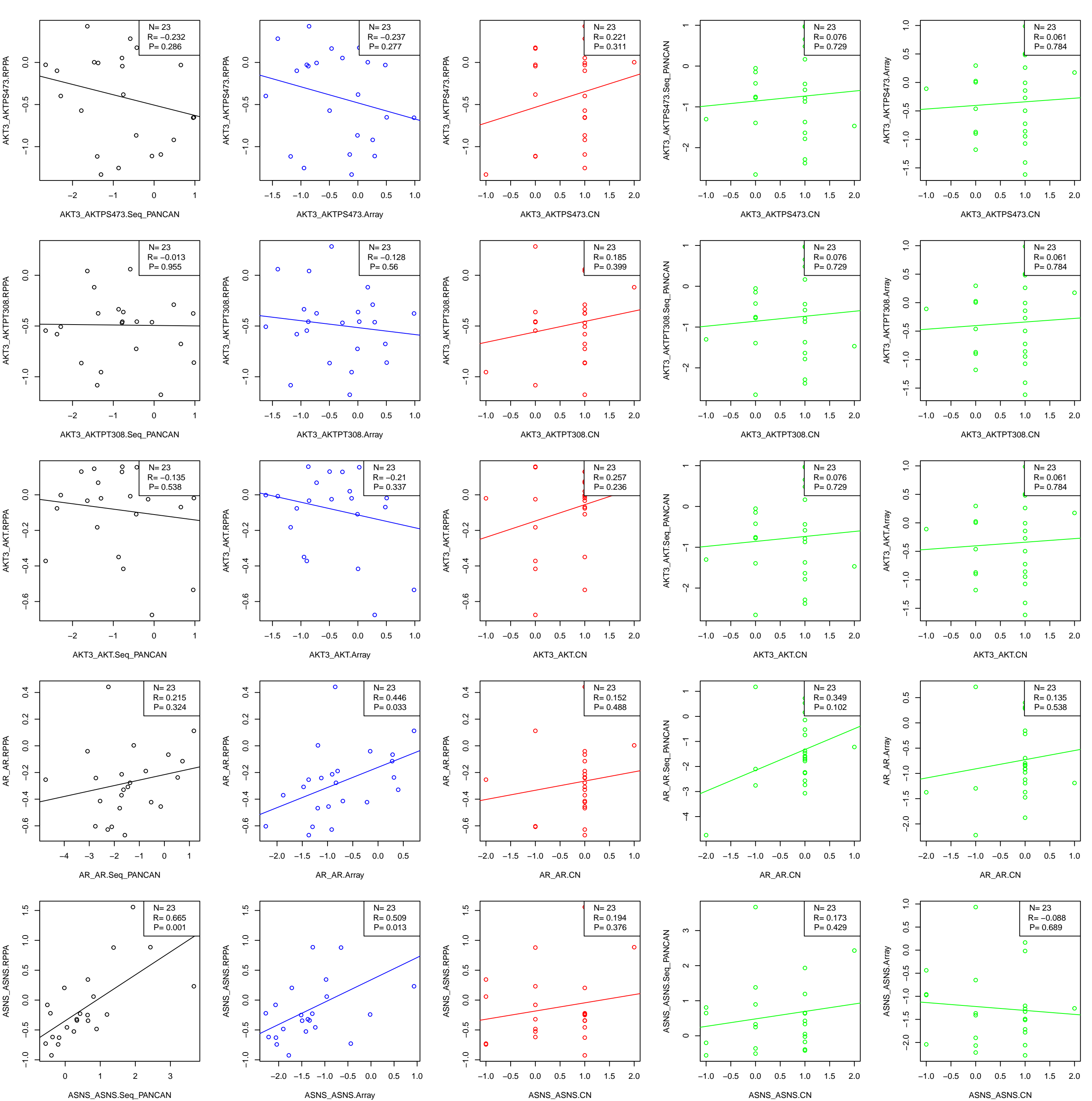

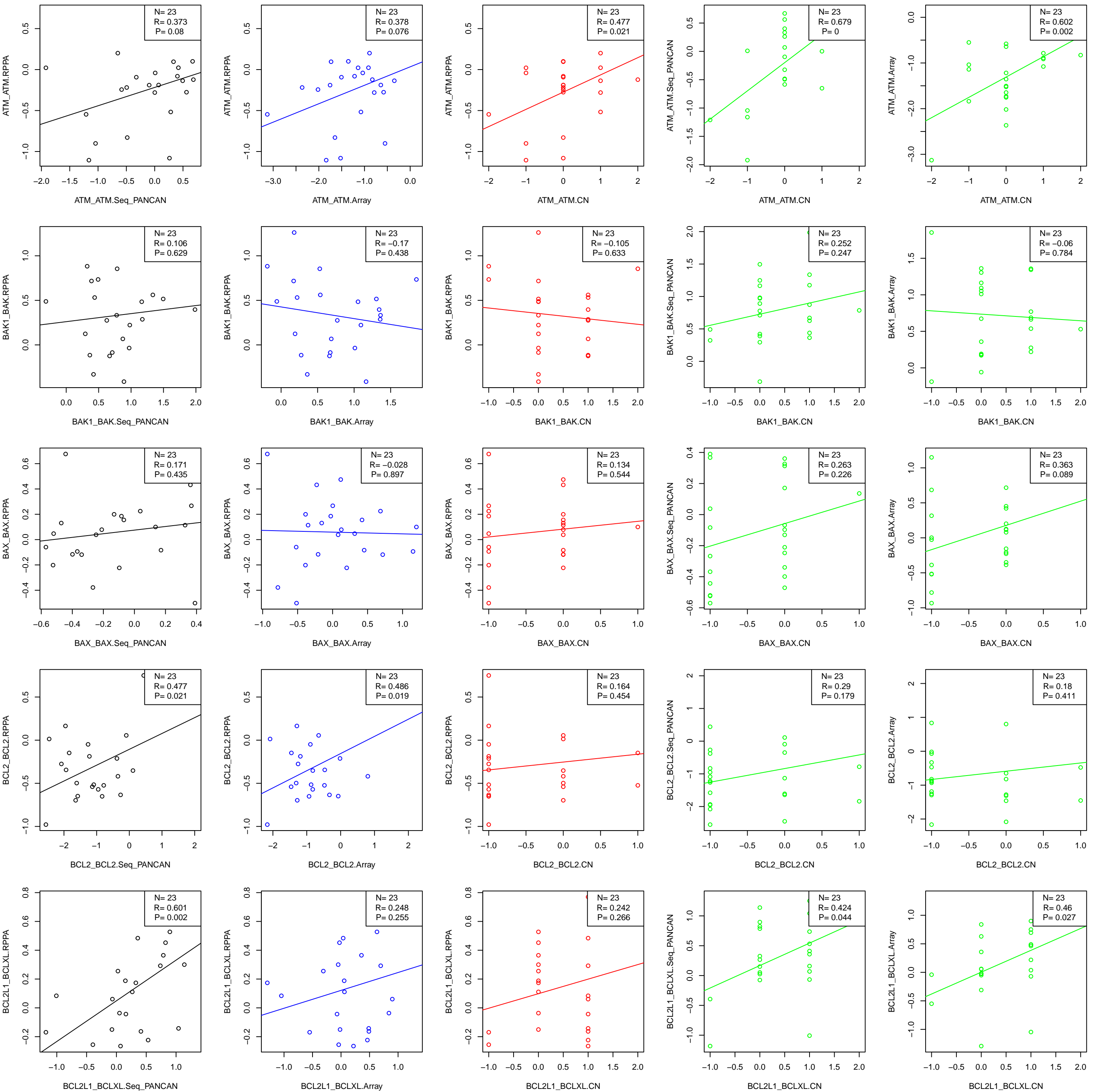

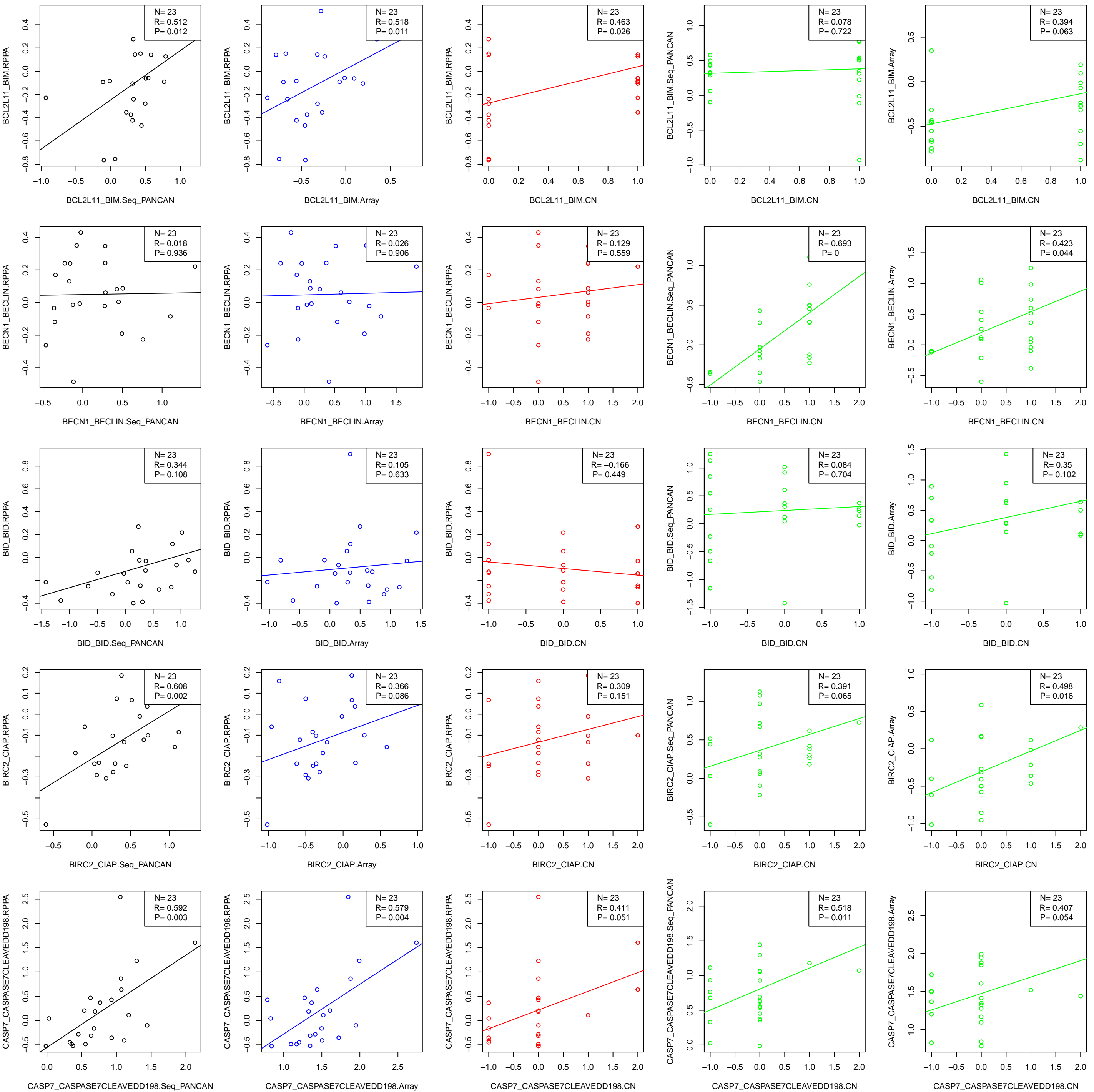

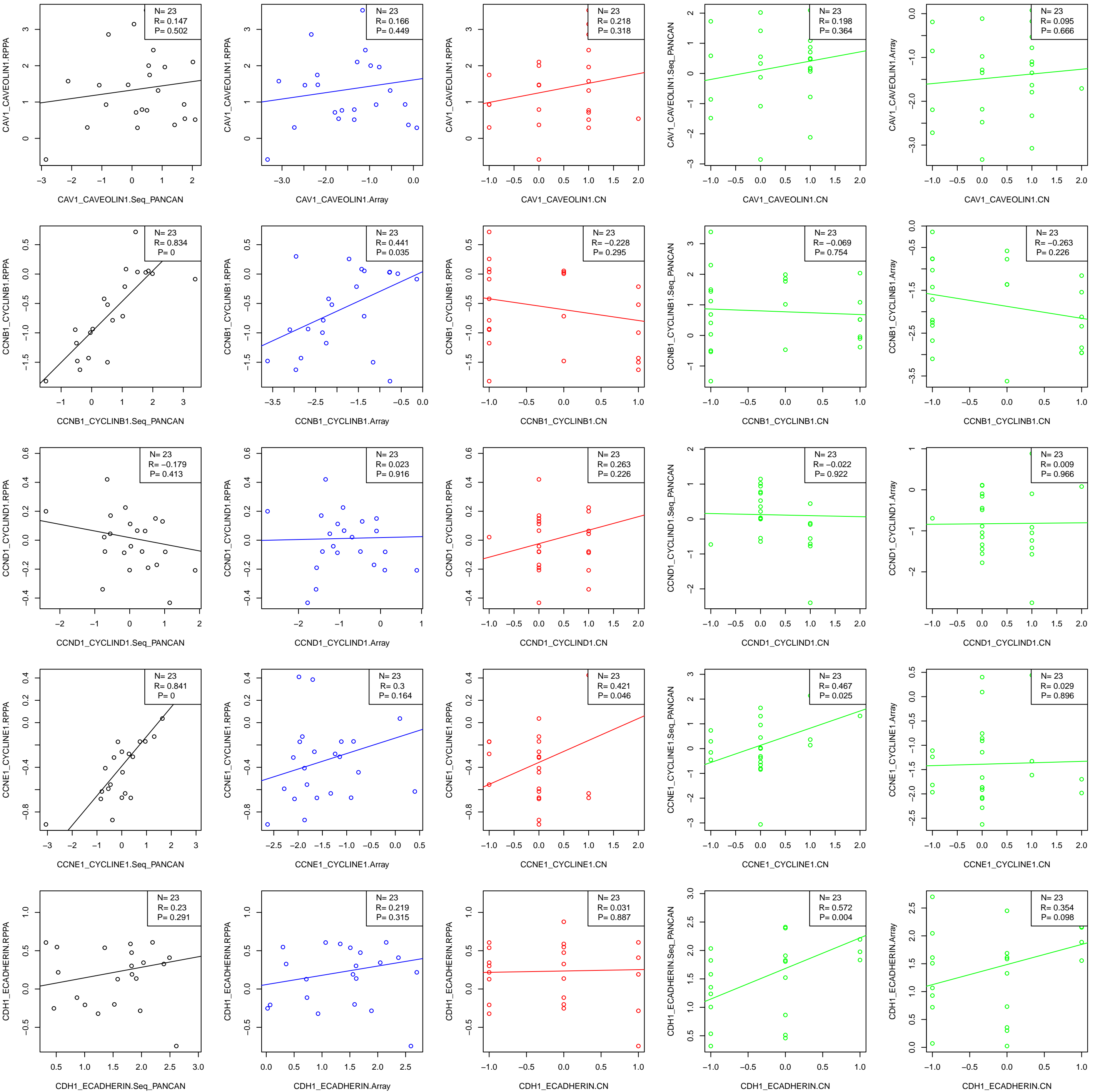

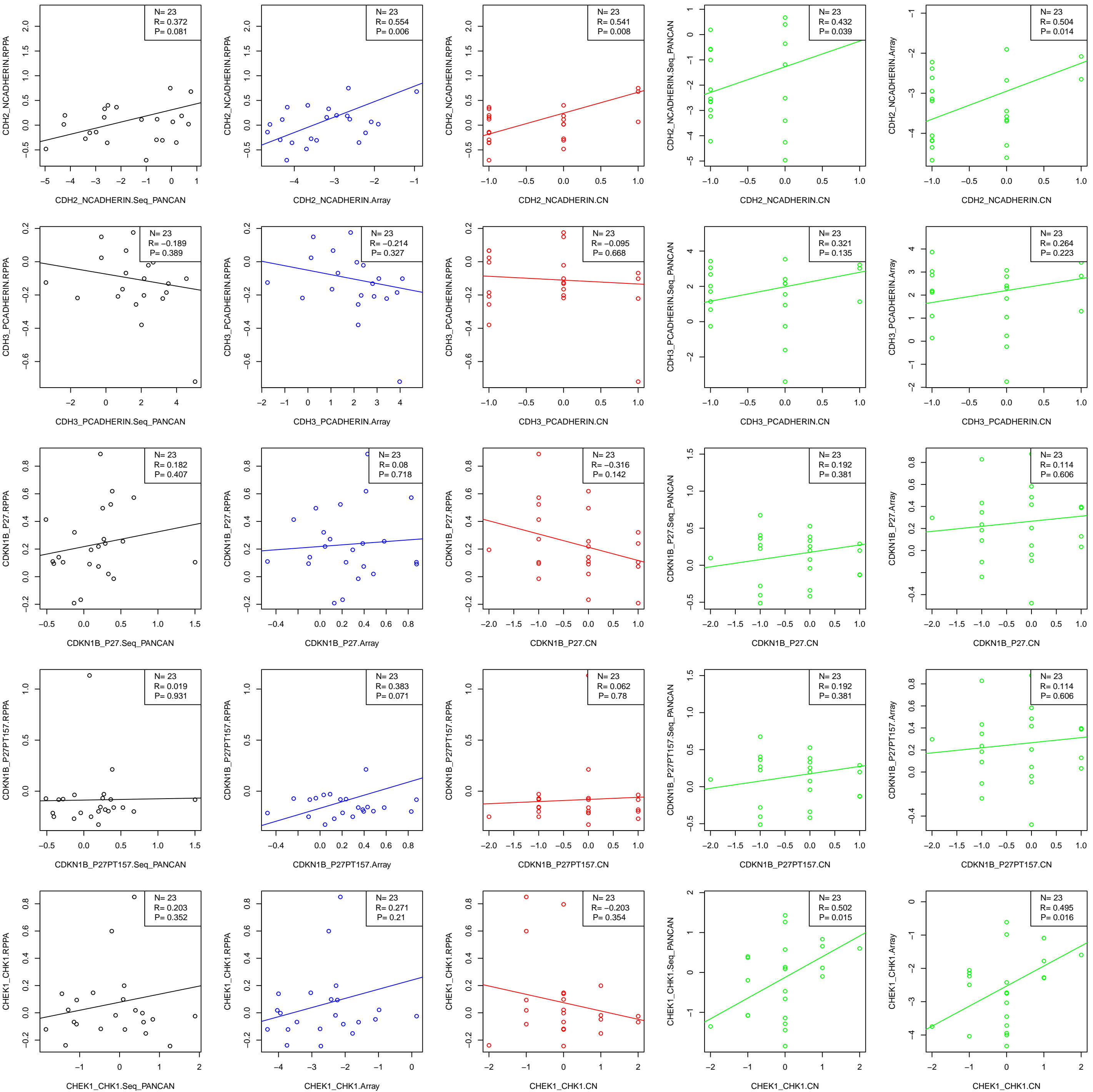

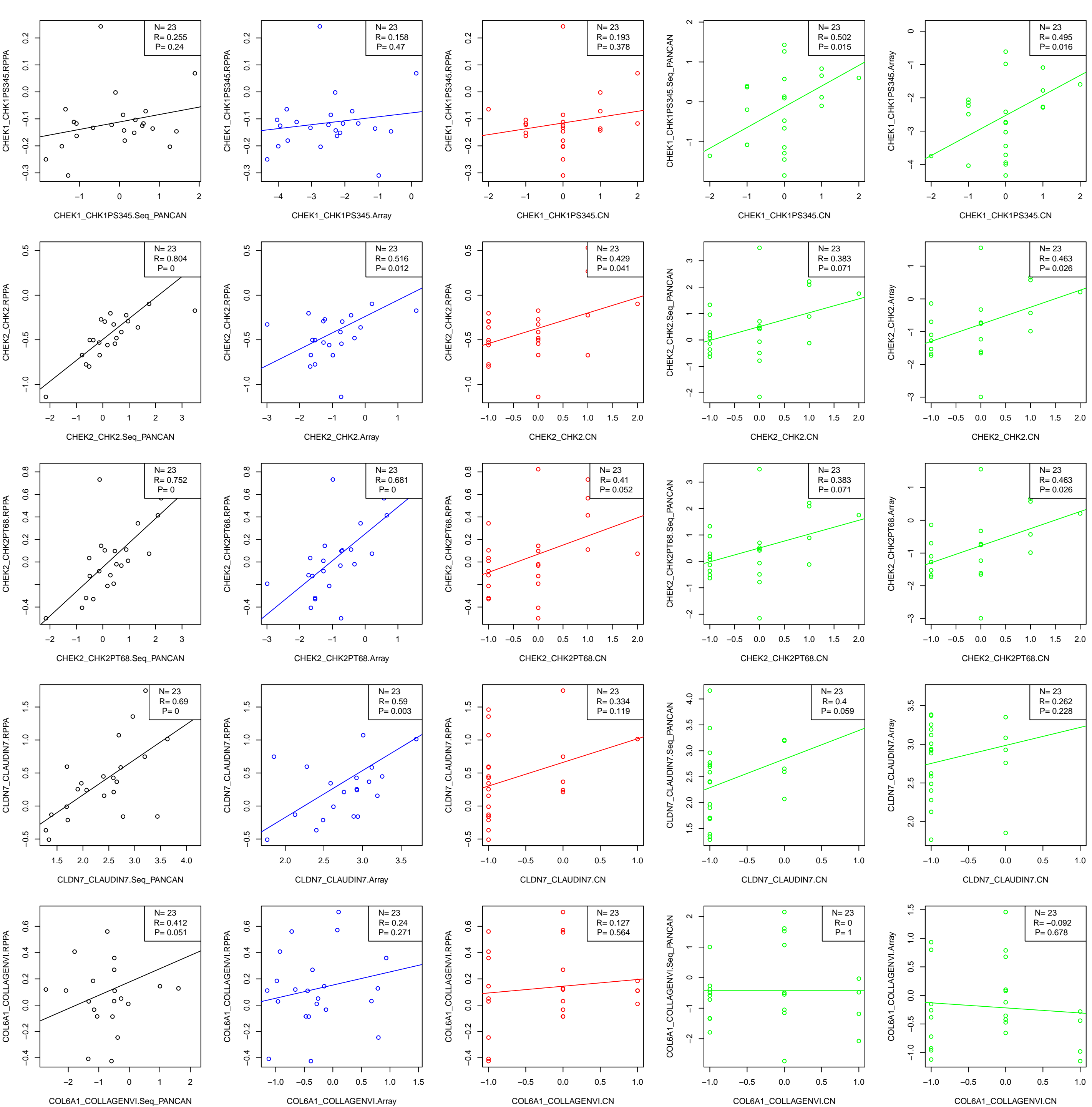

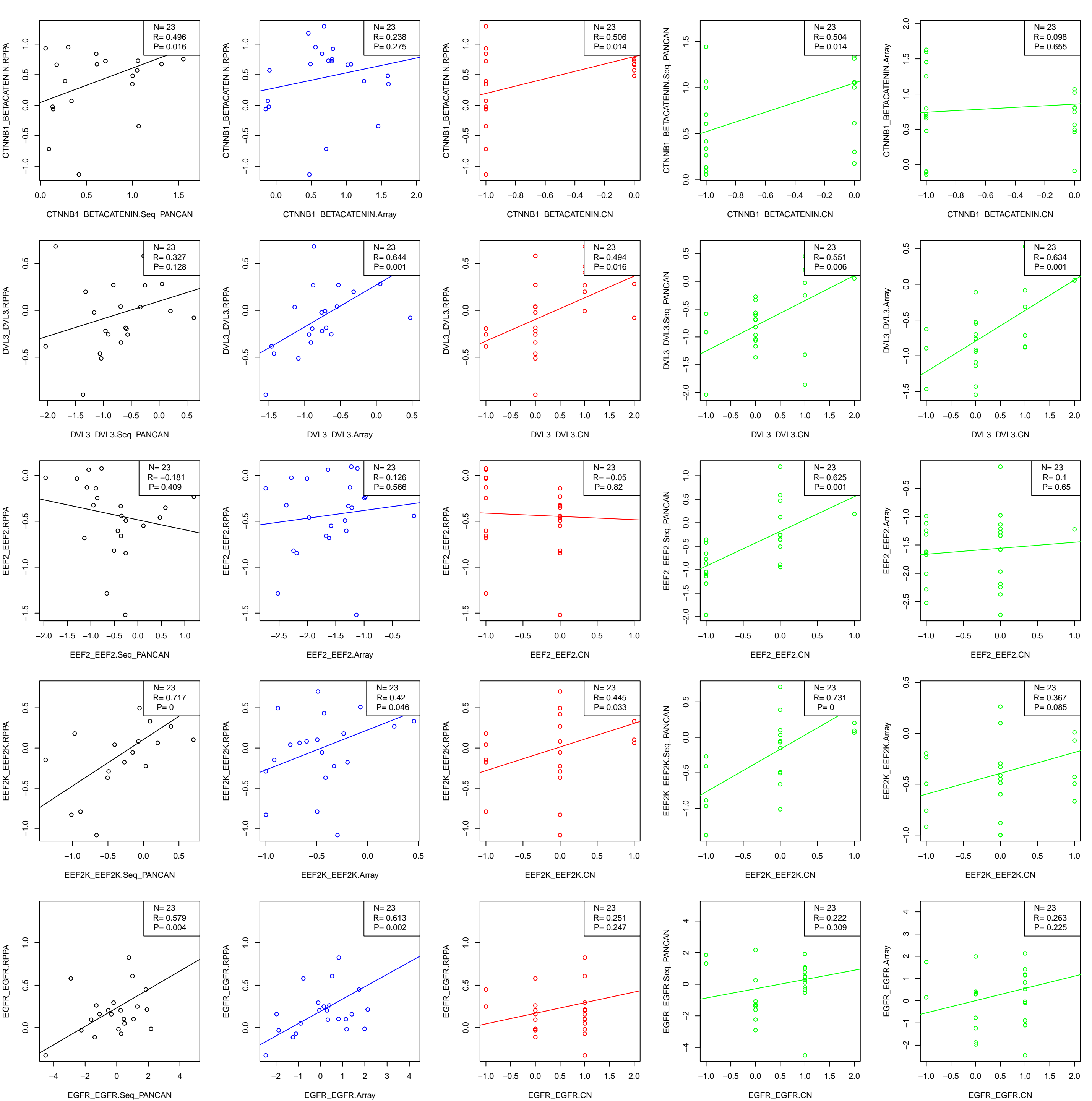

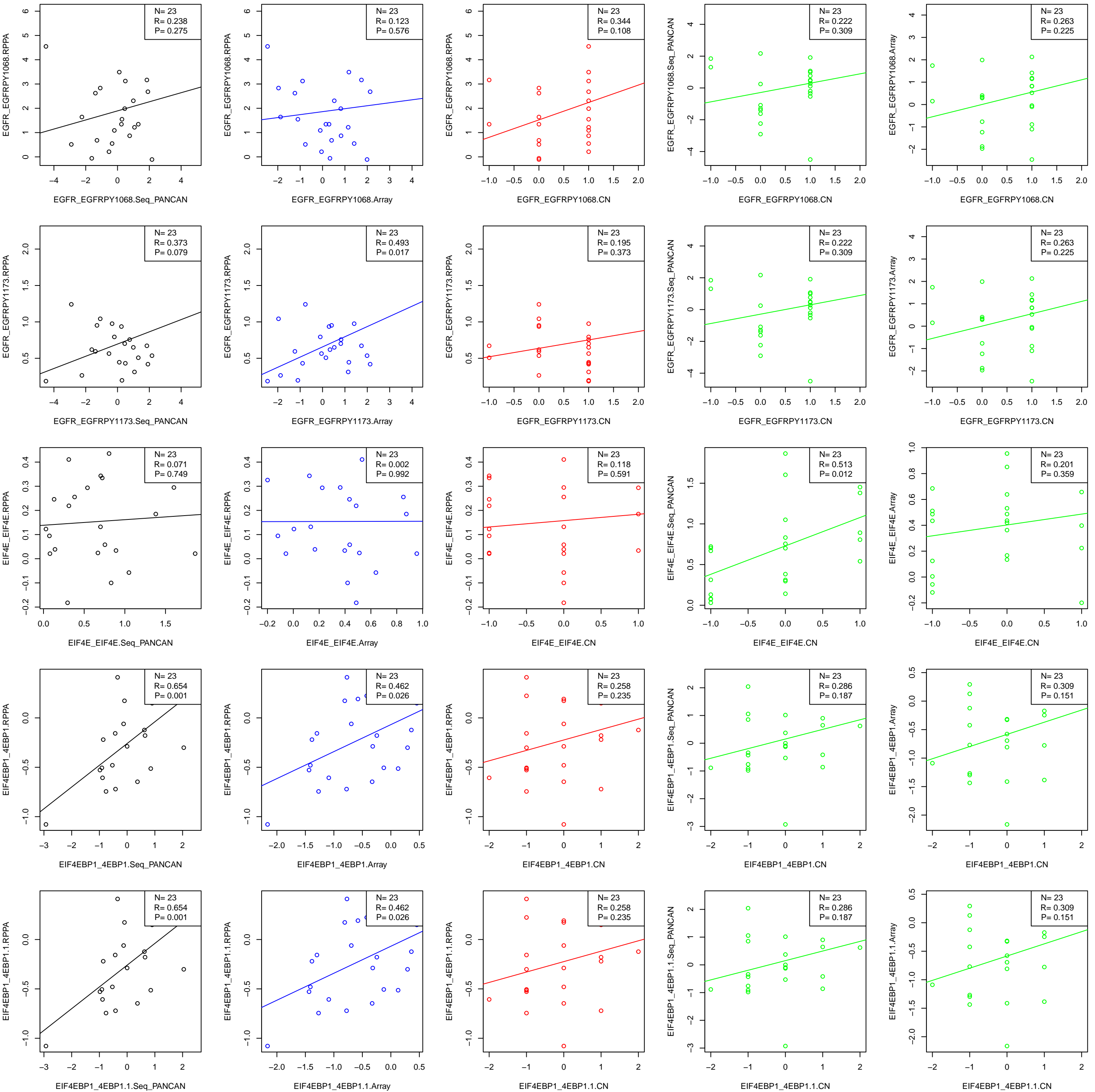

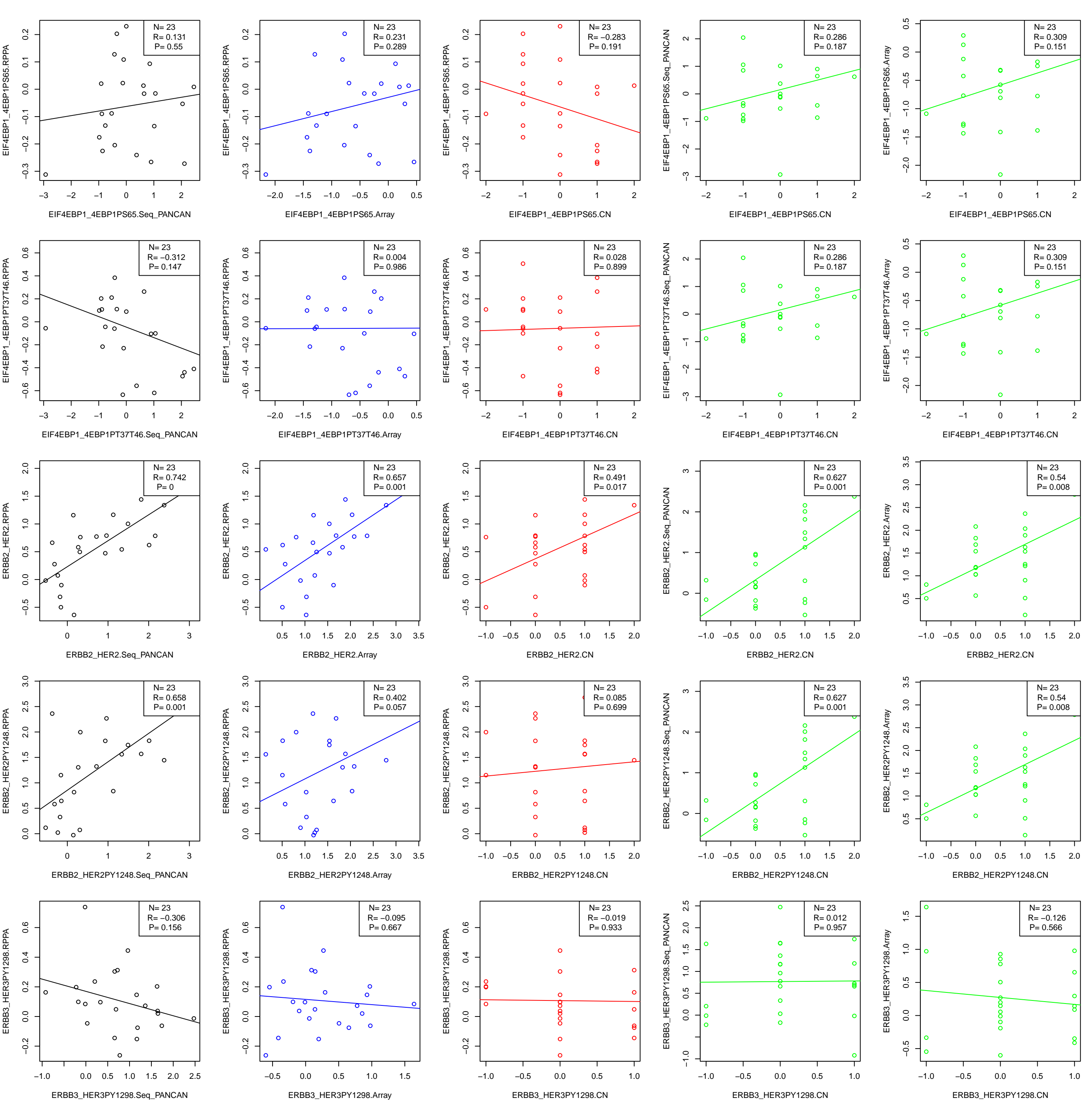

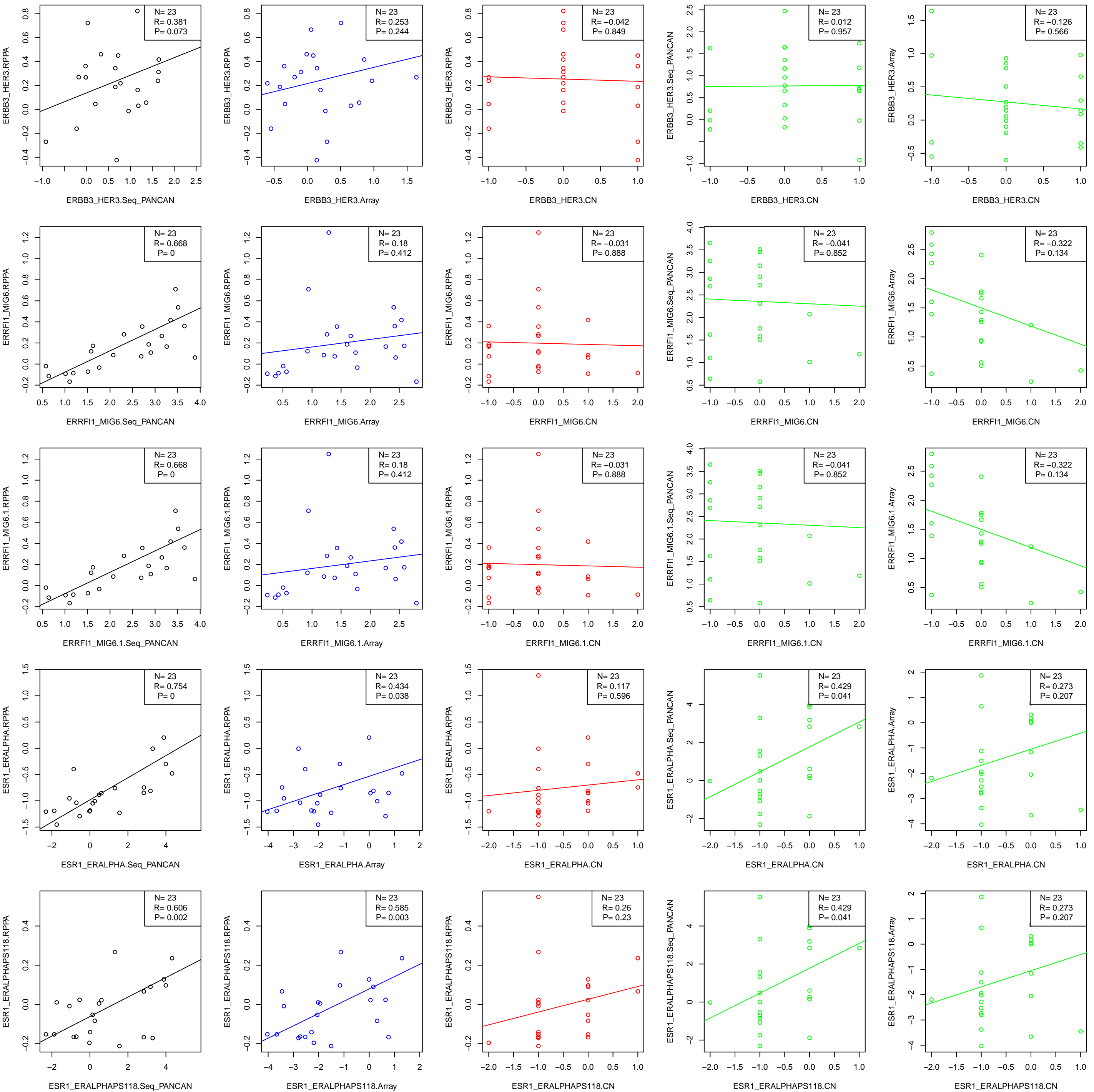

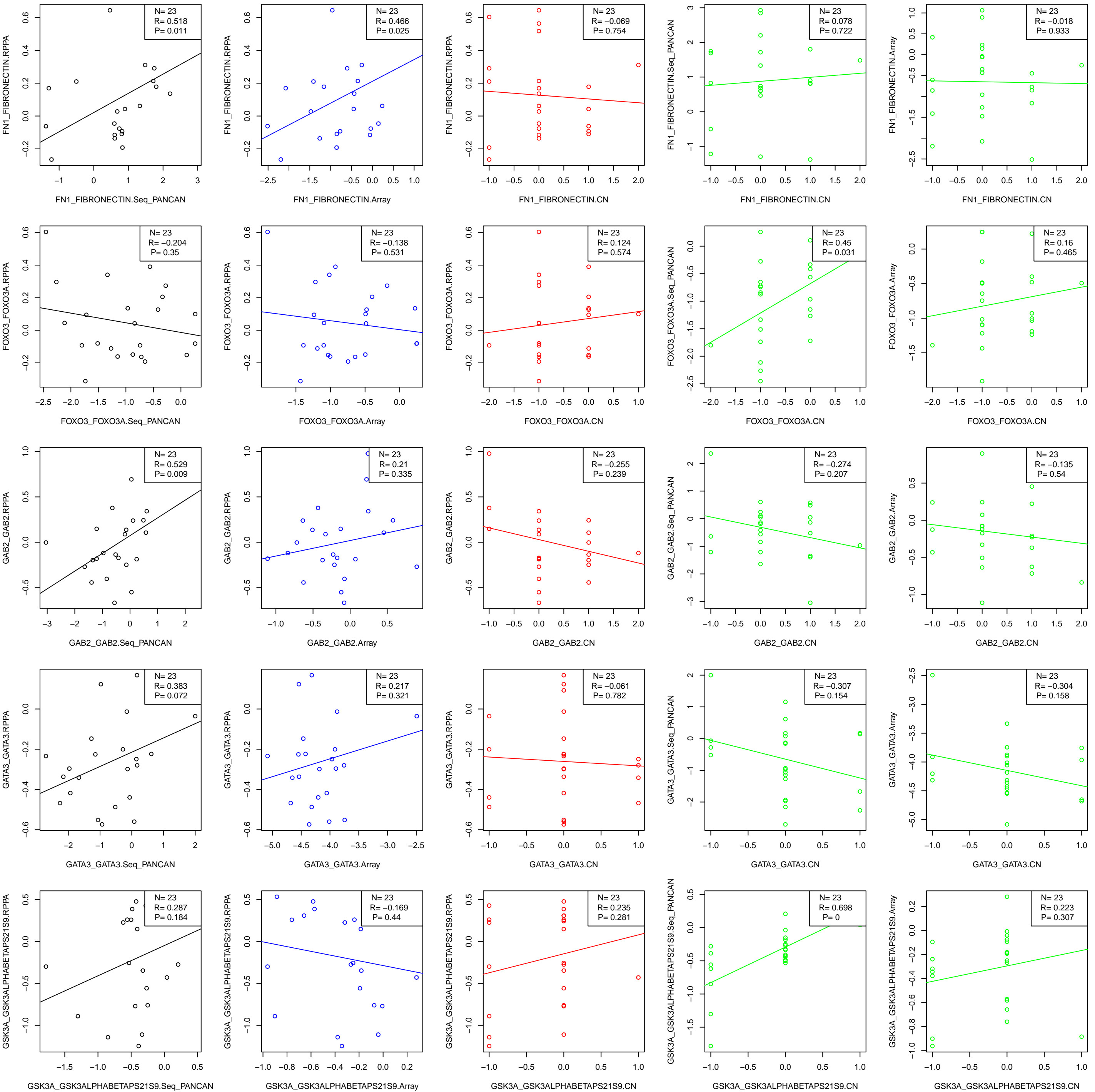

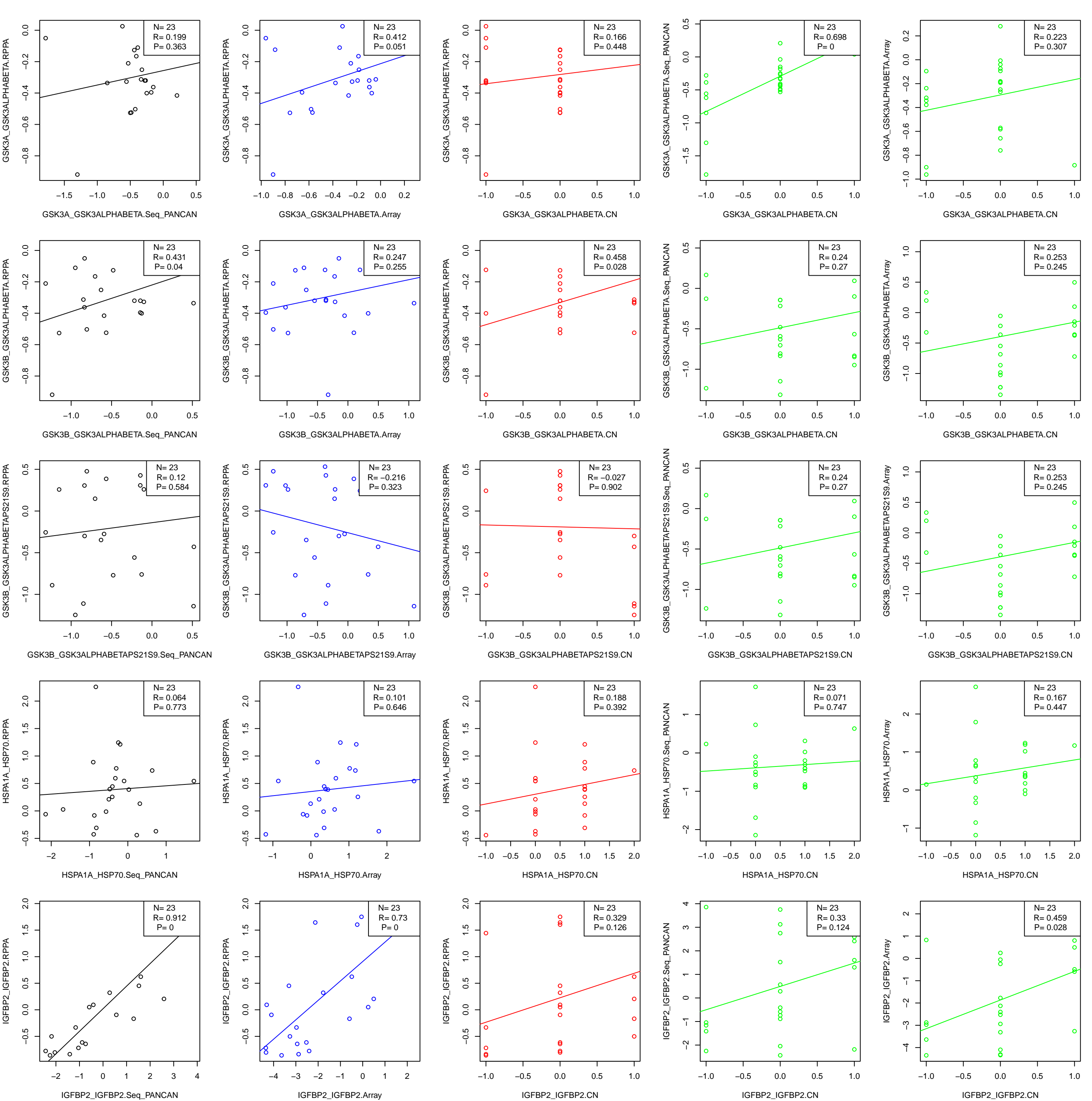

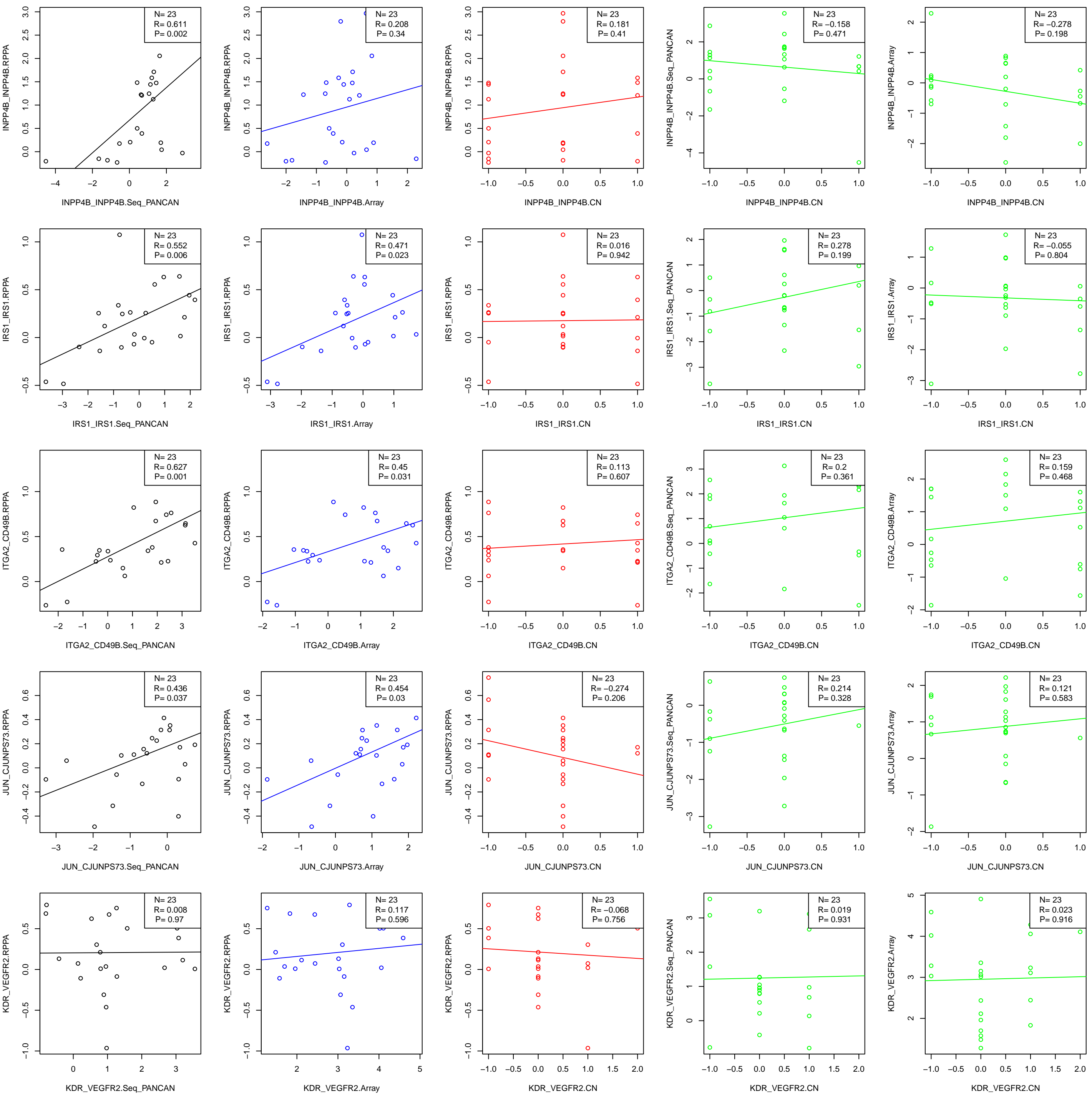

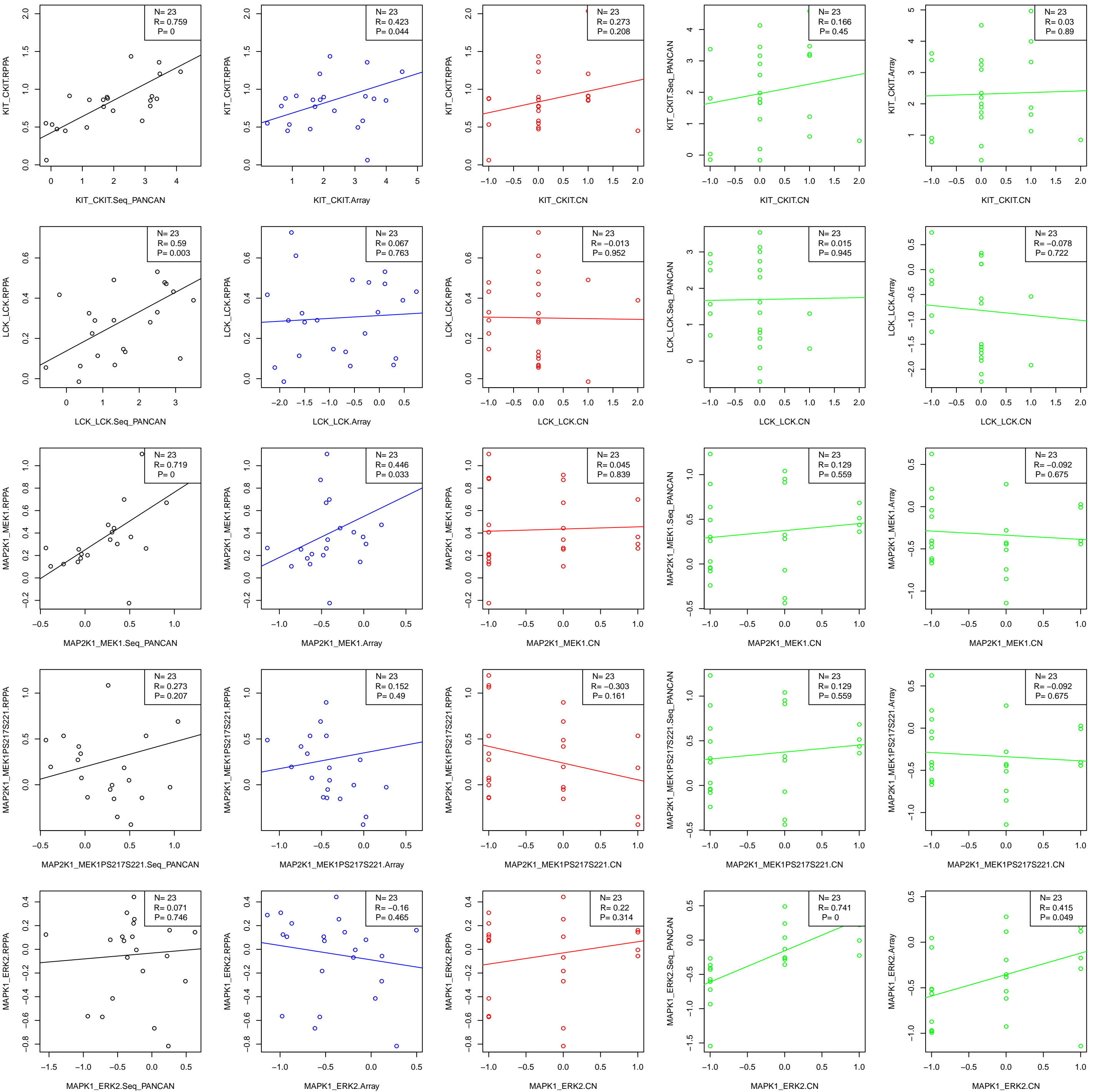

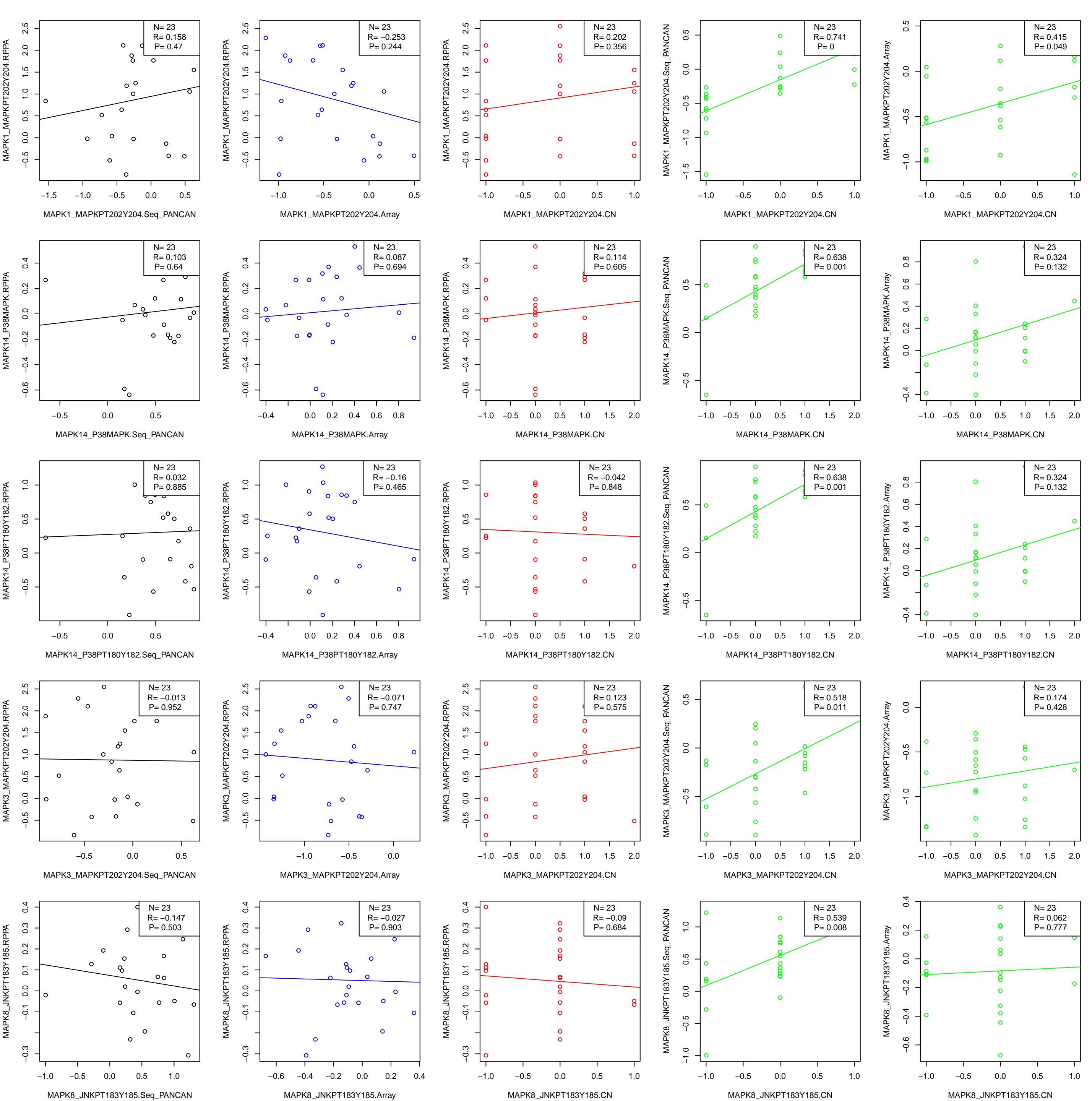

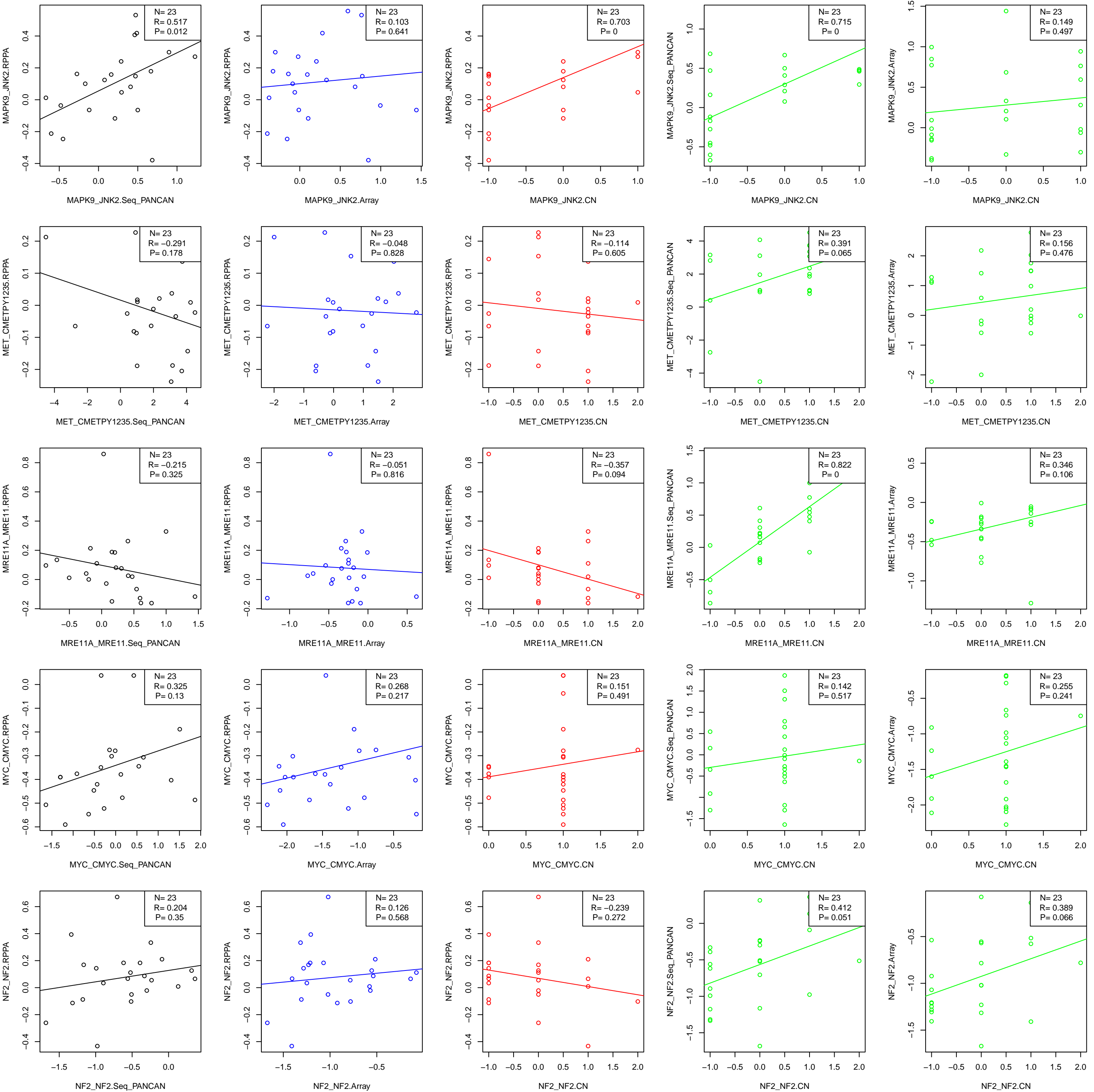

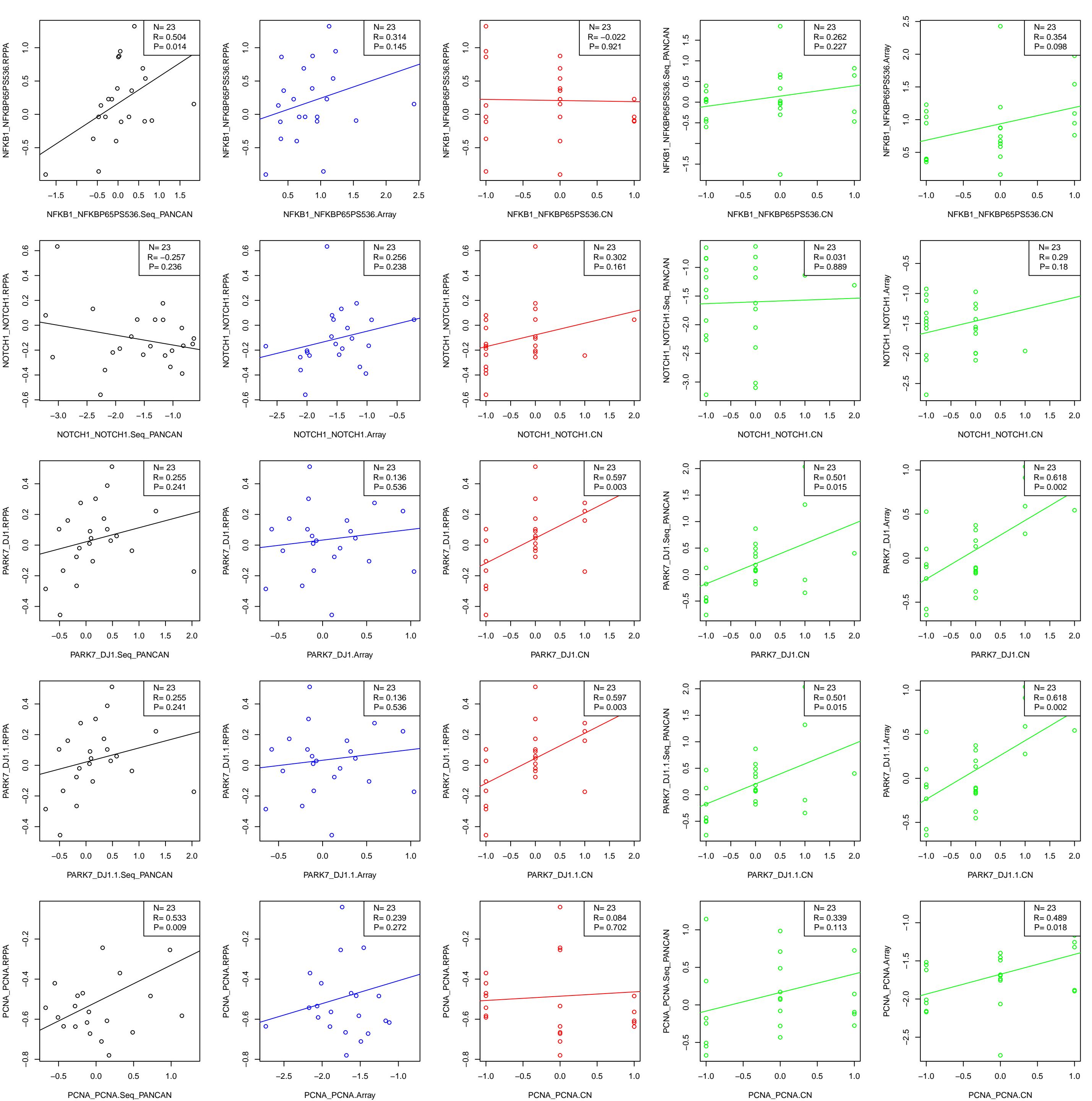

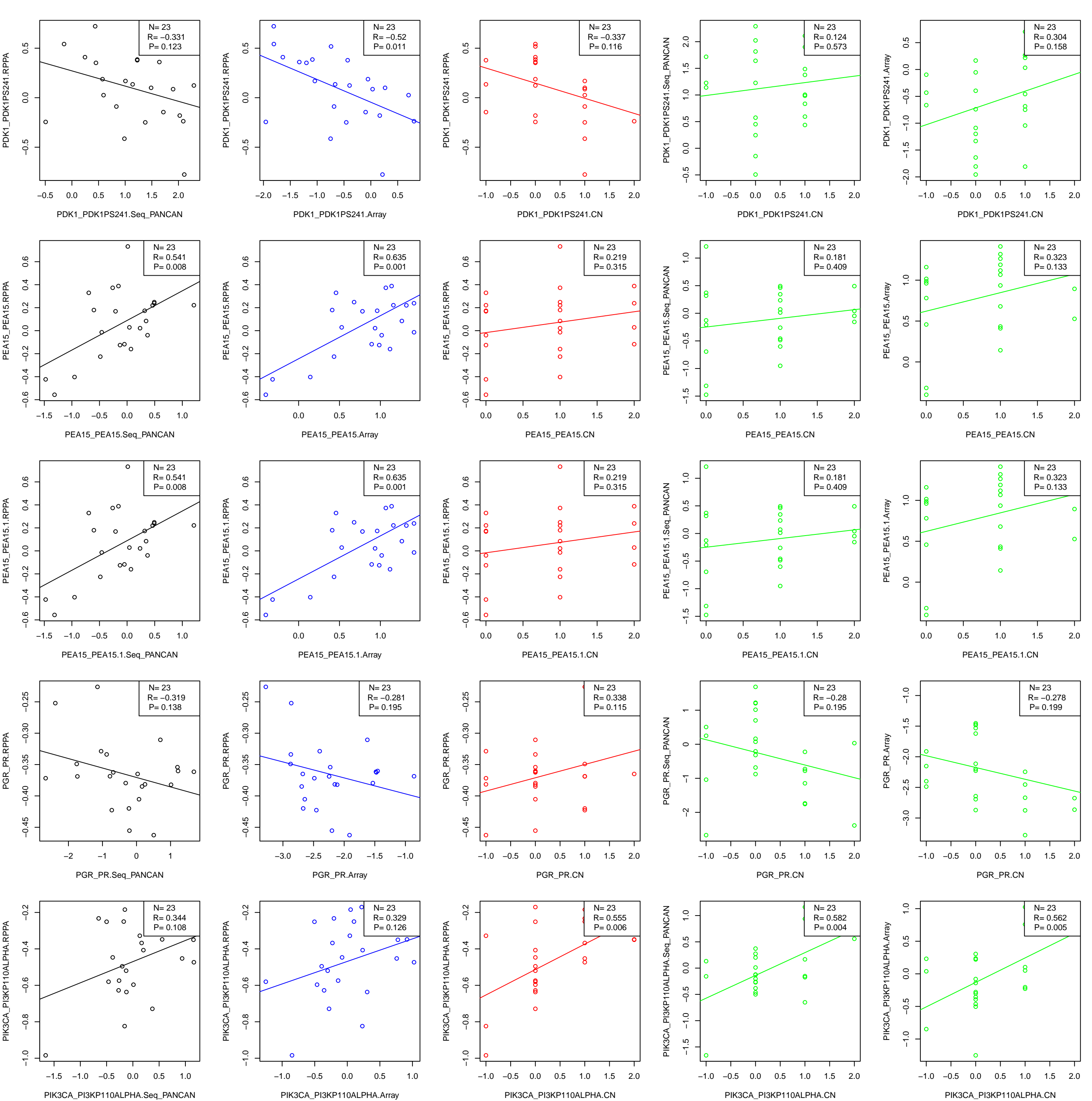

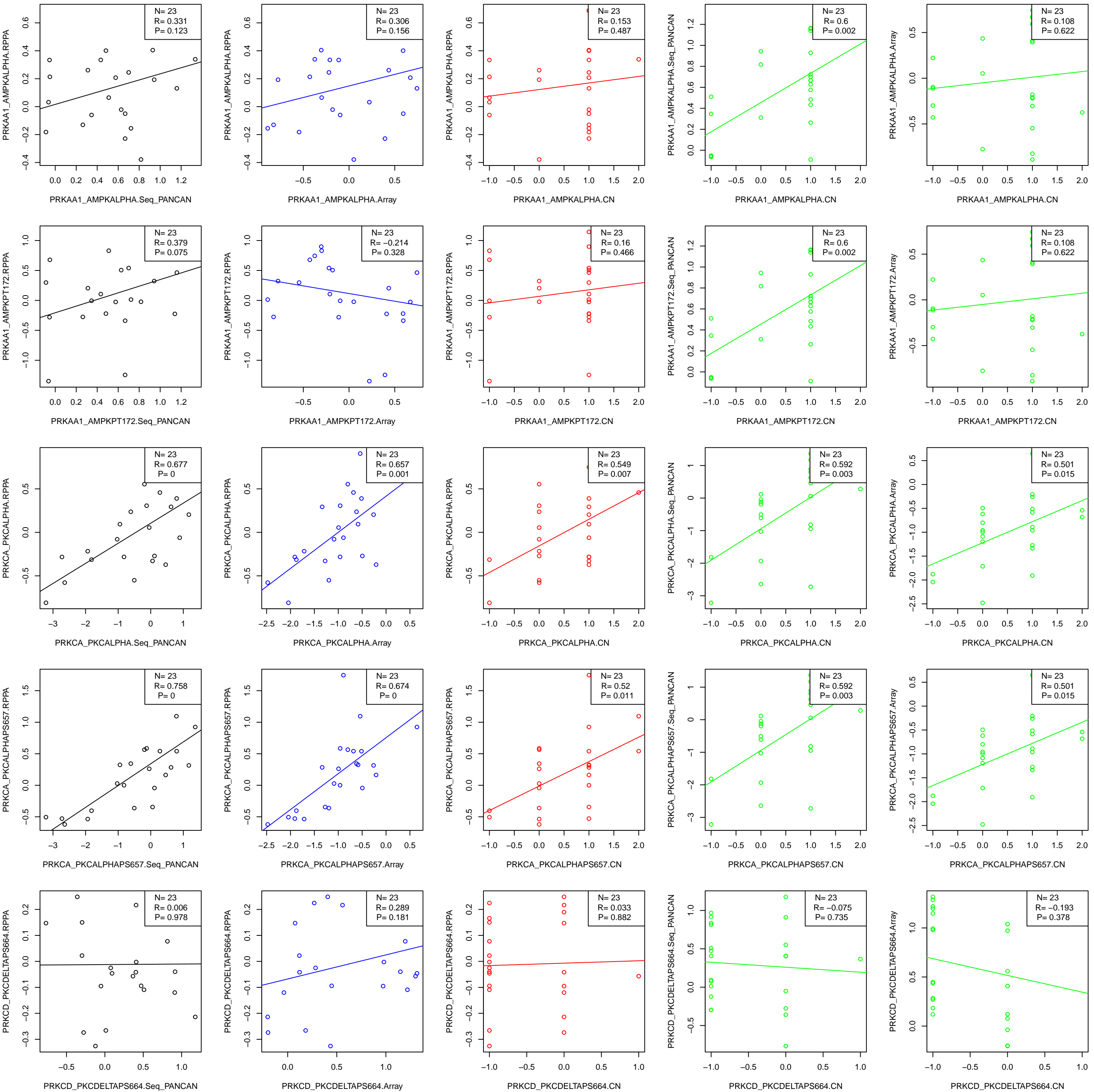

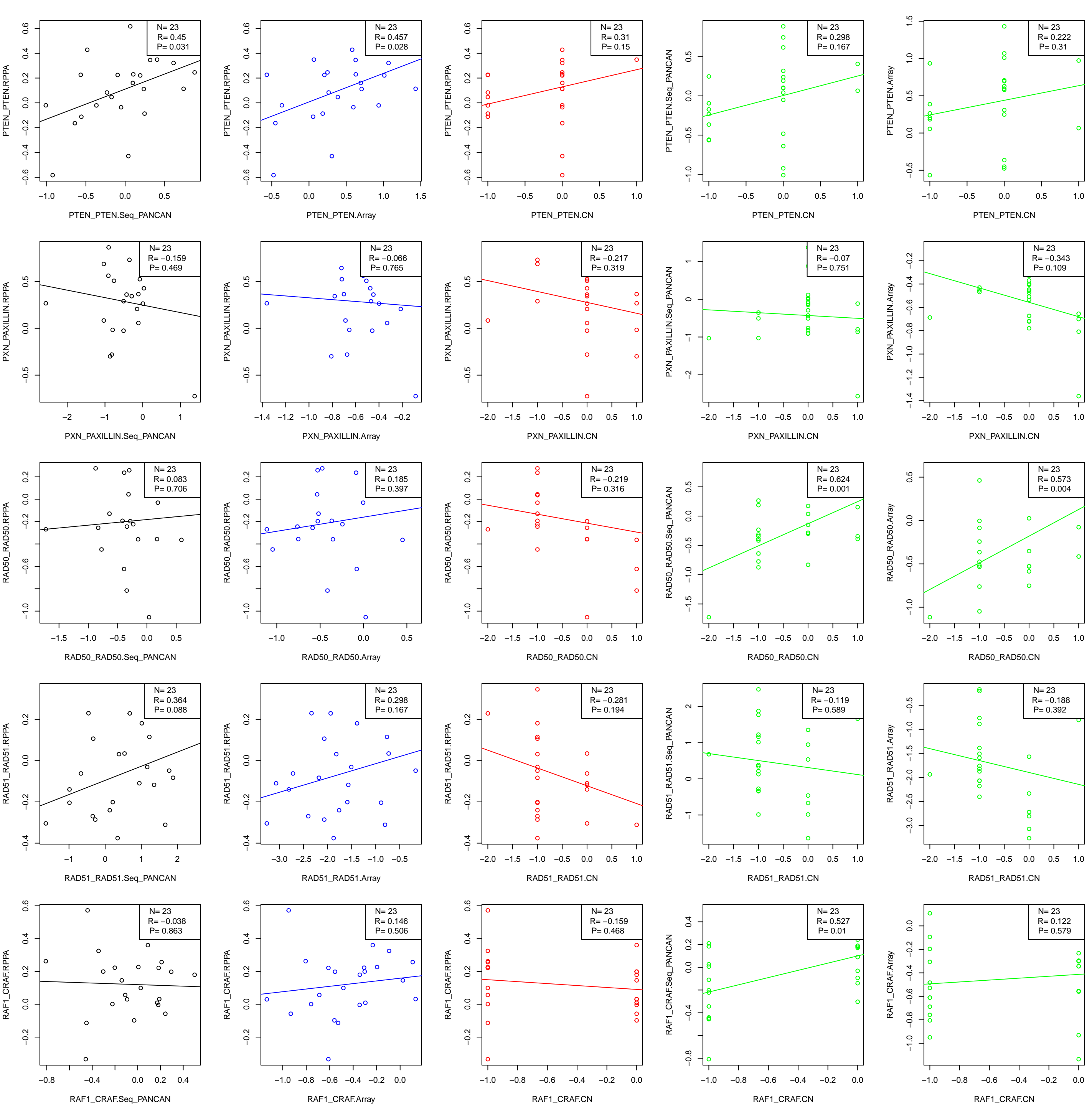

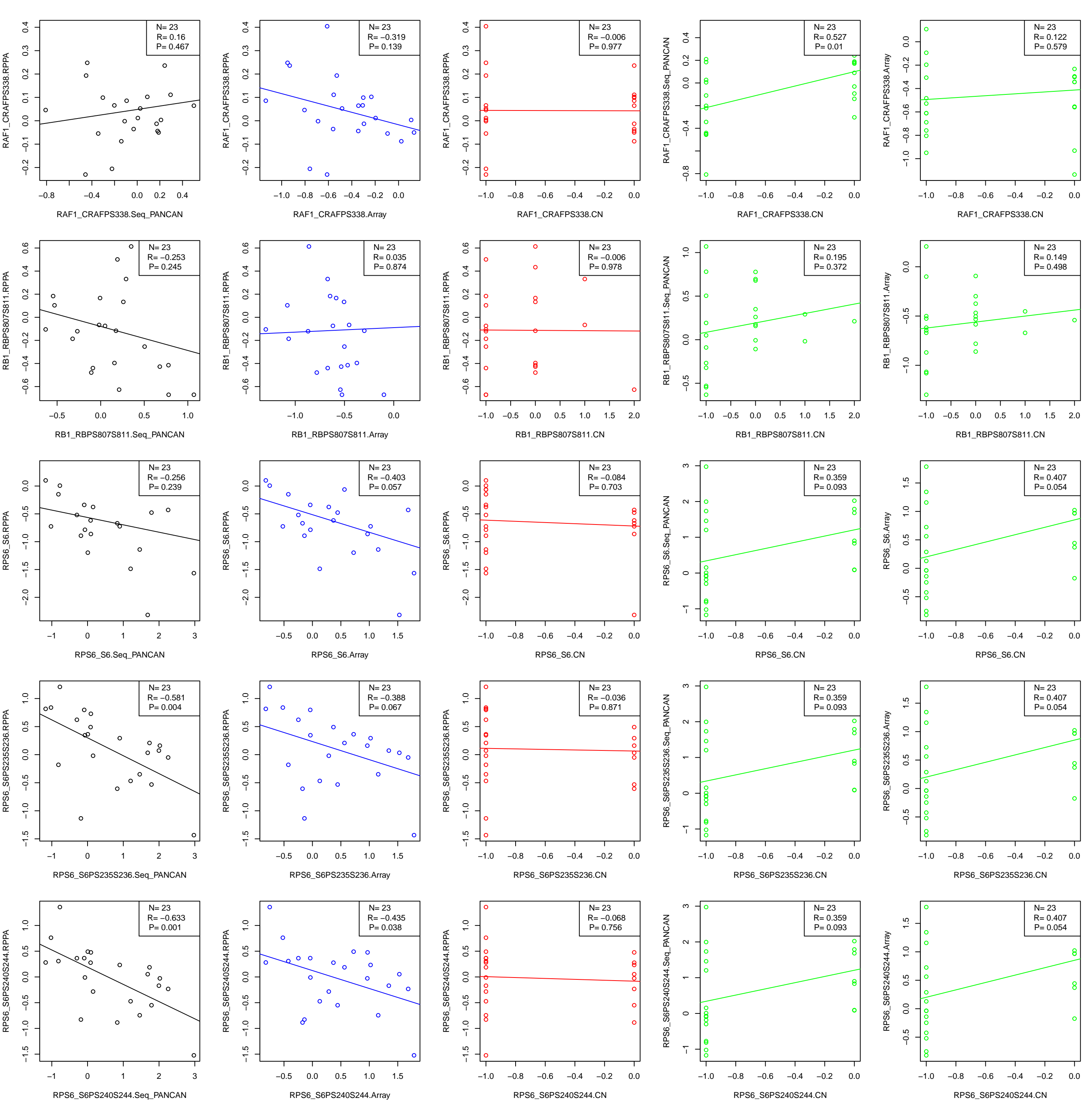

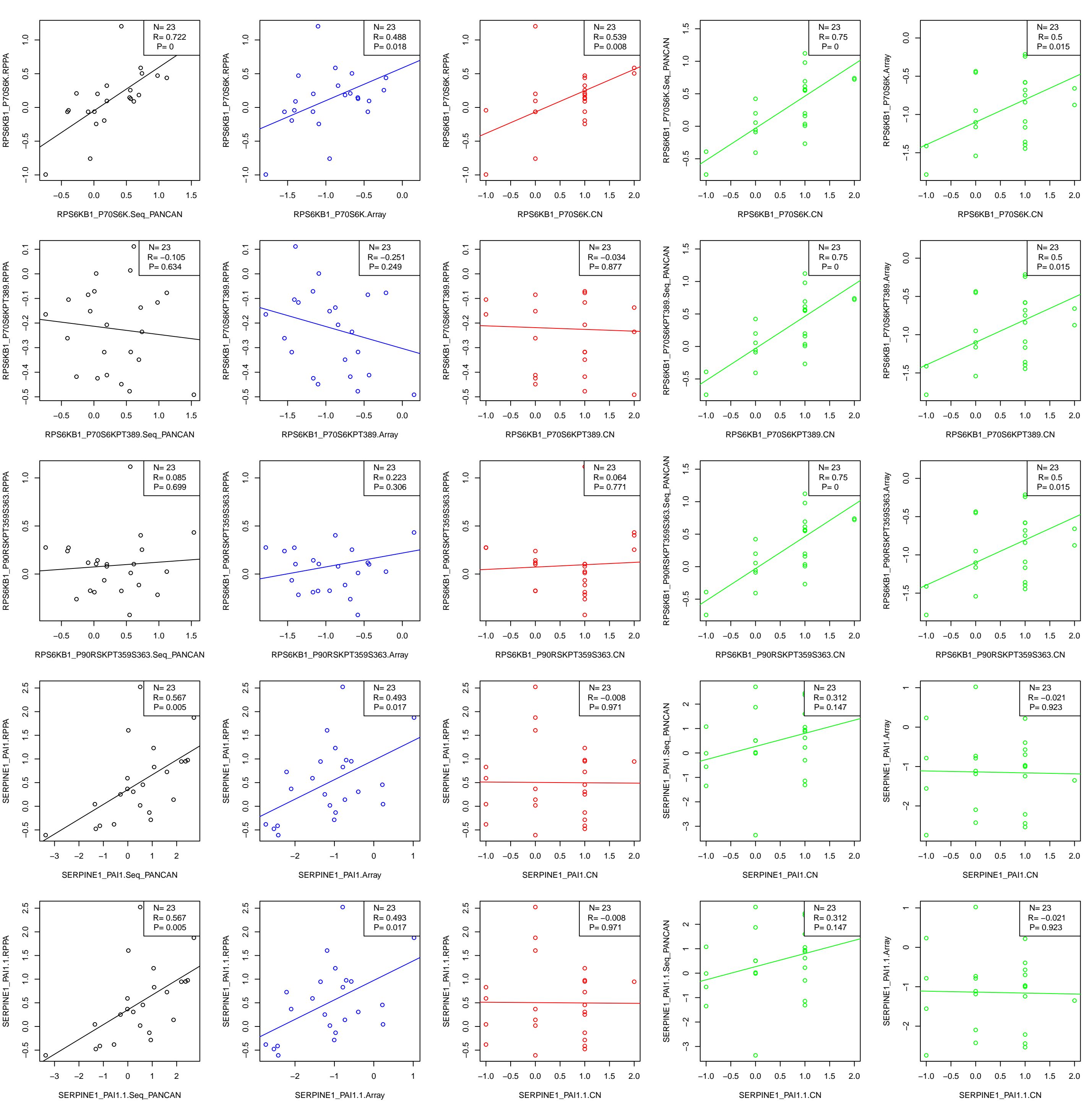

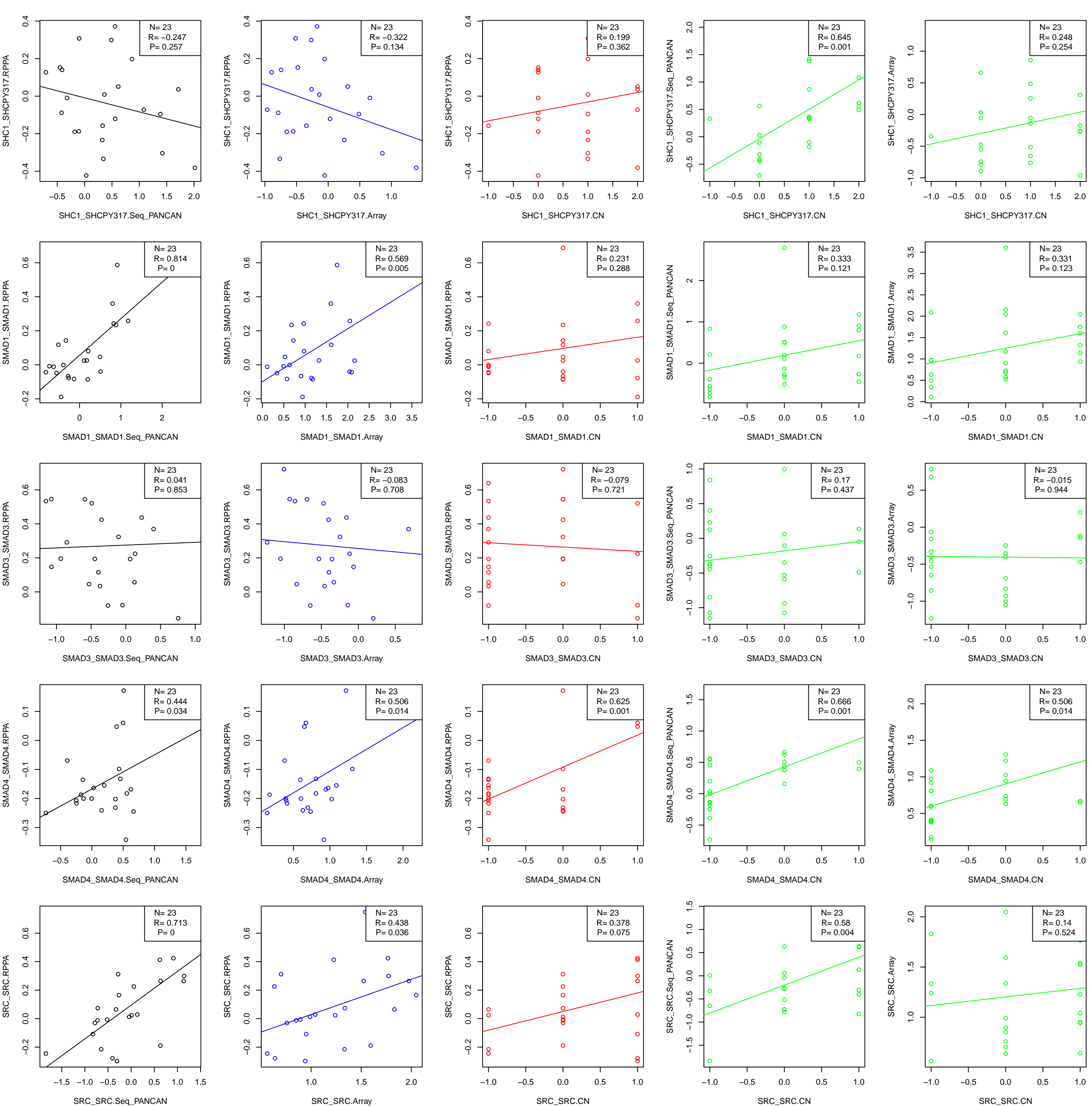

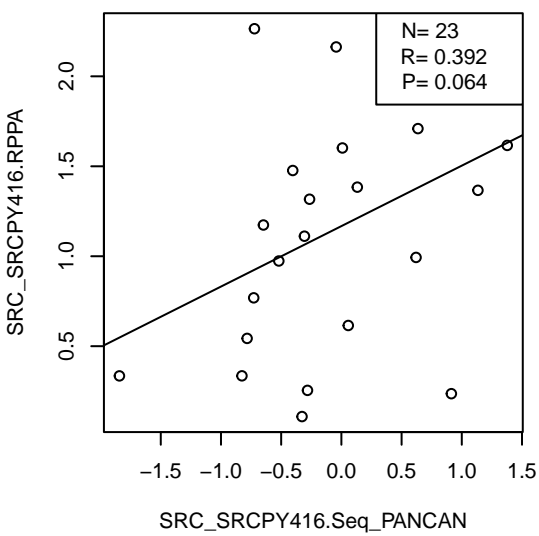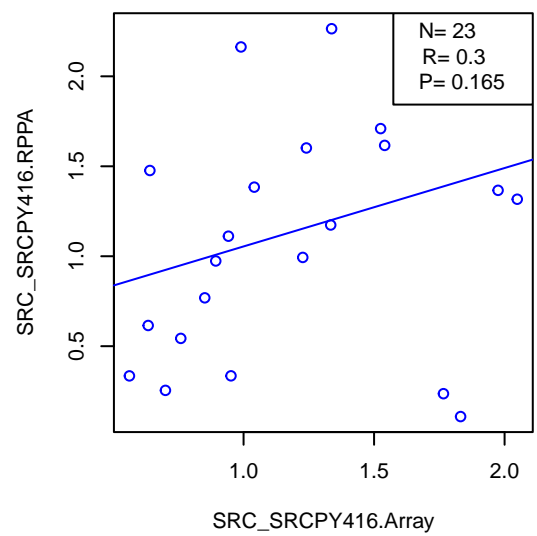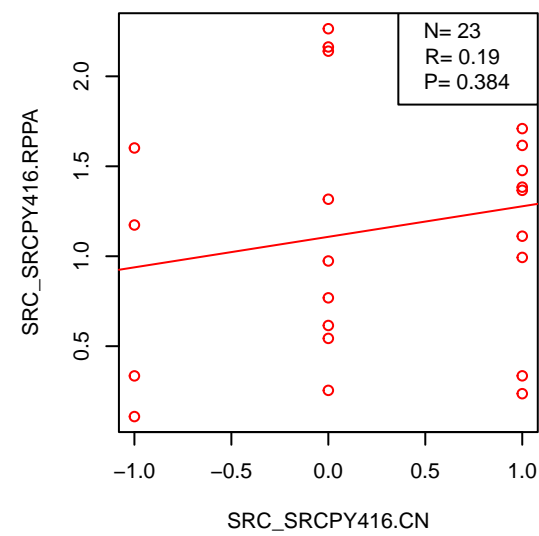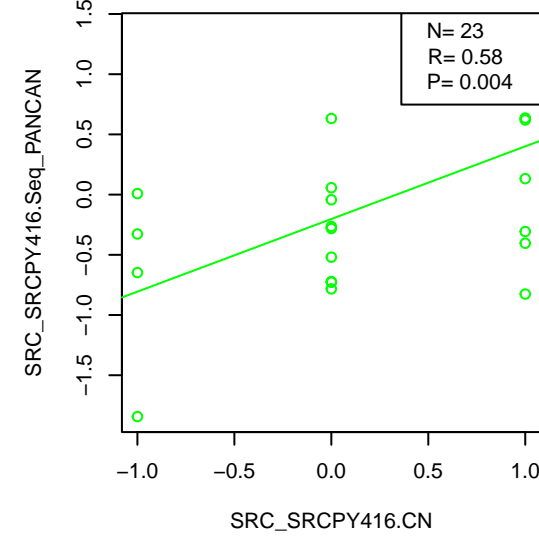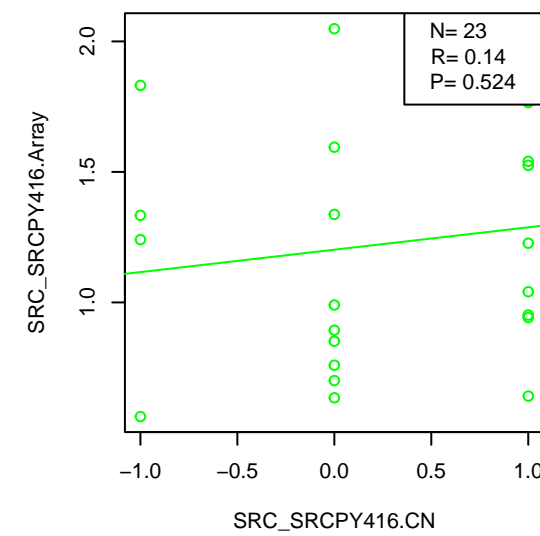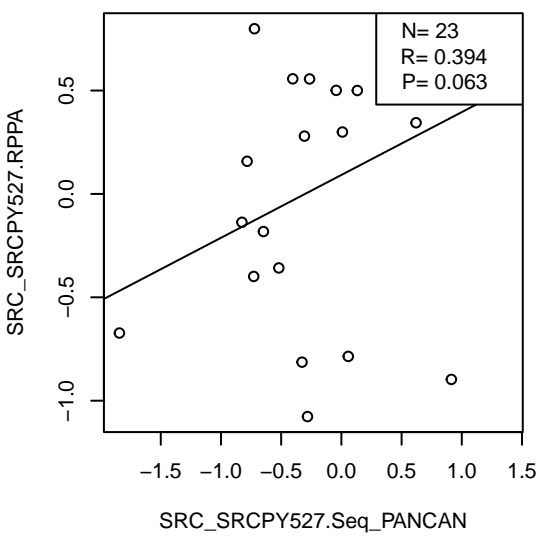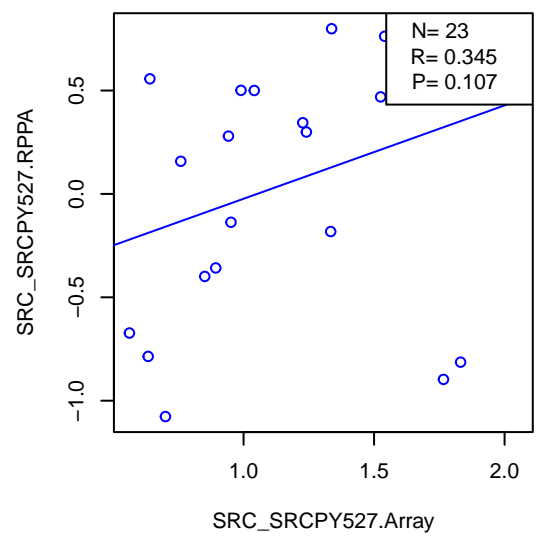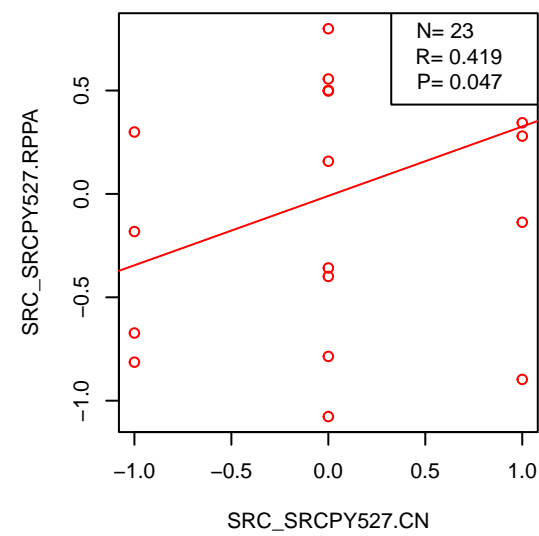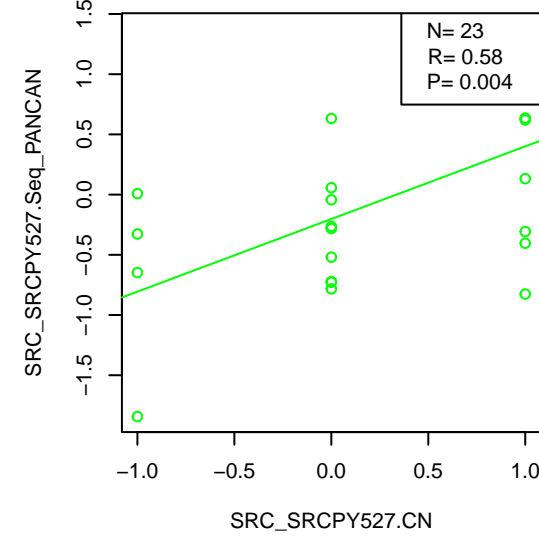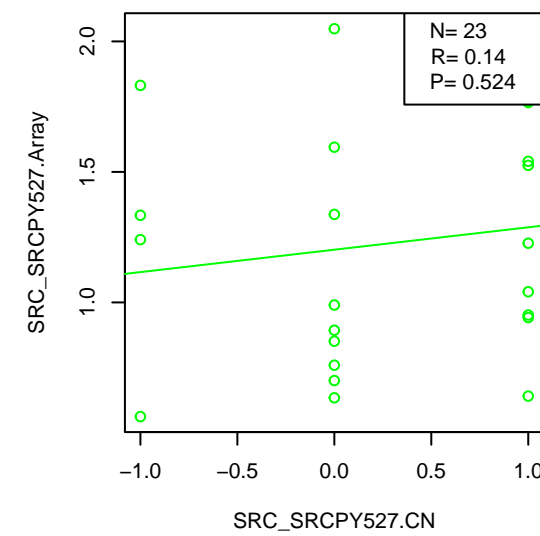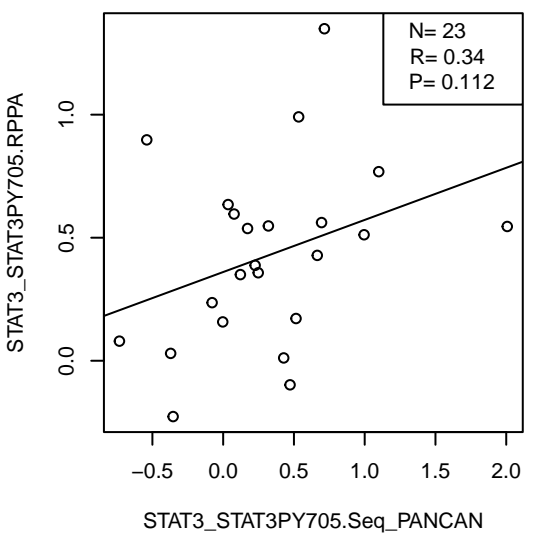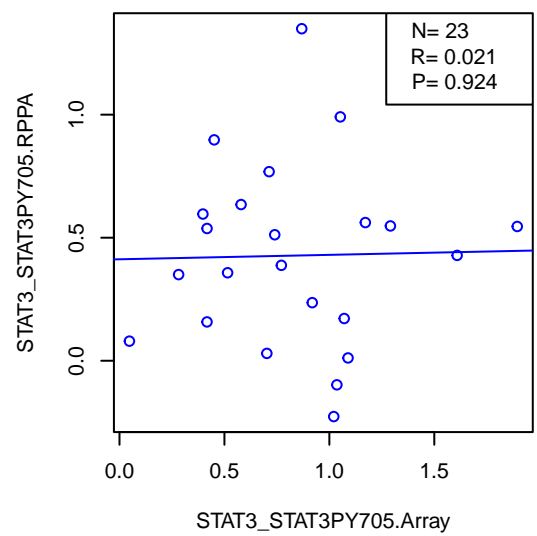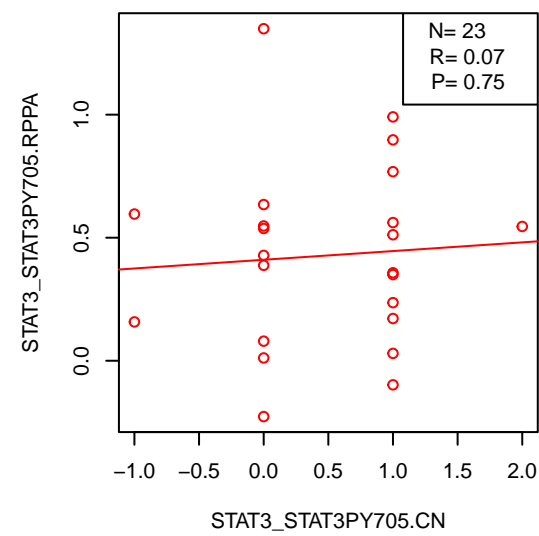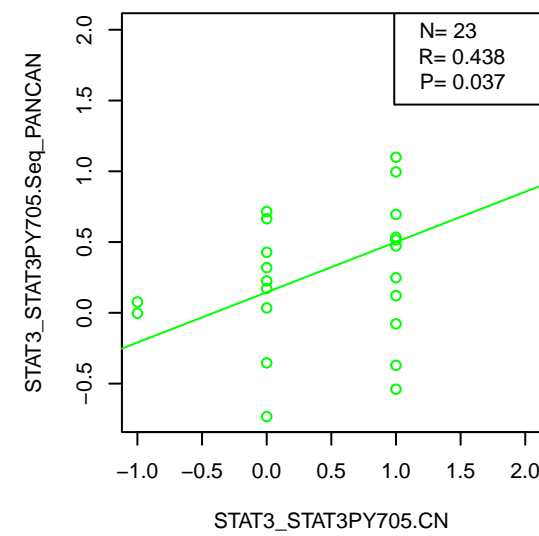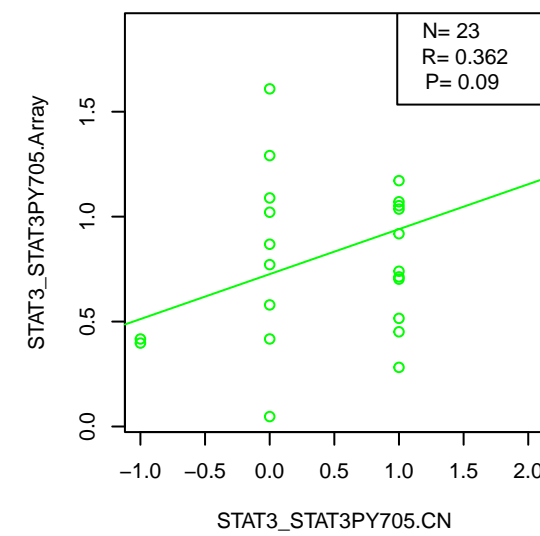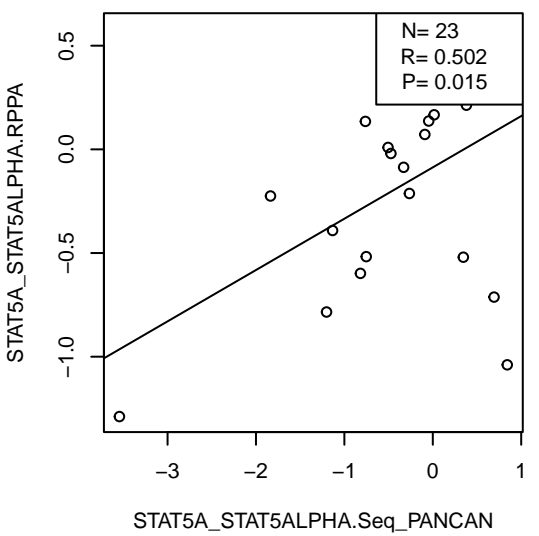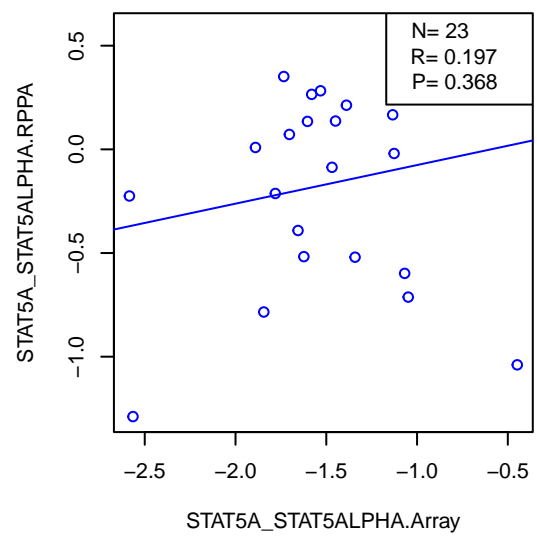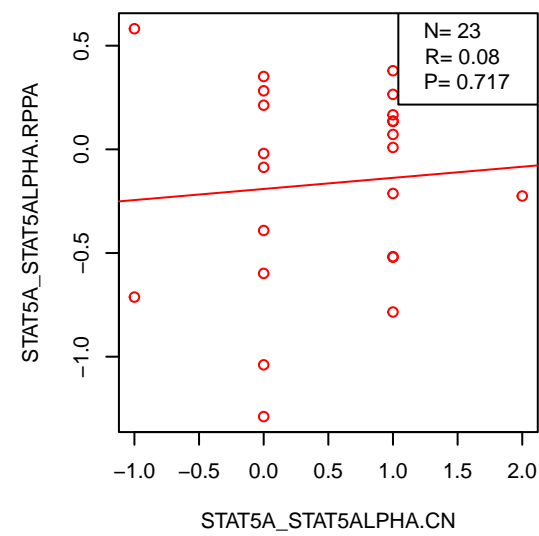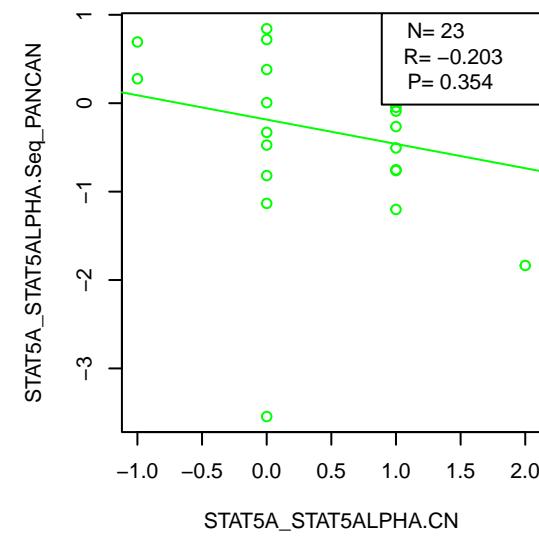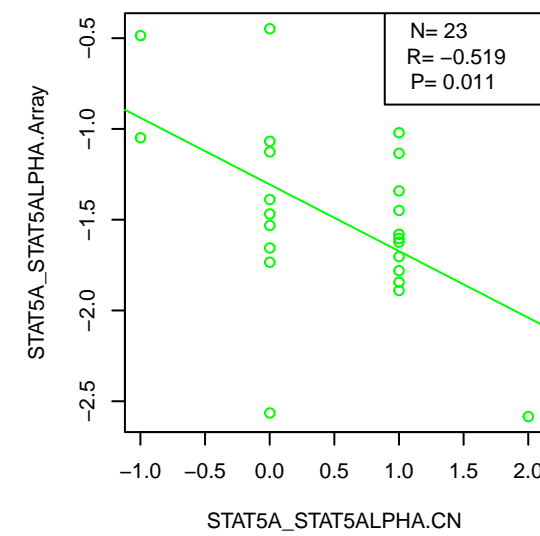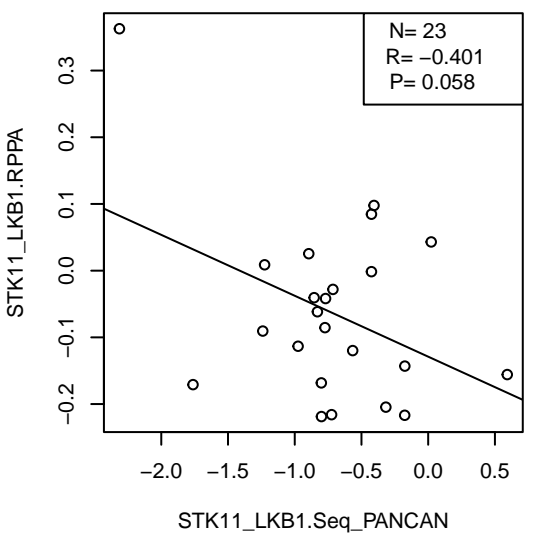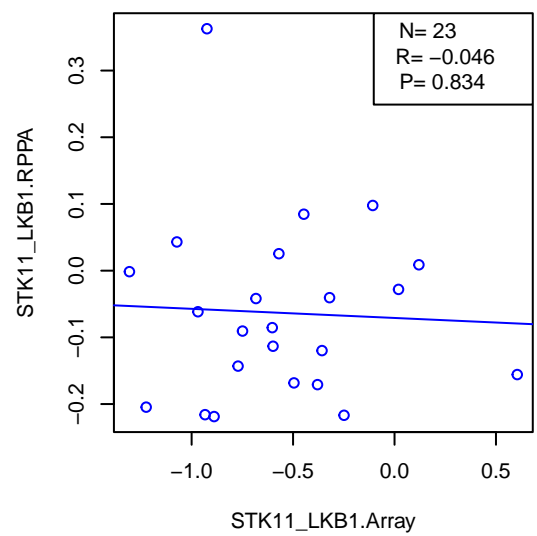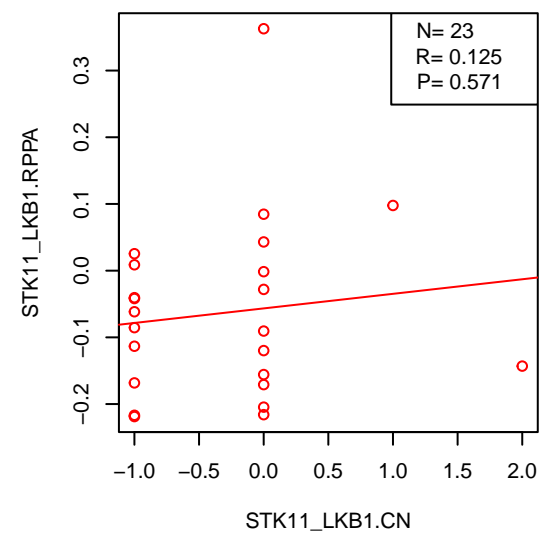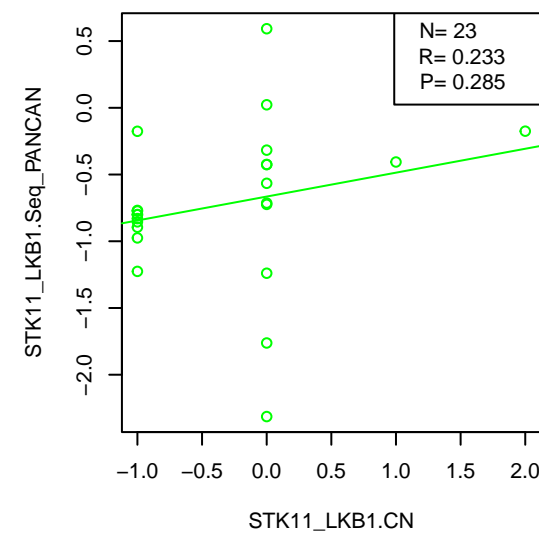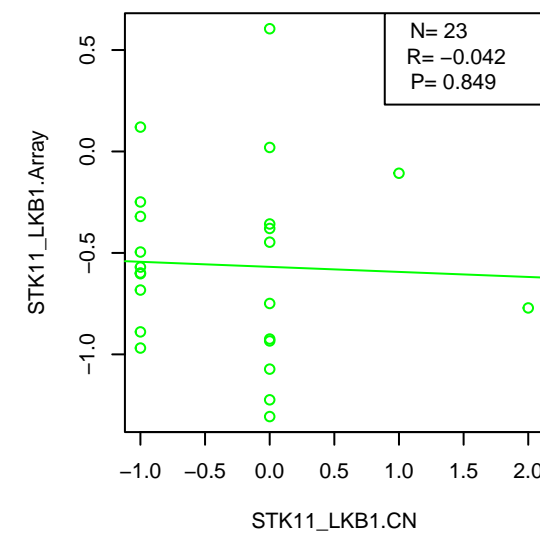

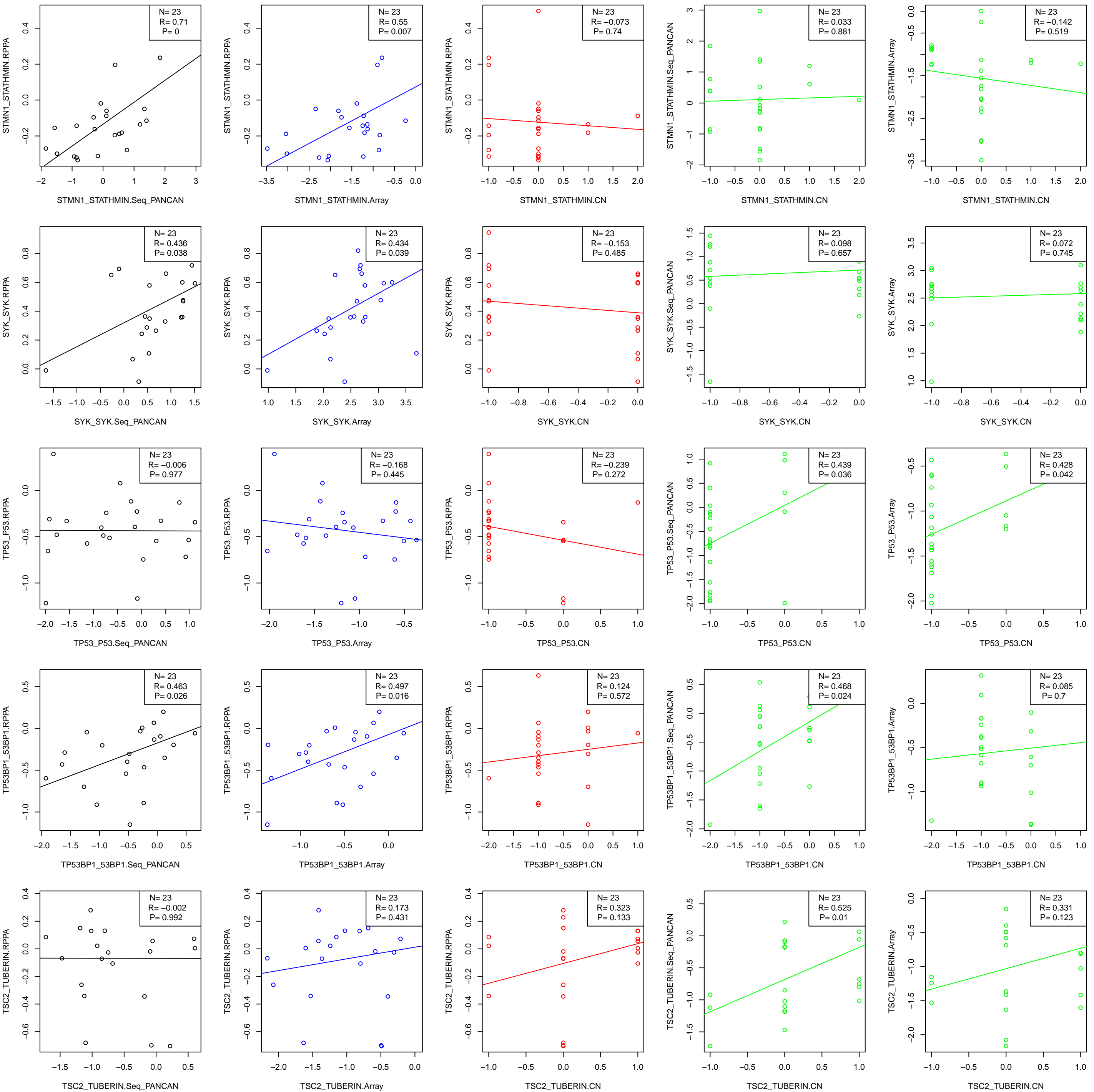

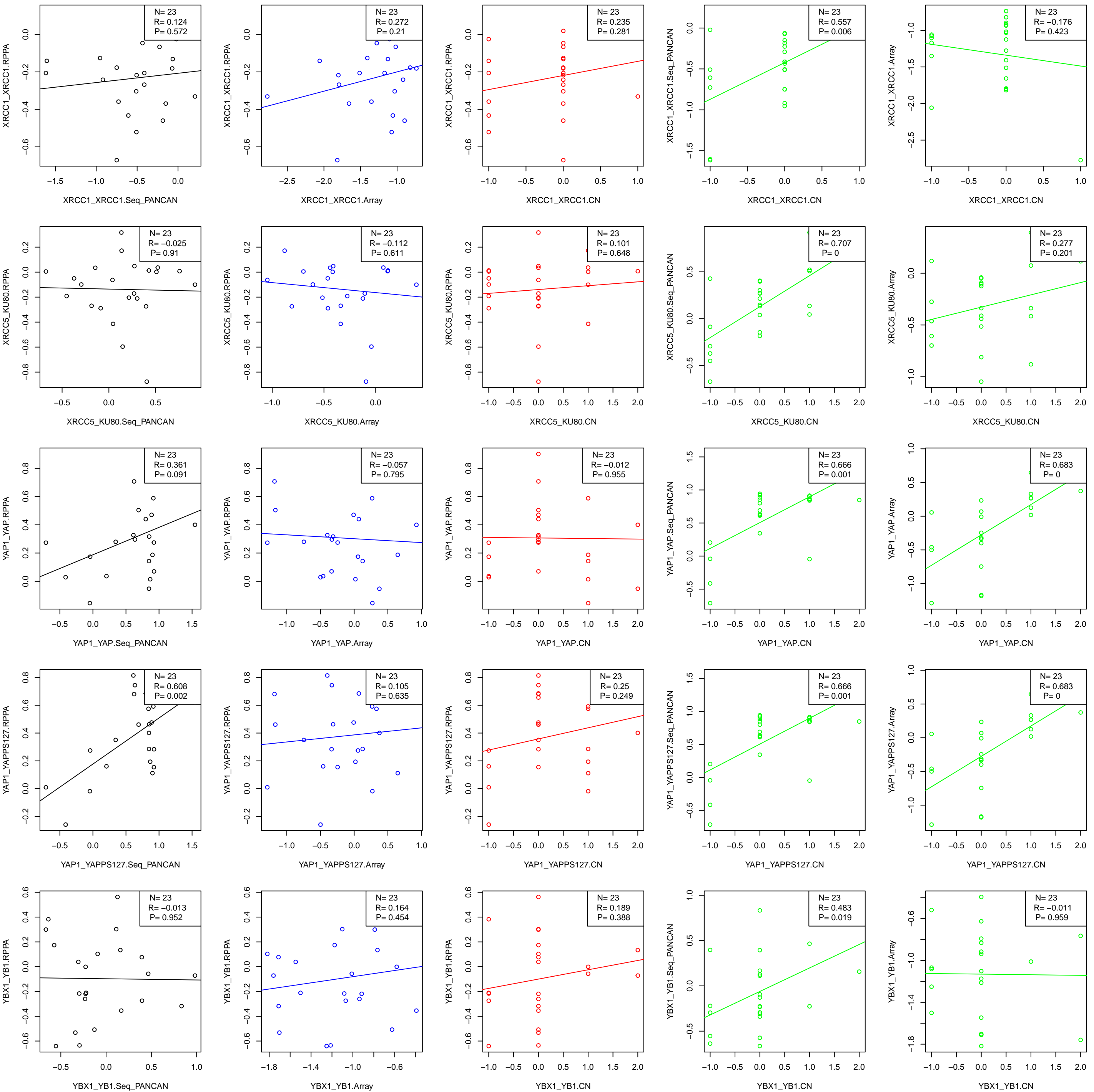

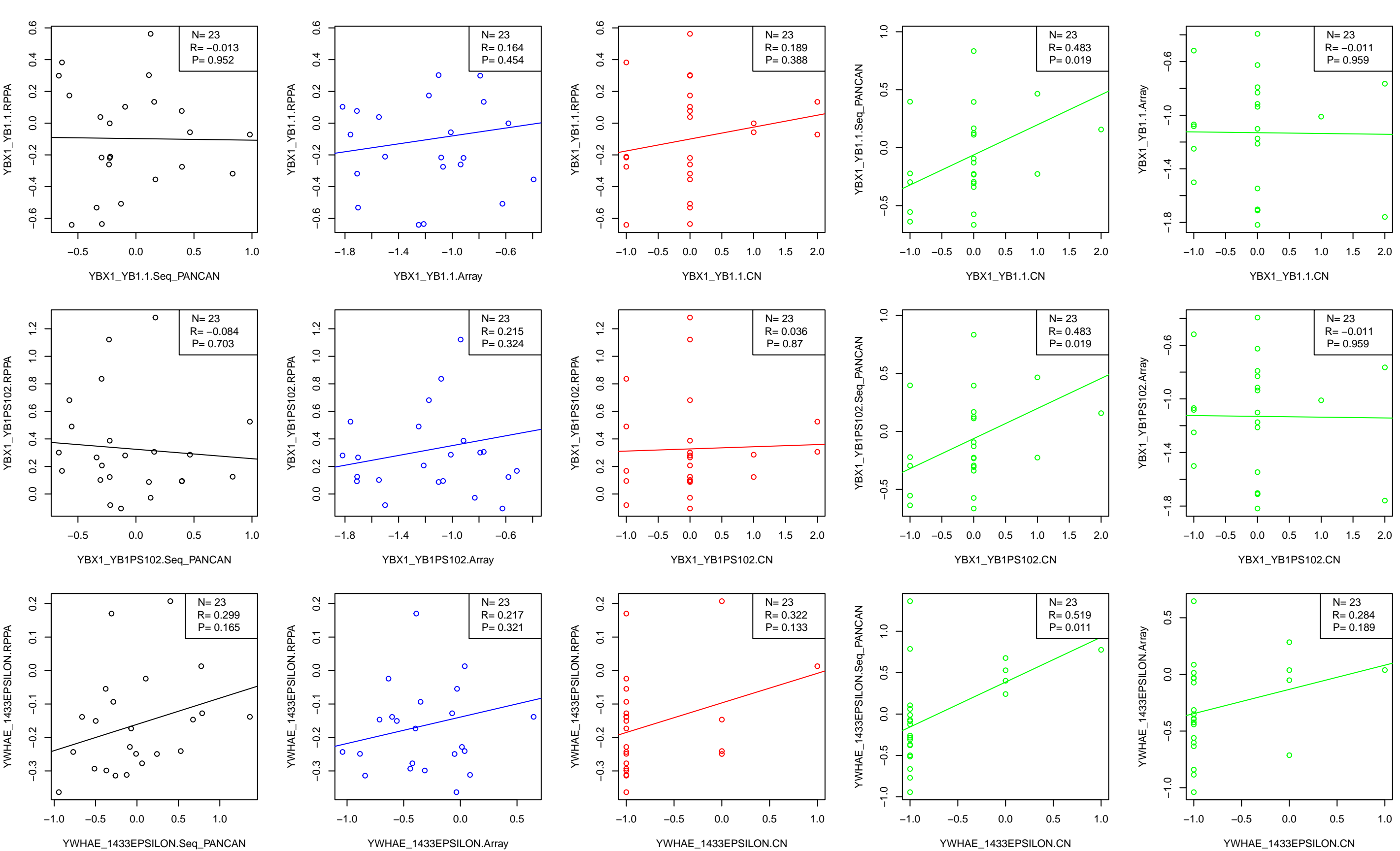

Supplement: Supplementary file 4 [file DataSheet4.PDF]

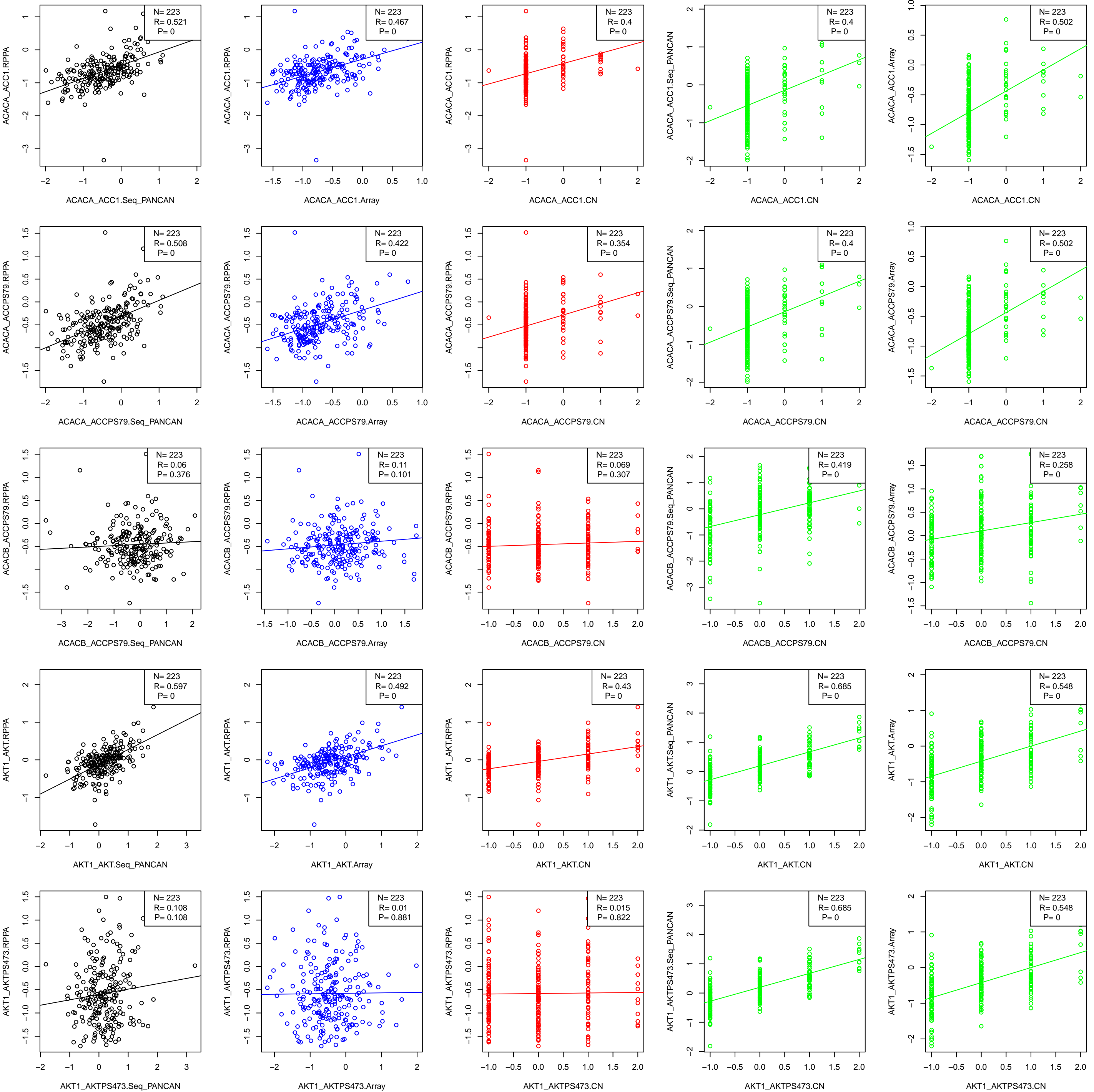

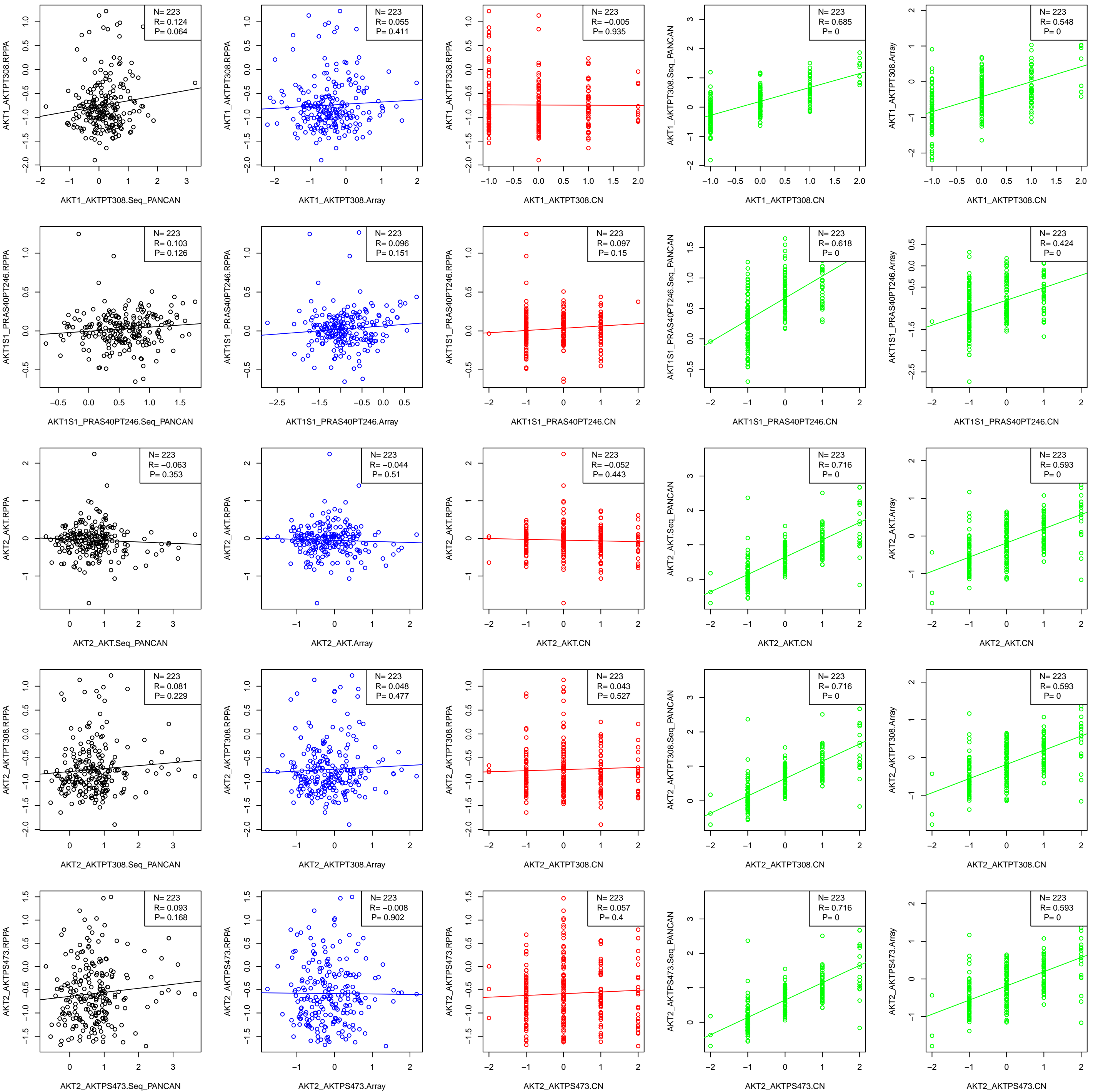

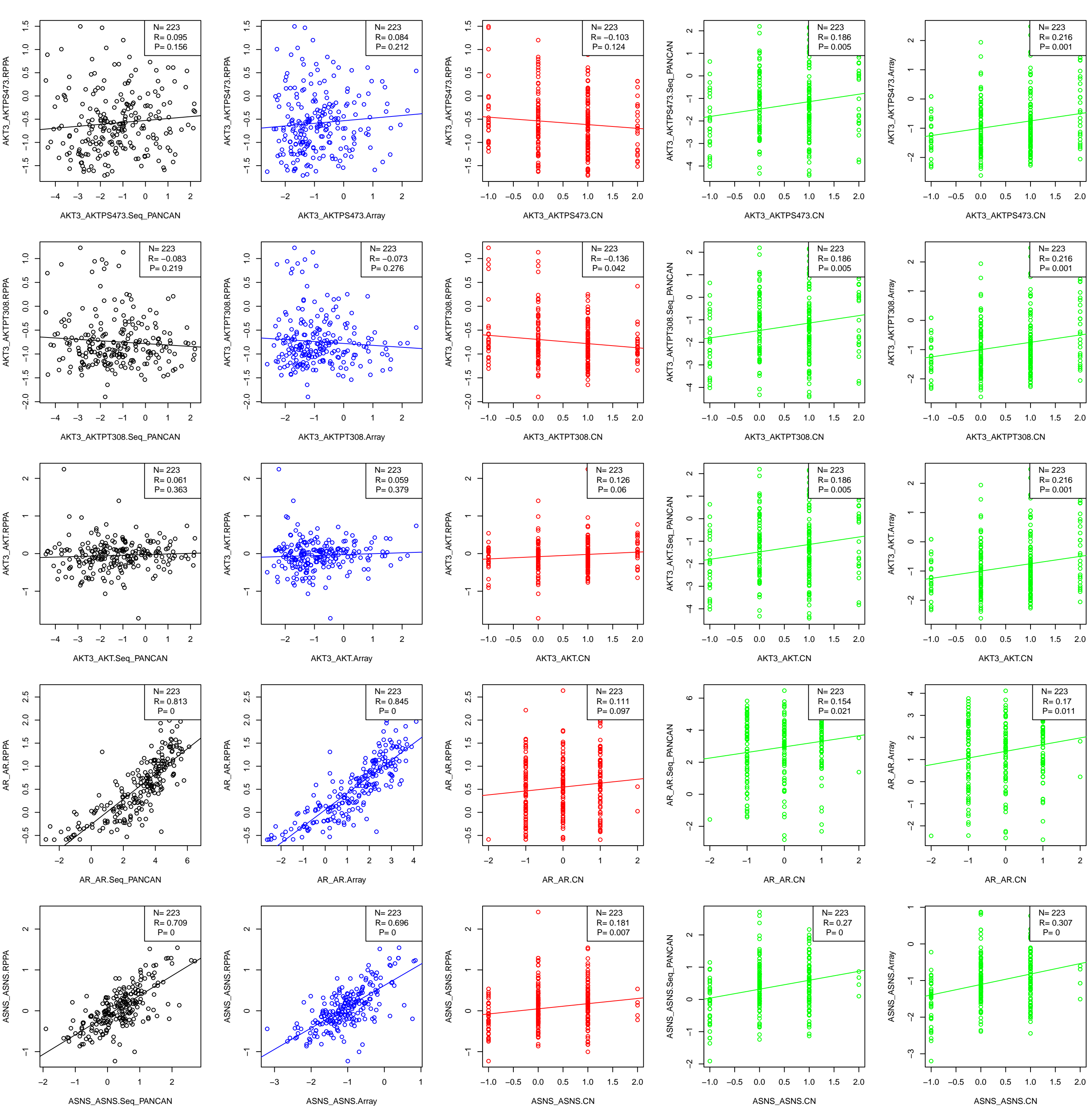

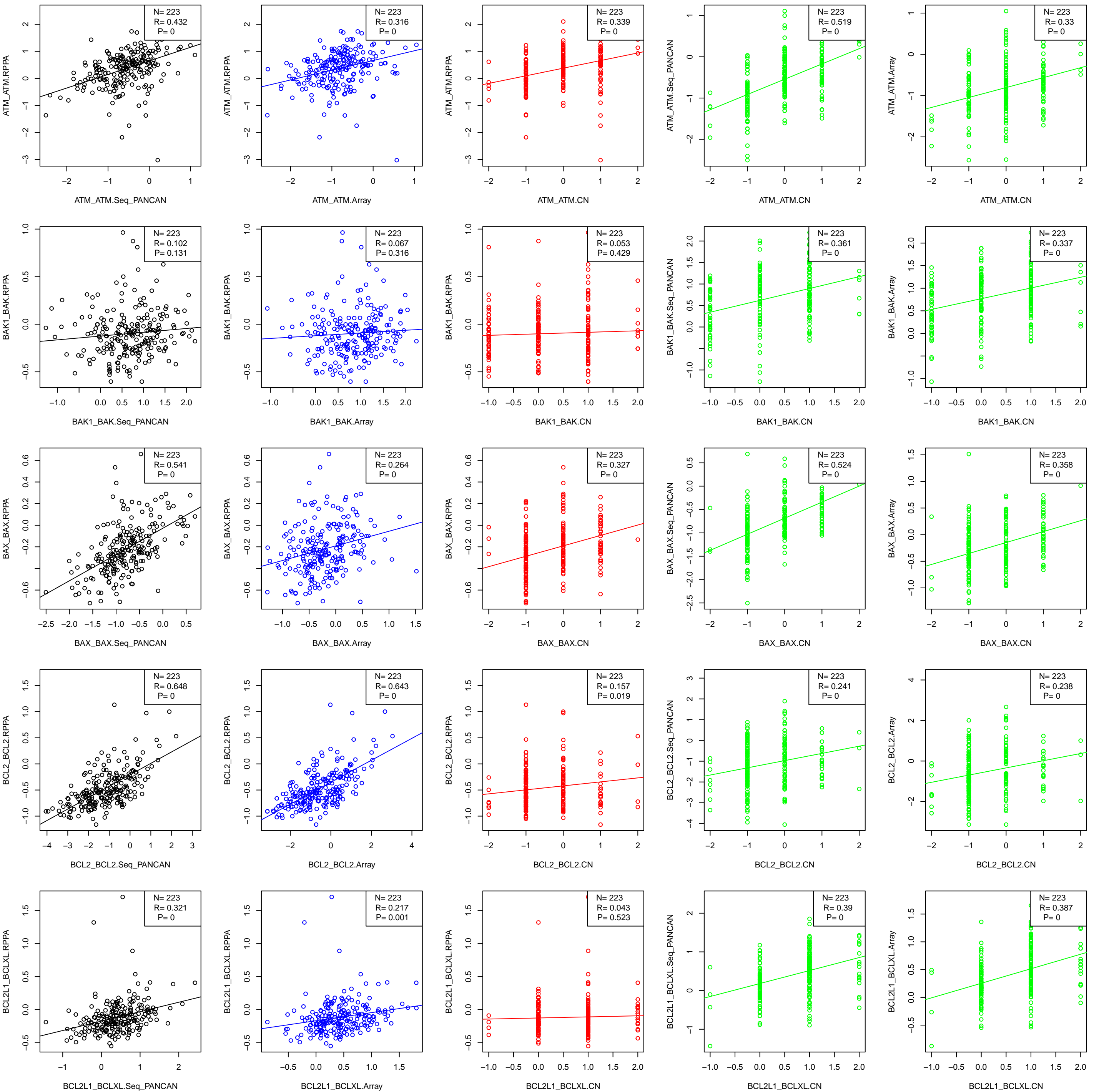

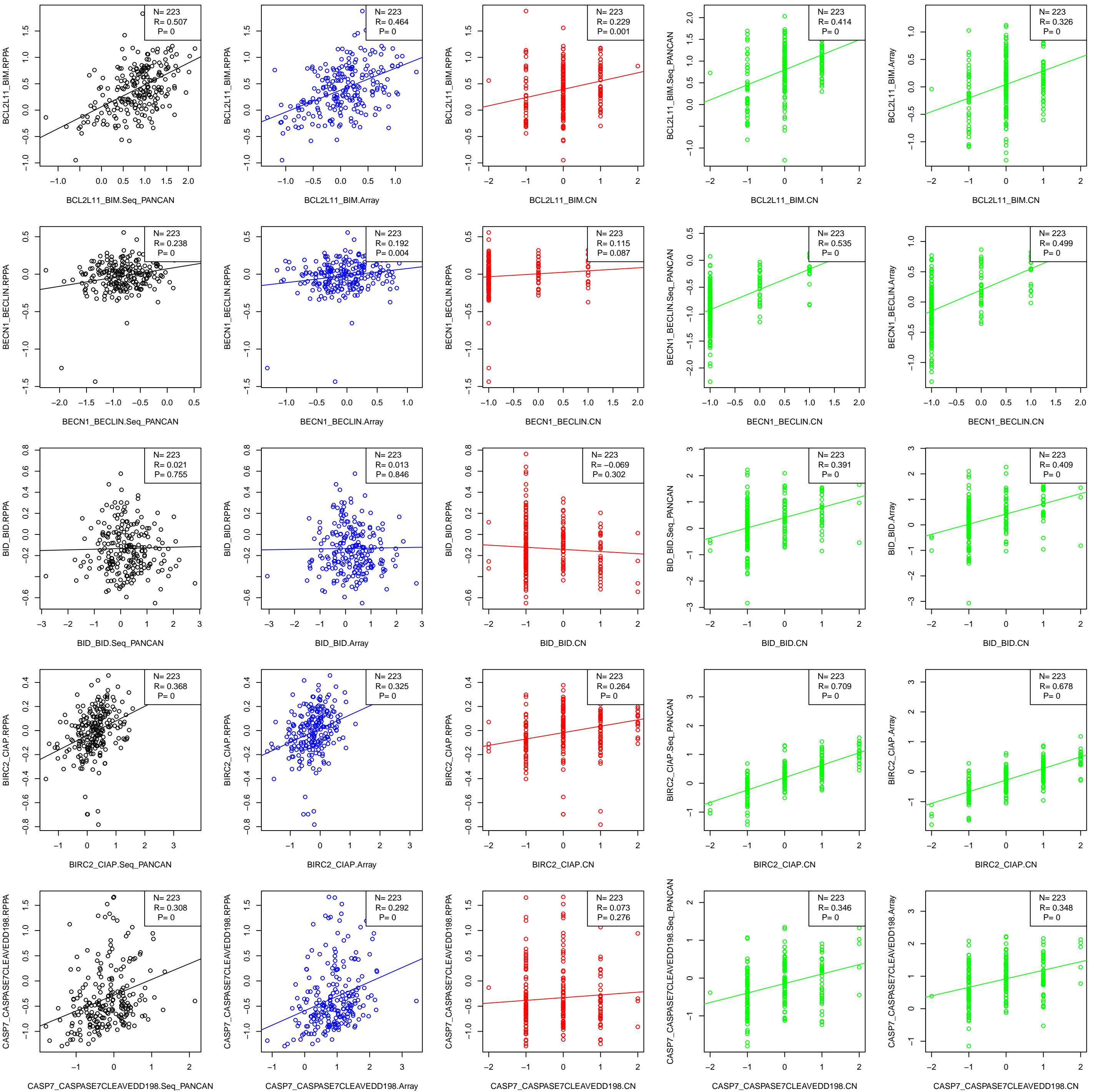

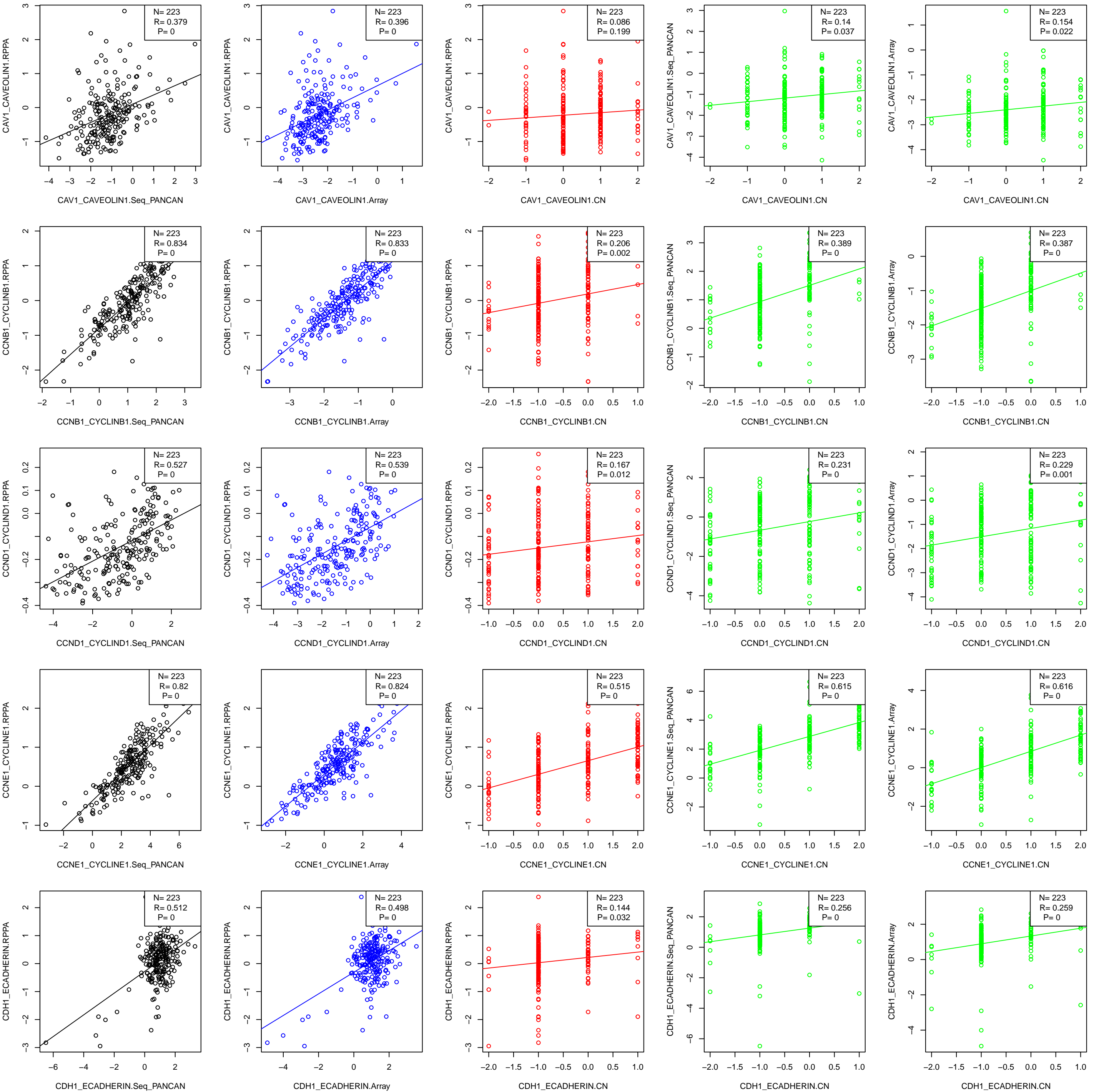

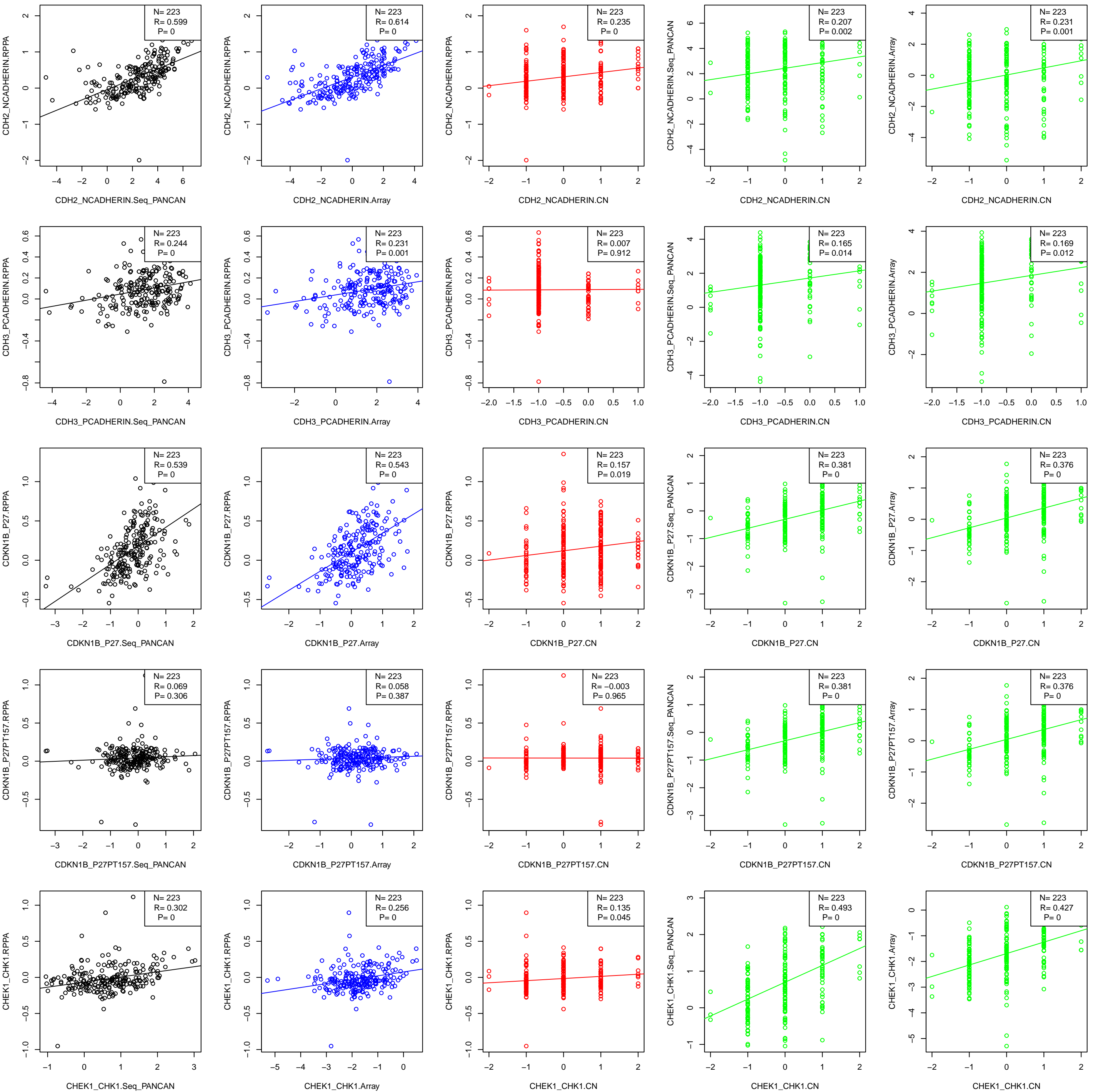

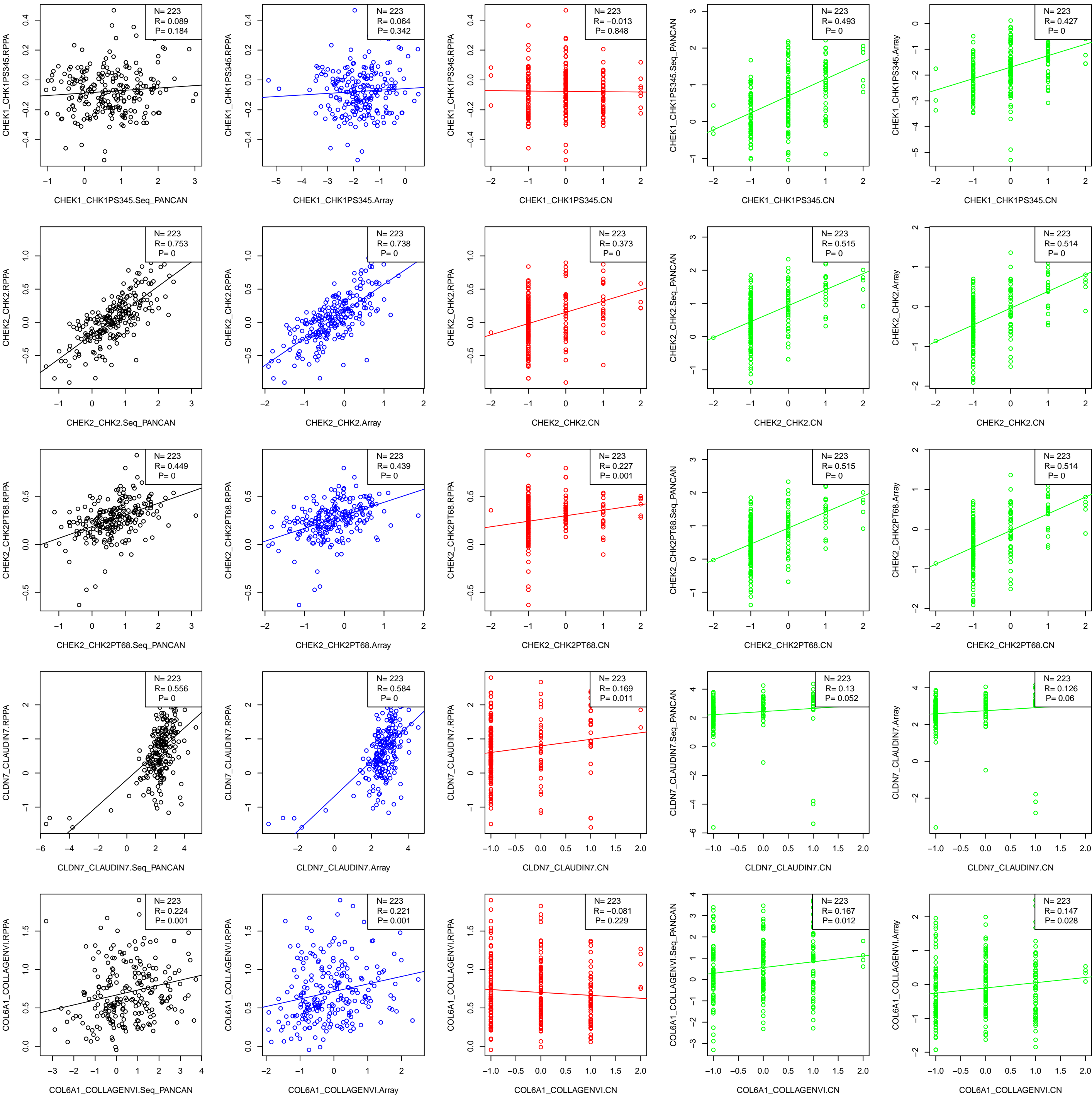

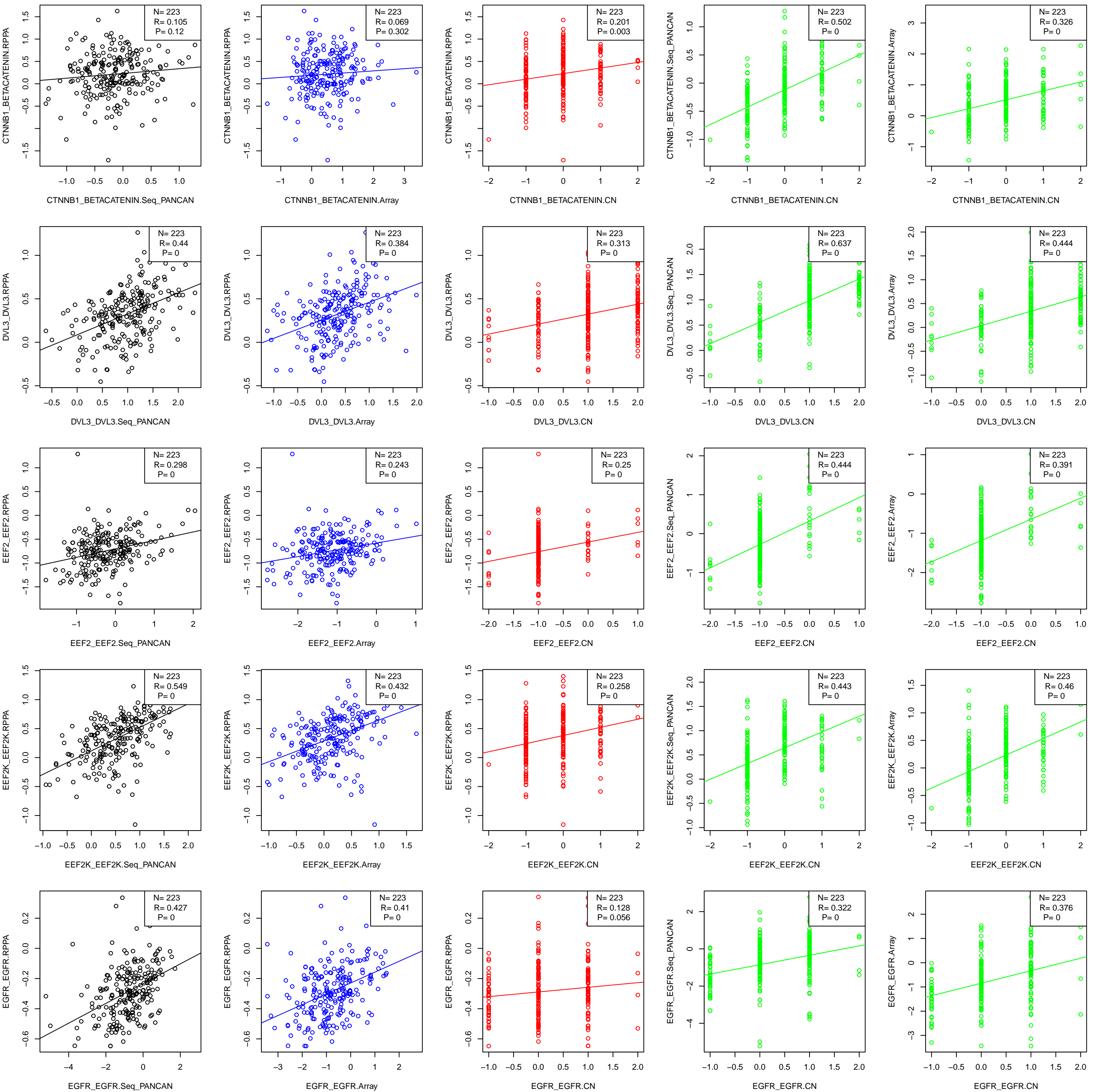

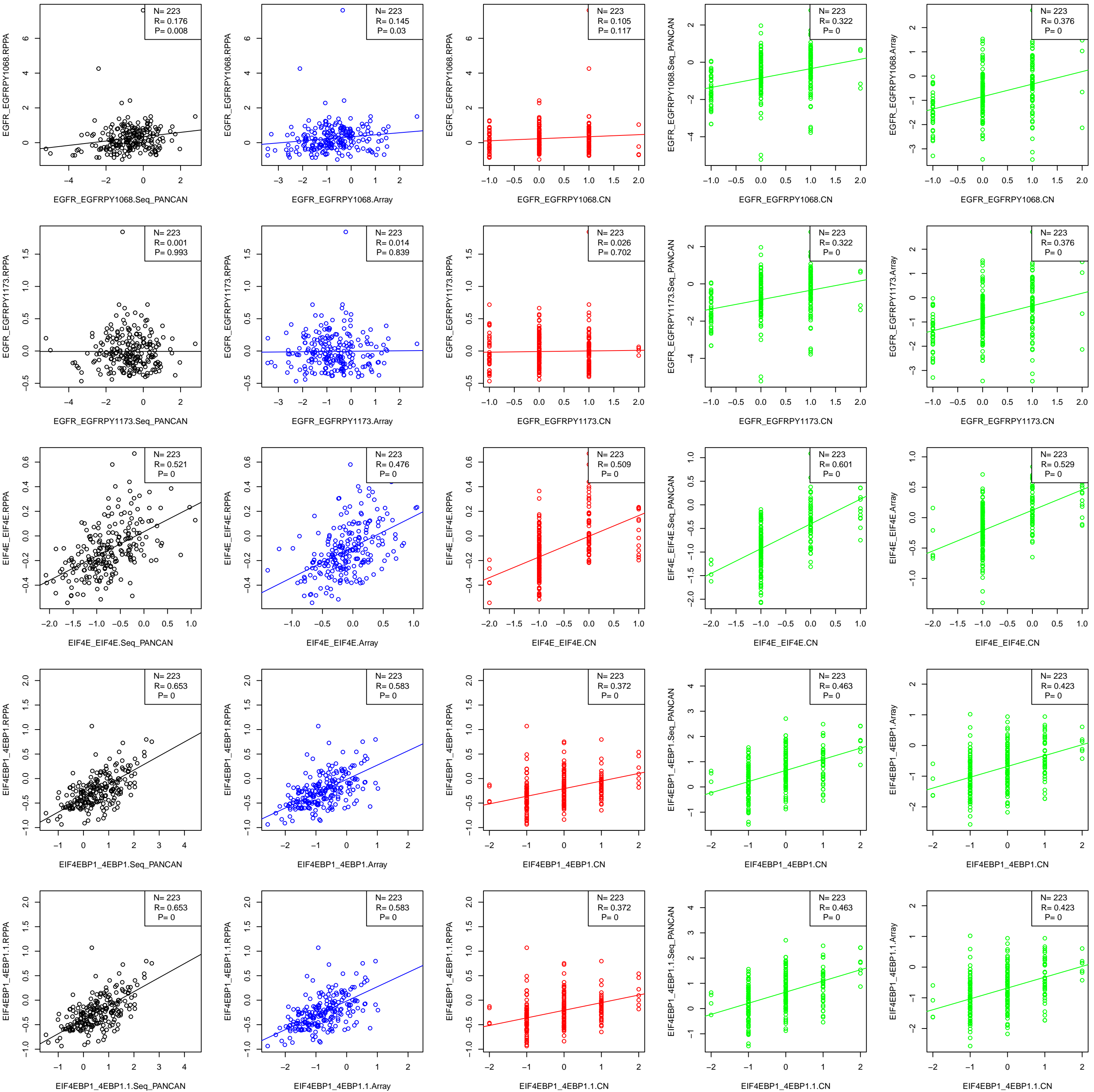

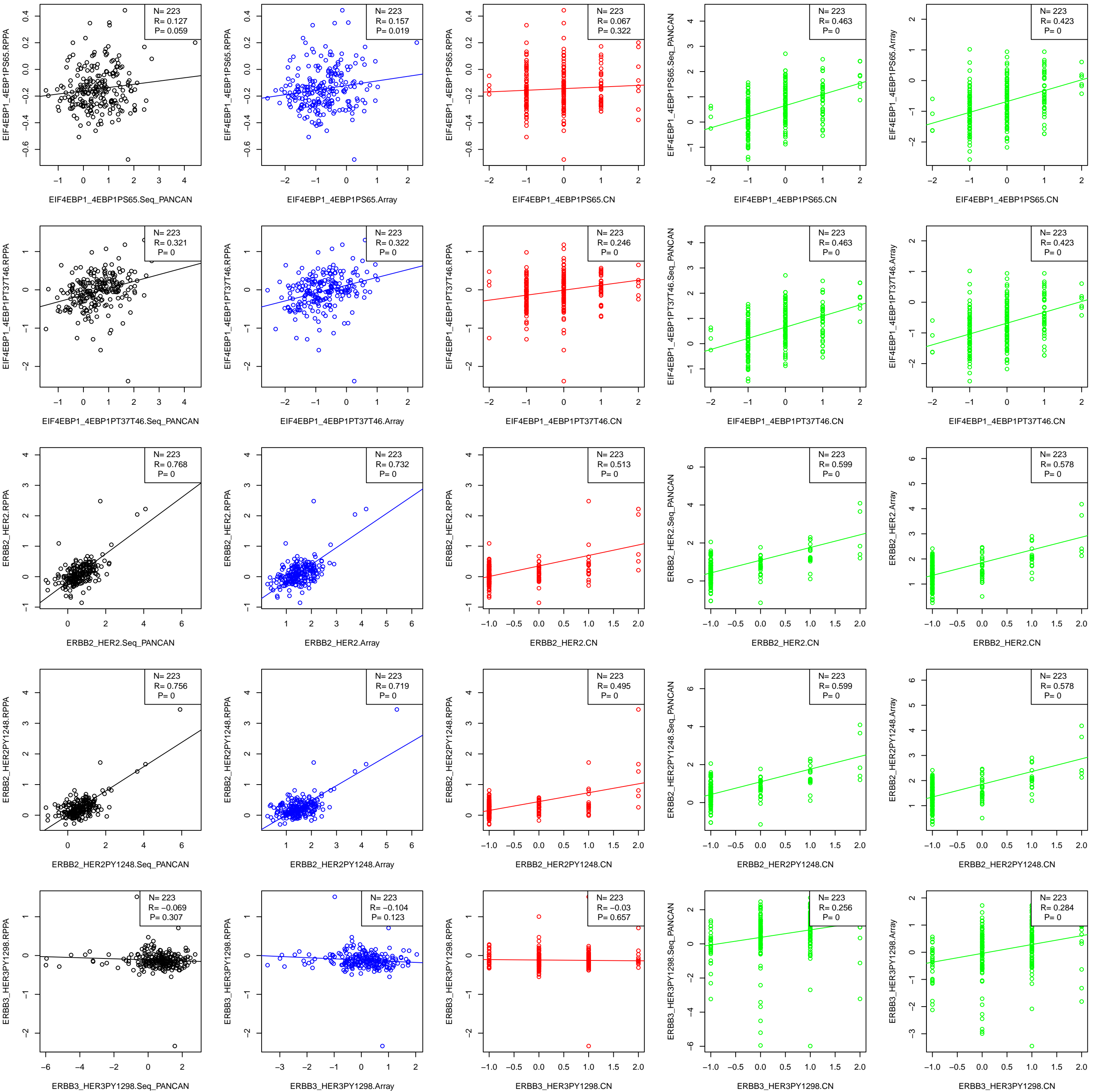

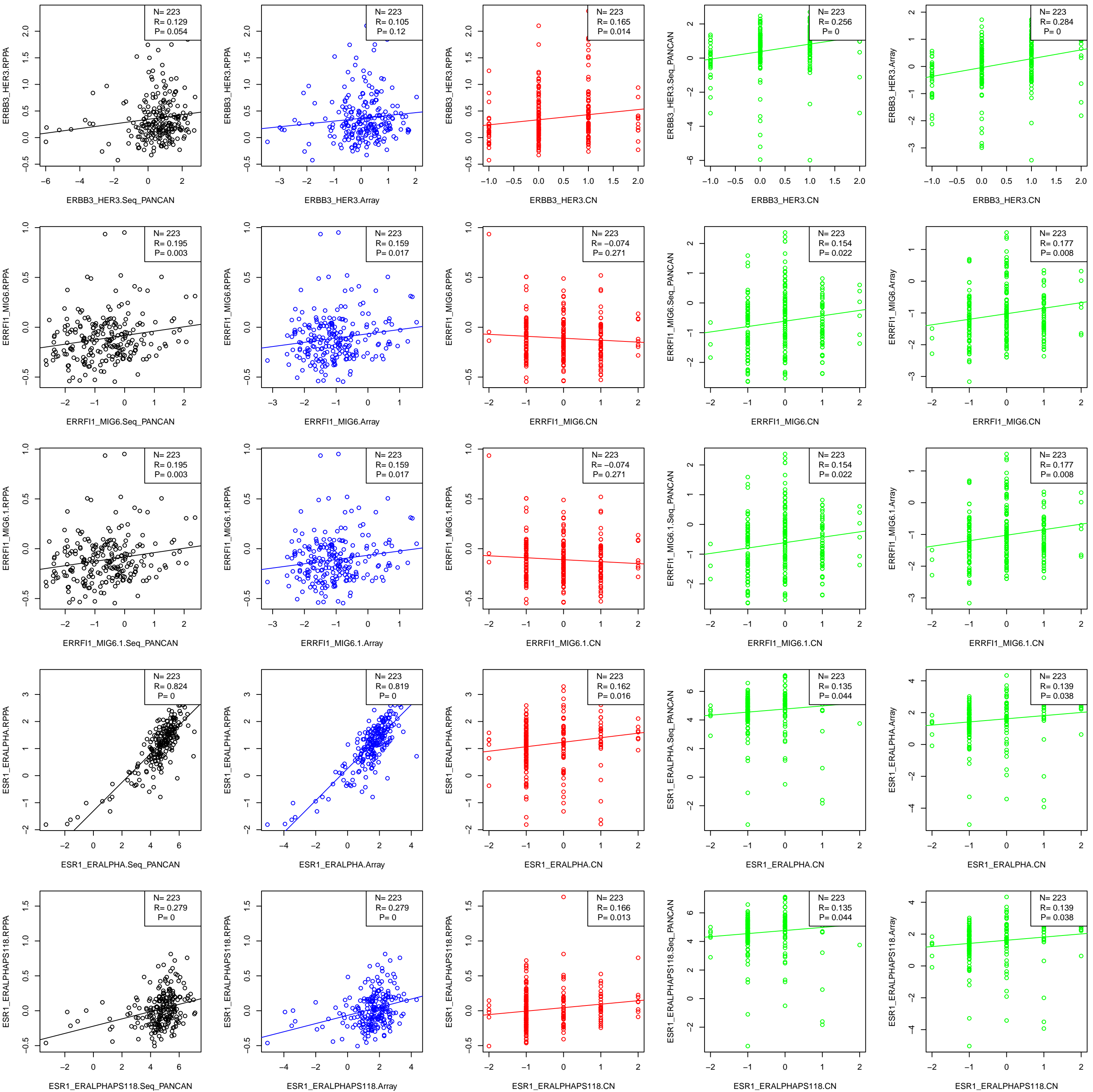

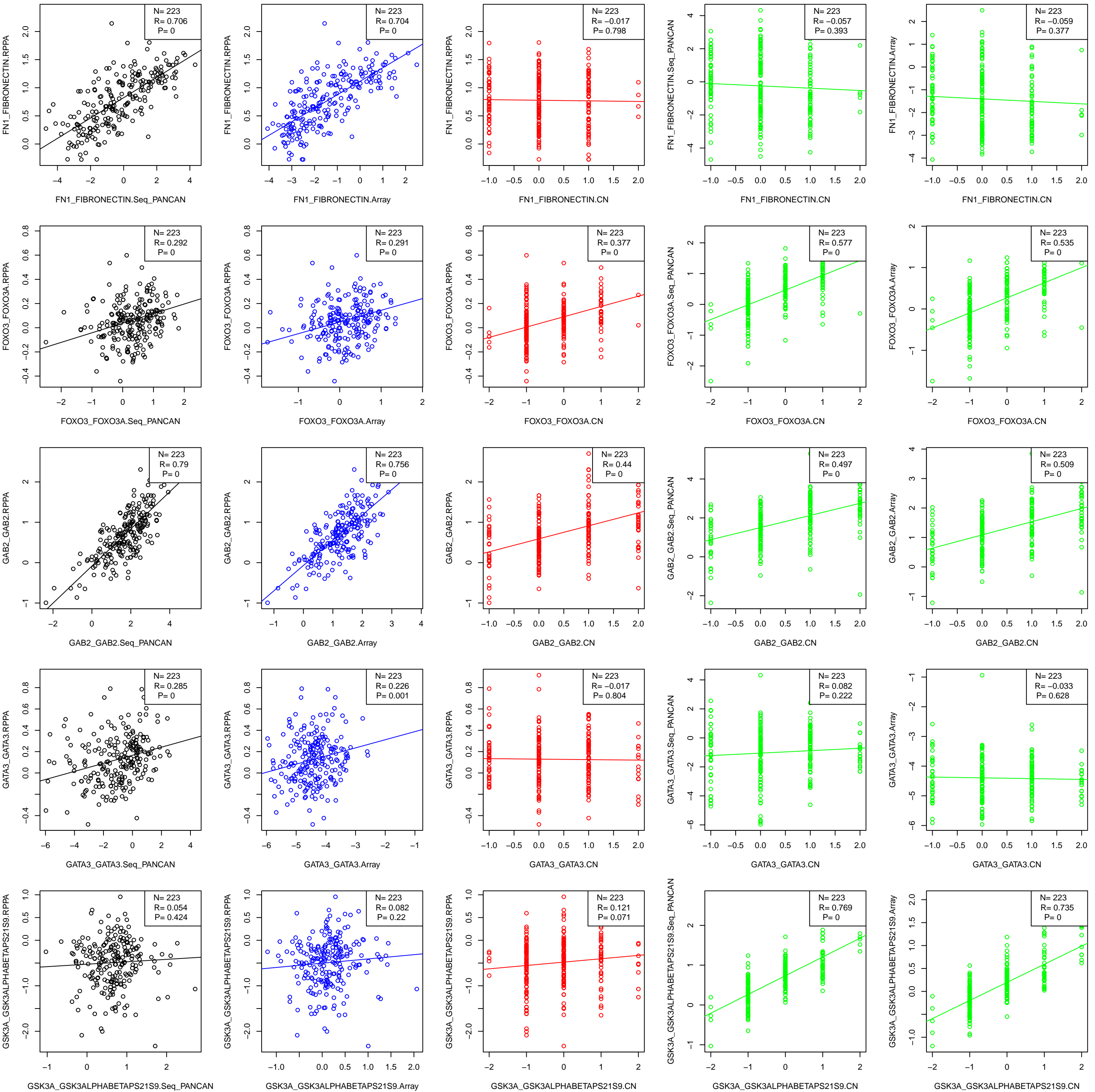

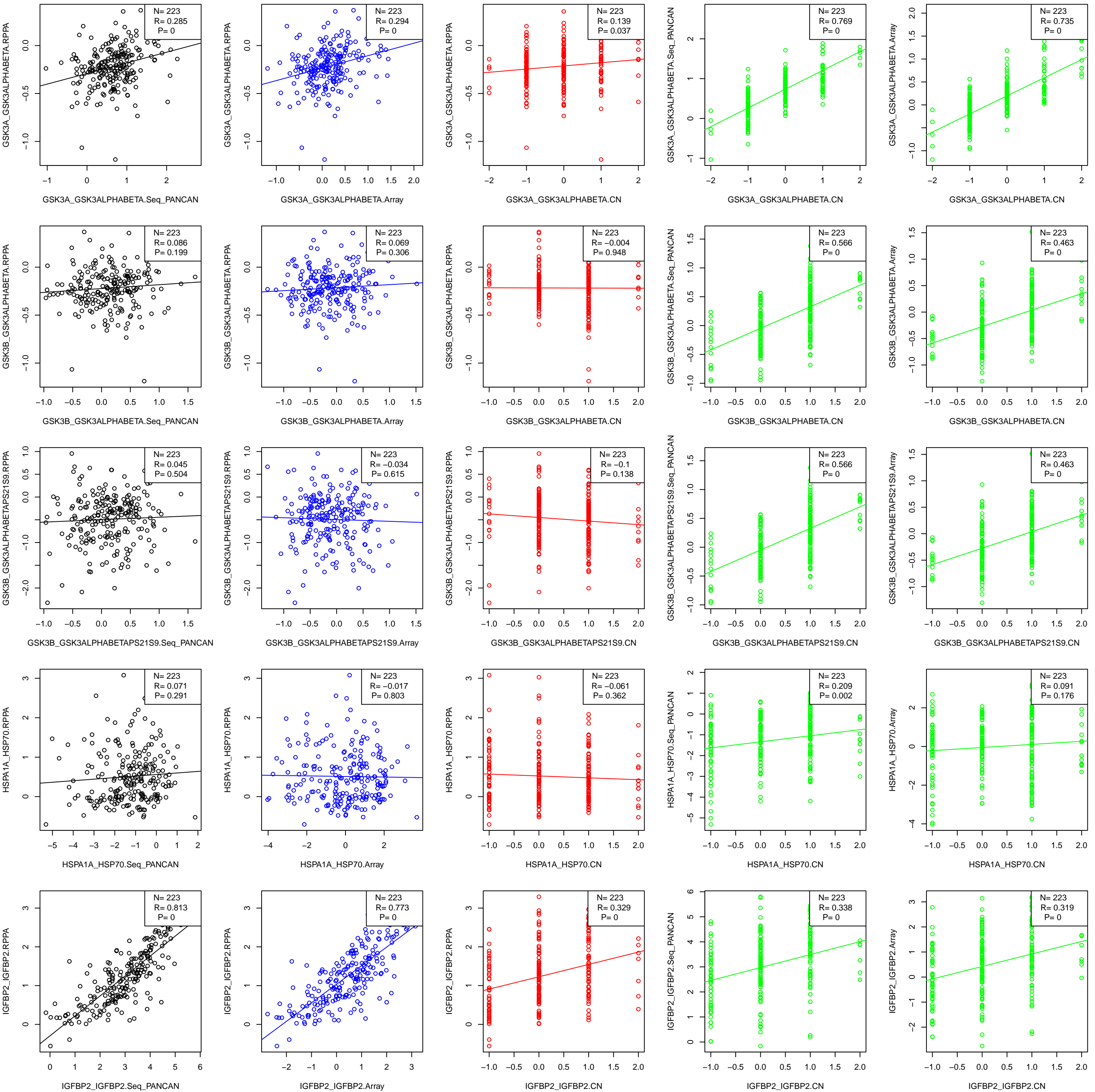

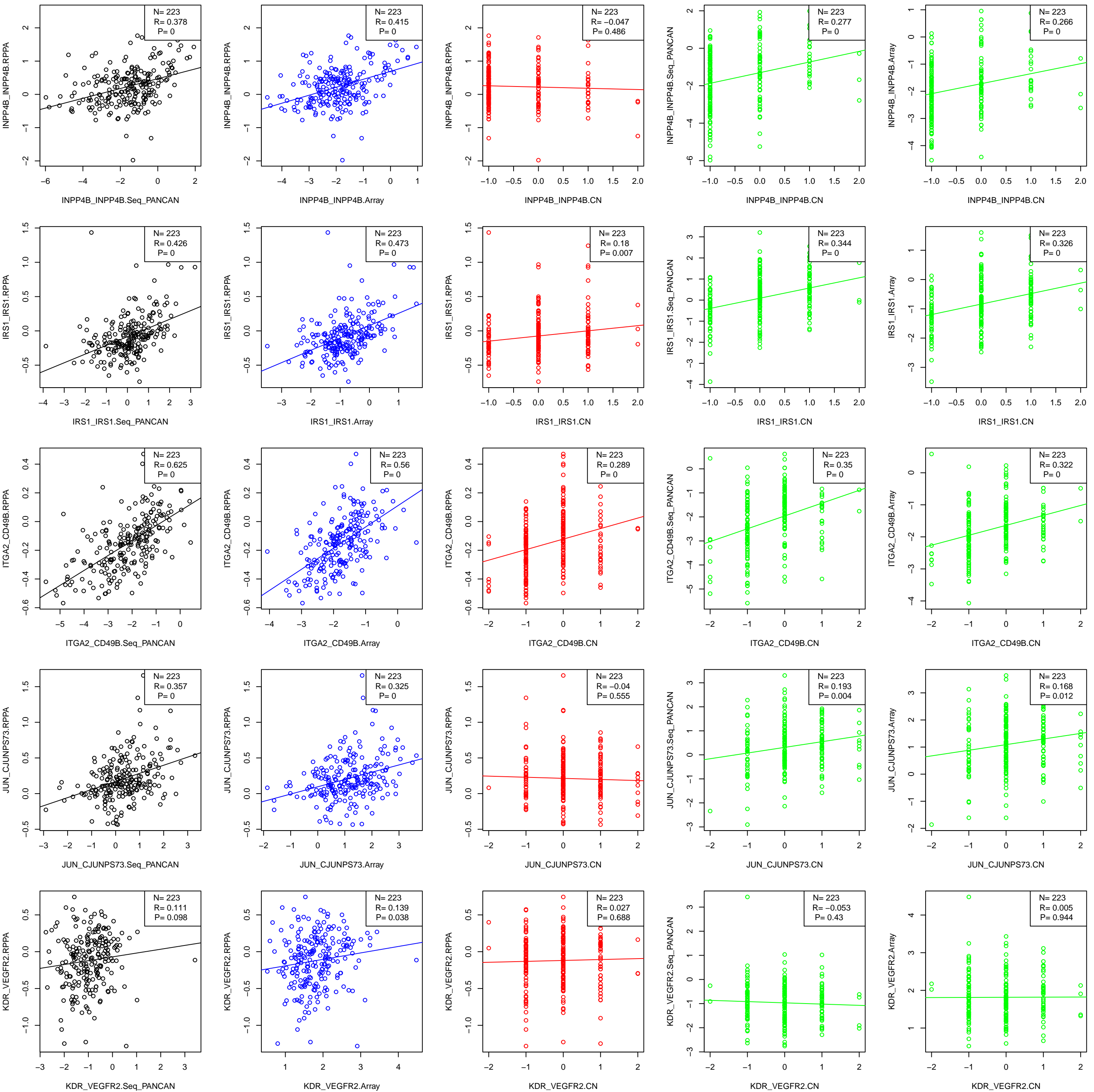

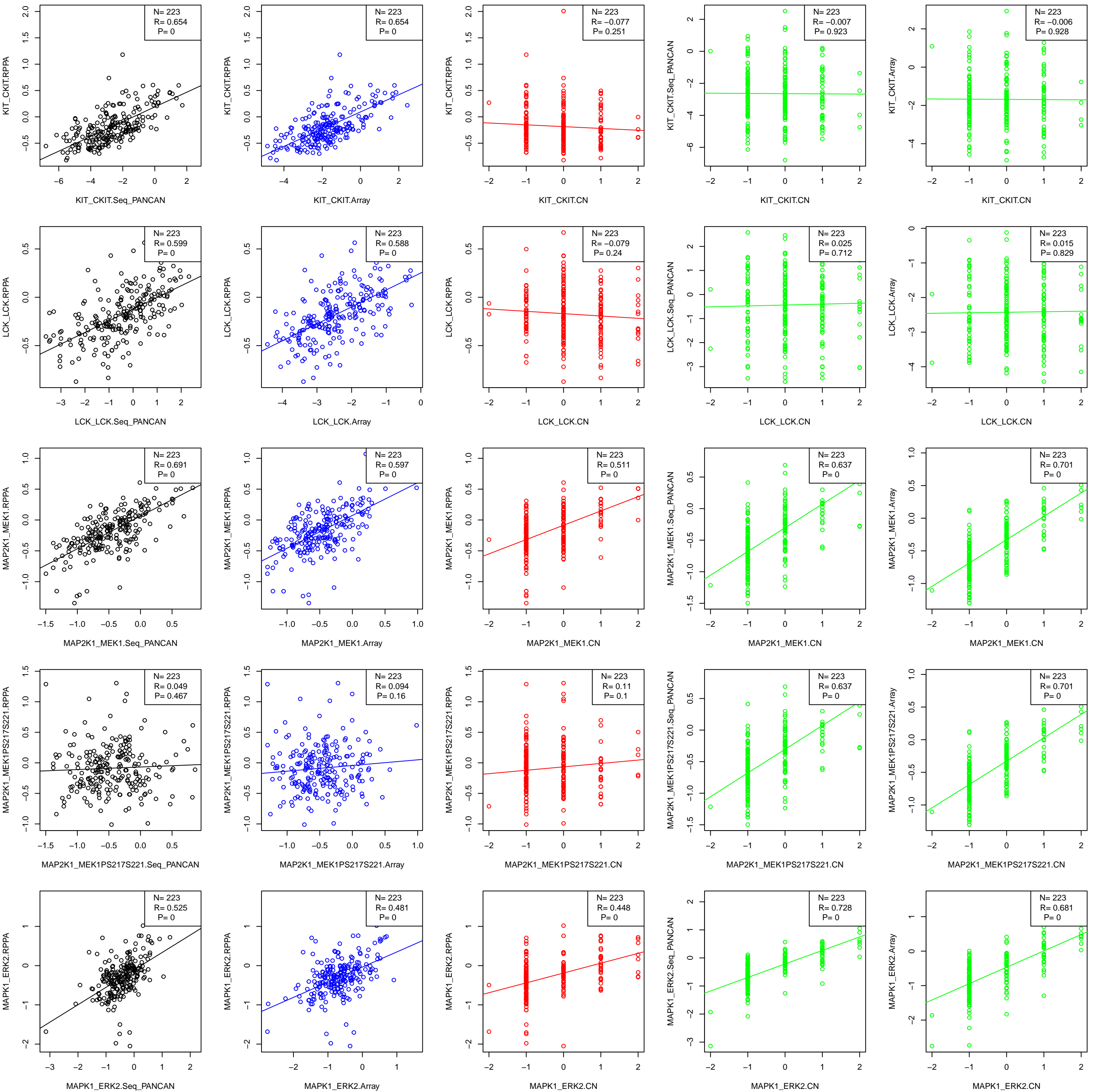

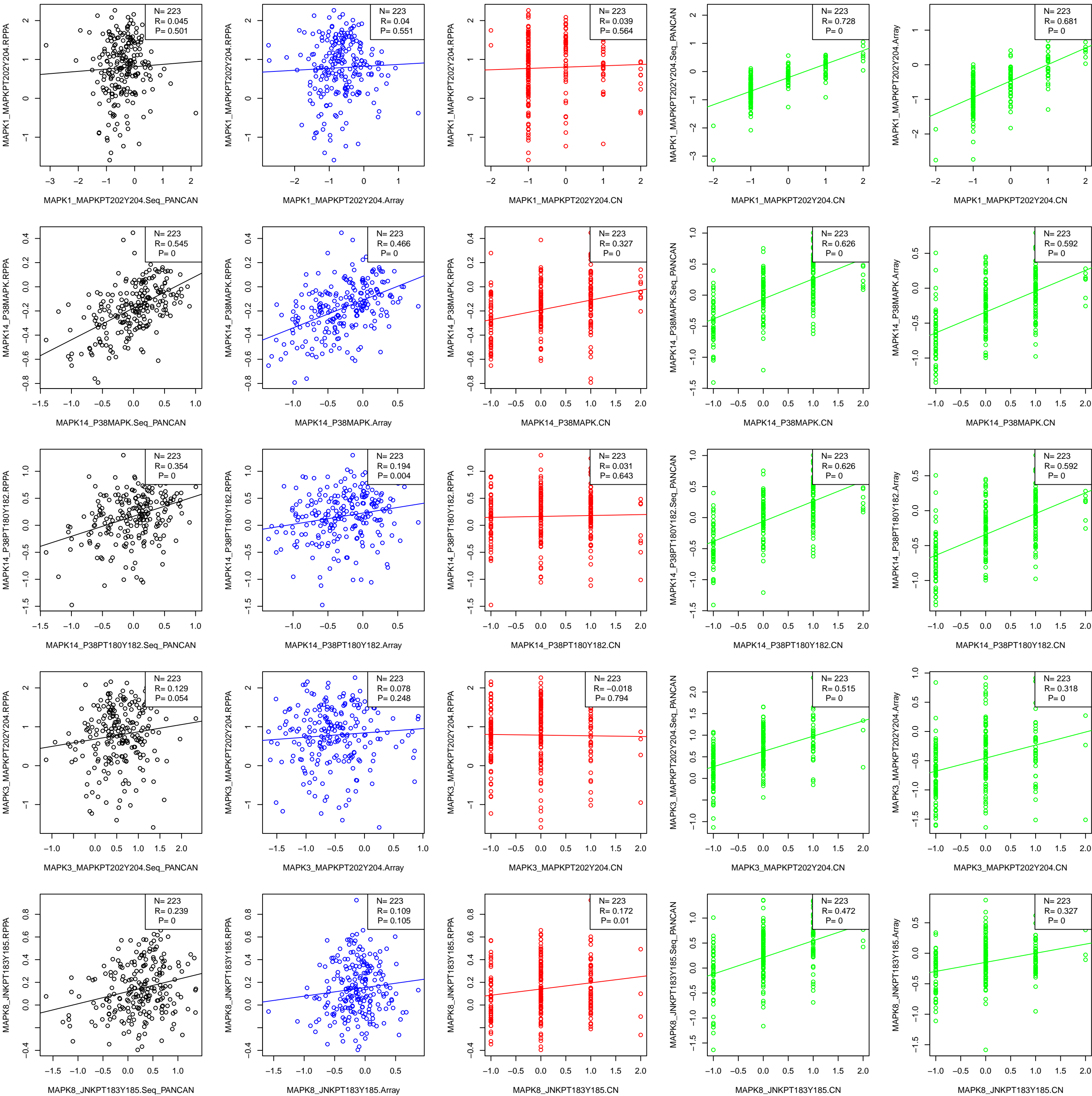

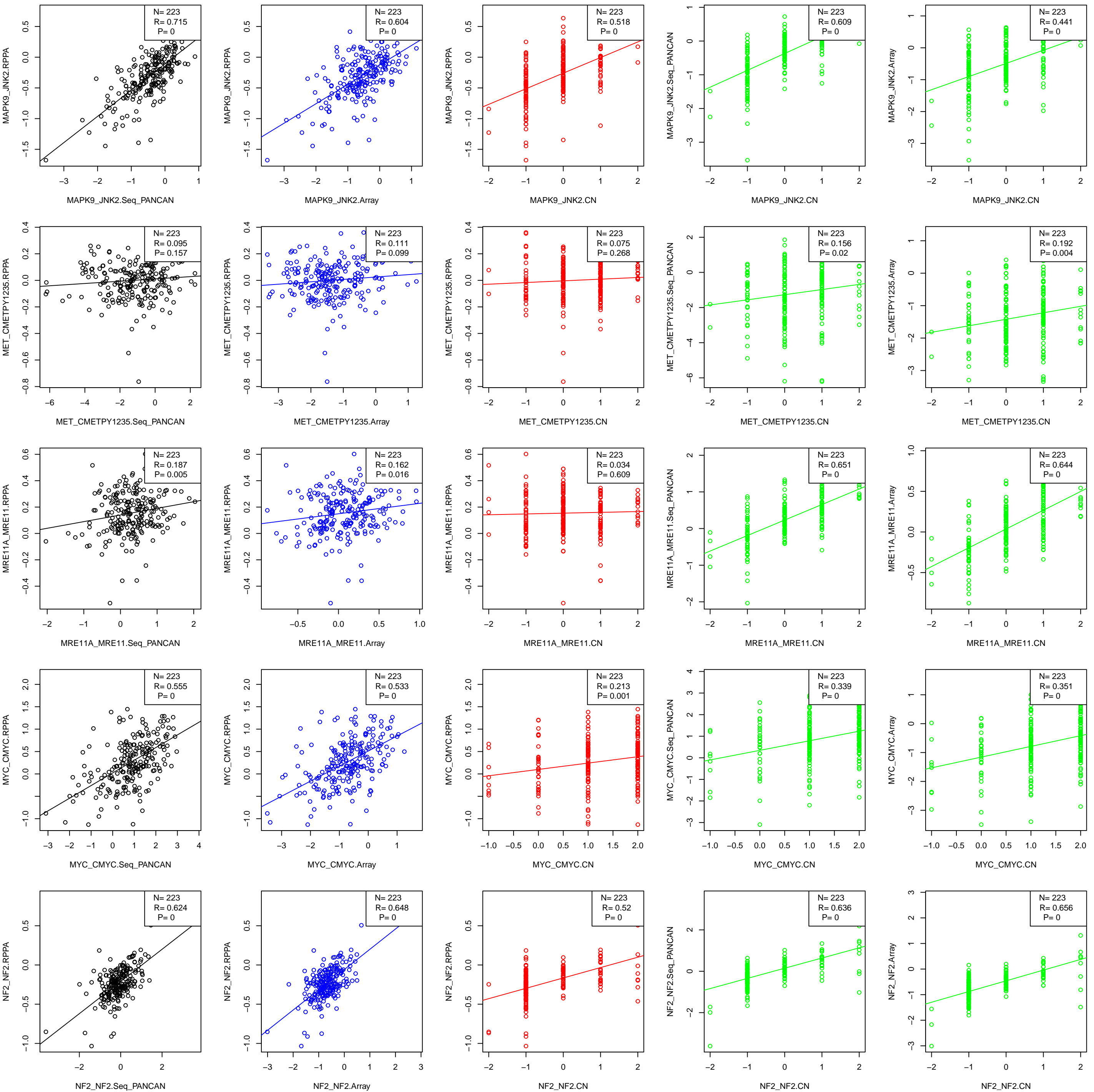

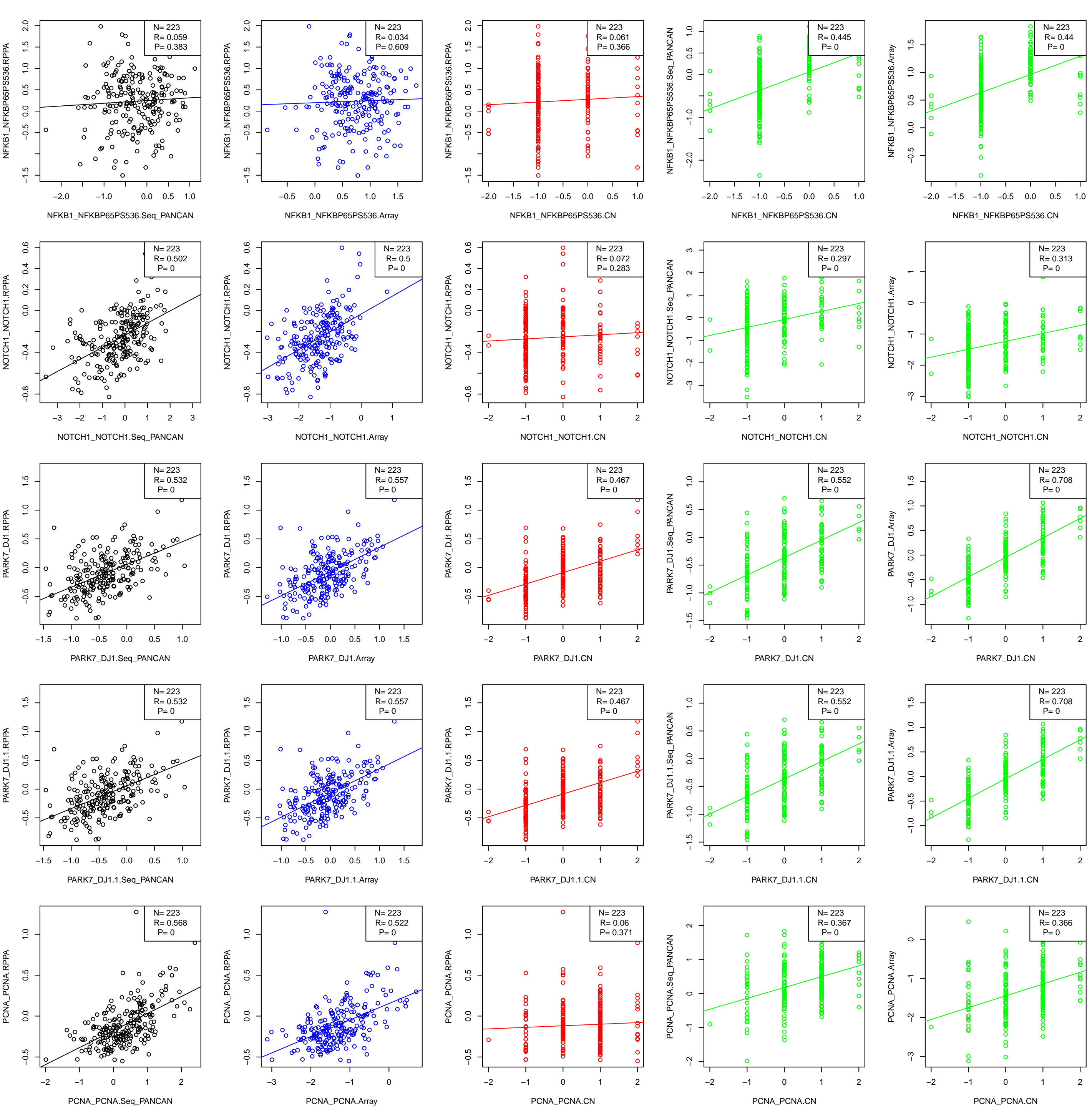

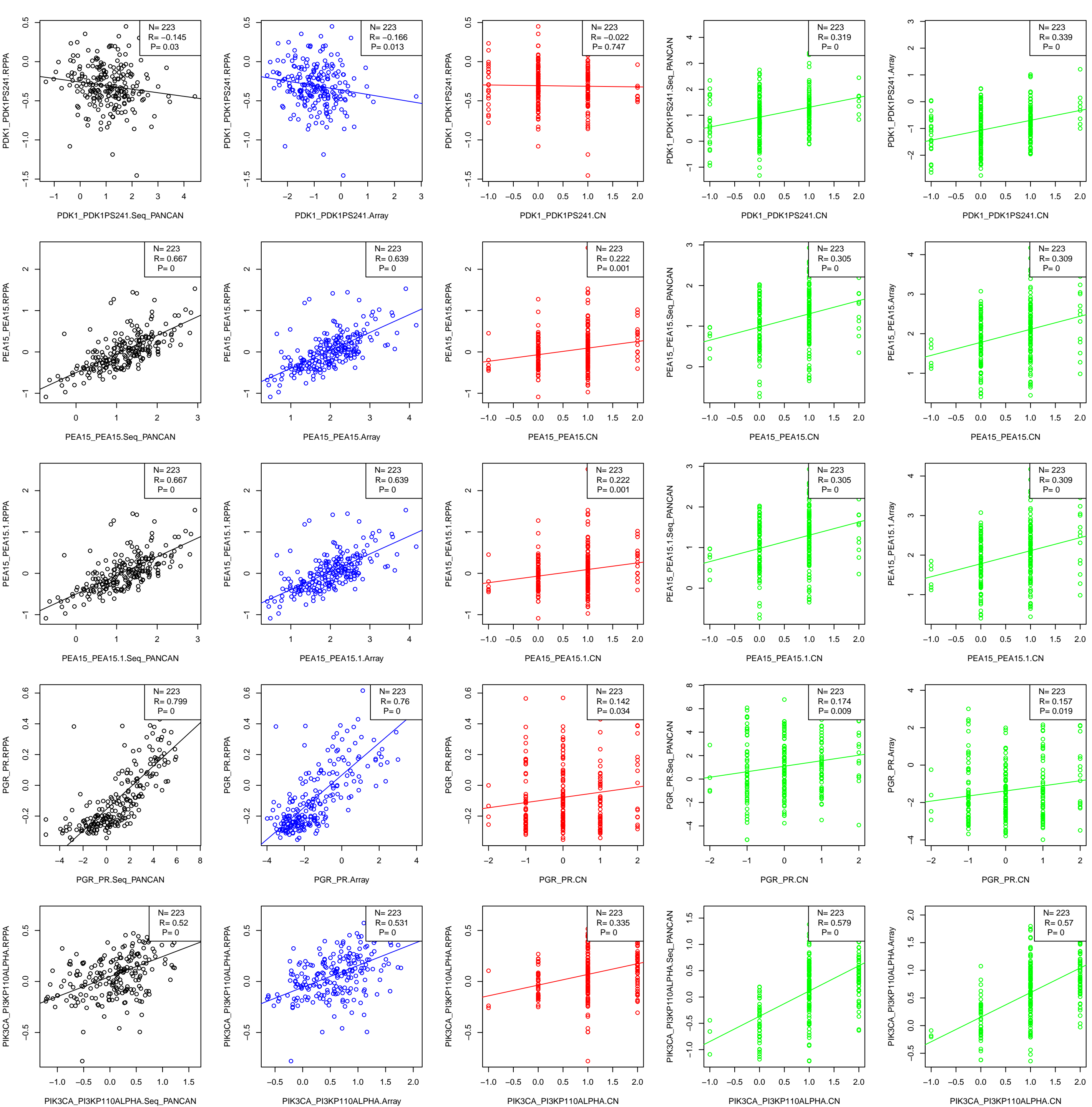

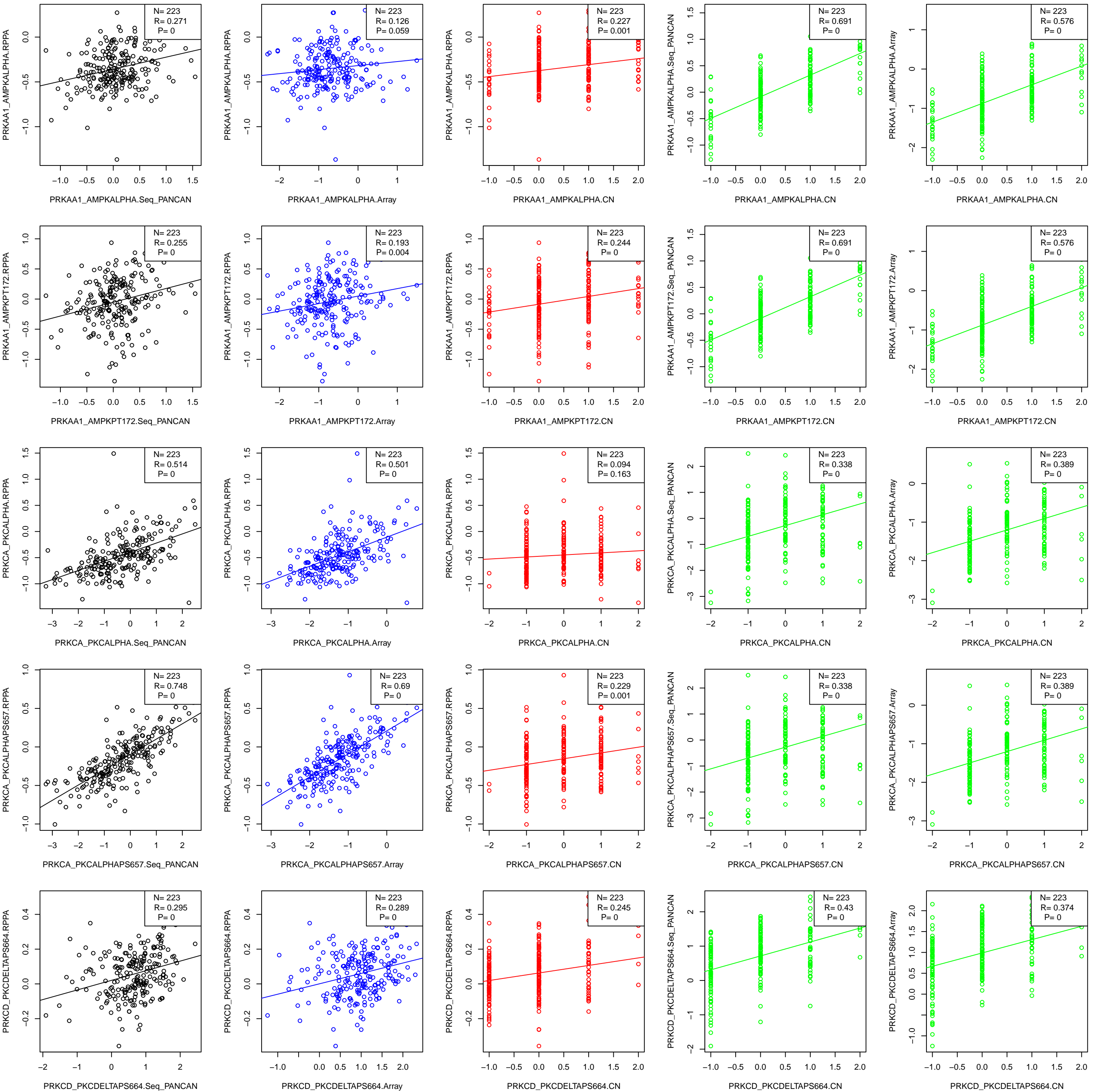

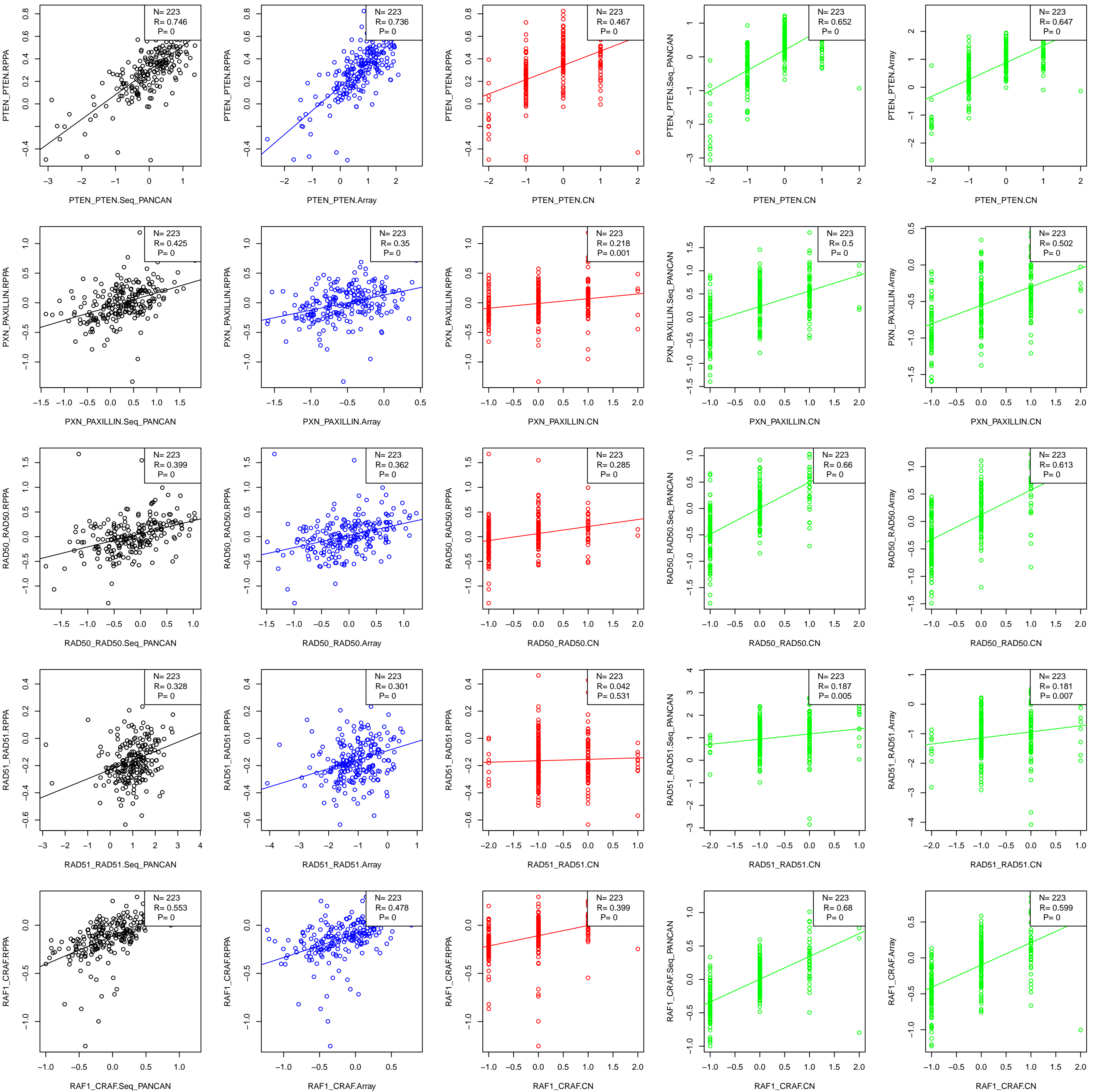

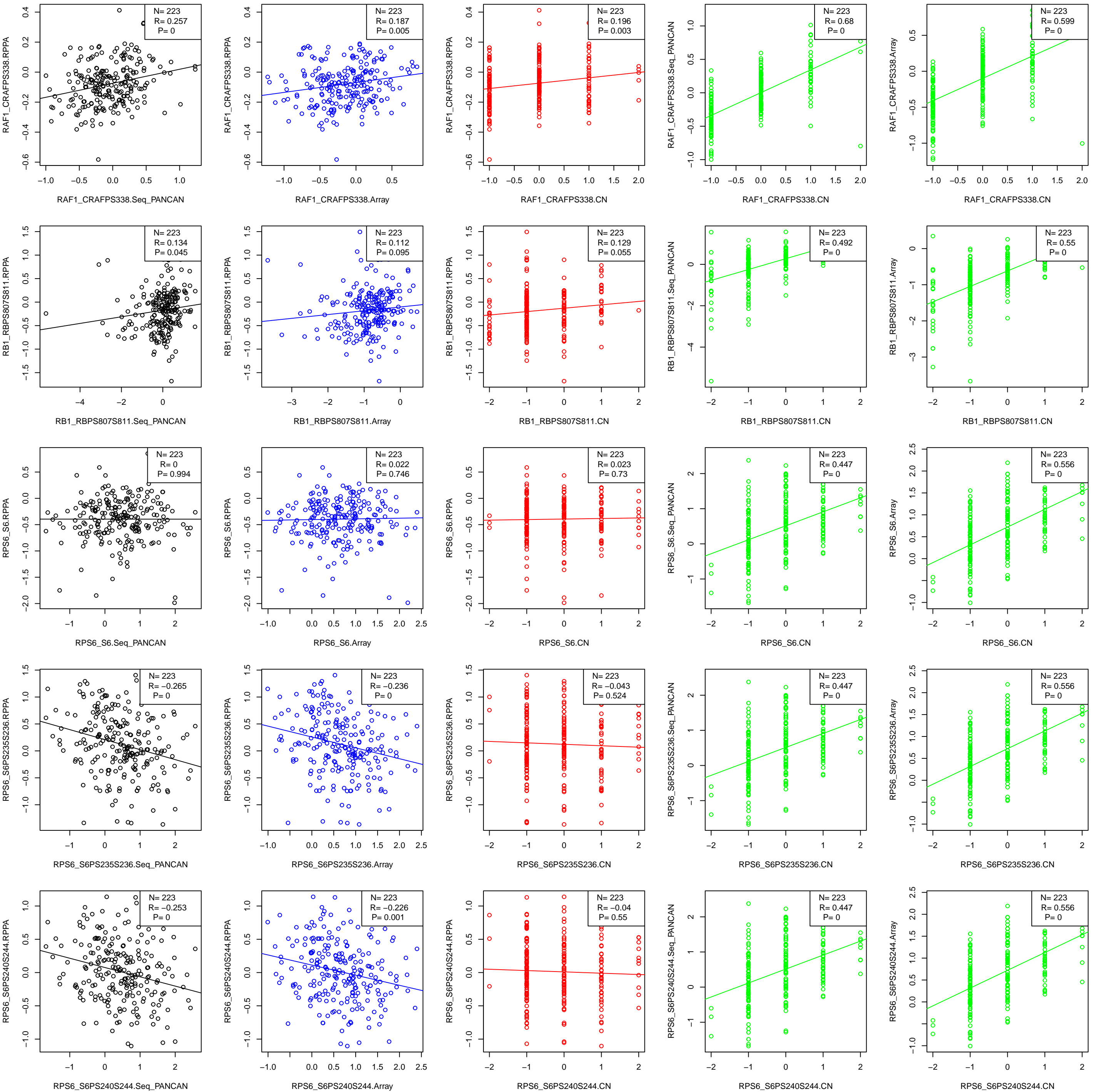

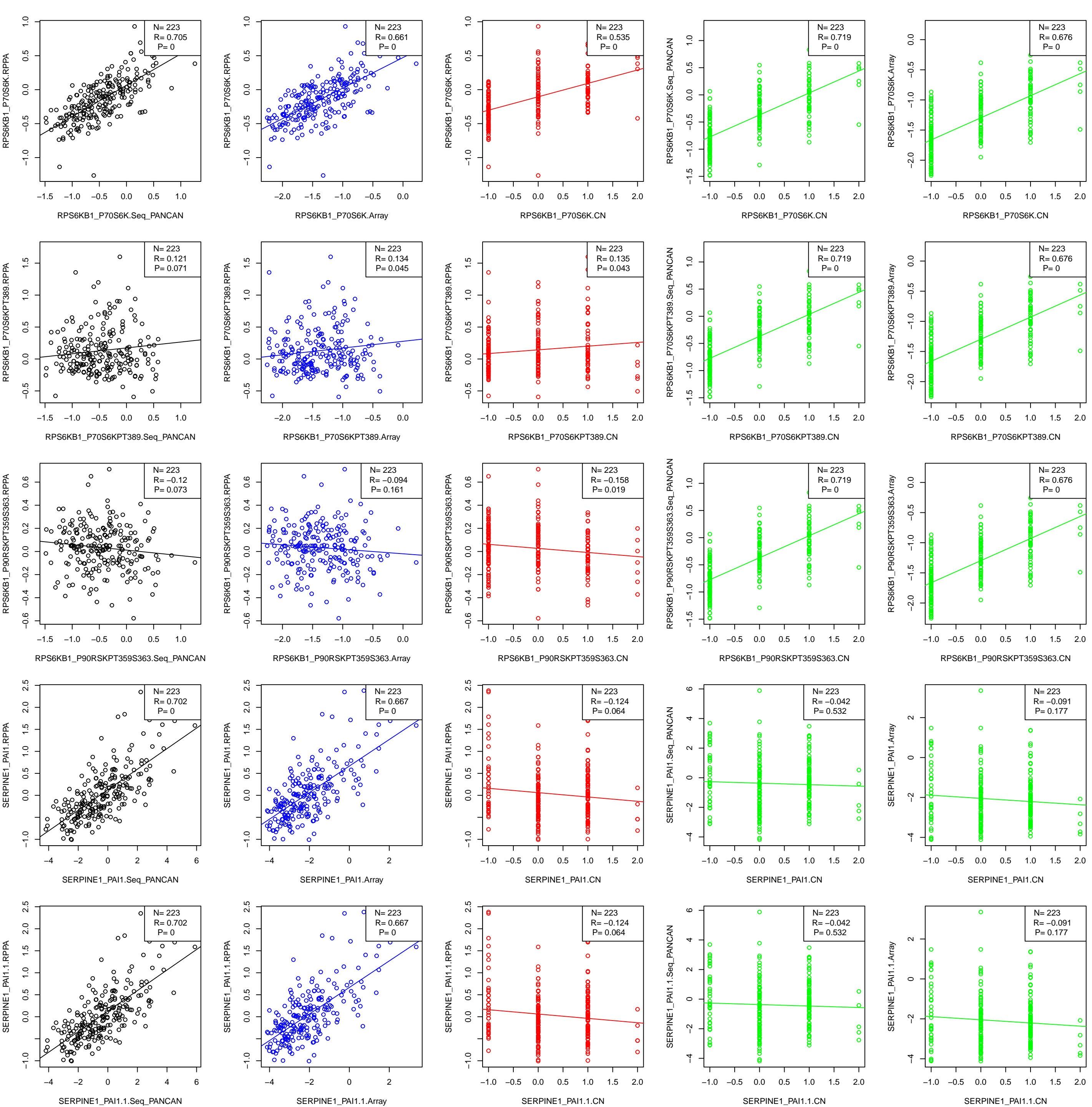

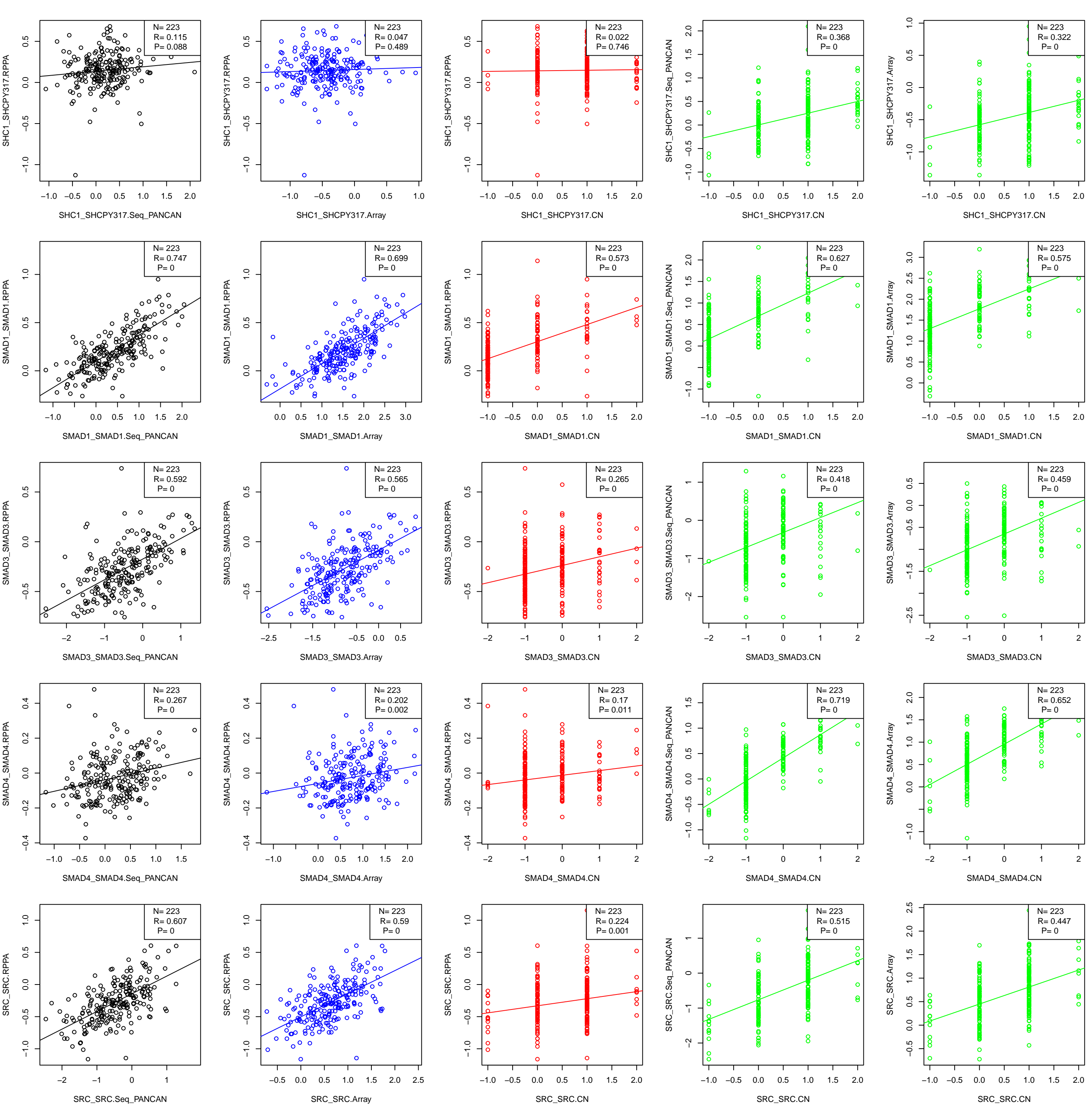

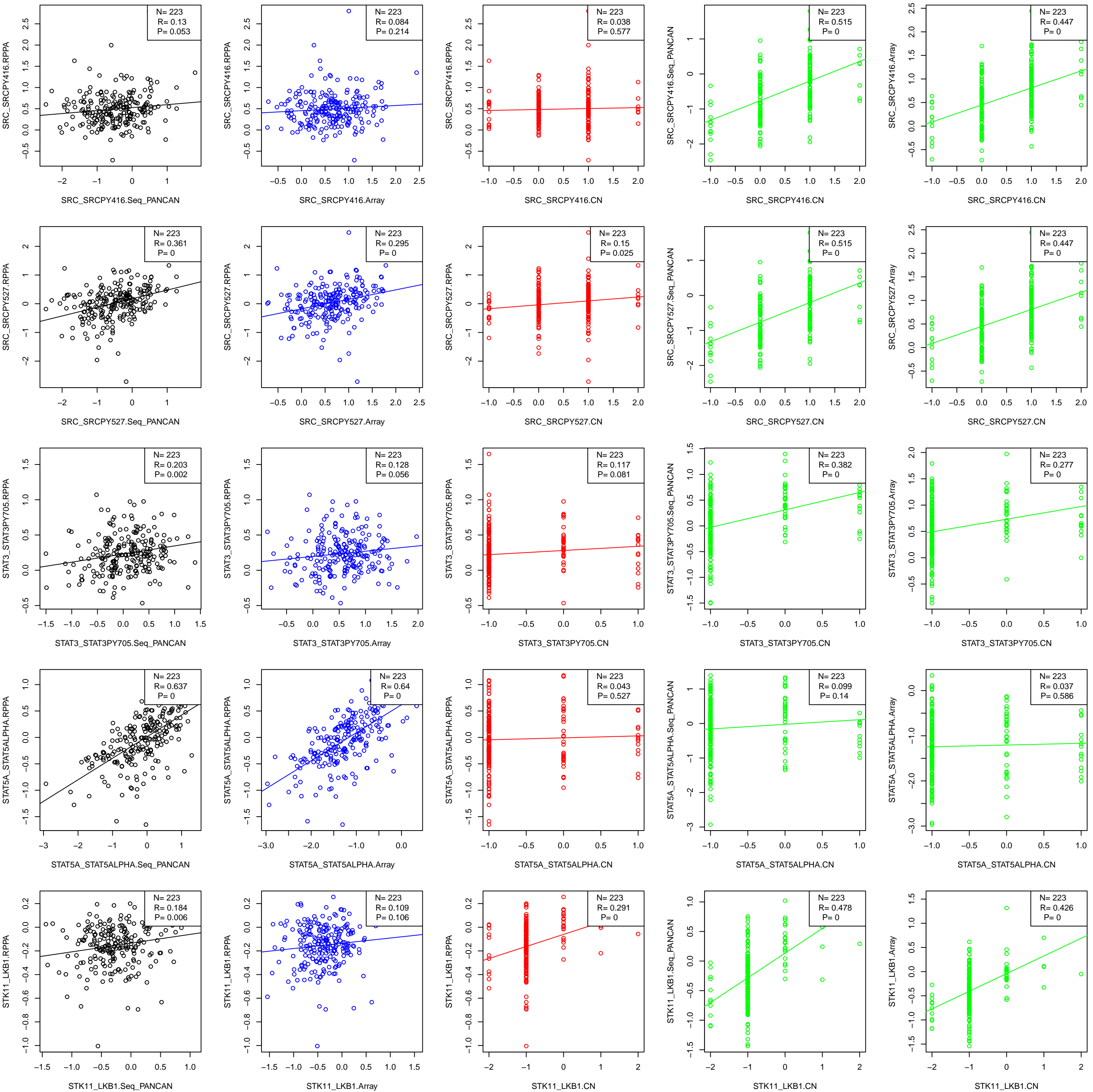

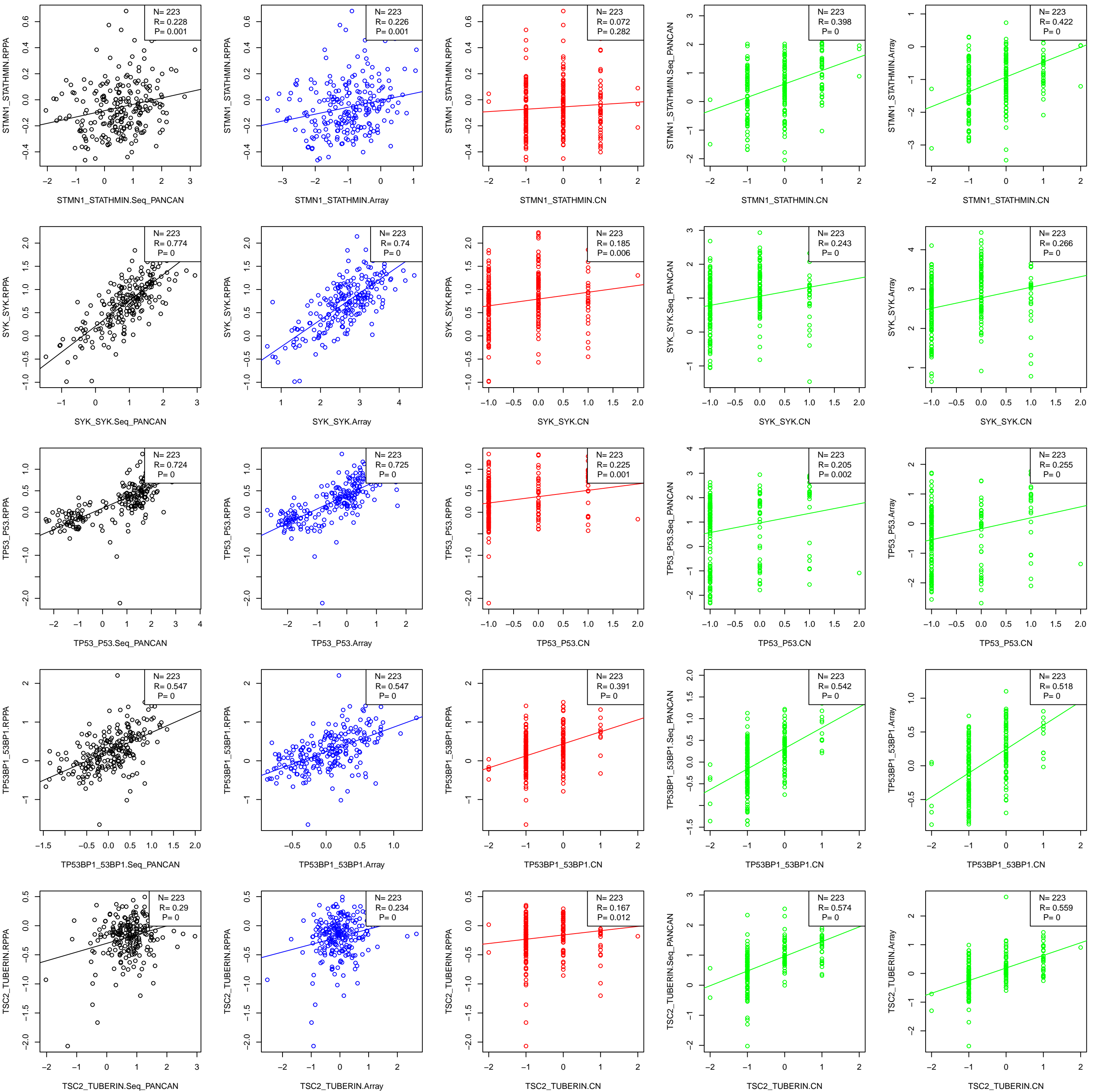

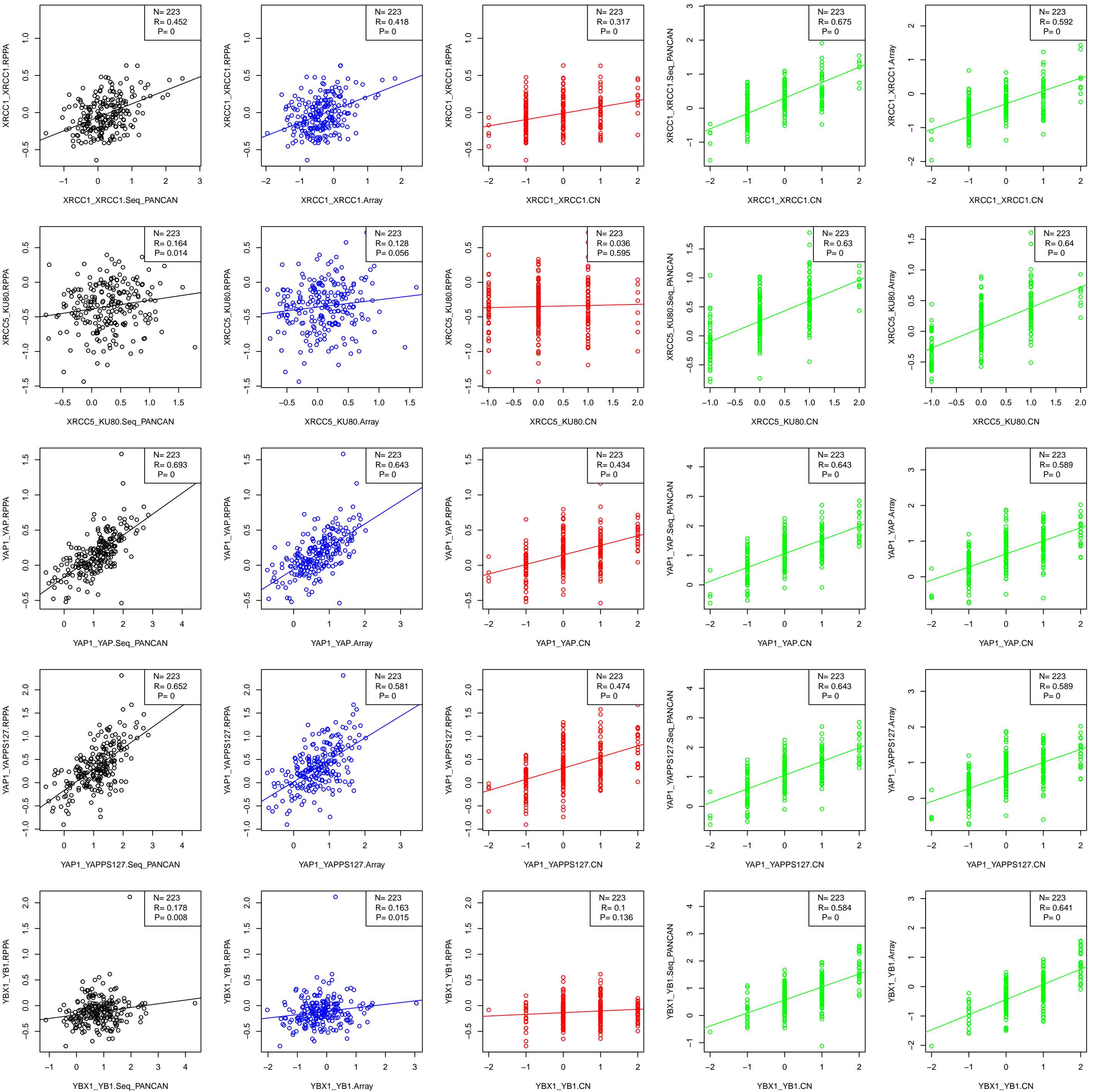

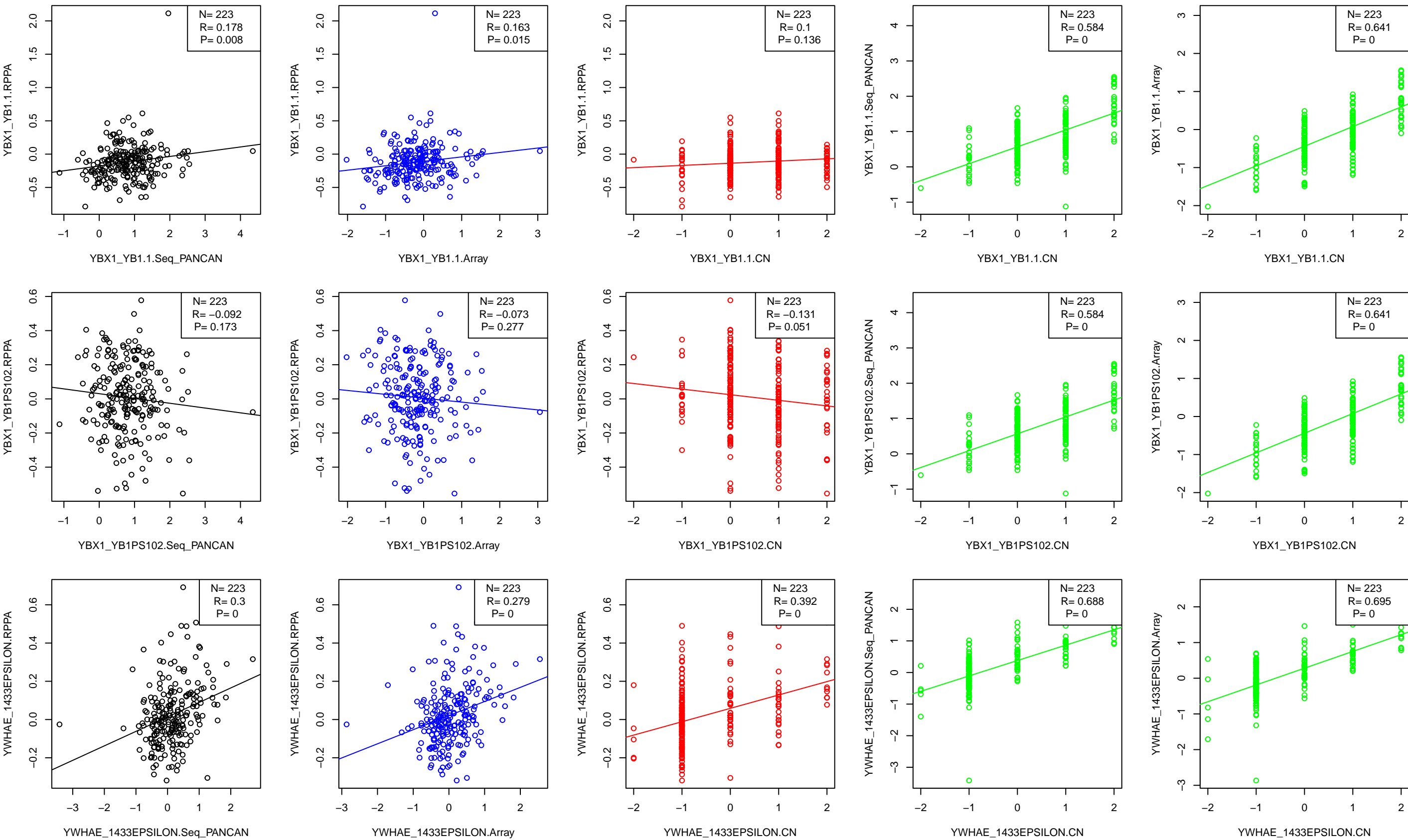

Supplement: Supplementary file 5 [file DataSheet6.PDF]

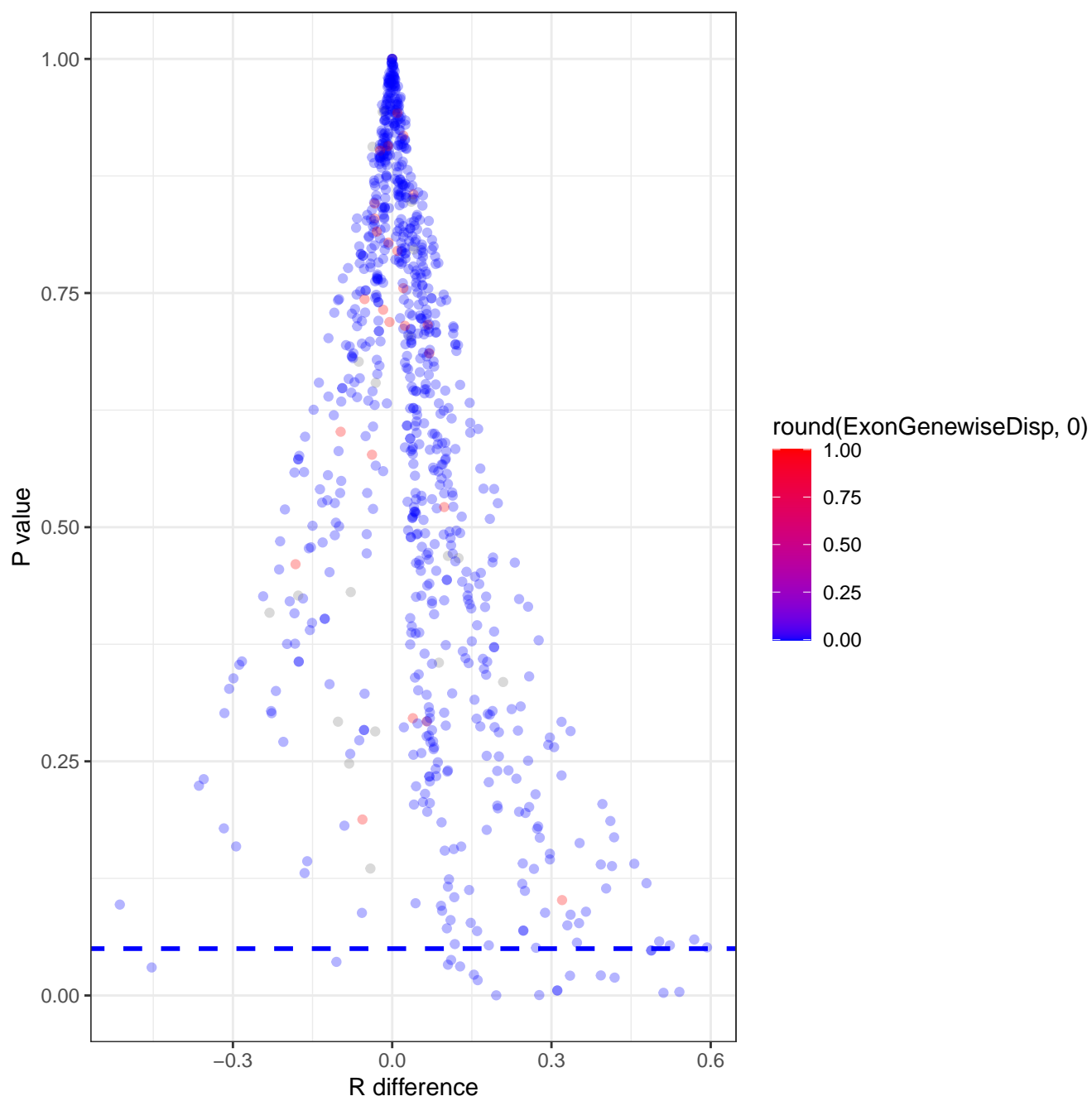

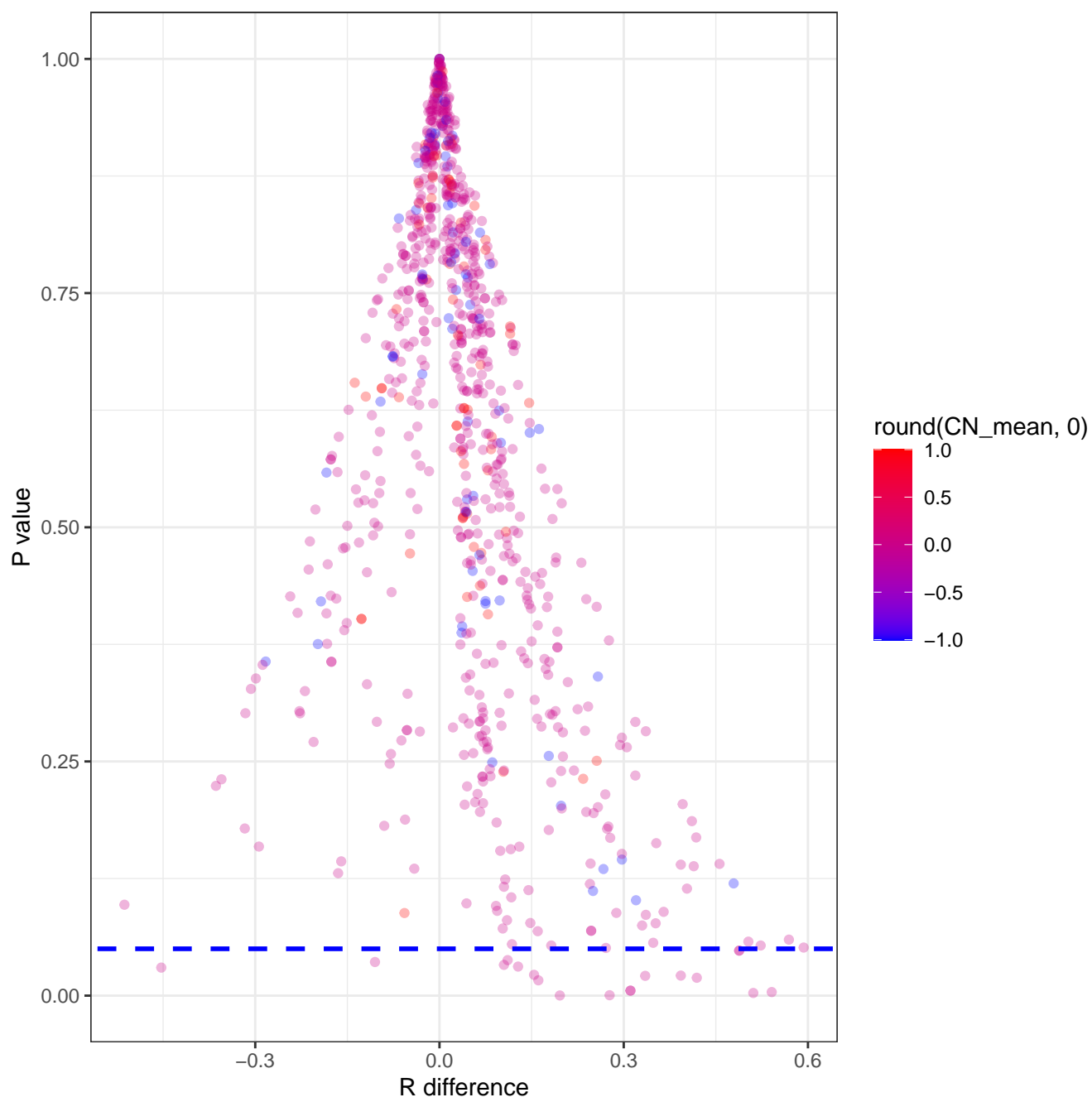

Supplement: Supplementary file 6 [file DataSheet14.PDF]

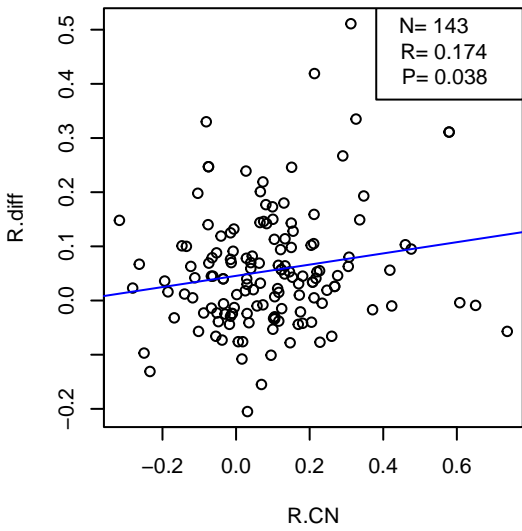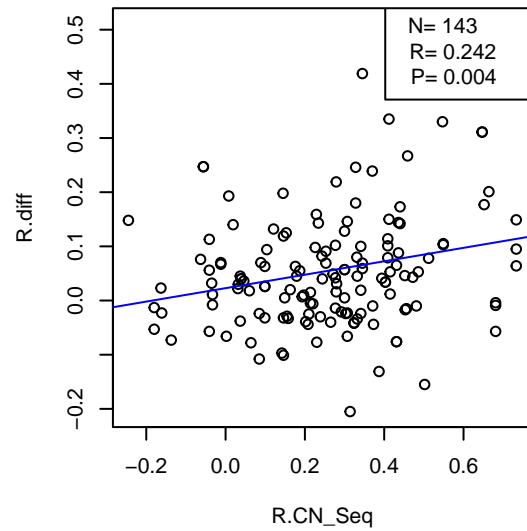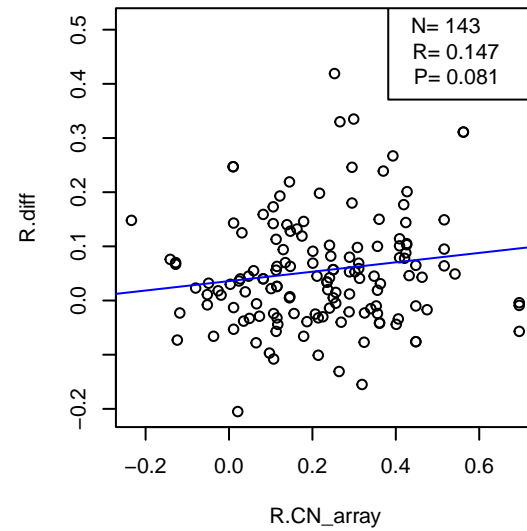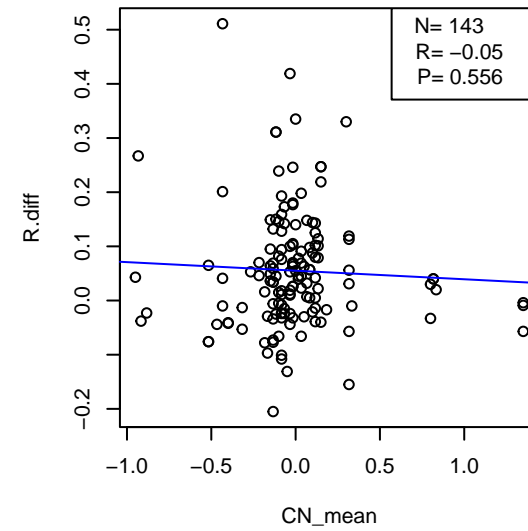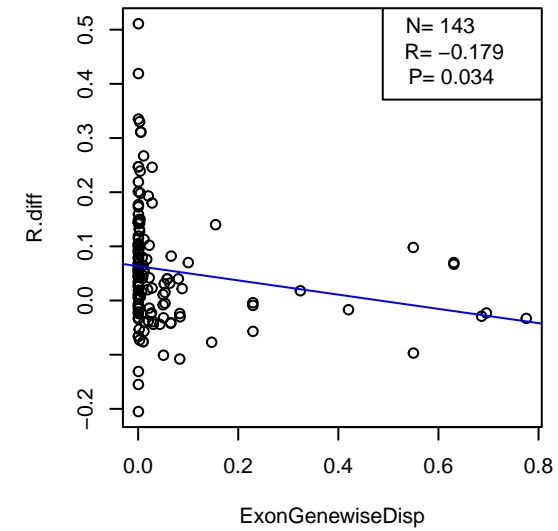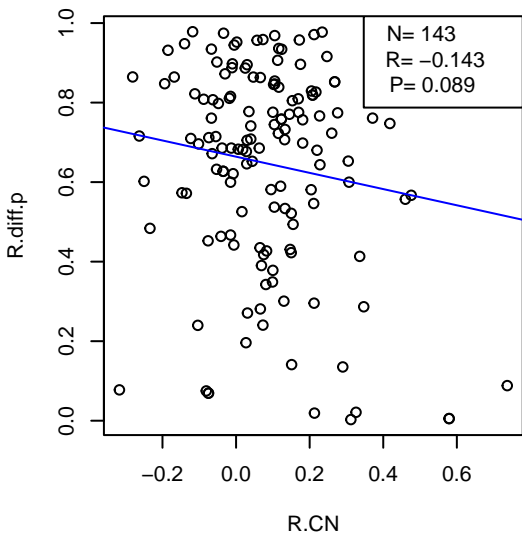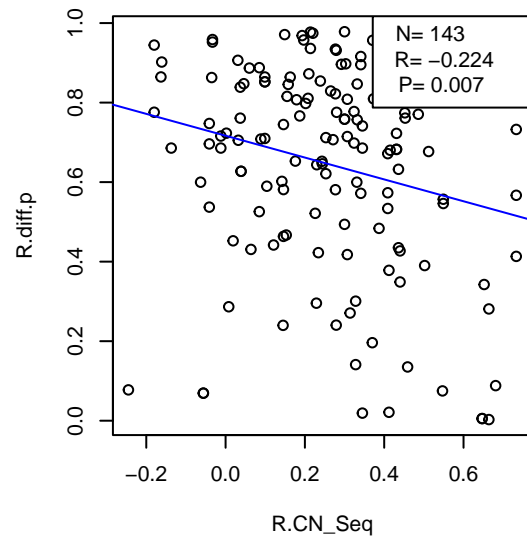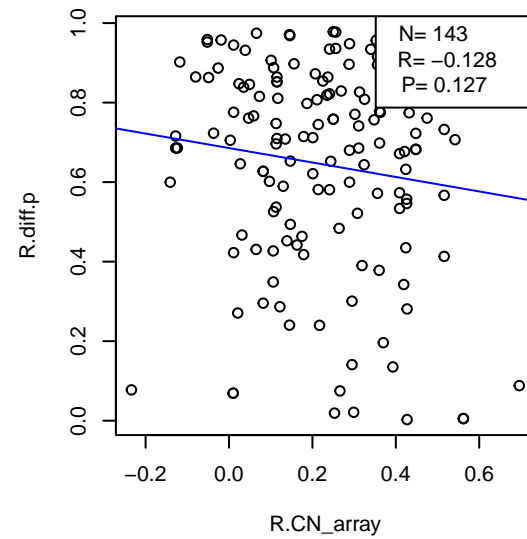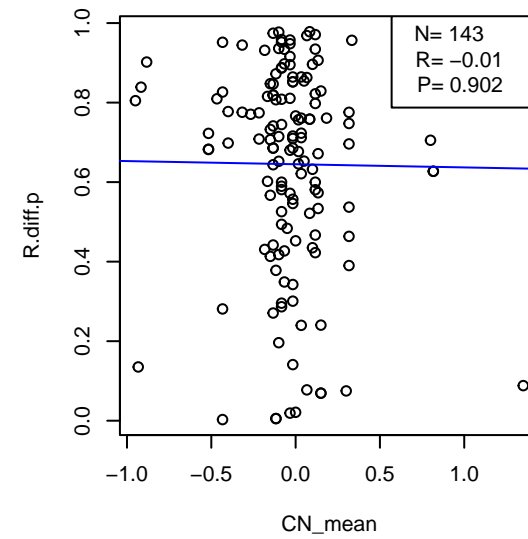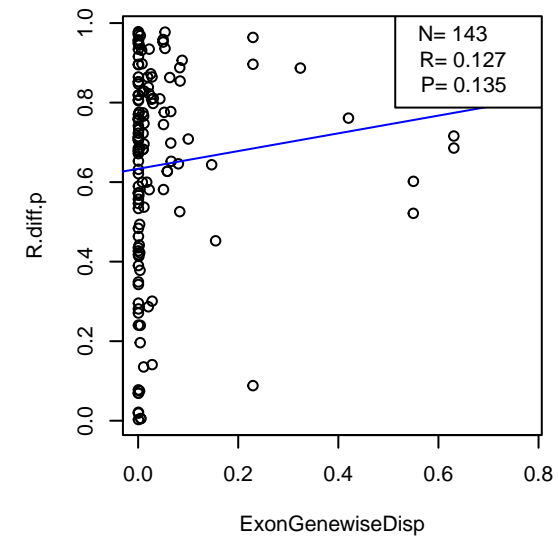

Supplement: Supplementary file 7 [file DataSheet9.PDF]

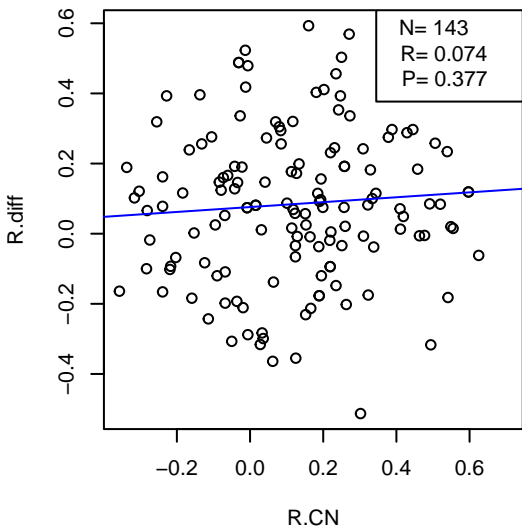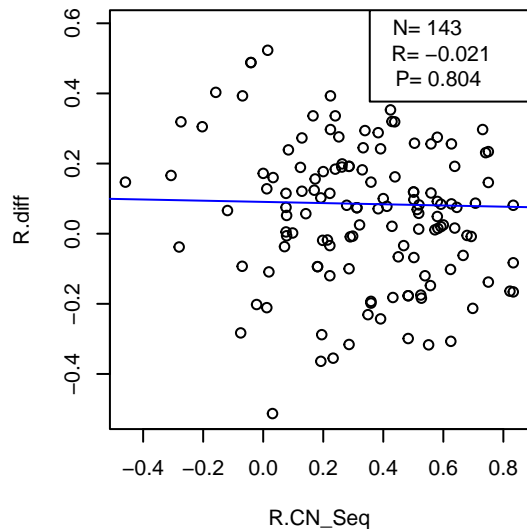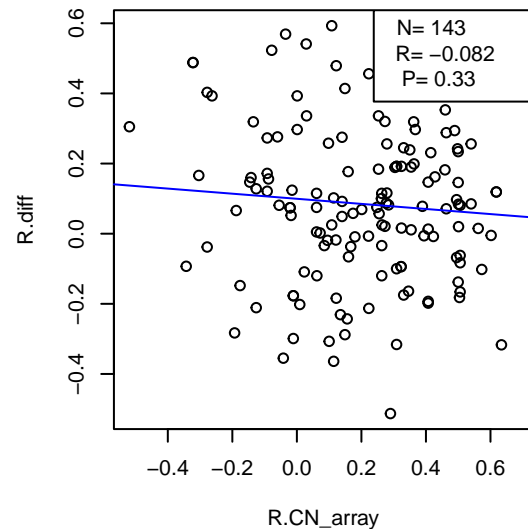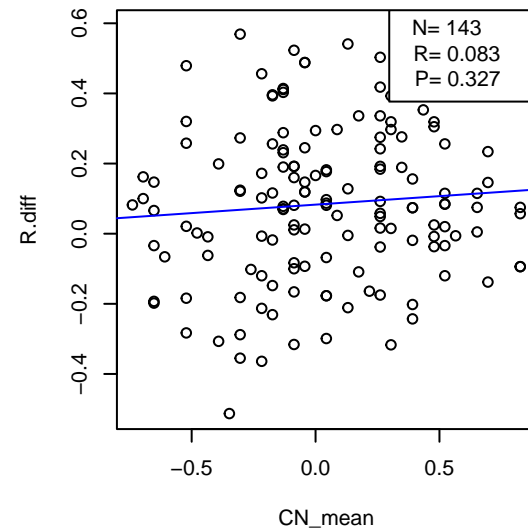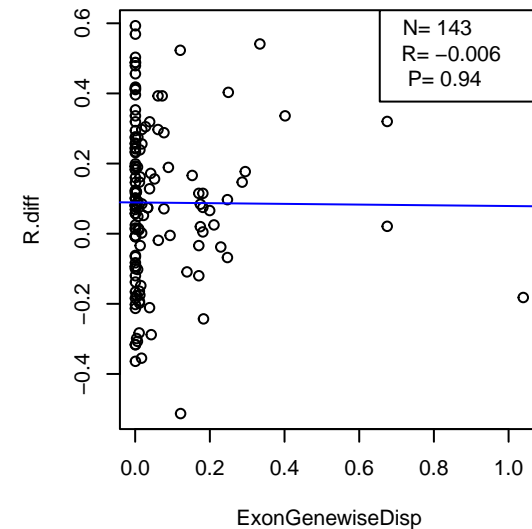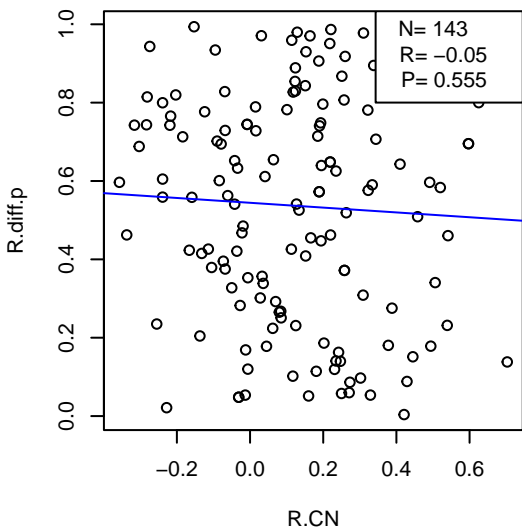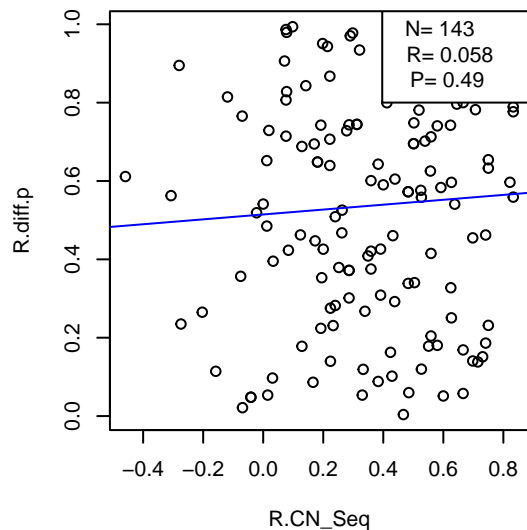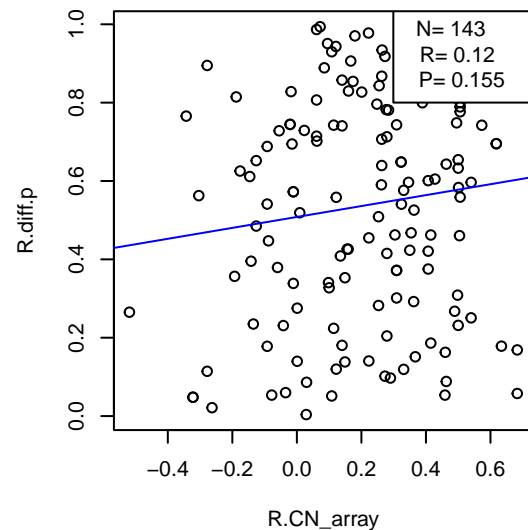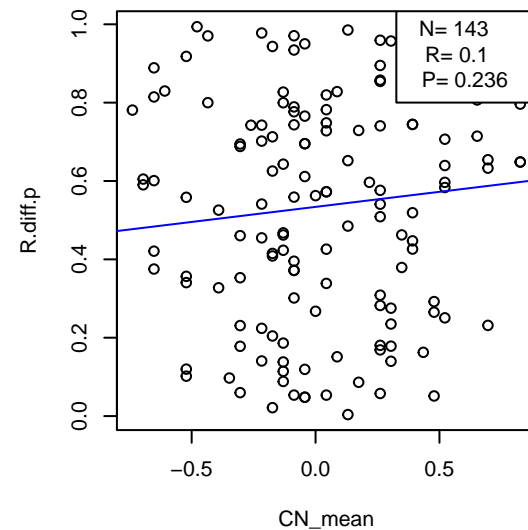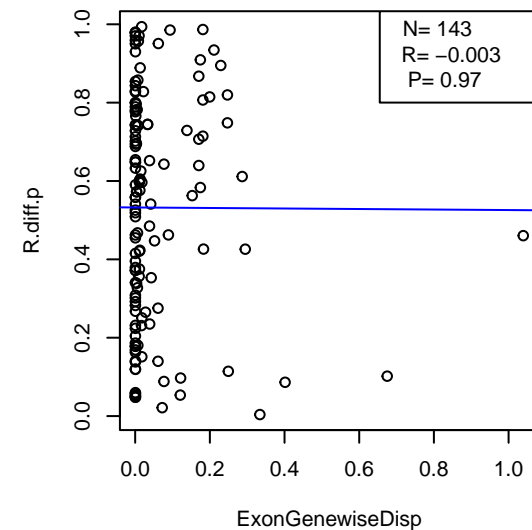

Supplement: Supplementary file 9 [file DataSheet11.PDF]

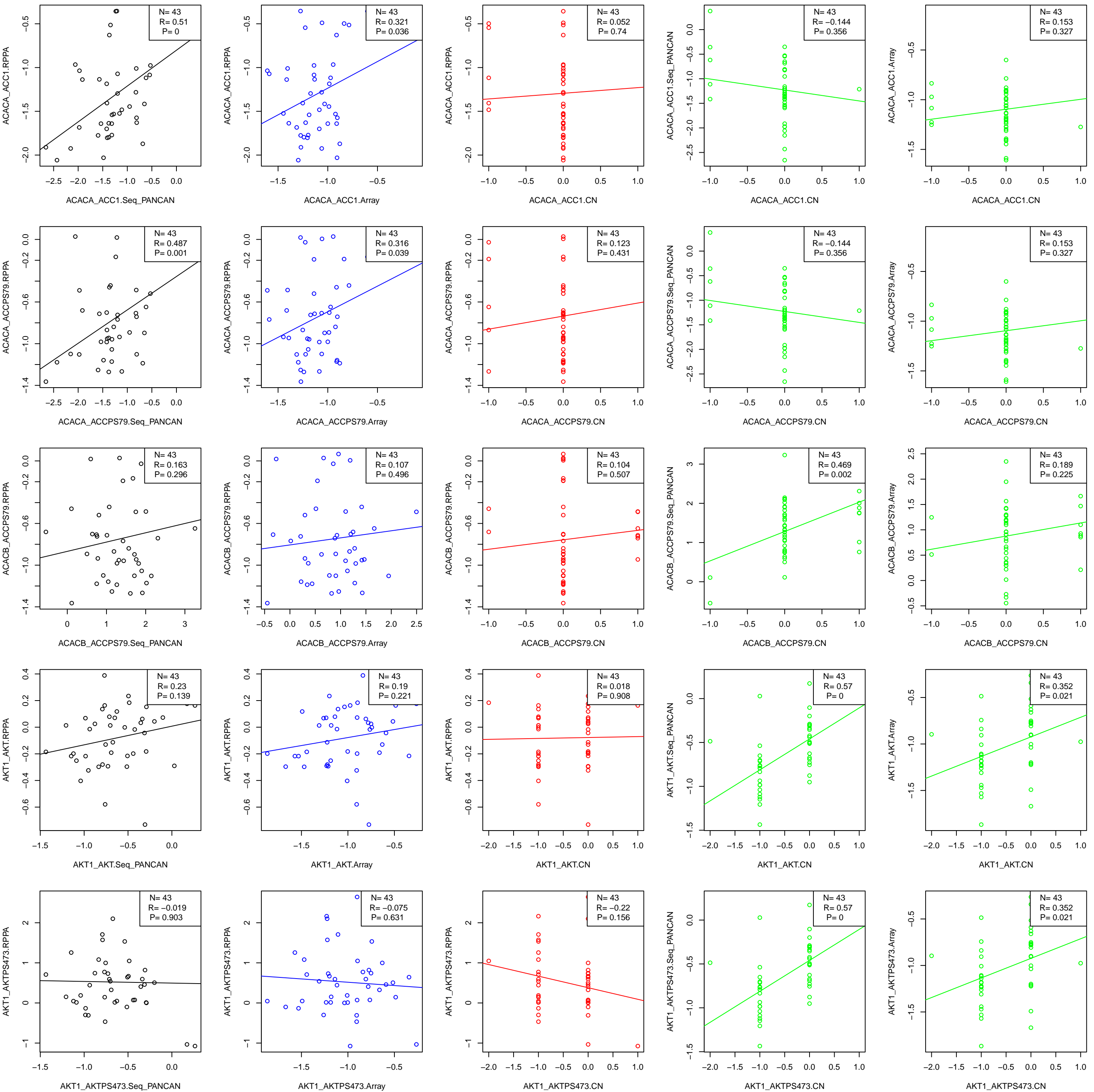

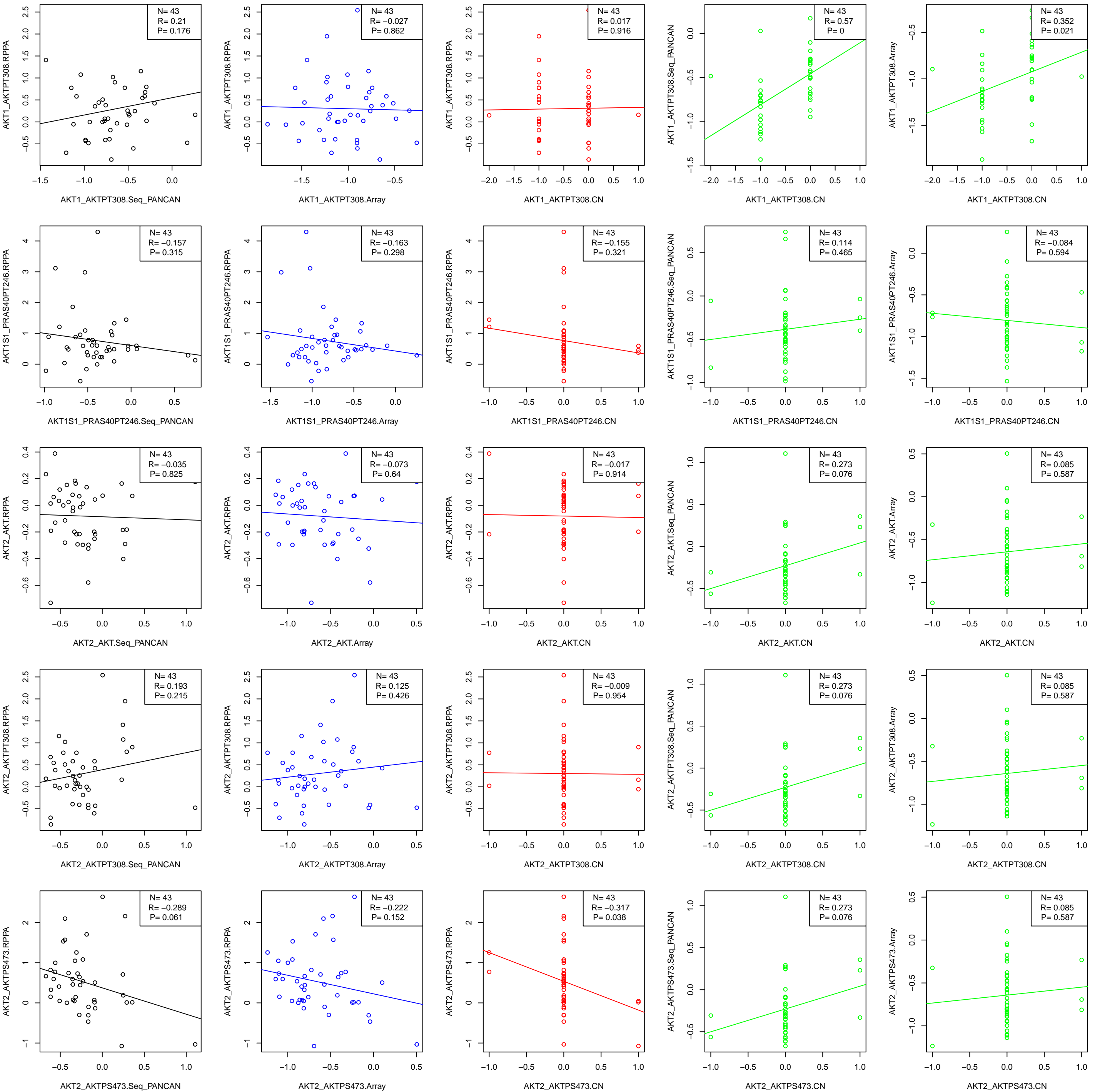

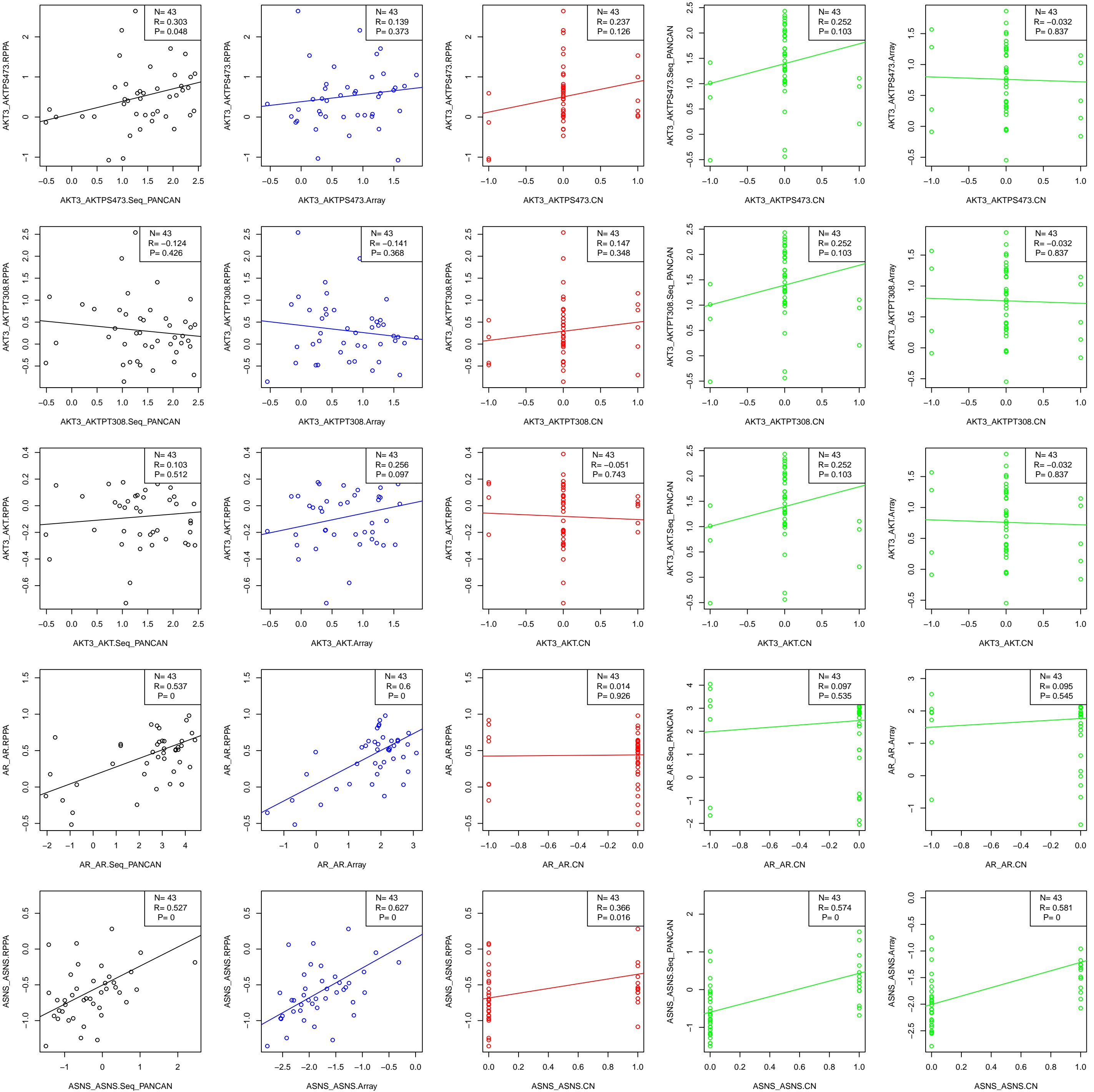

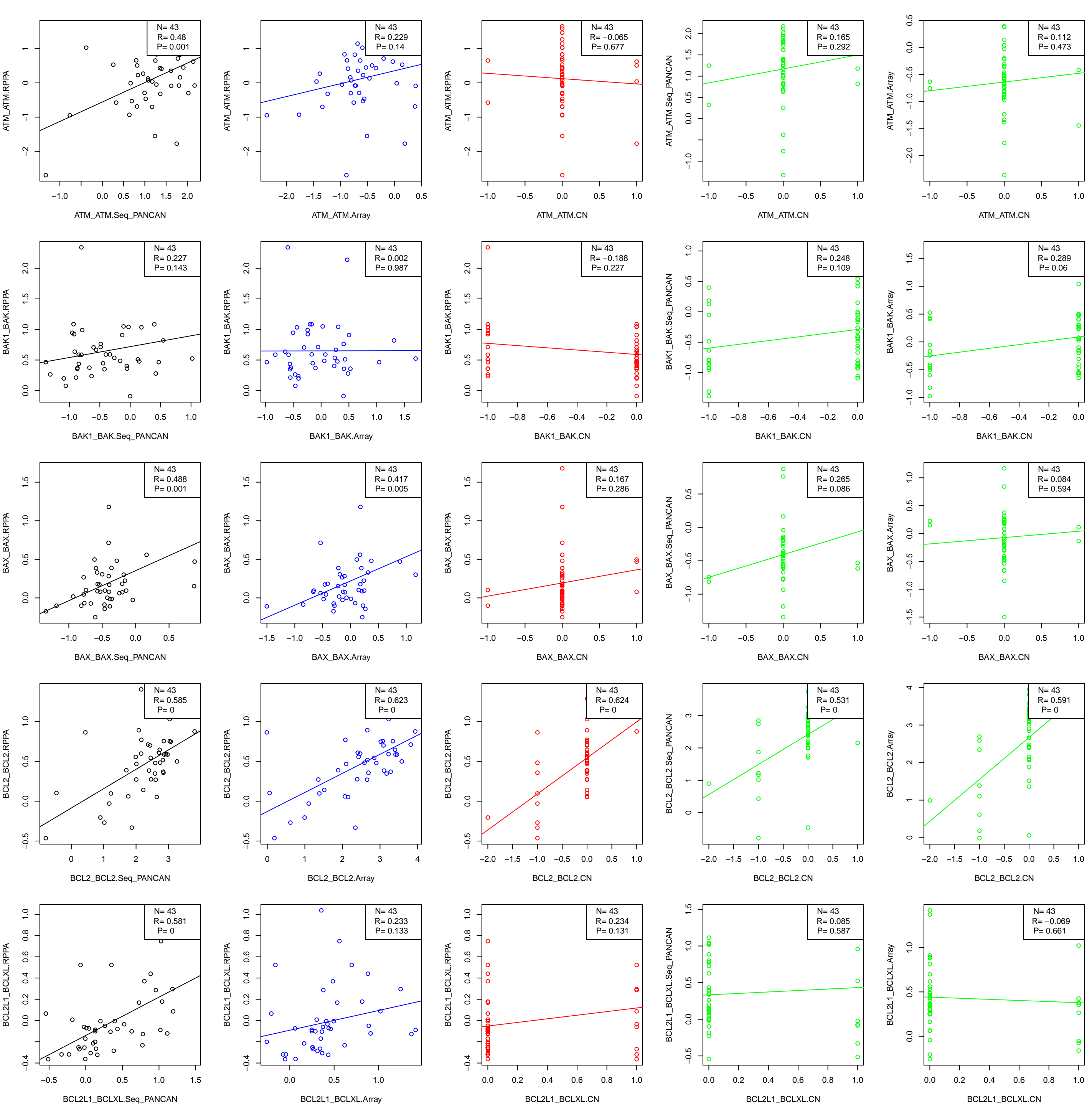

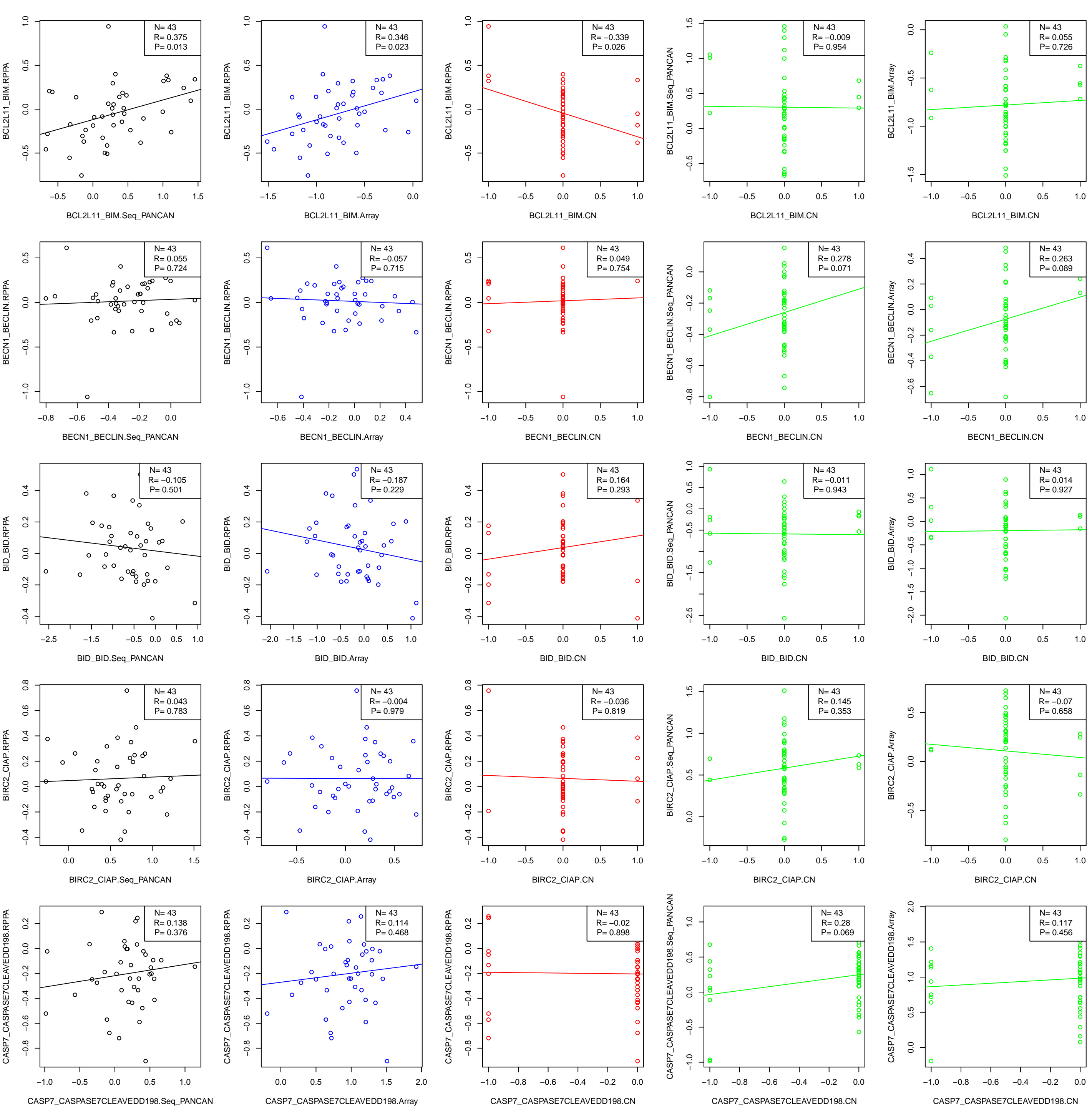

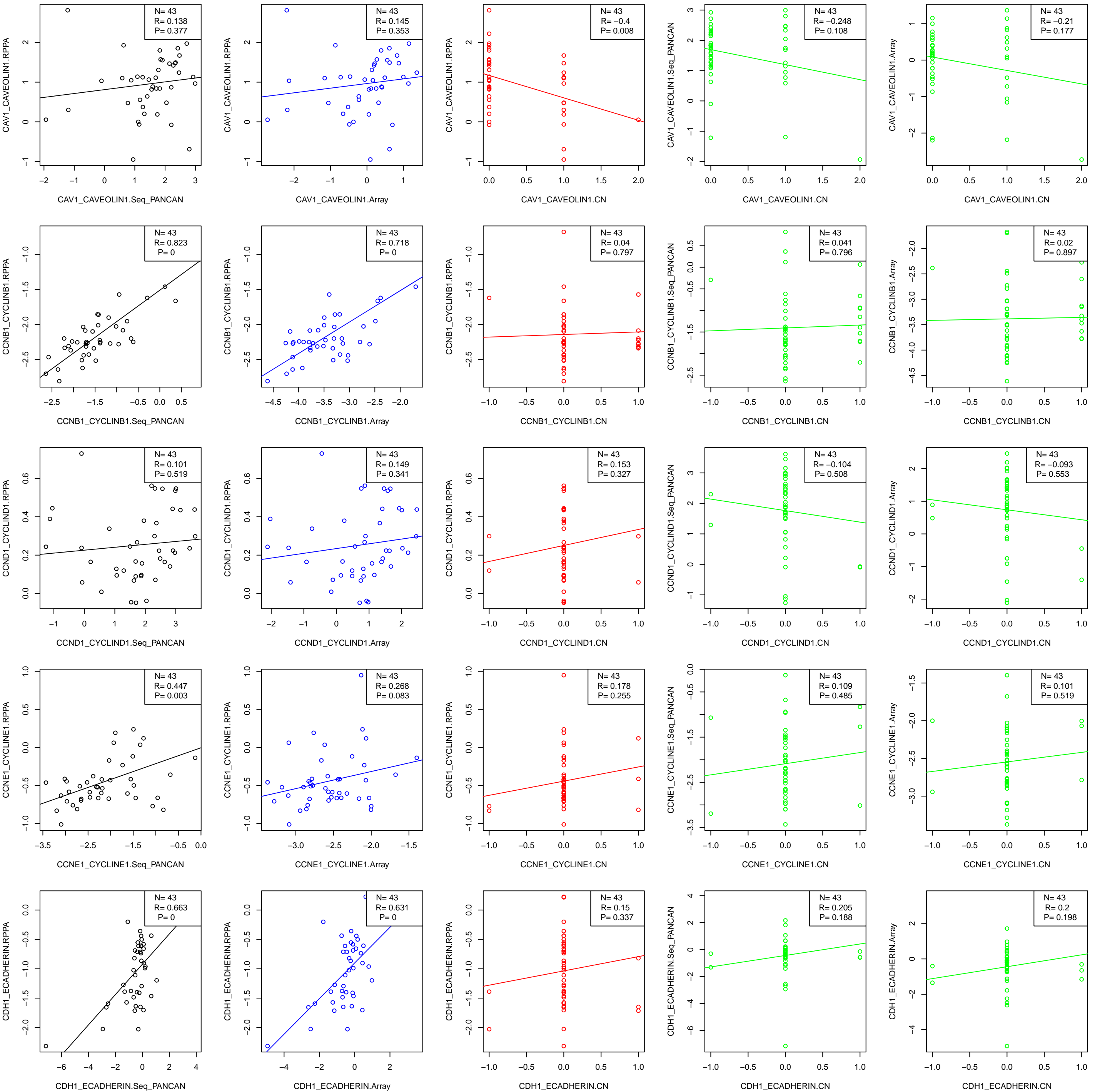

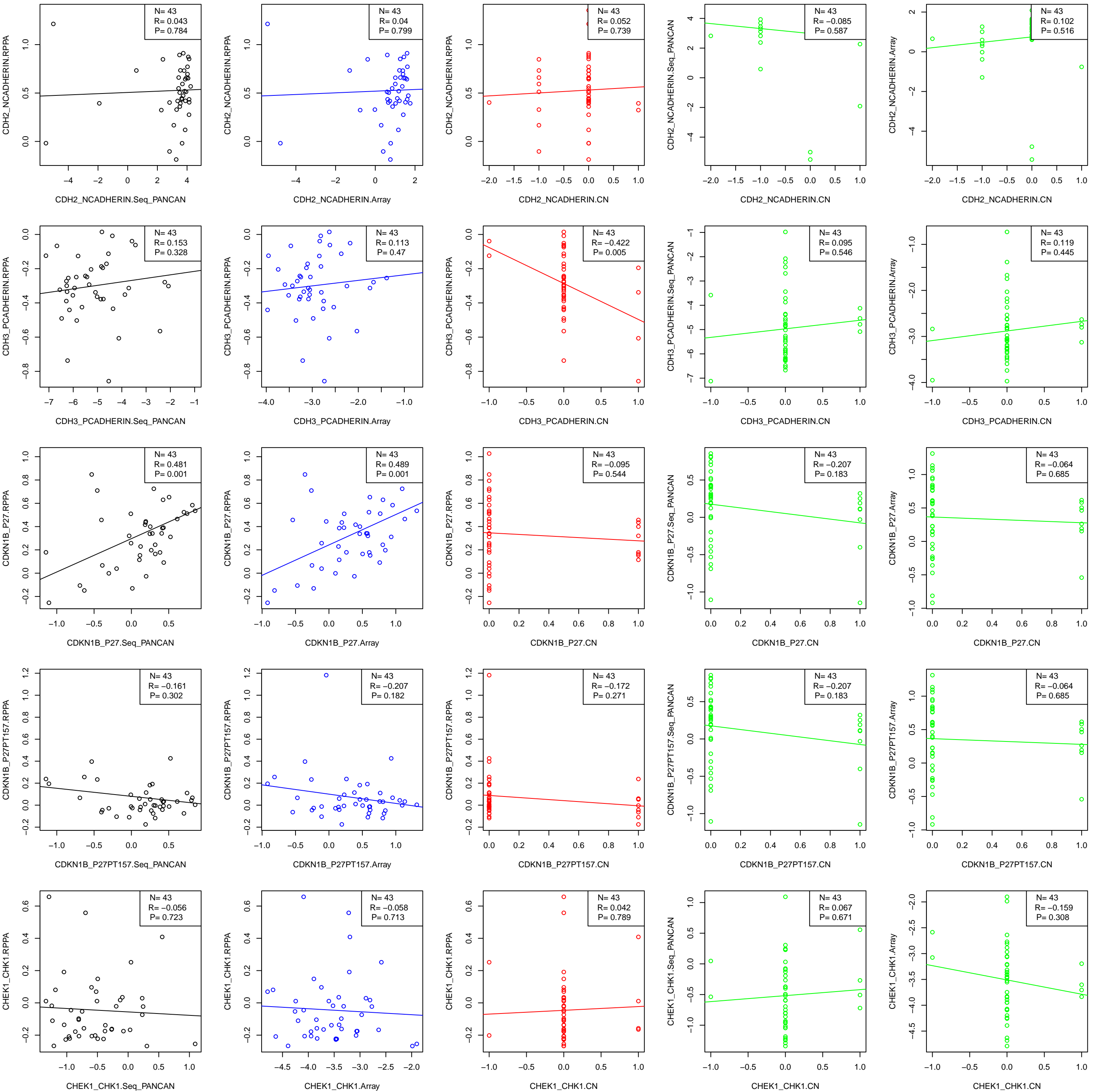

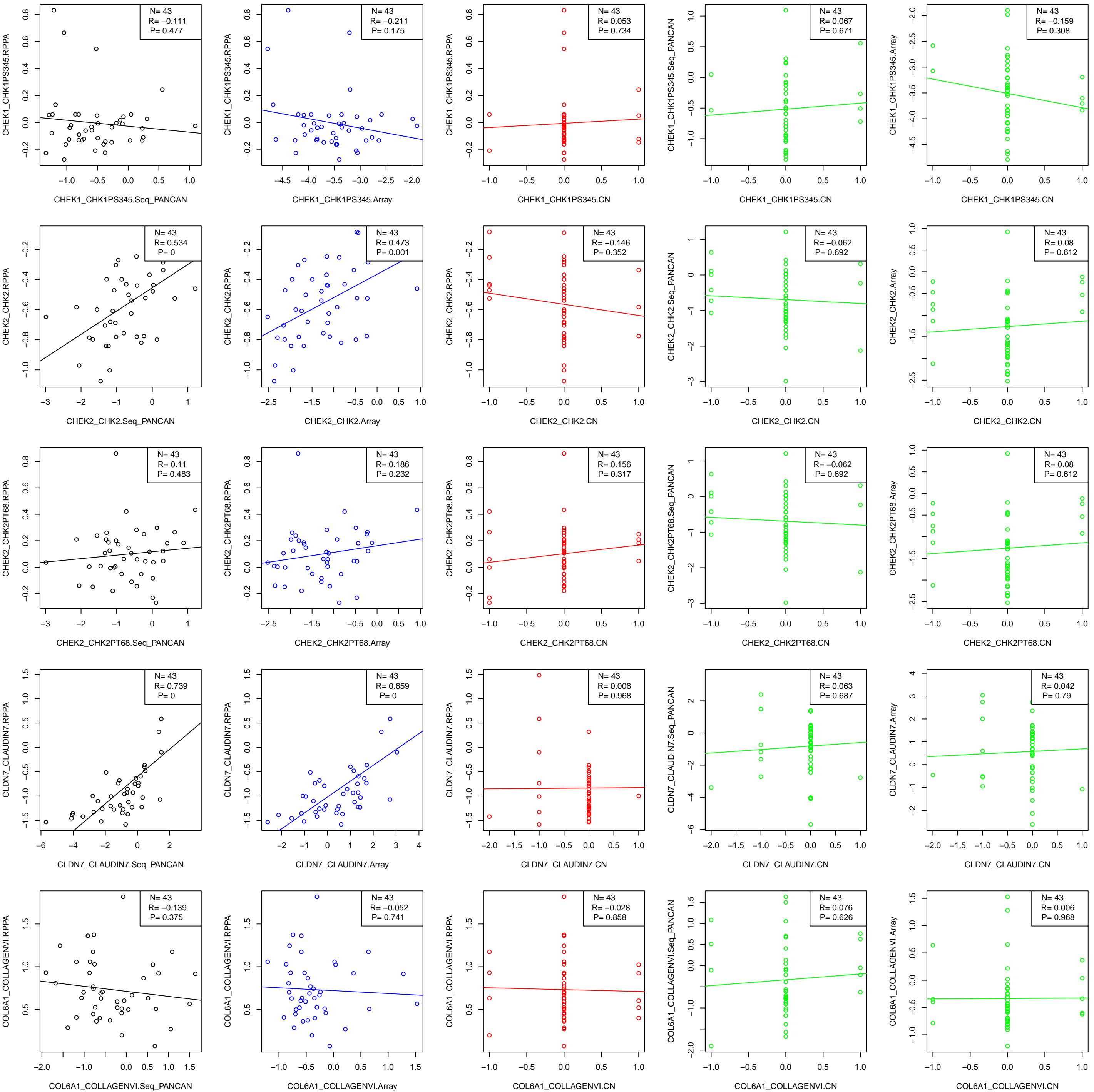

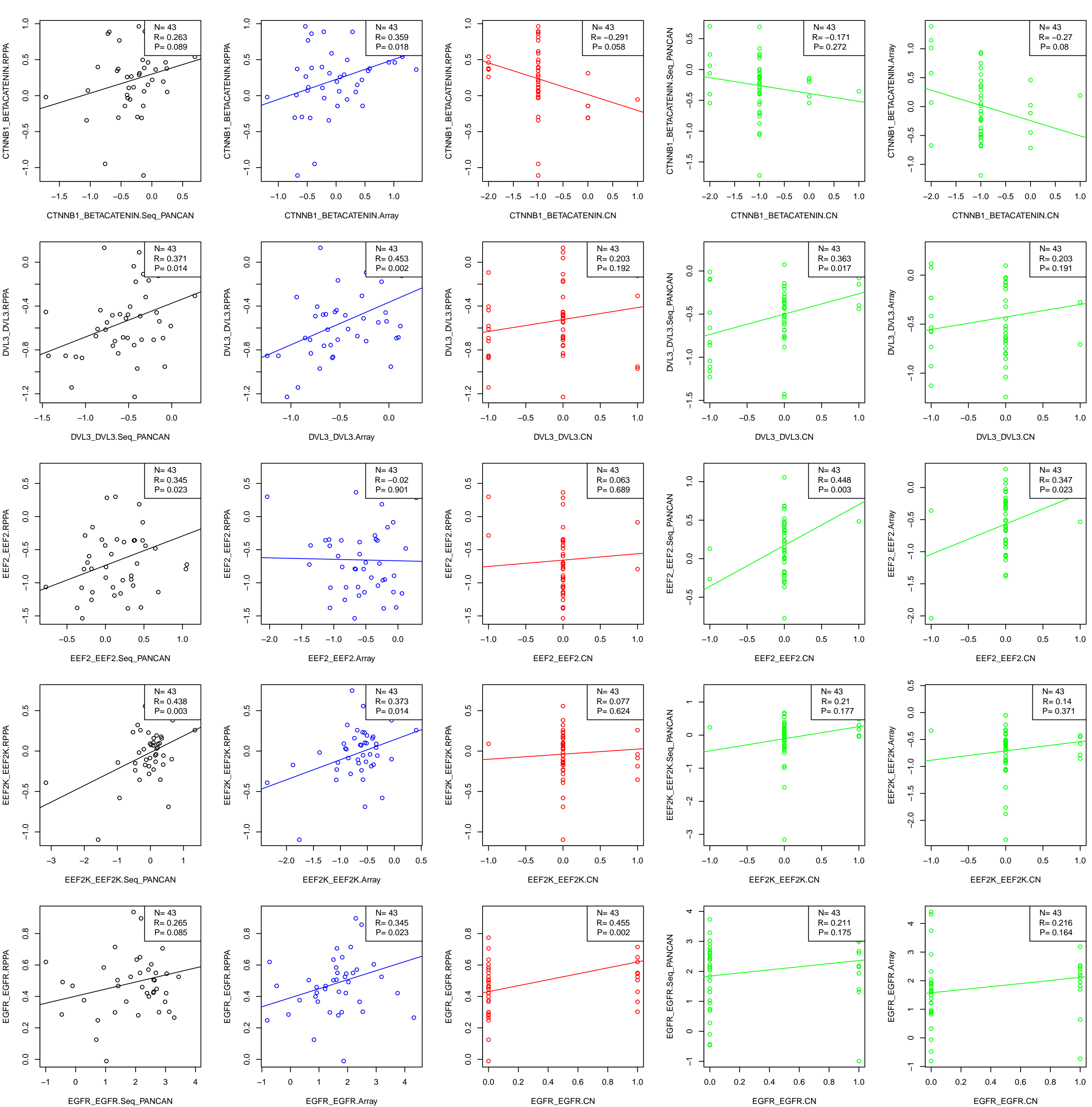

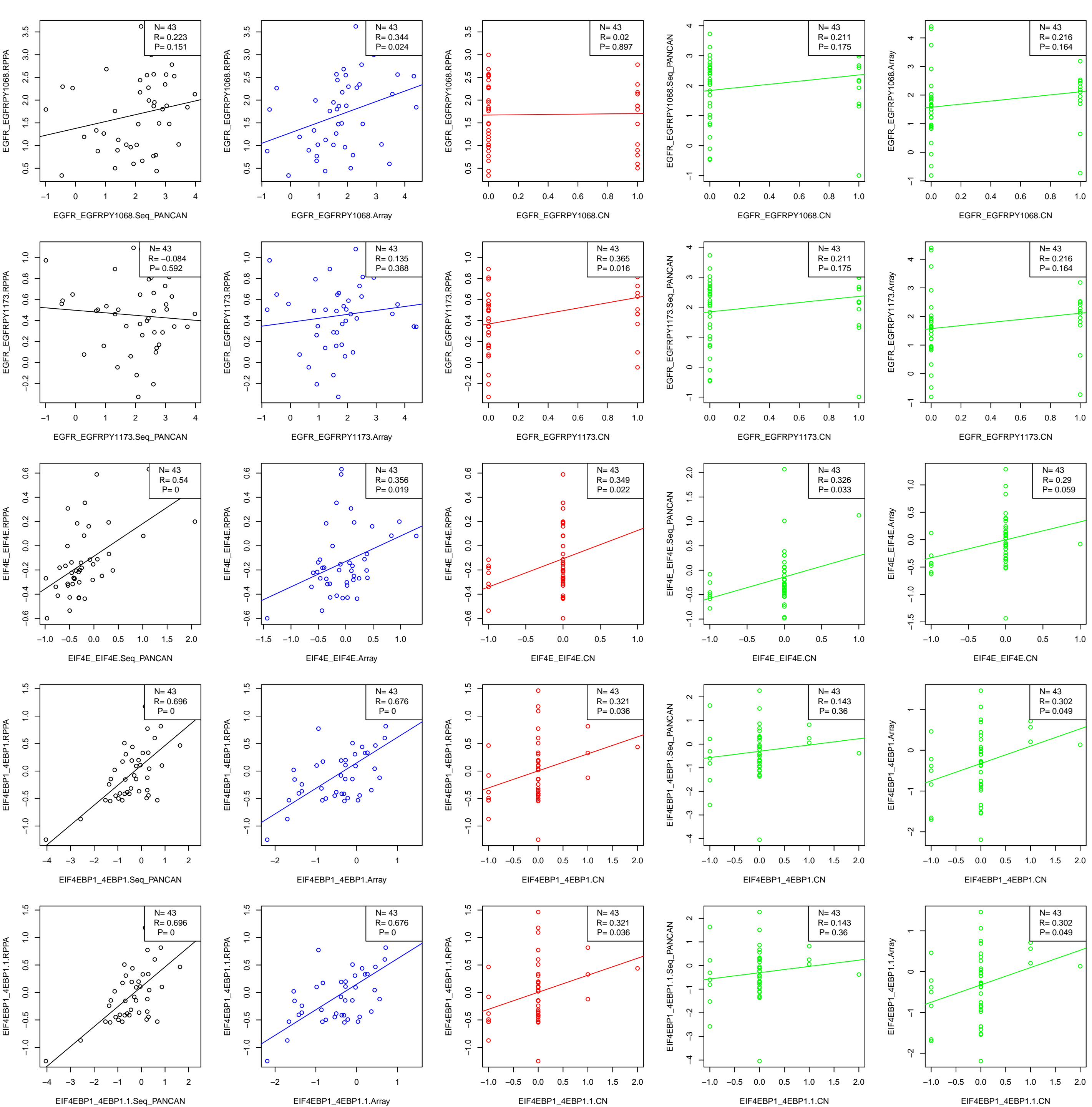

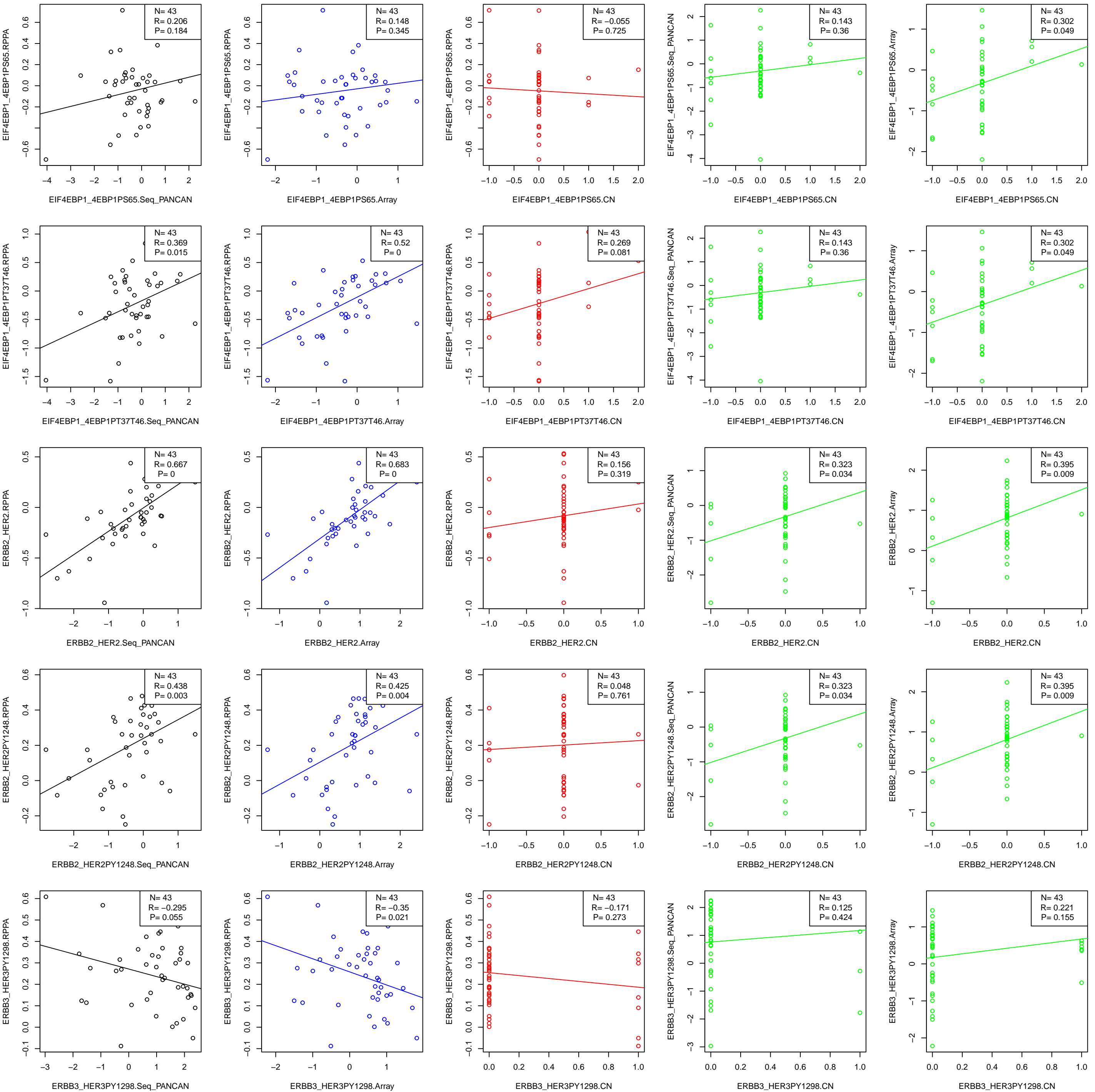

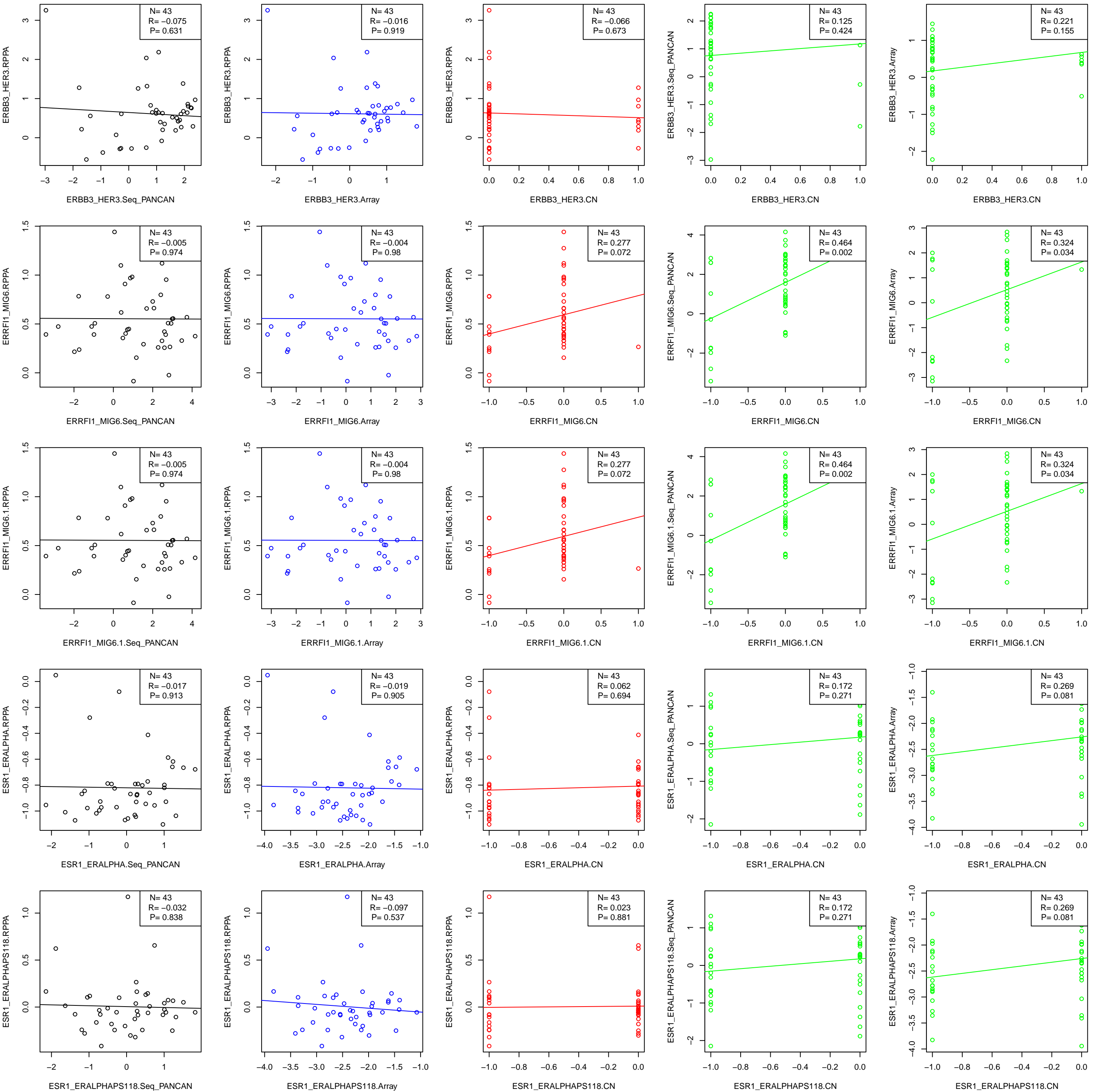

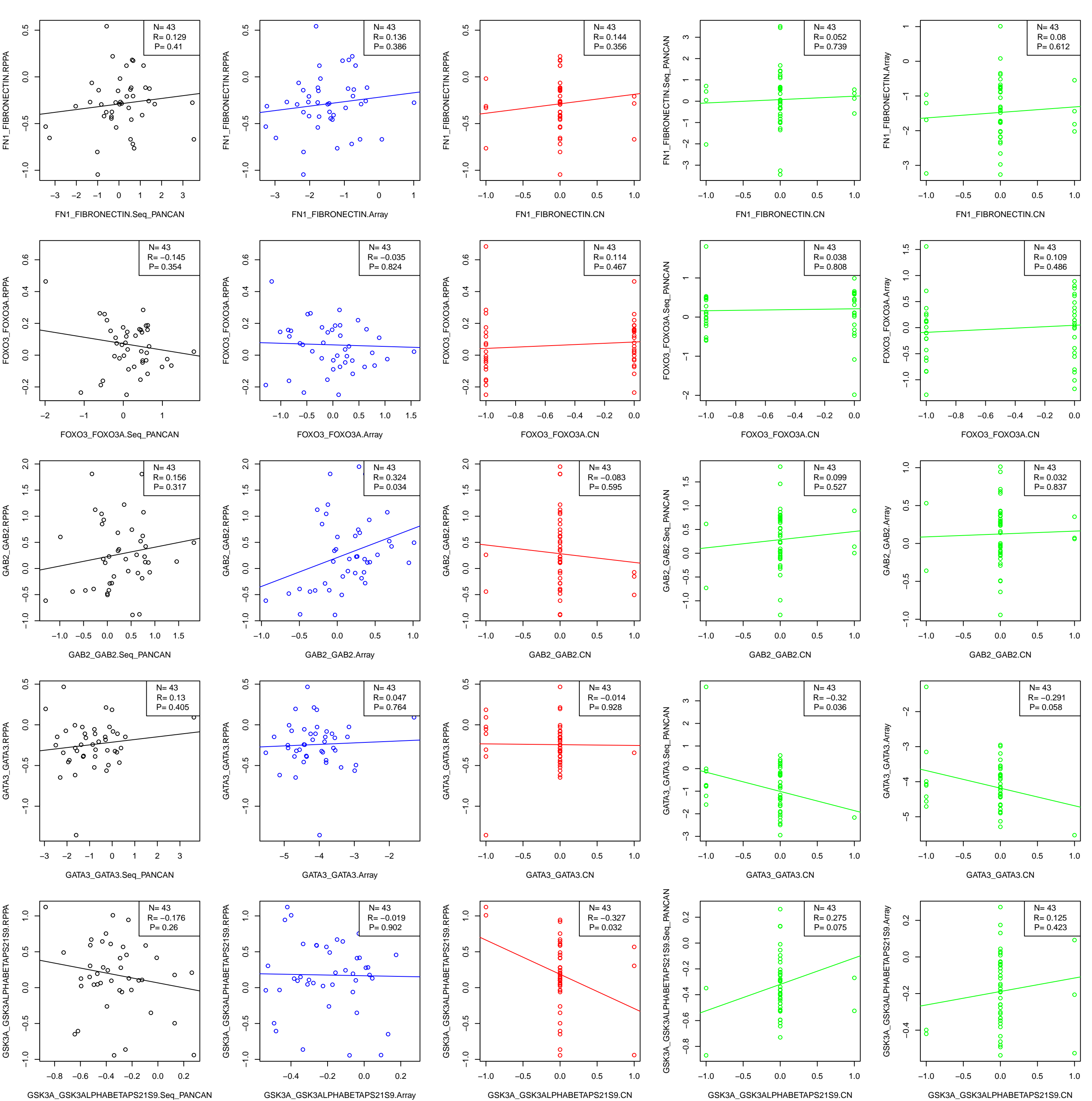

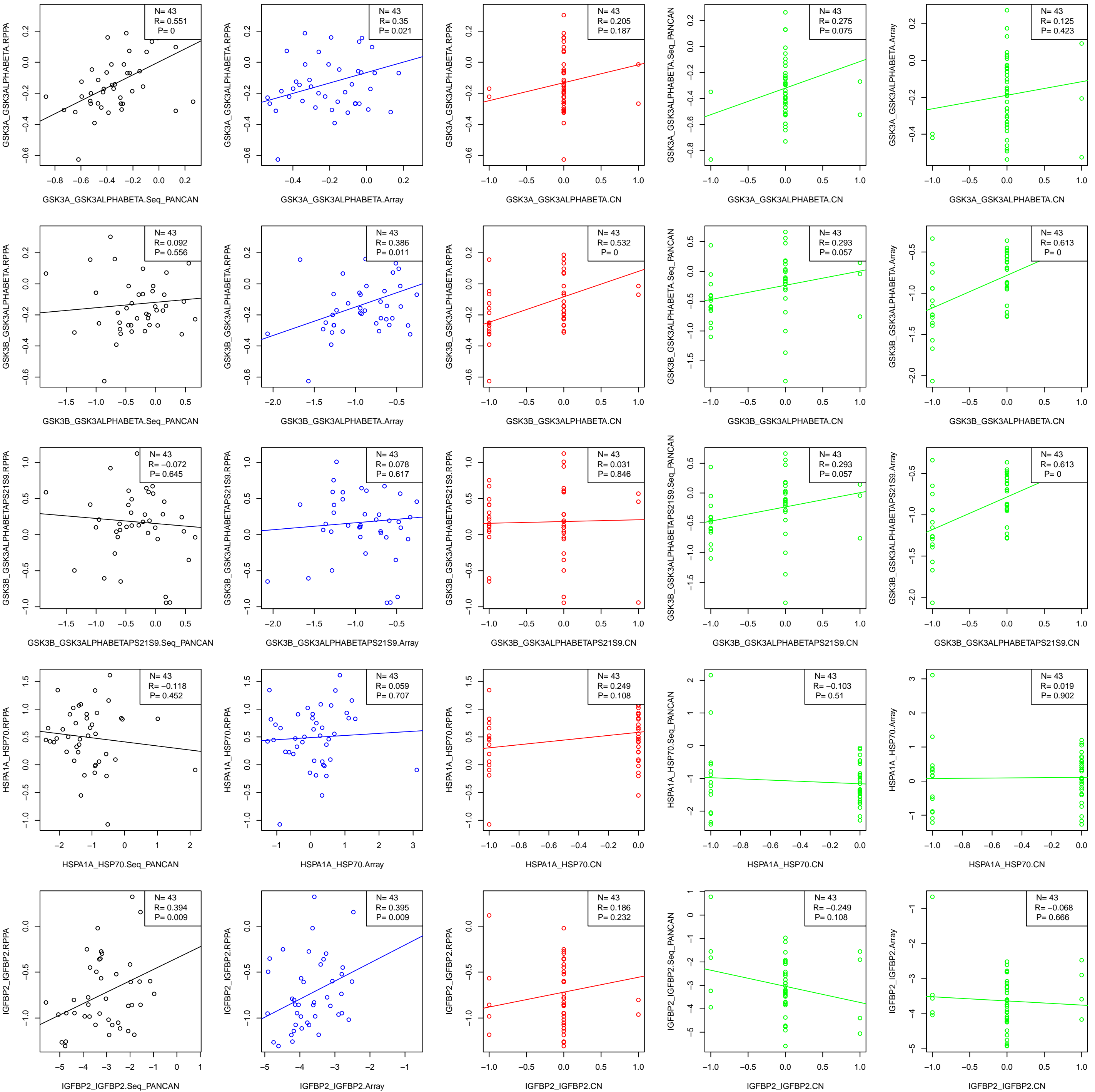

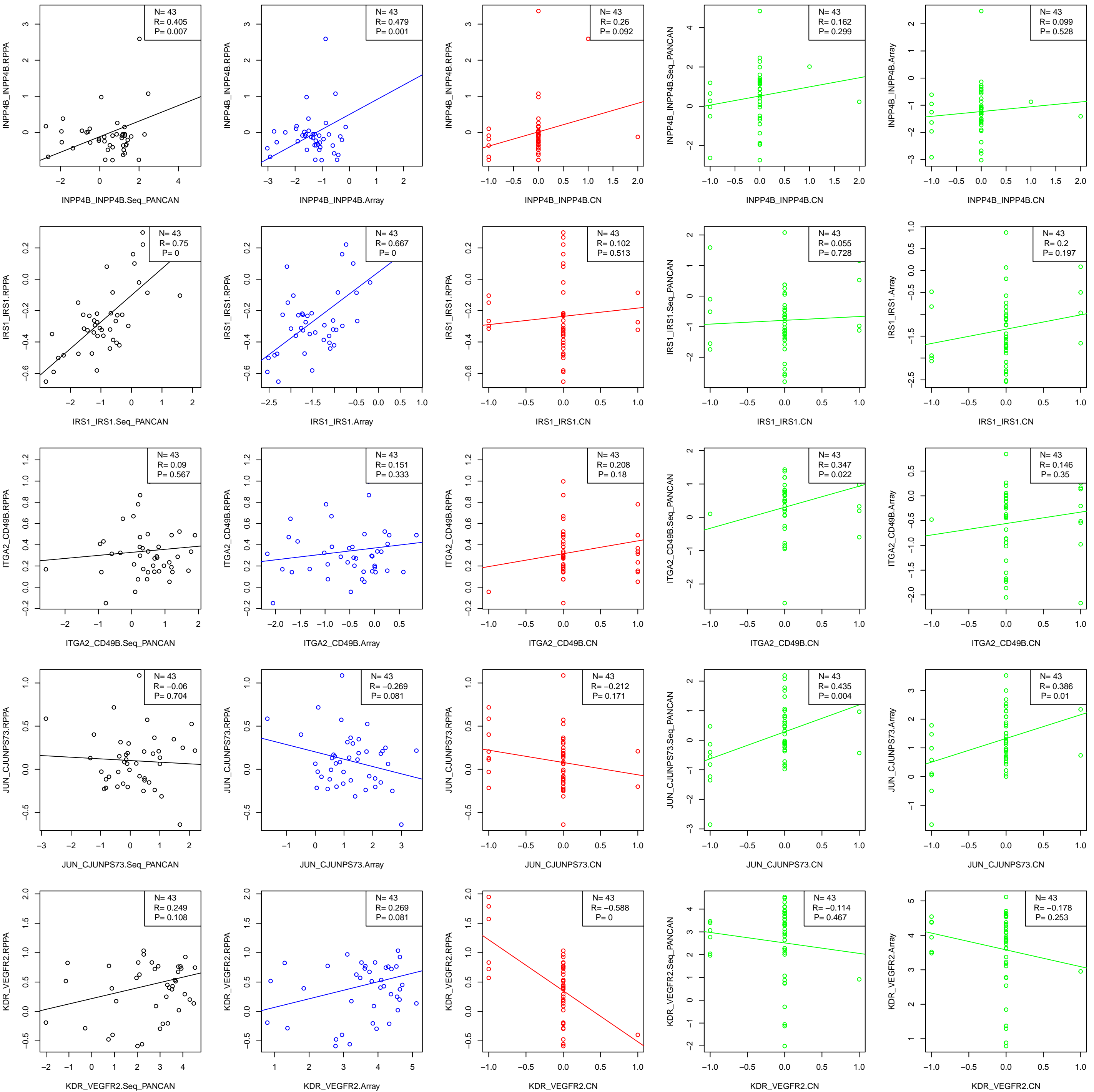

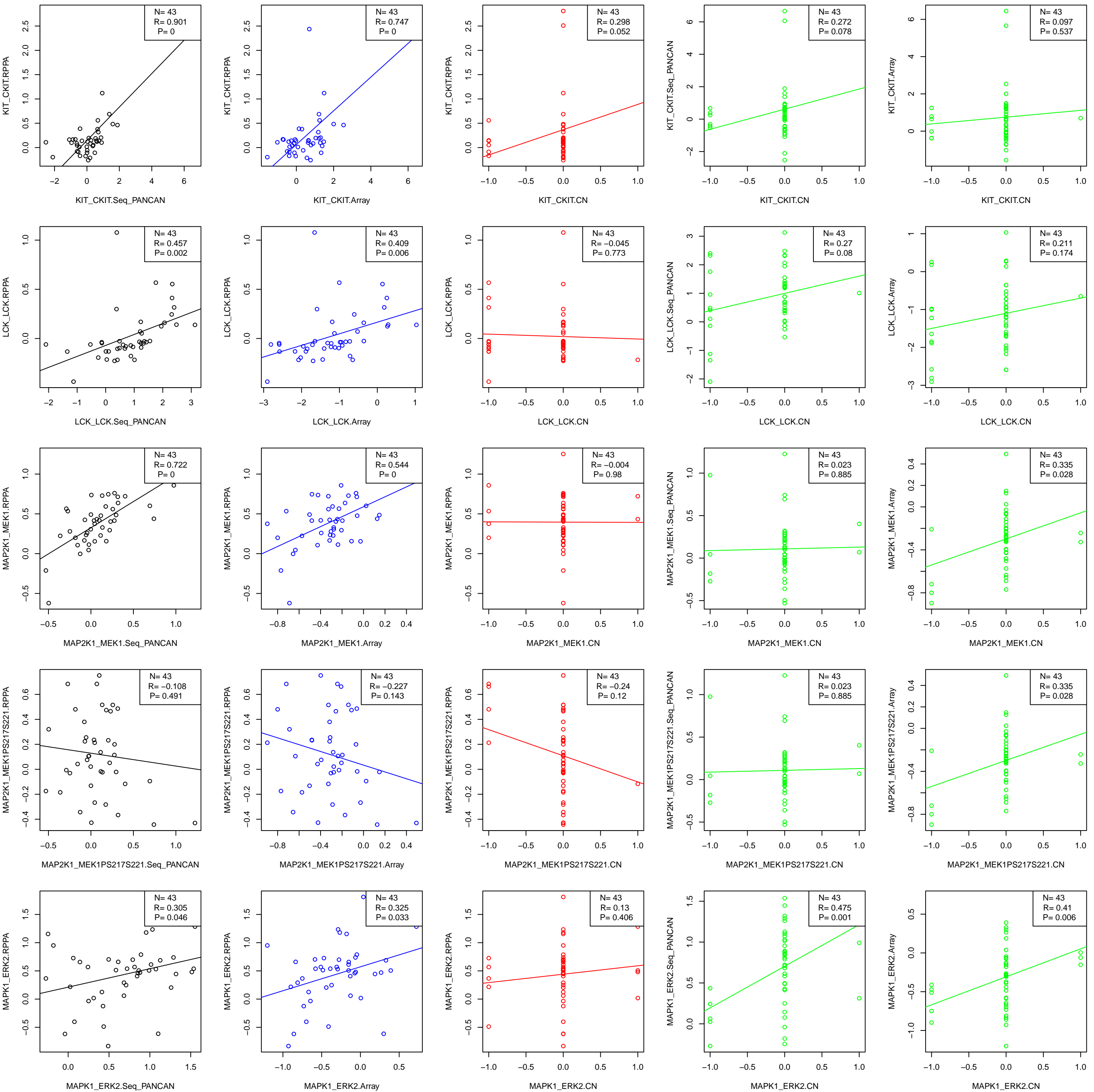

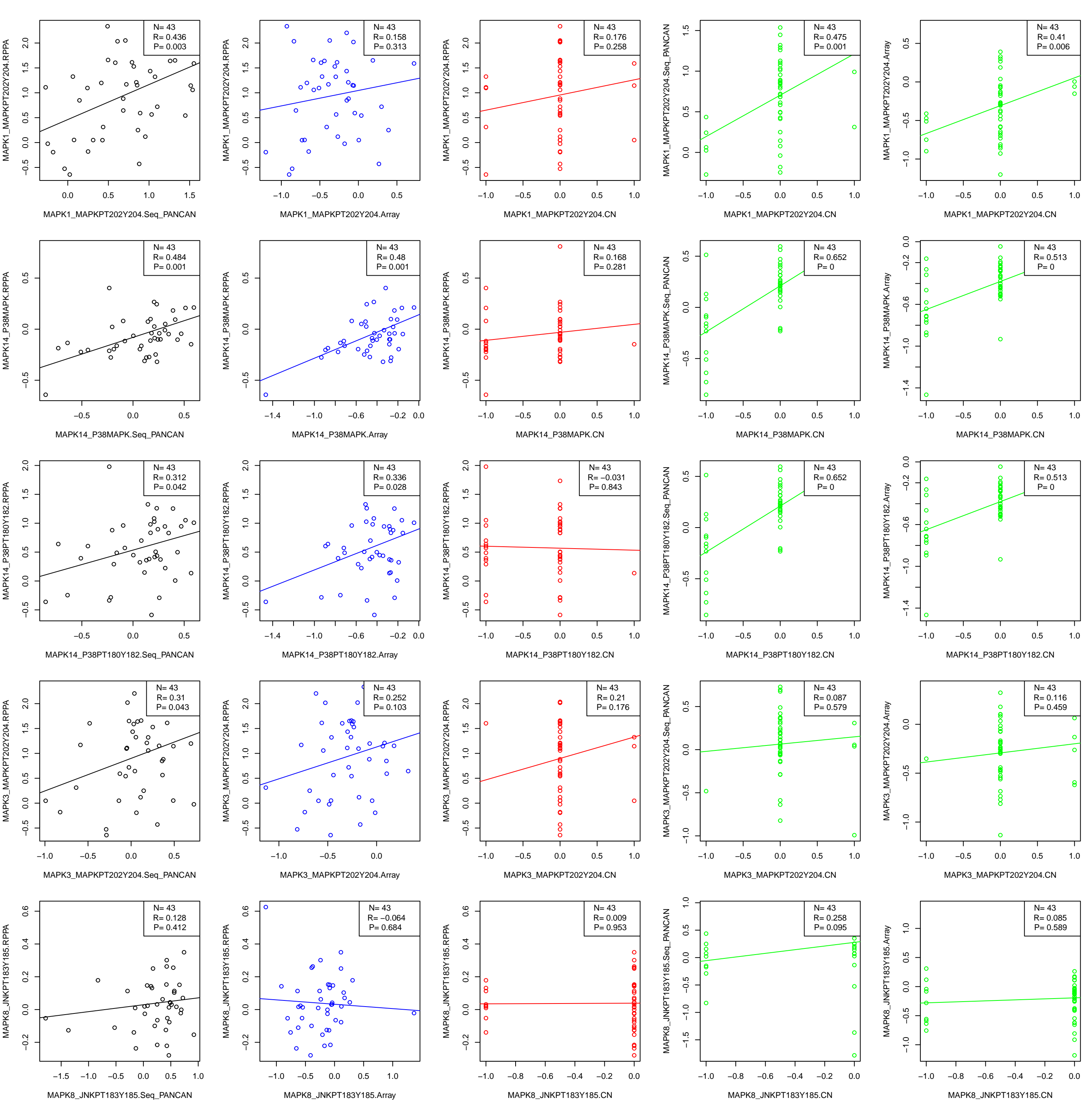

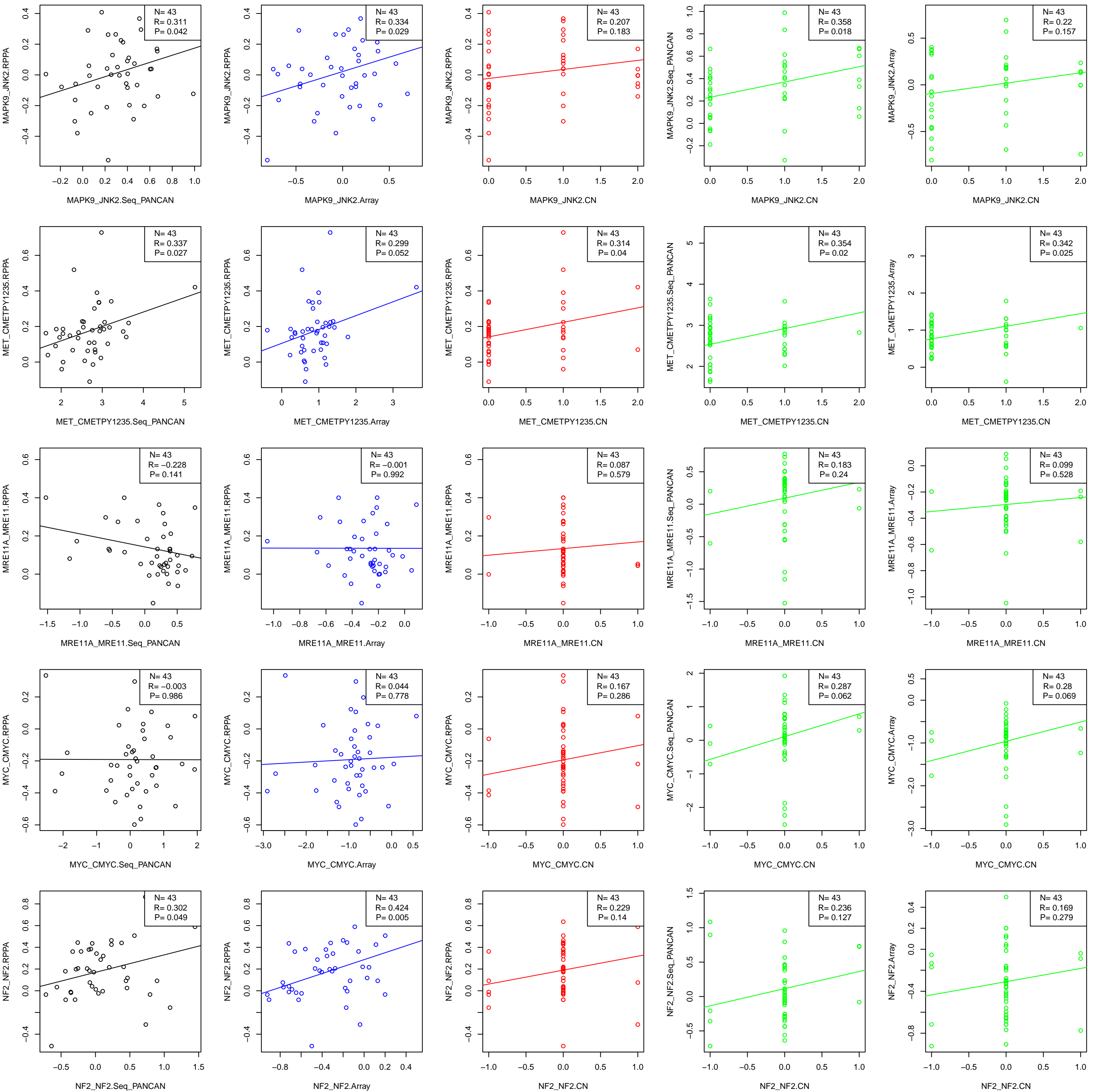

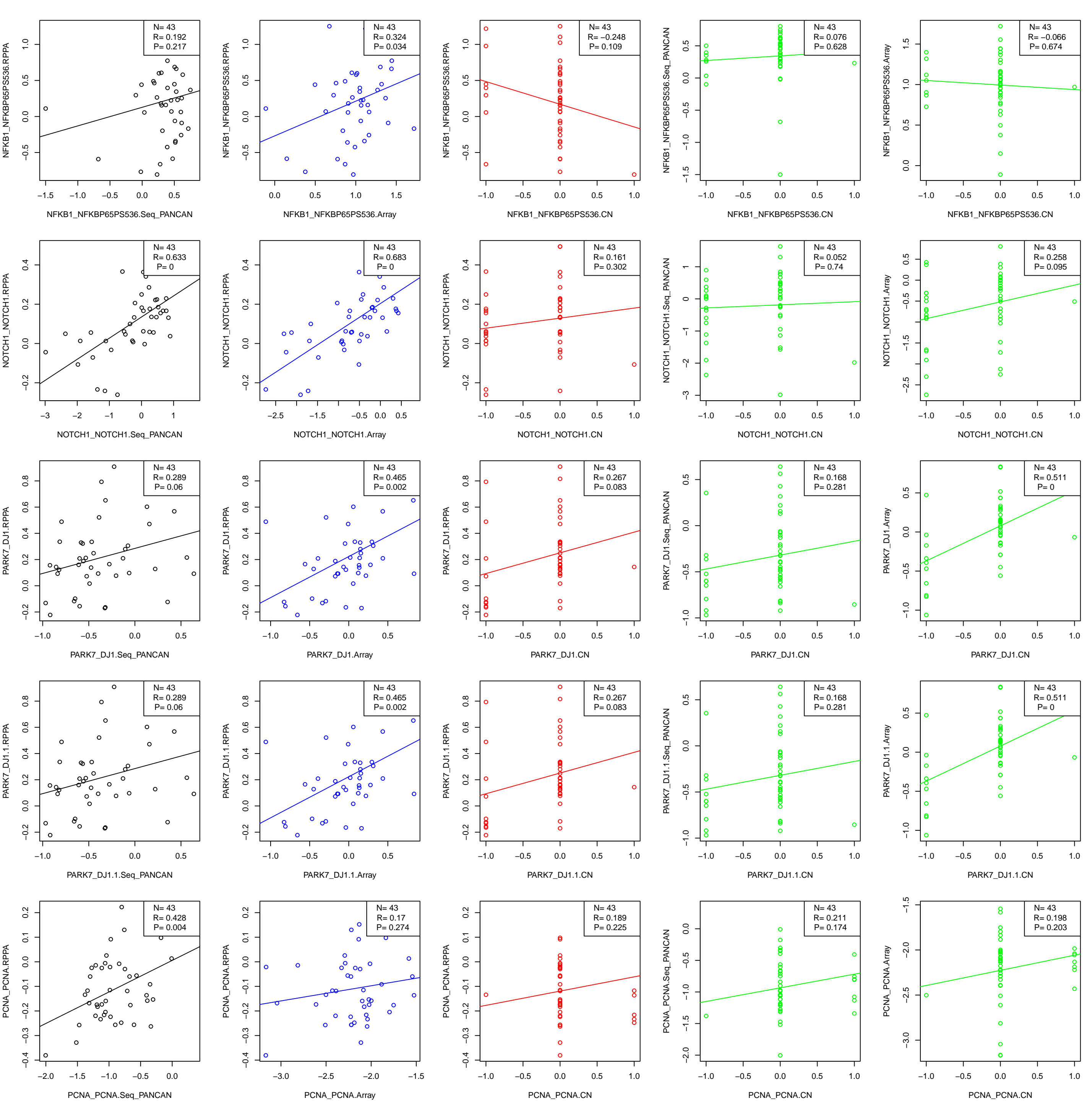

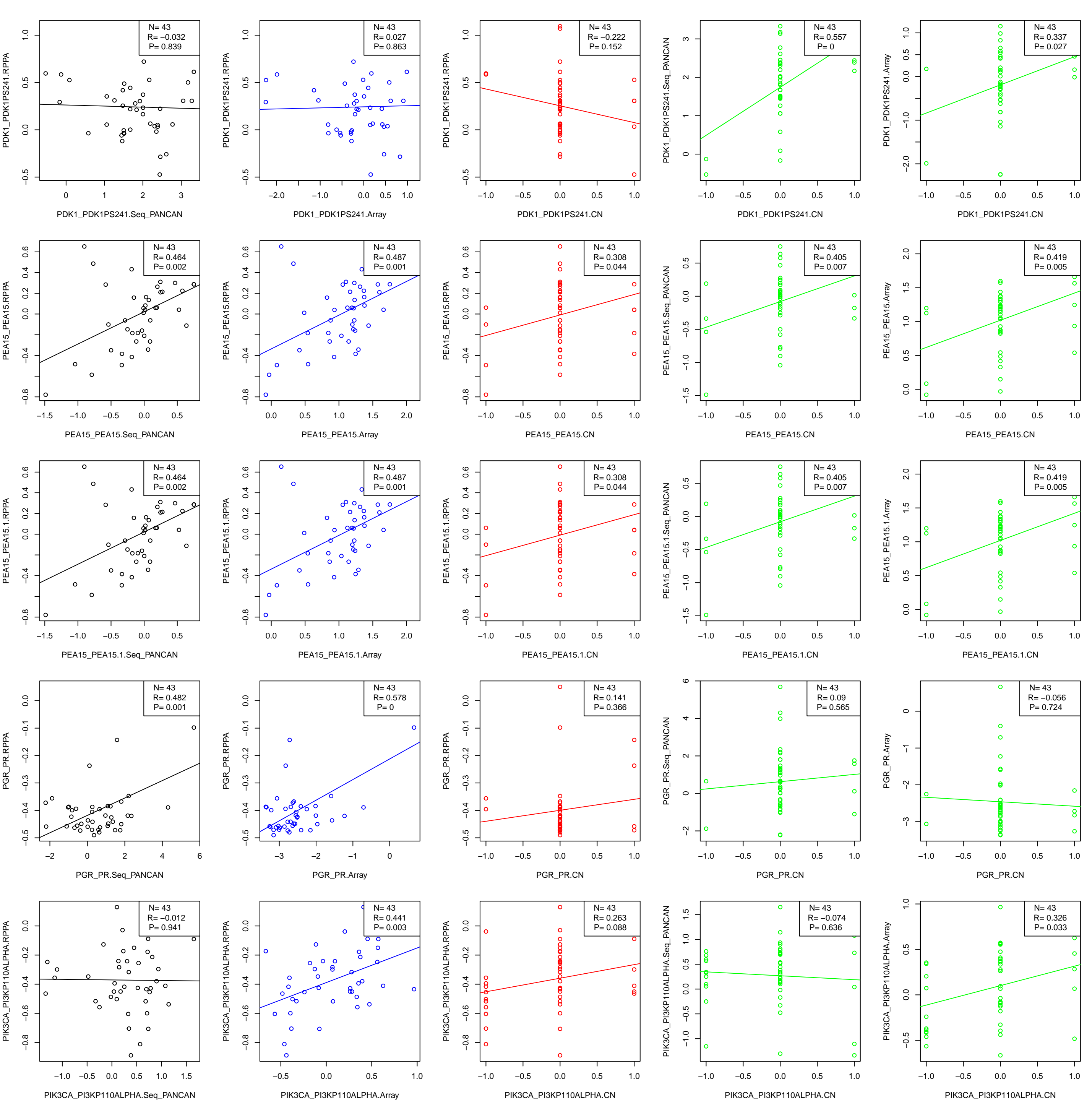

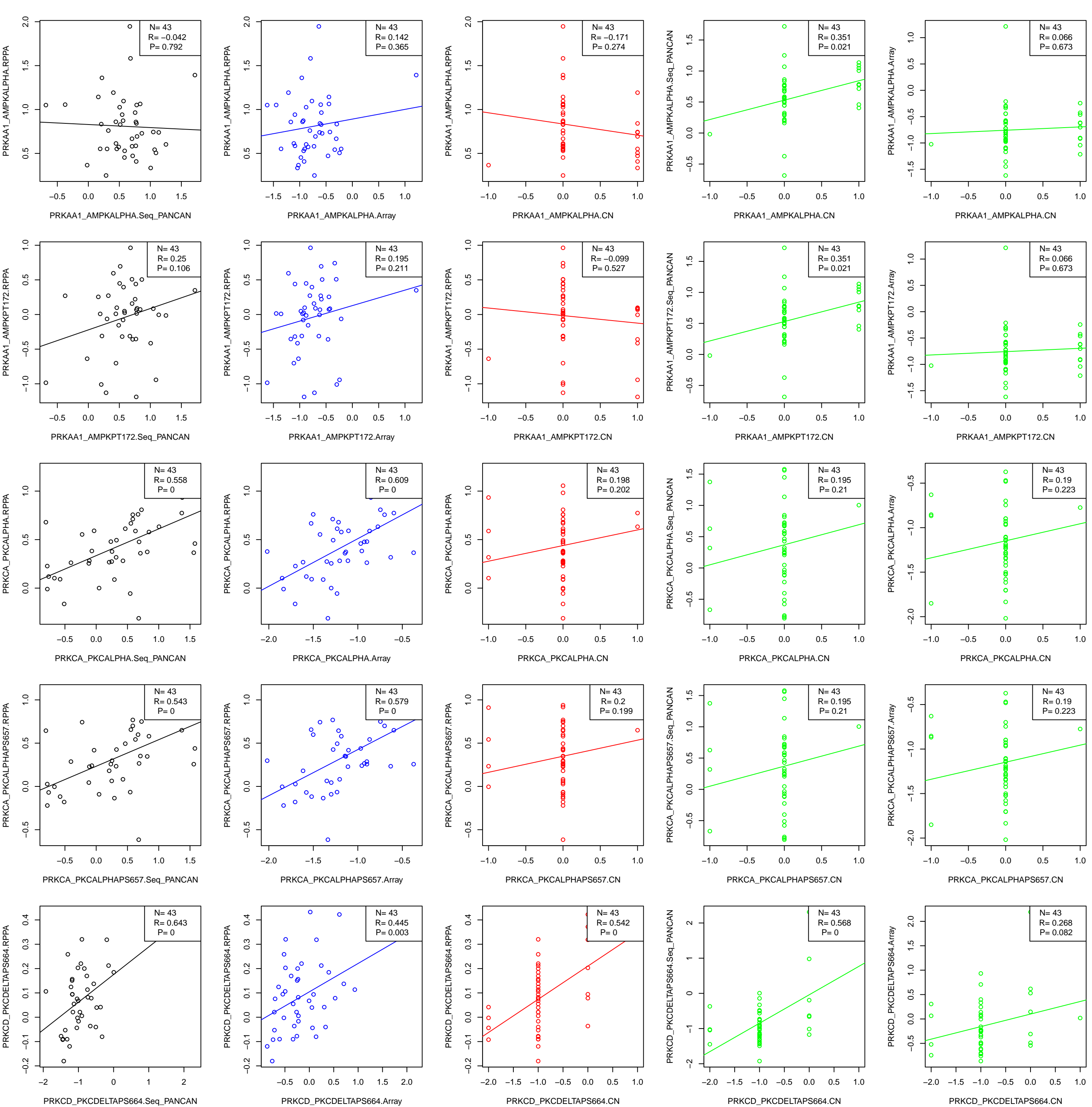

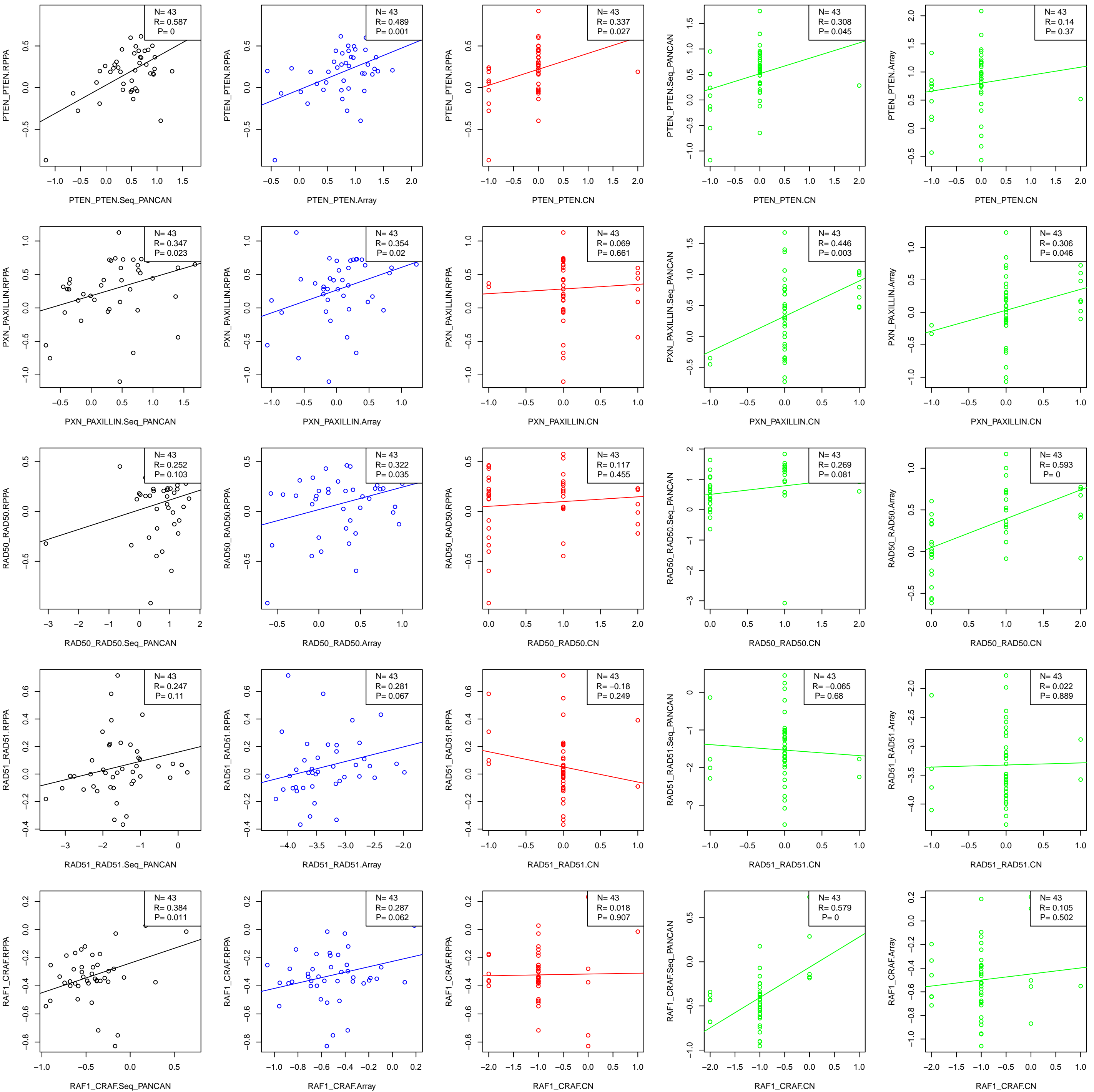

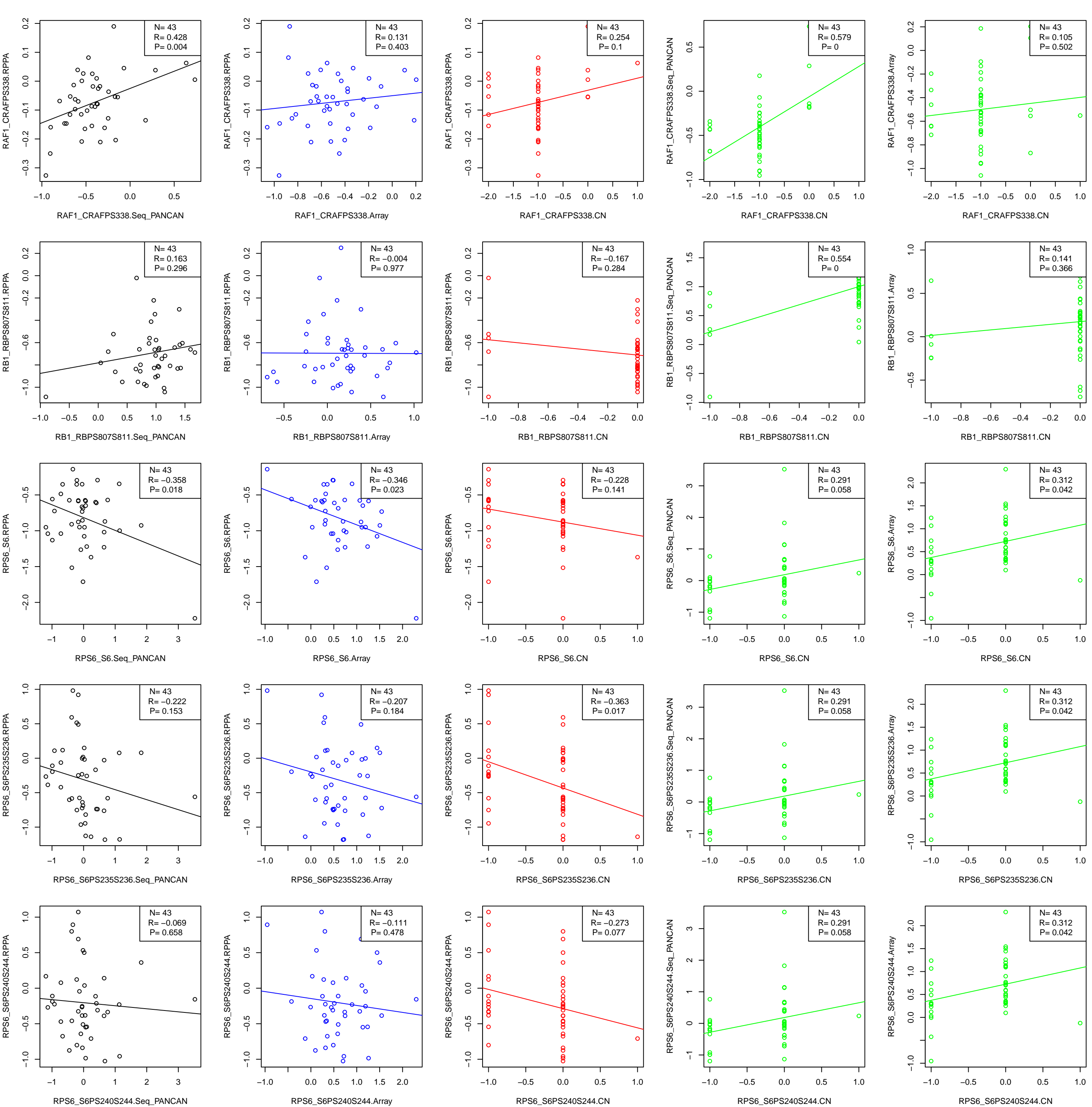

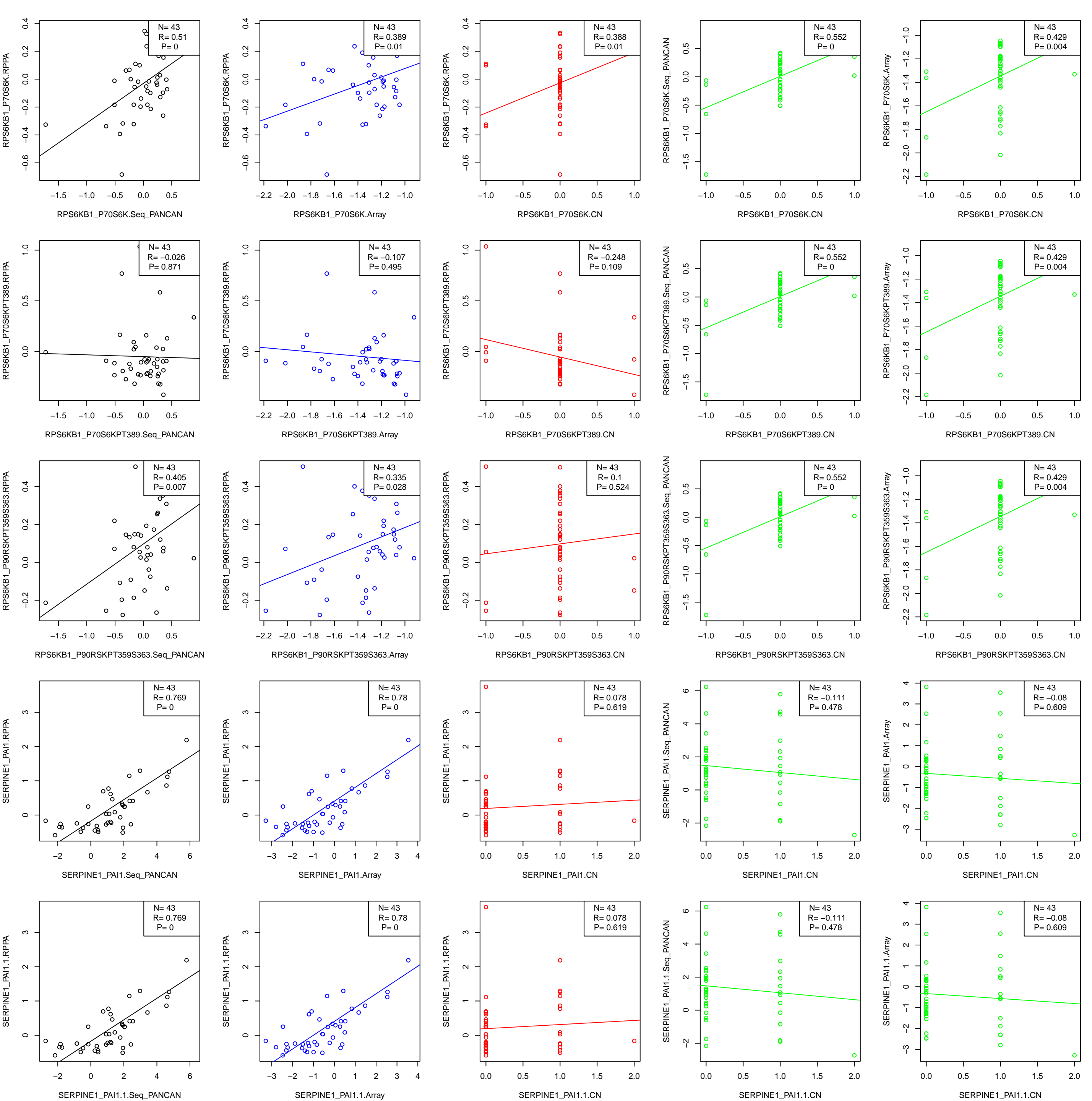

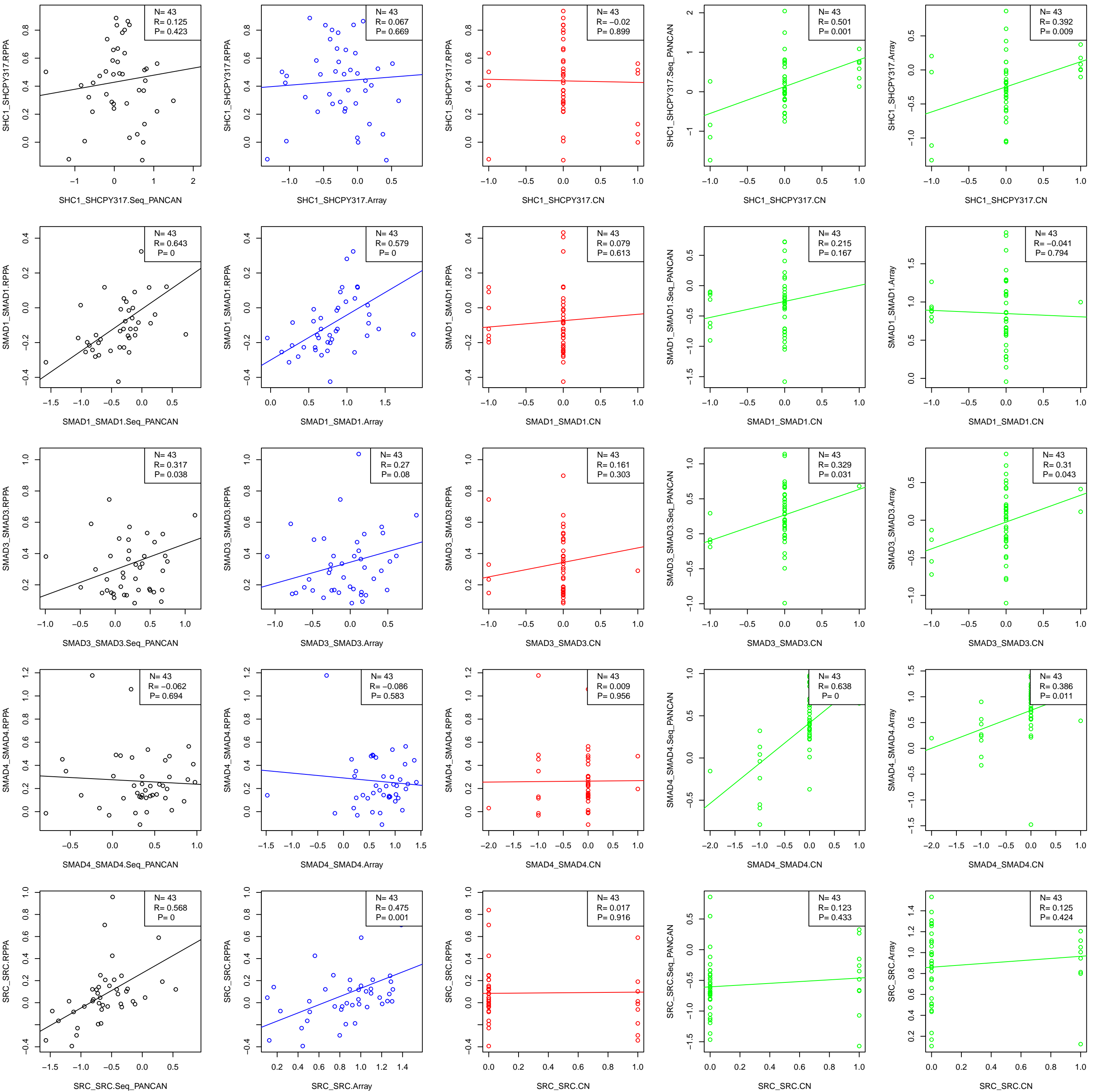

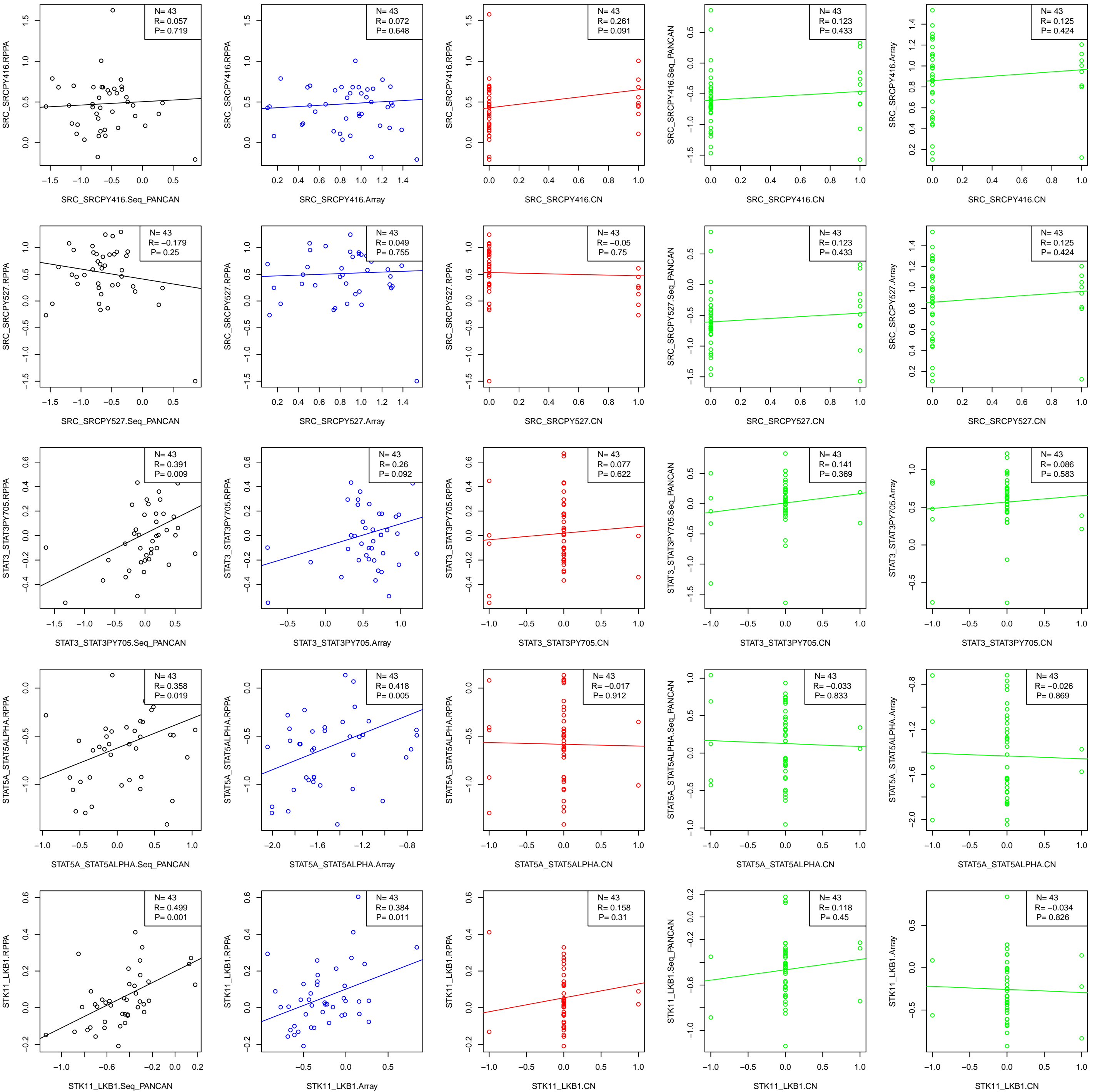

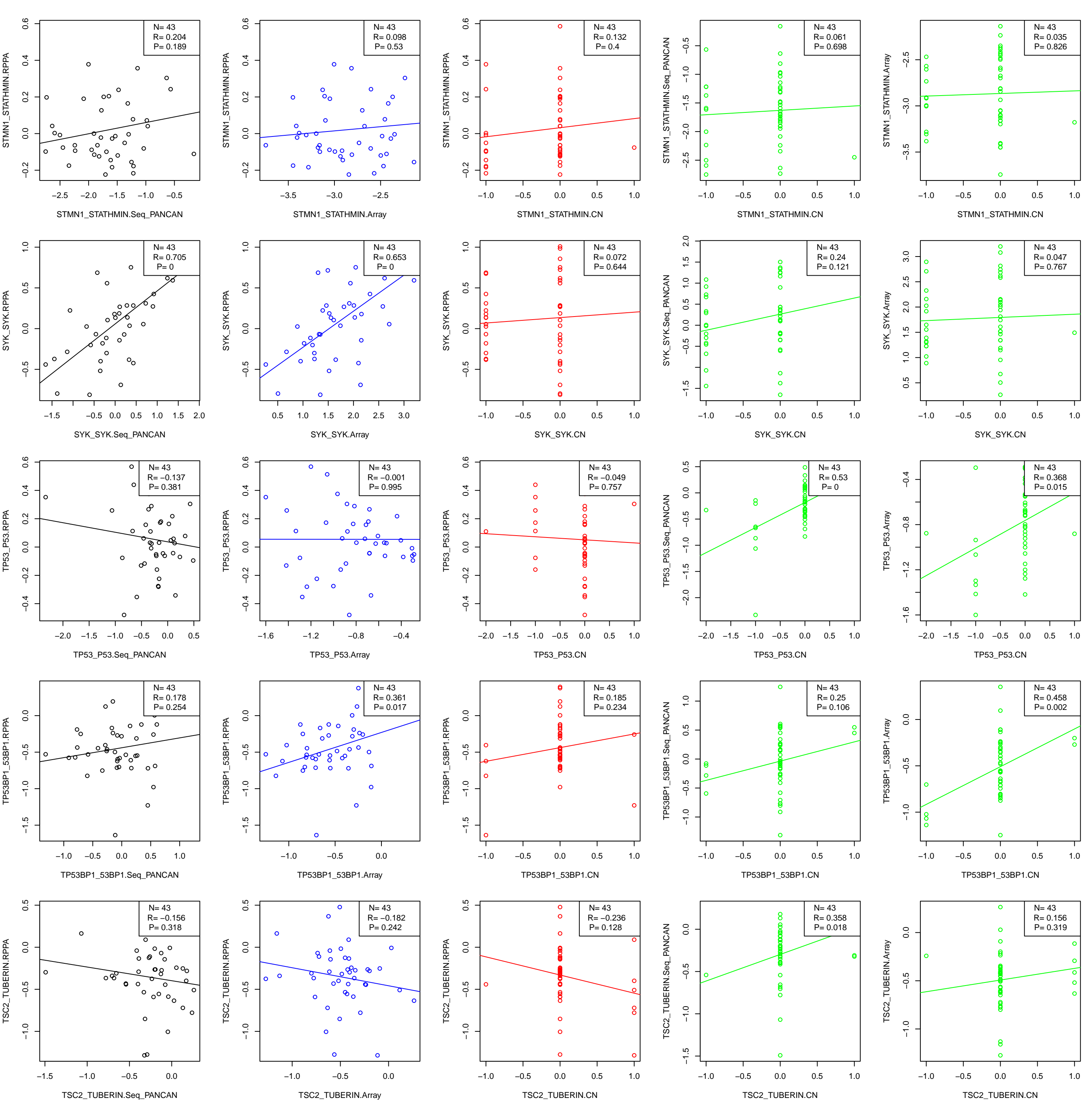

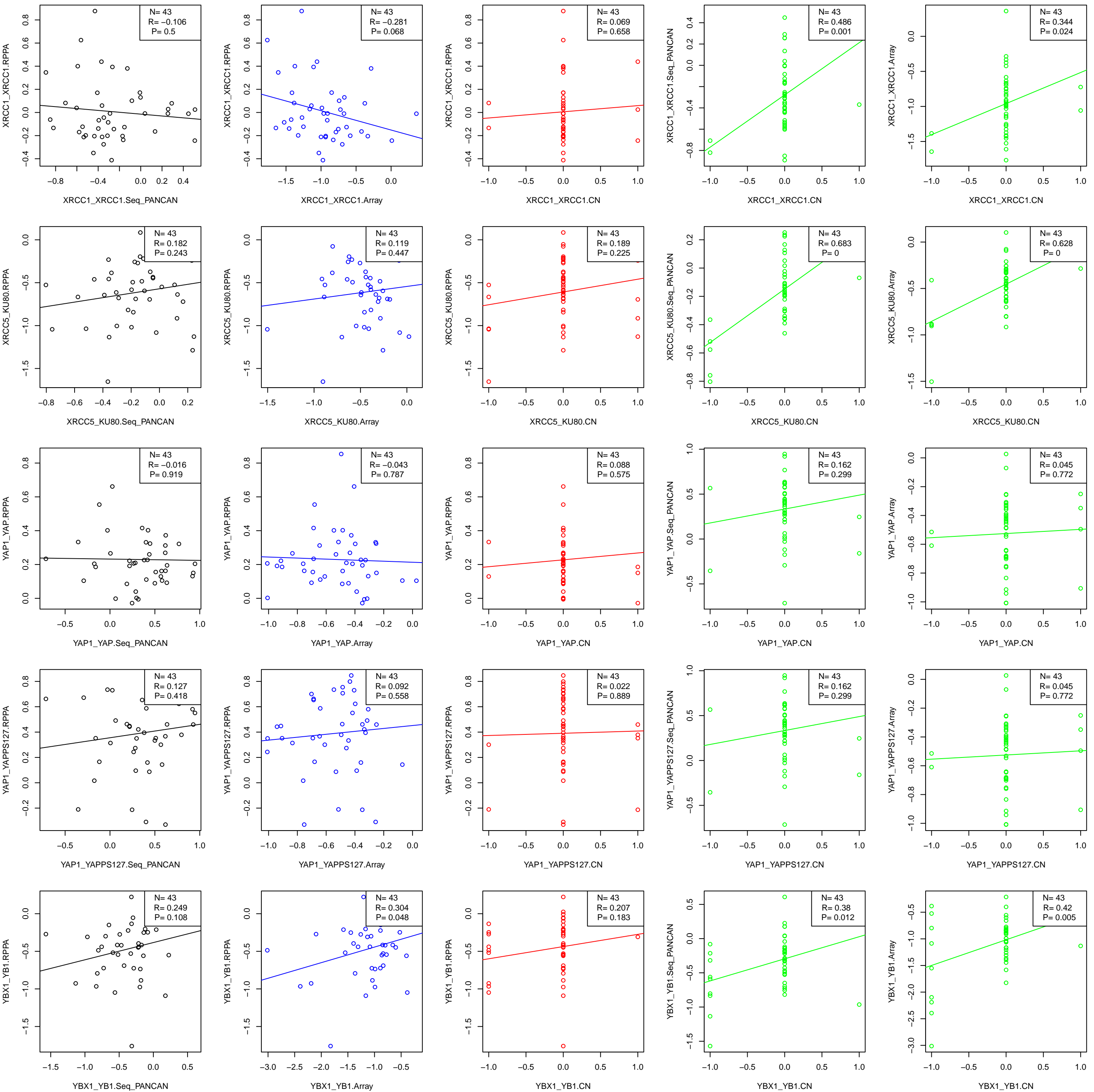

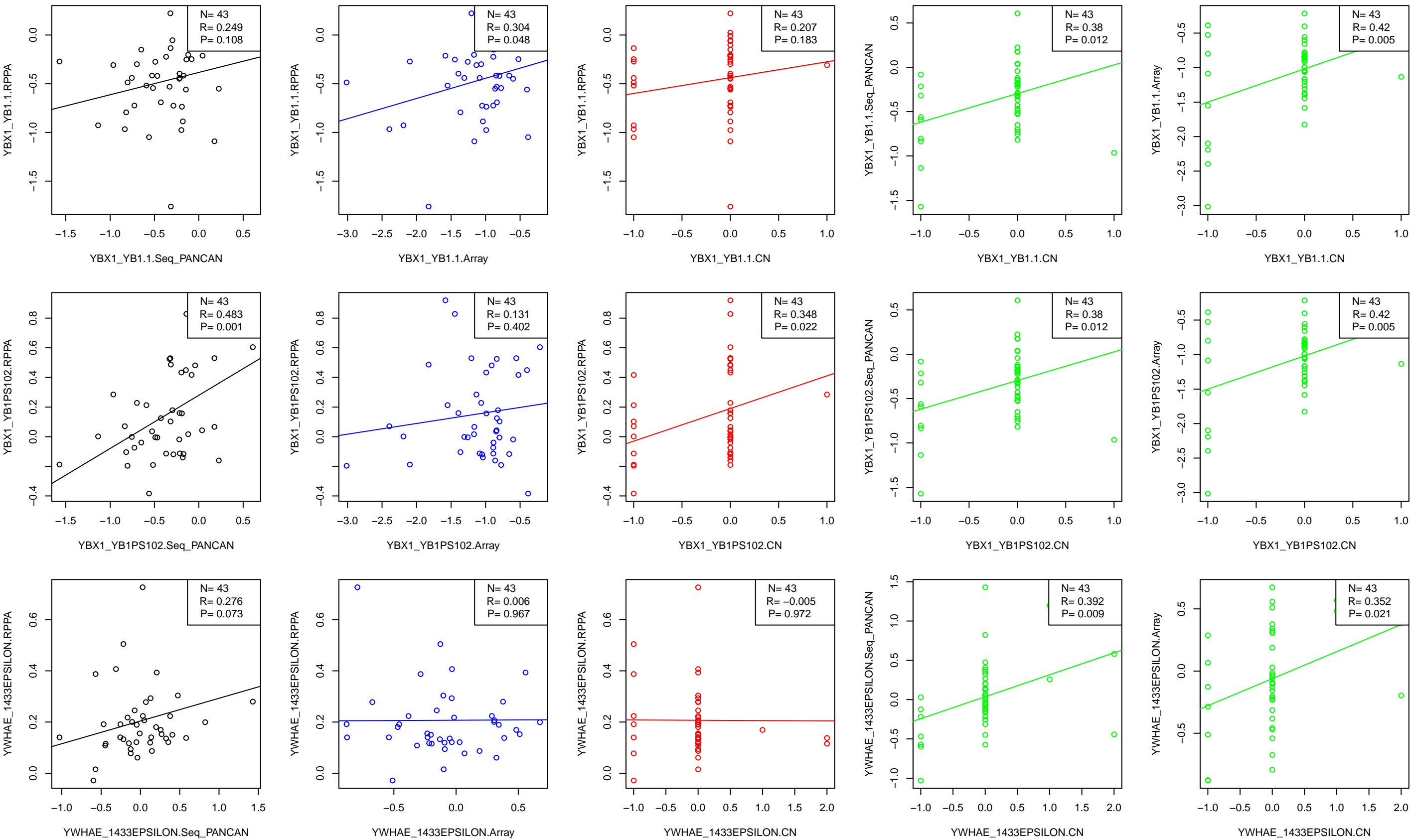

Supplement: Supplementary file 10 [file DataSheet3.PDF]

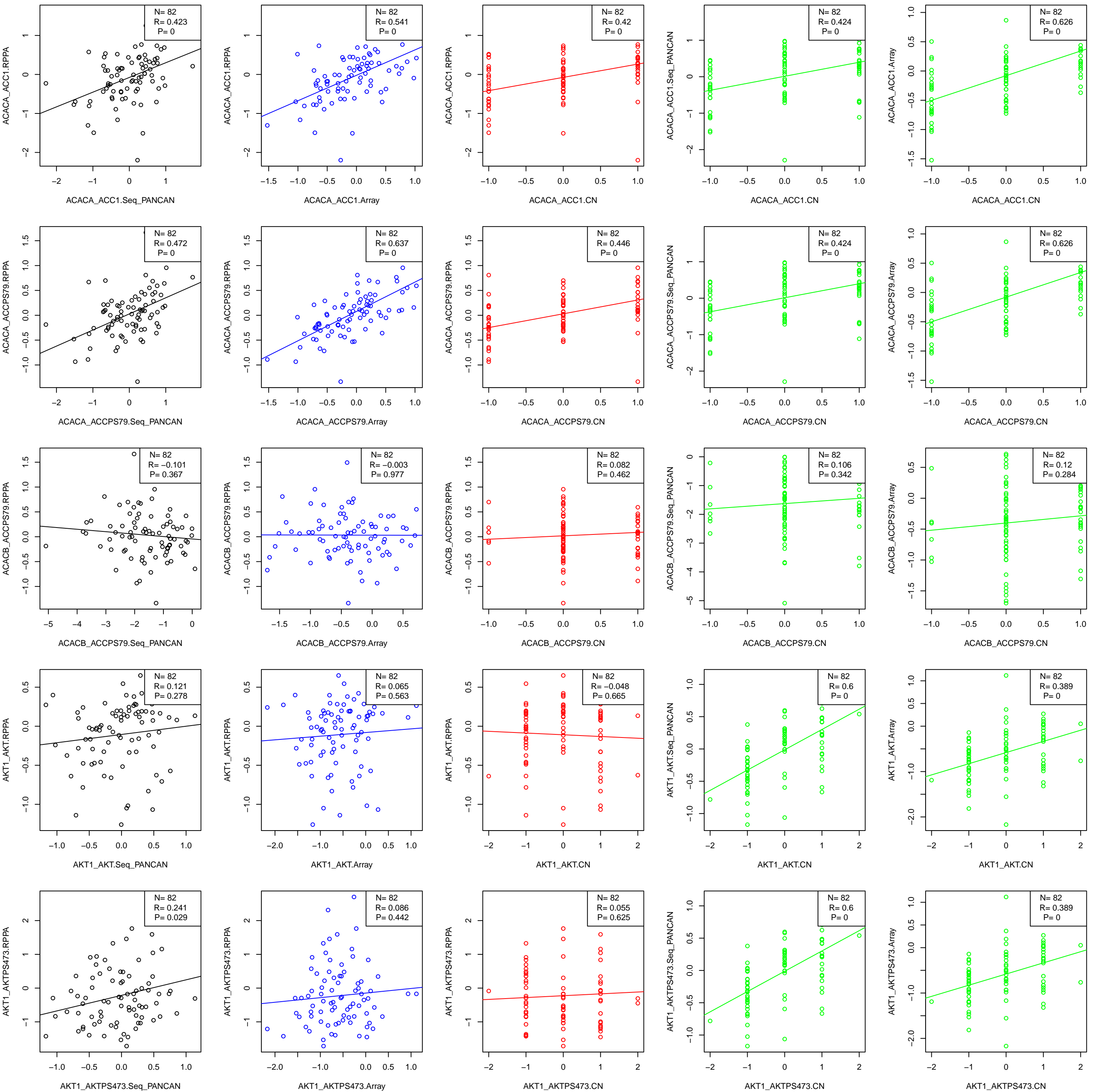

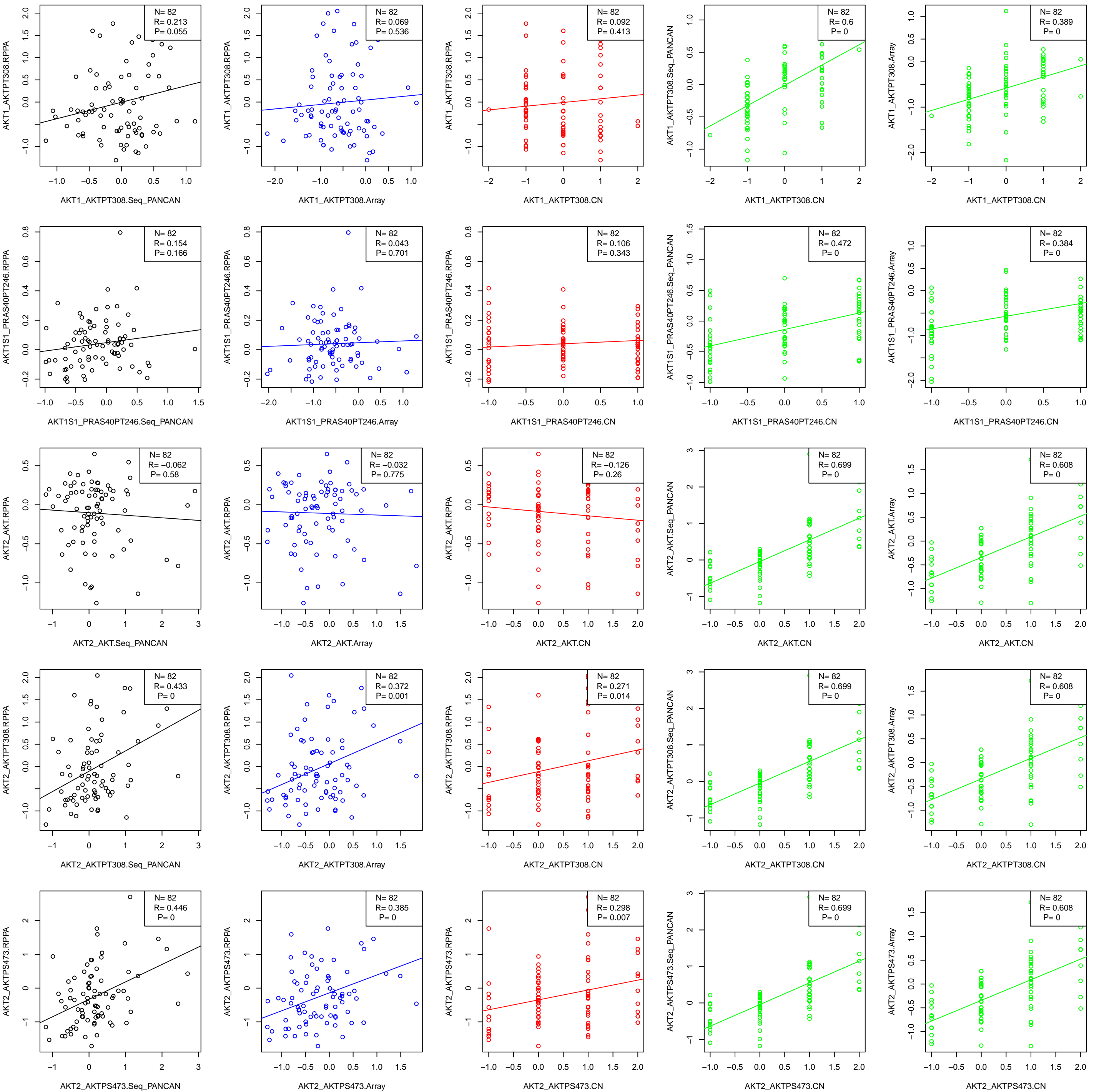

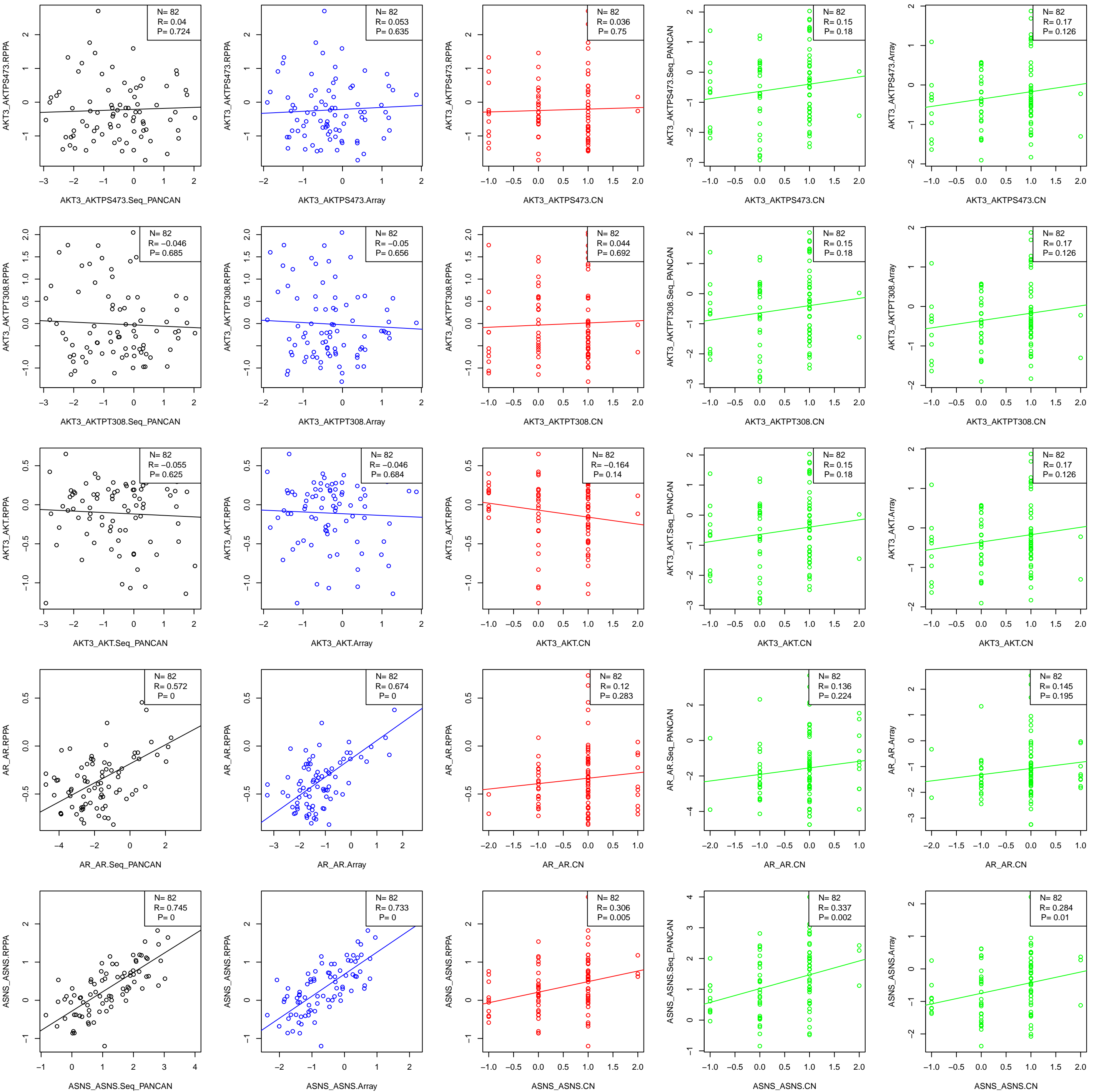

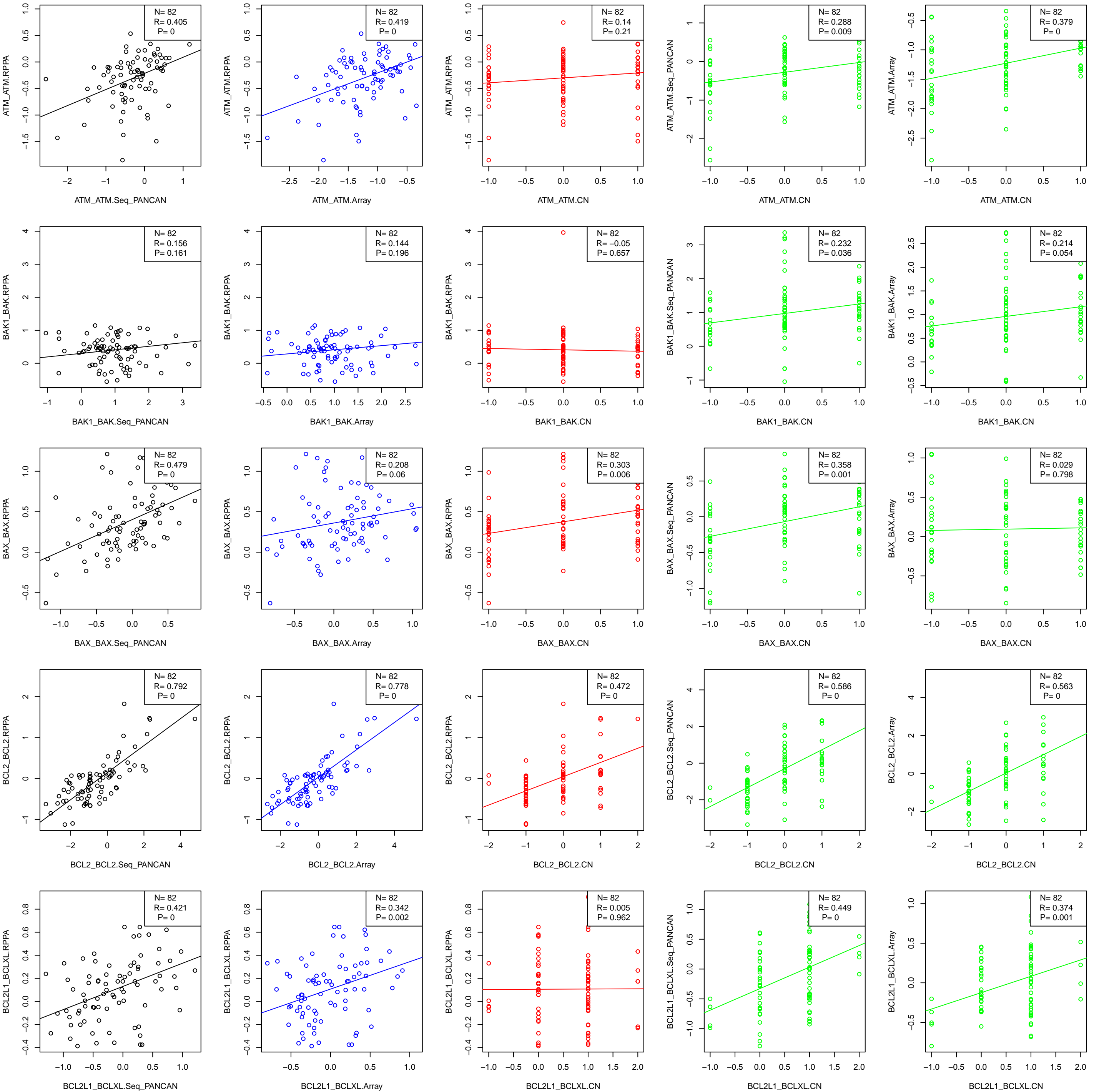

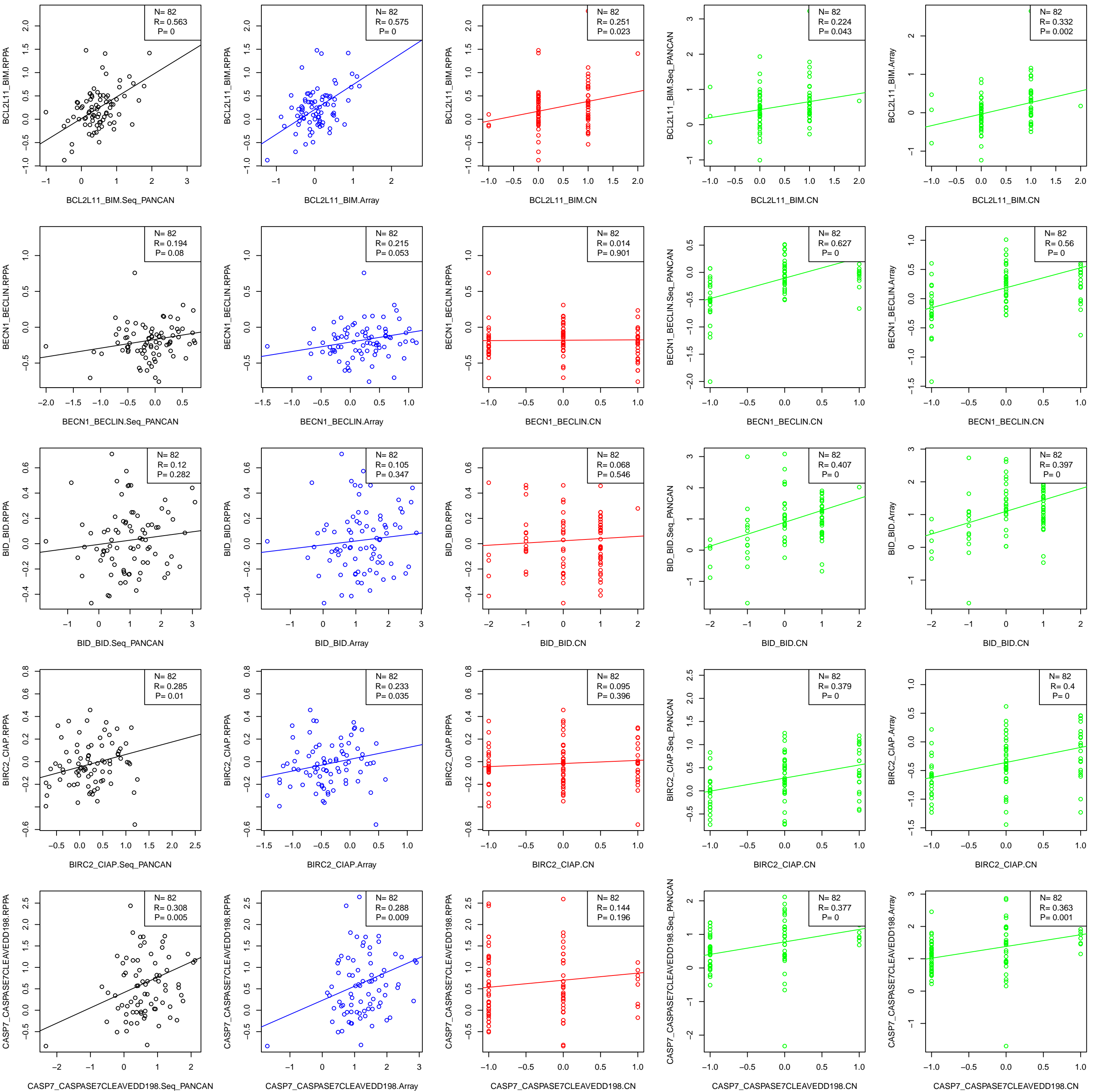

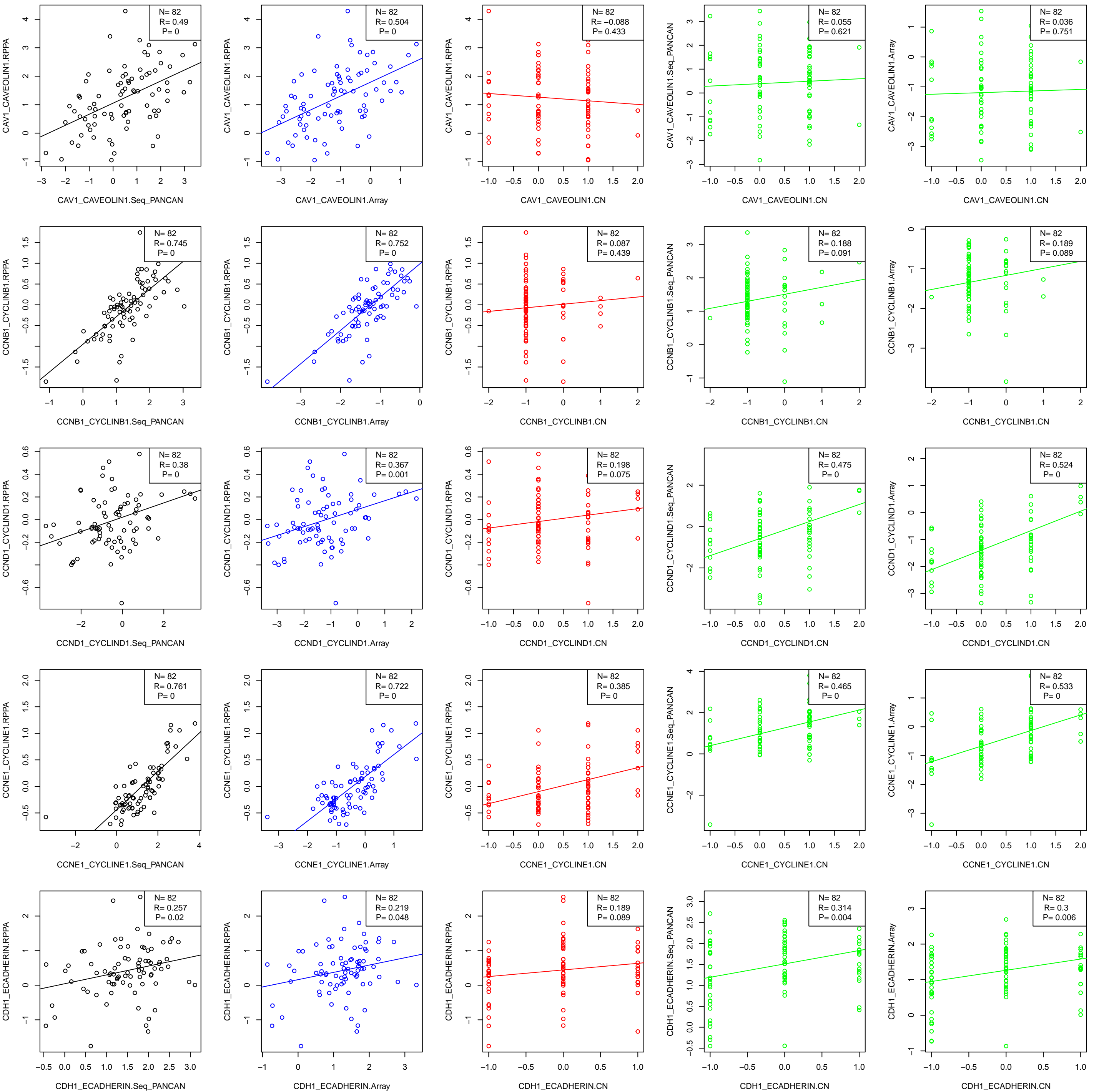

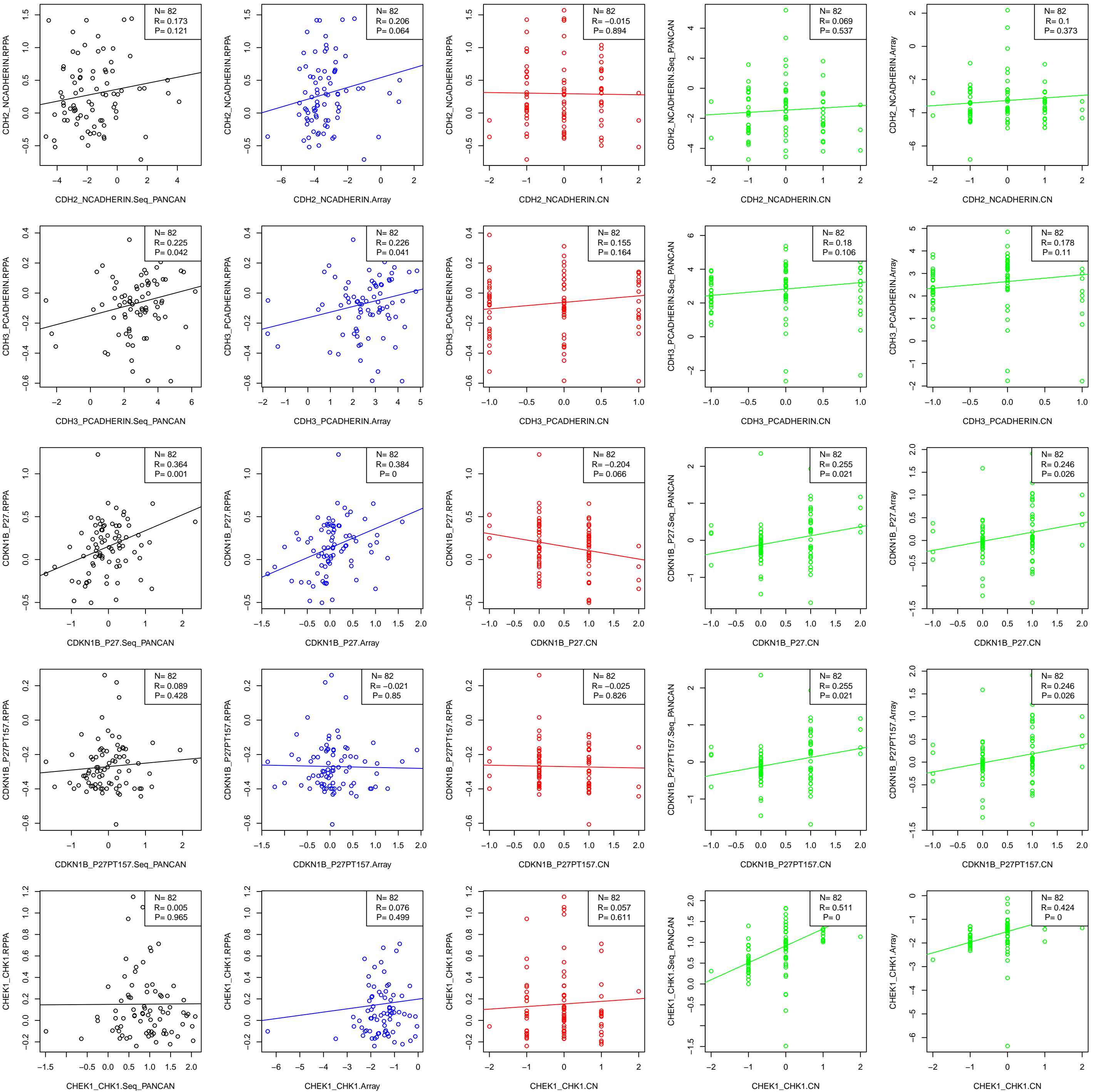

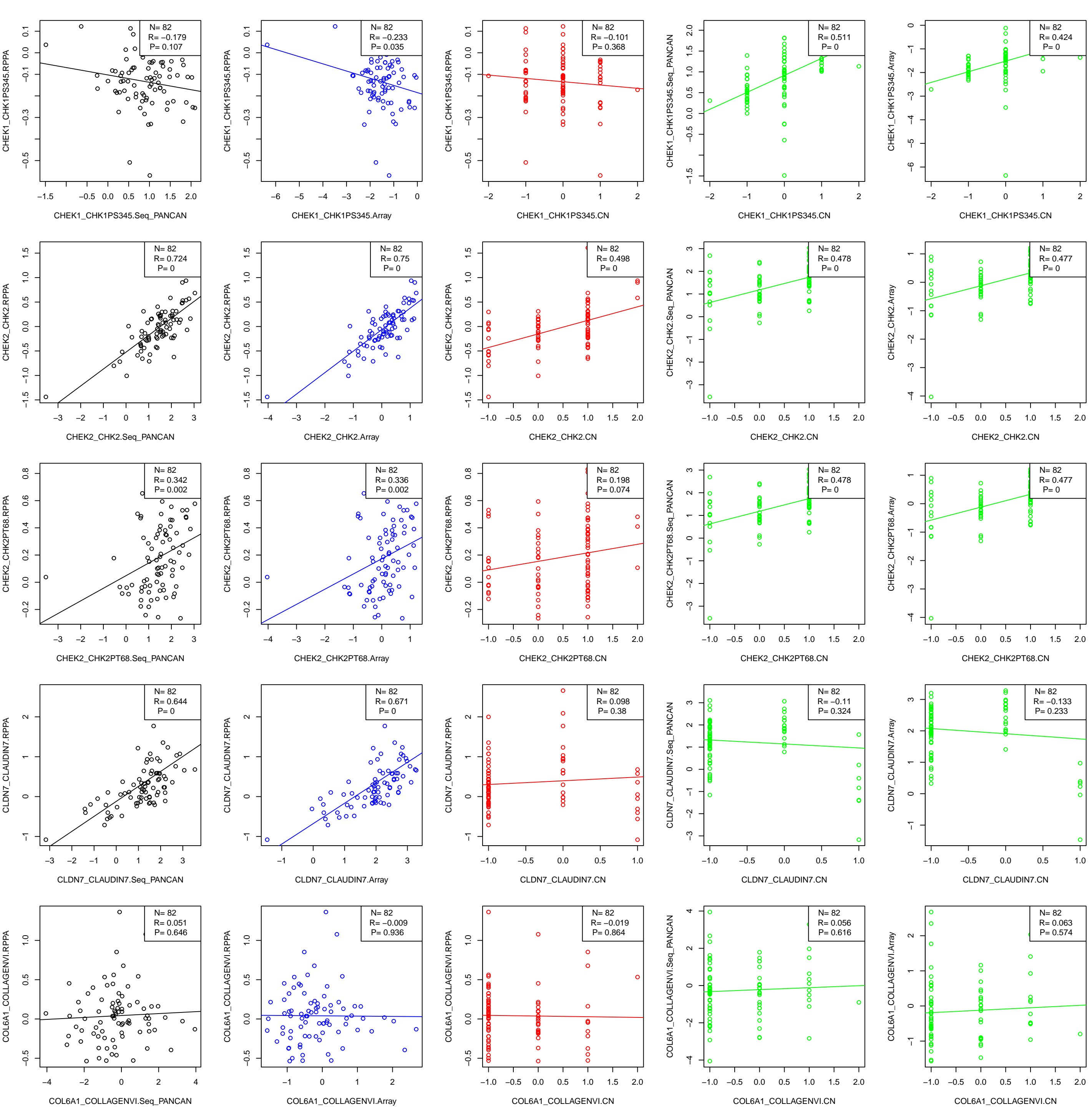

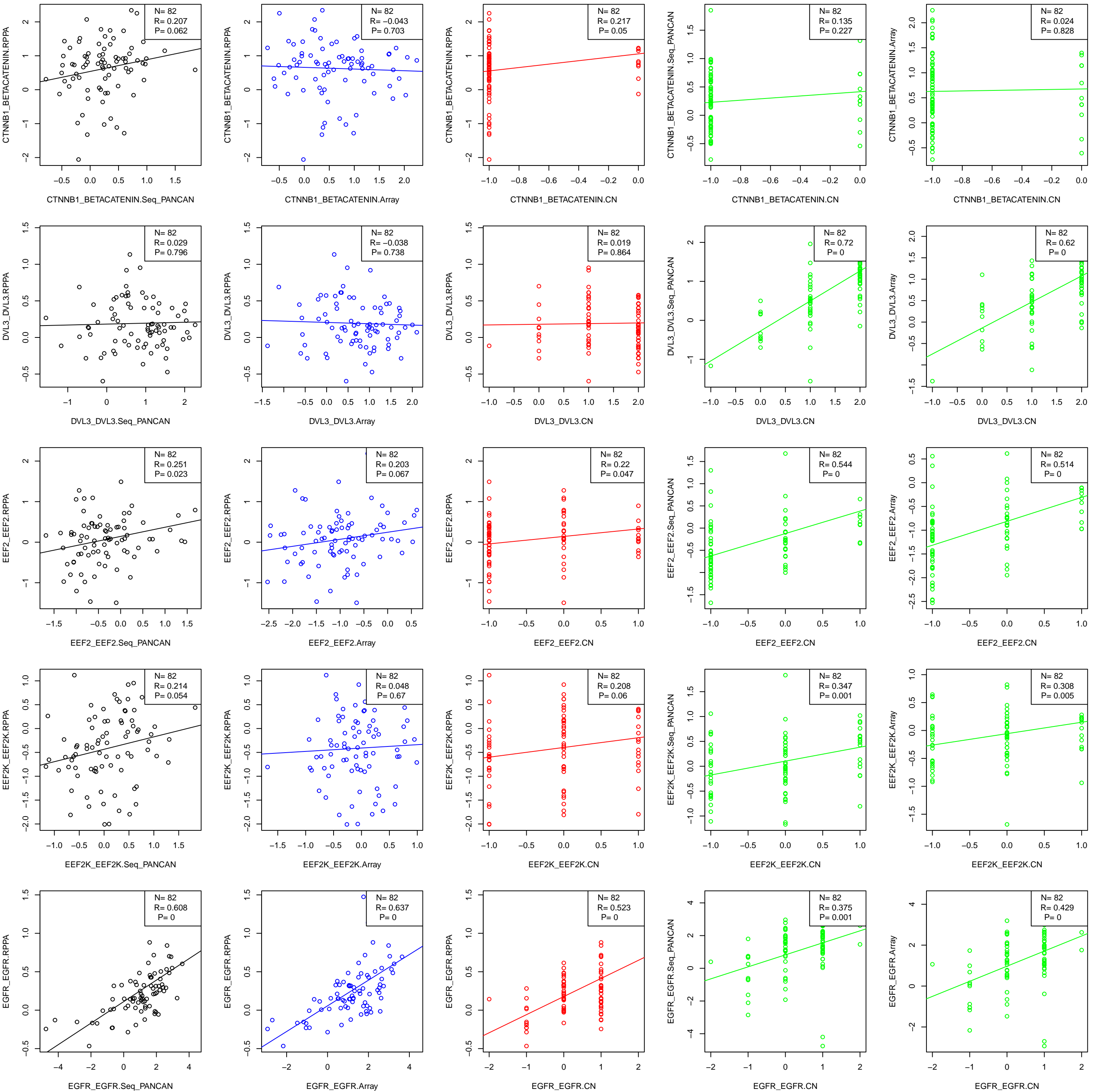

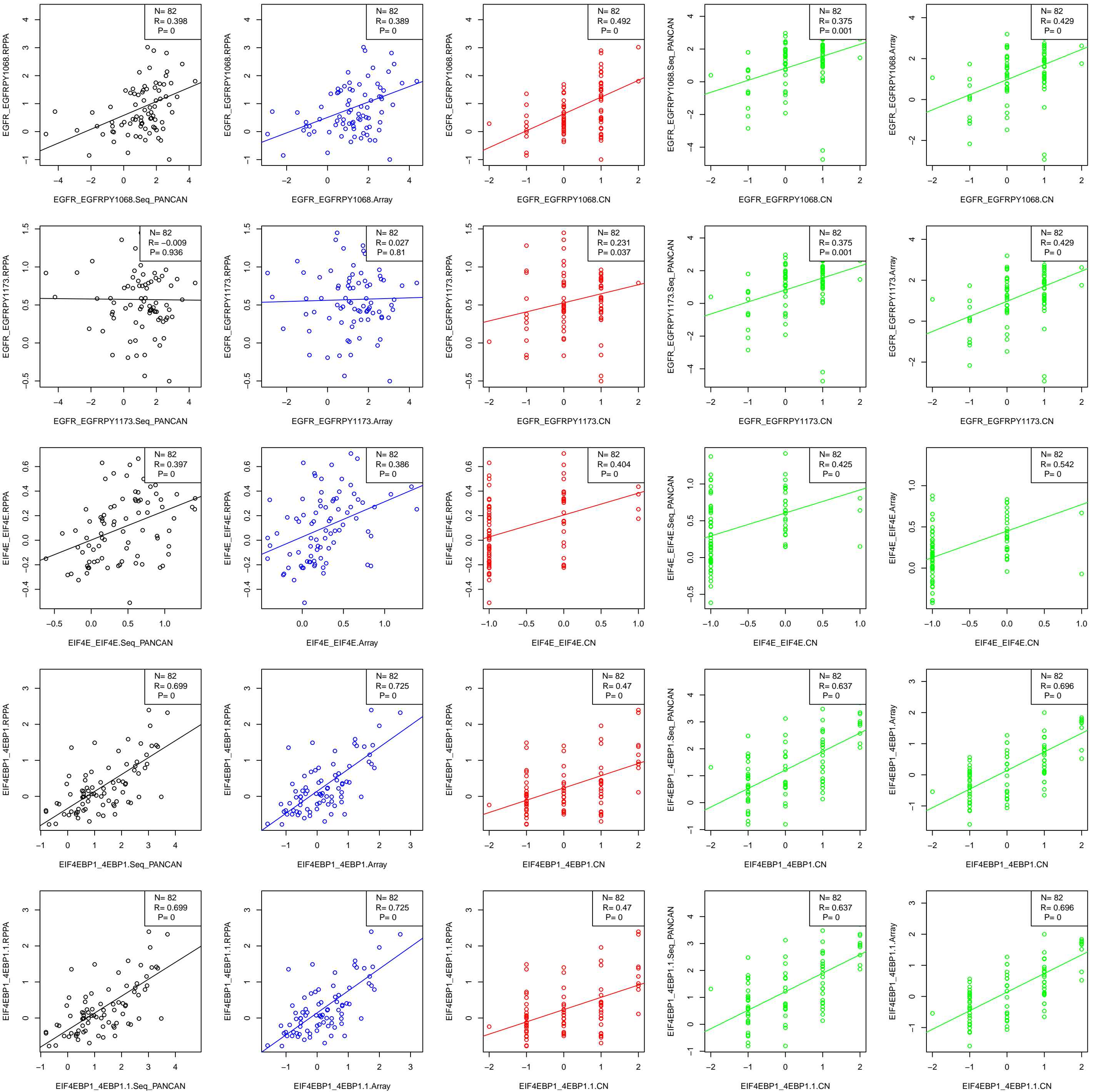

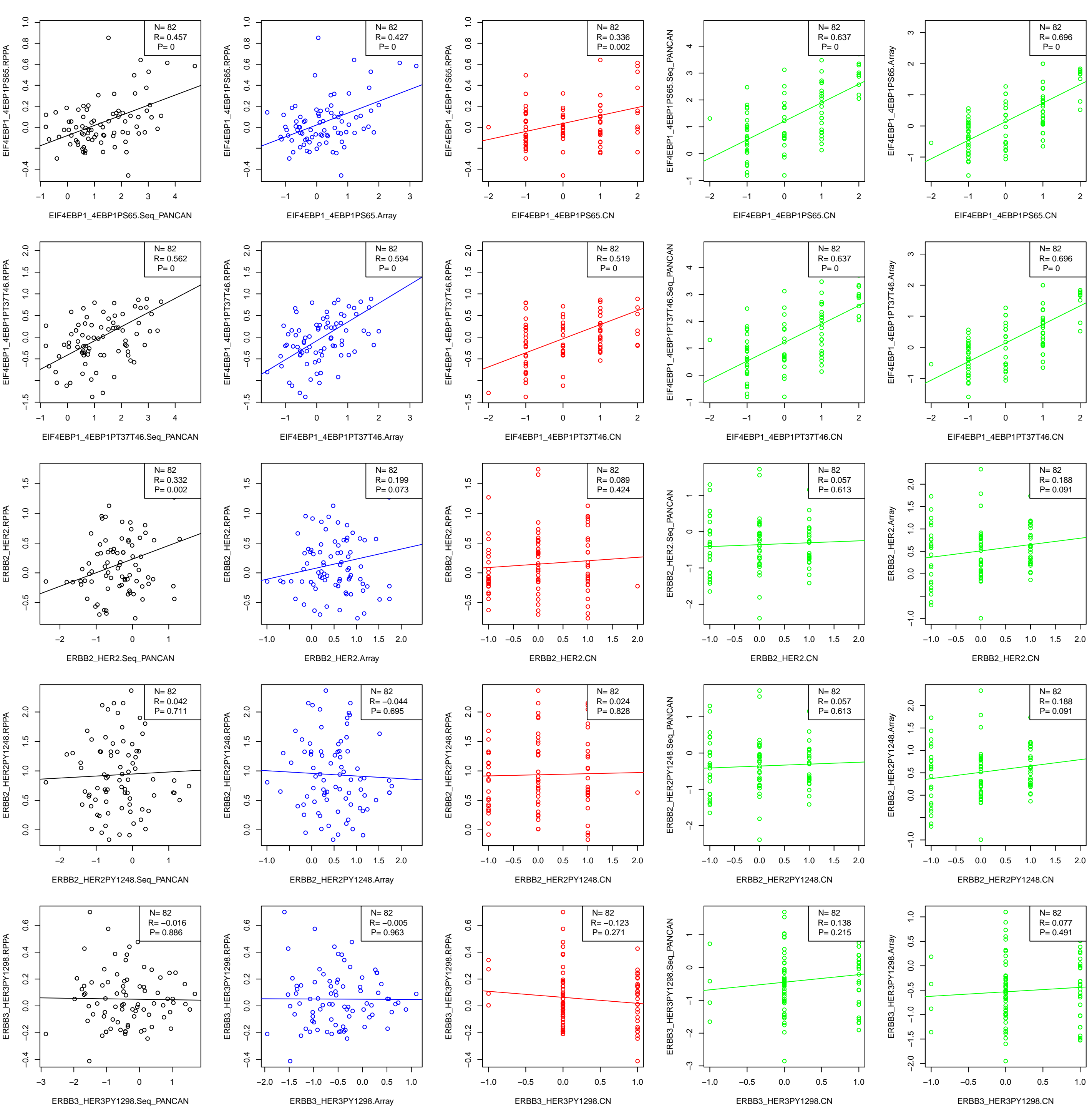

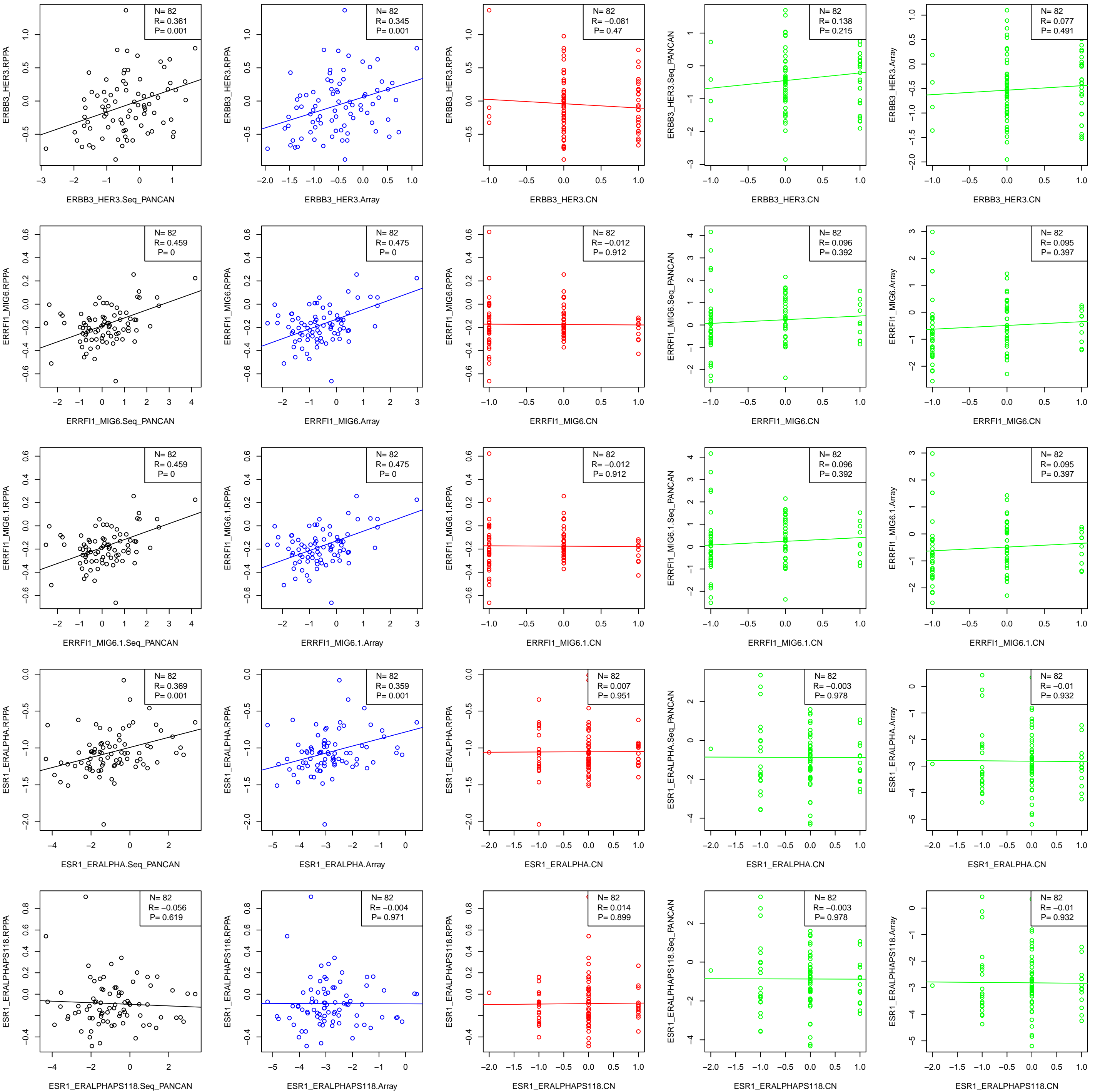

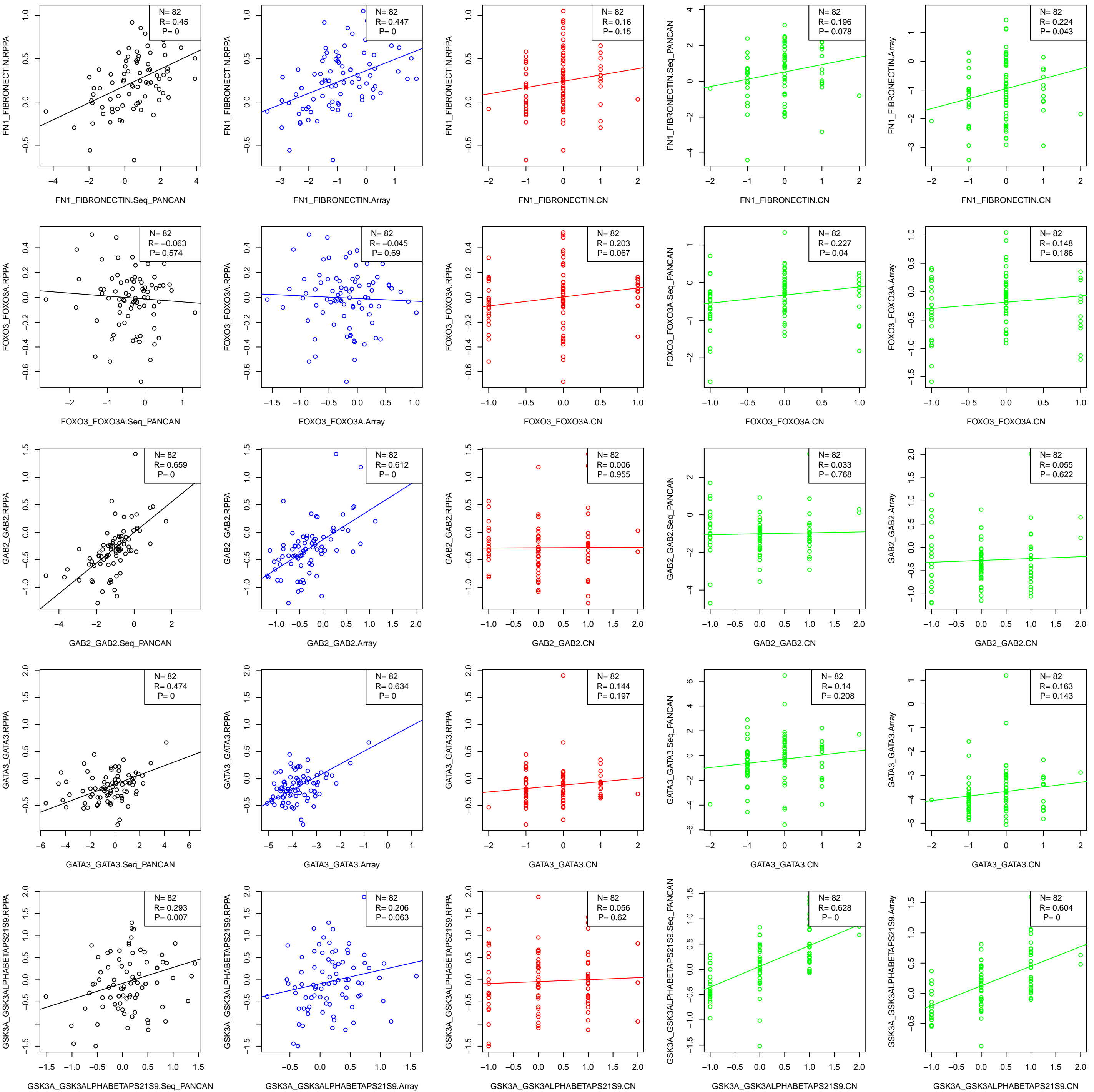

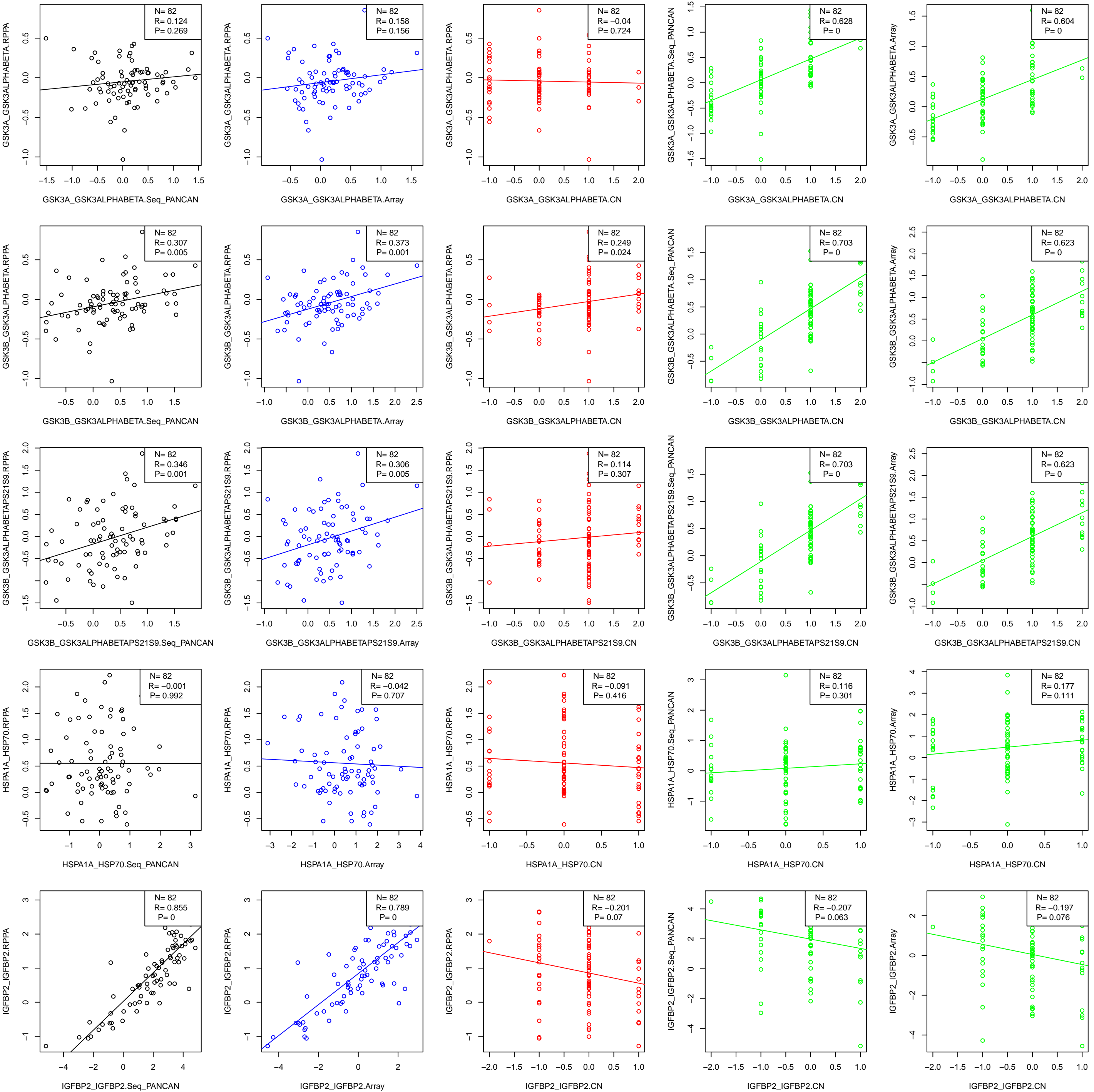

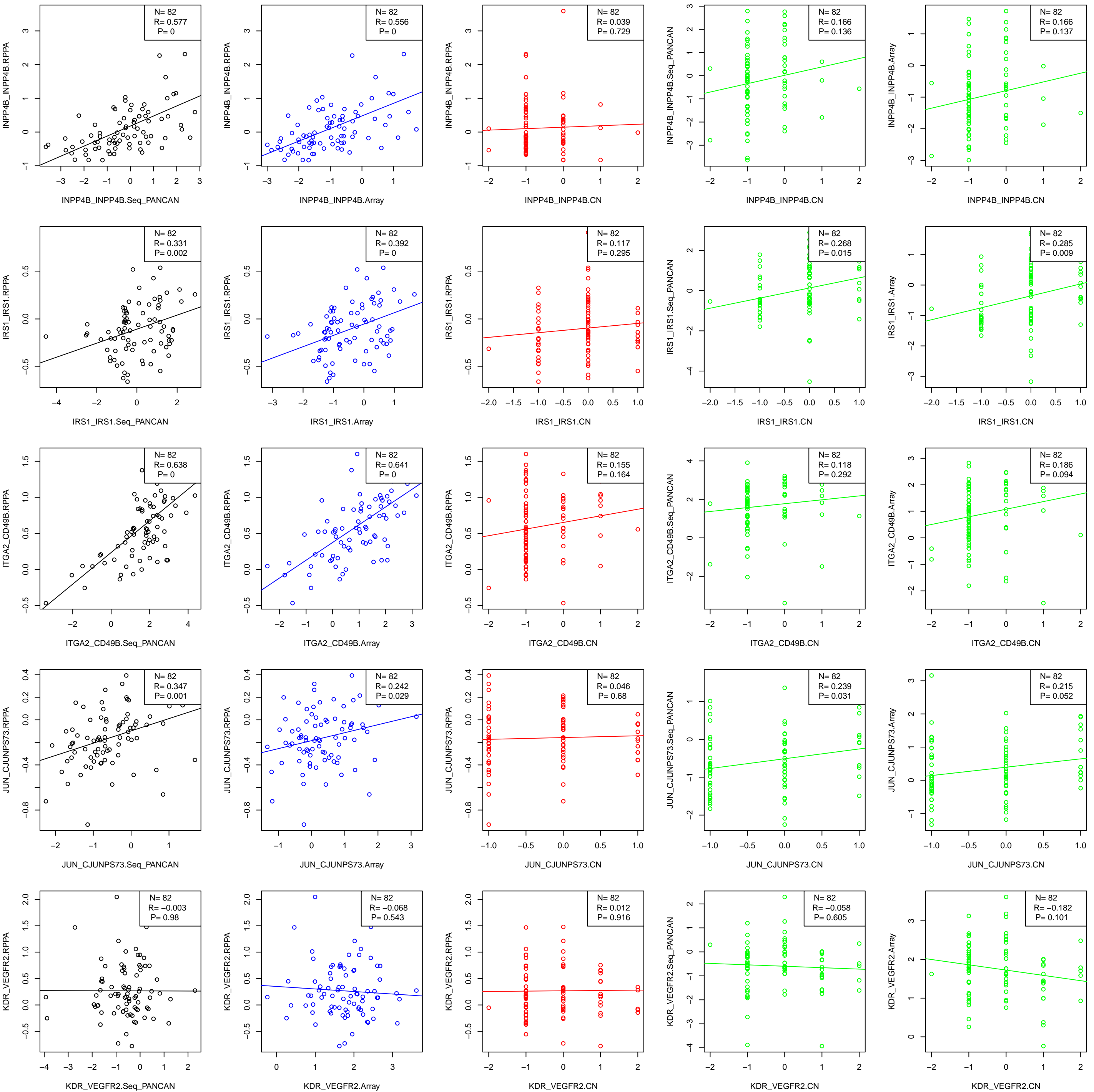

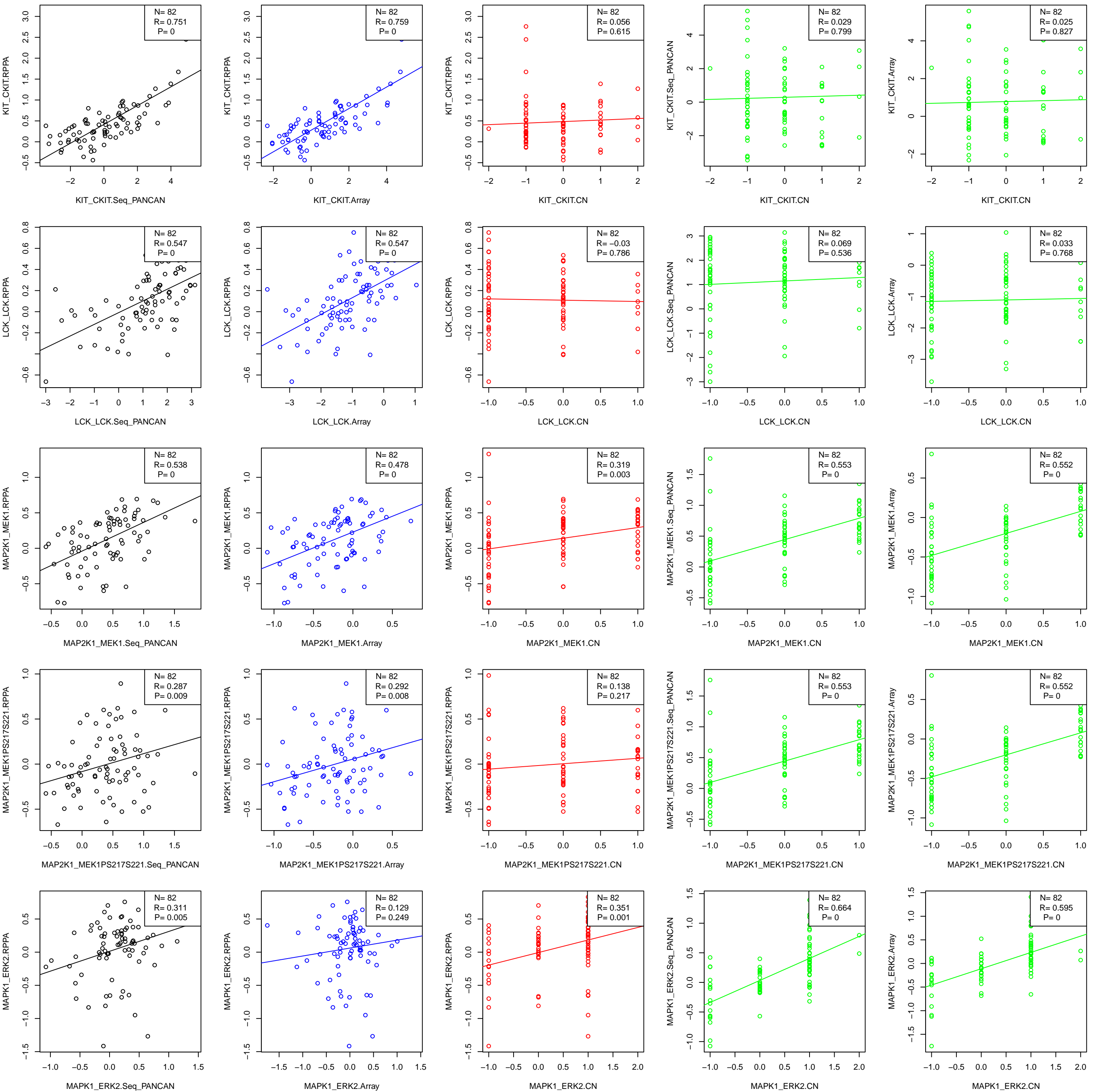

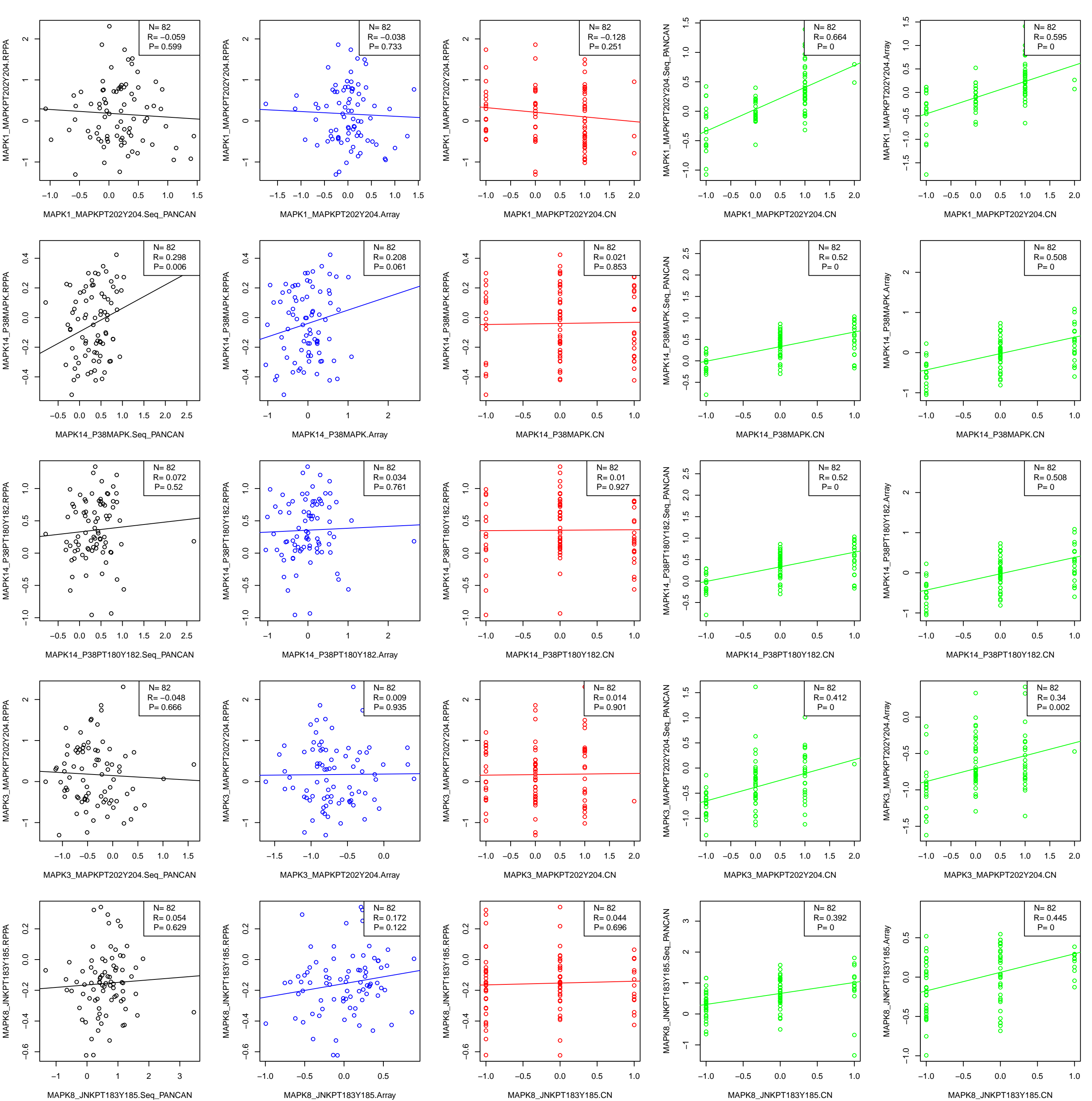

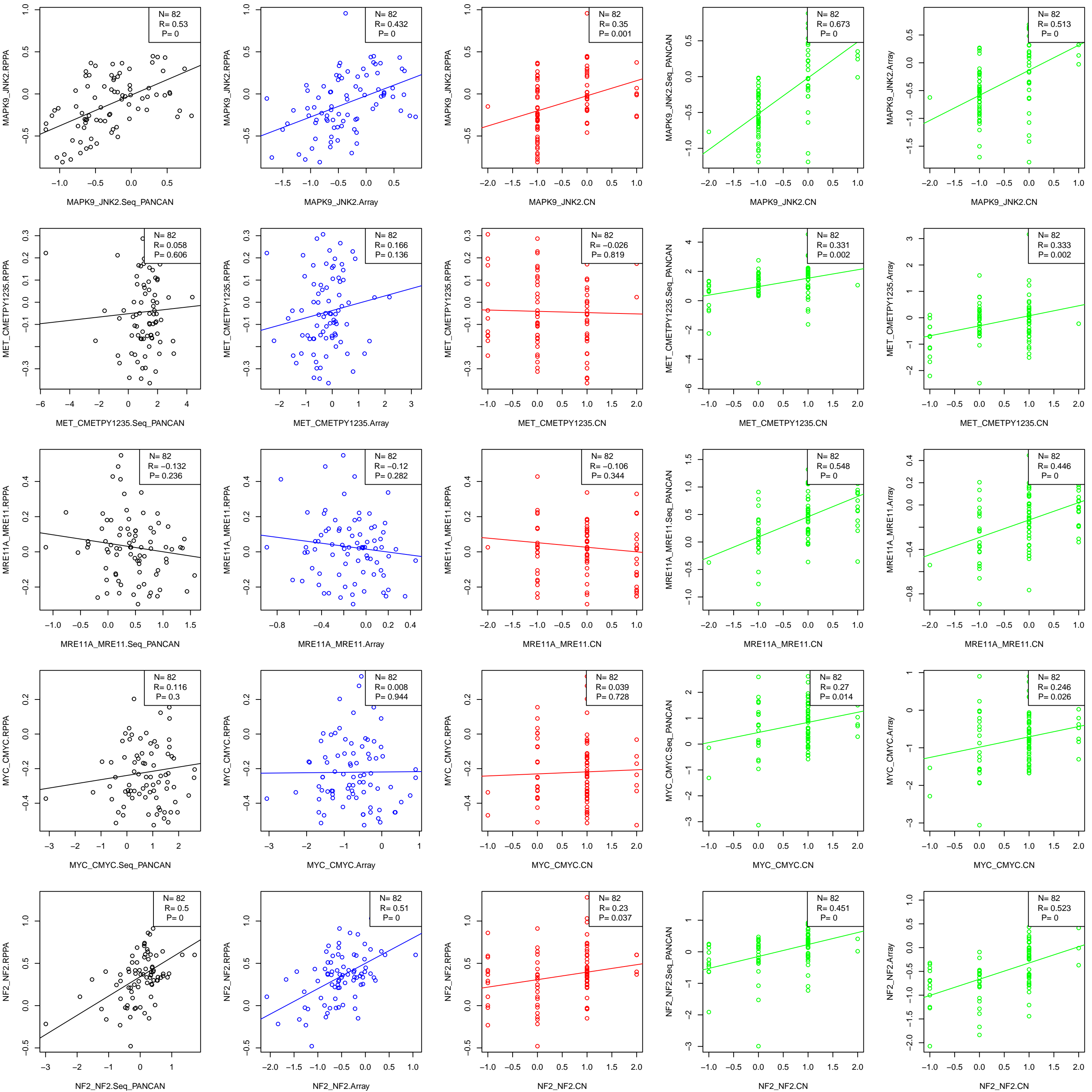

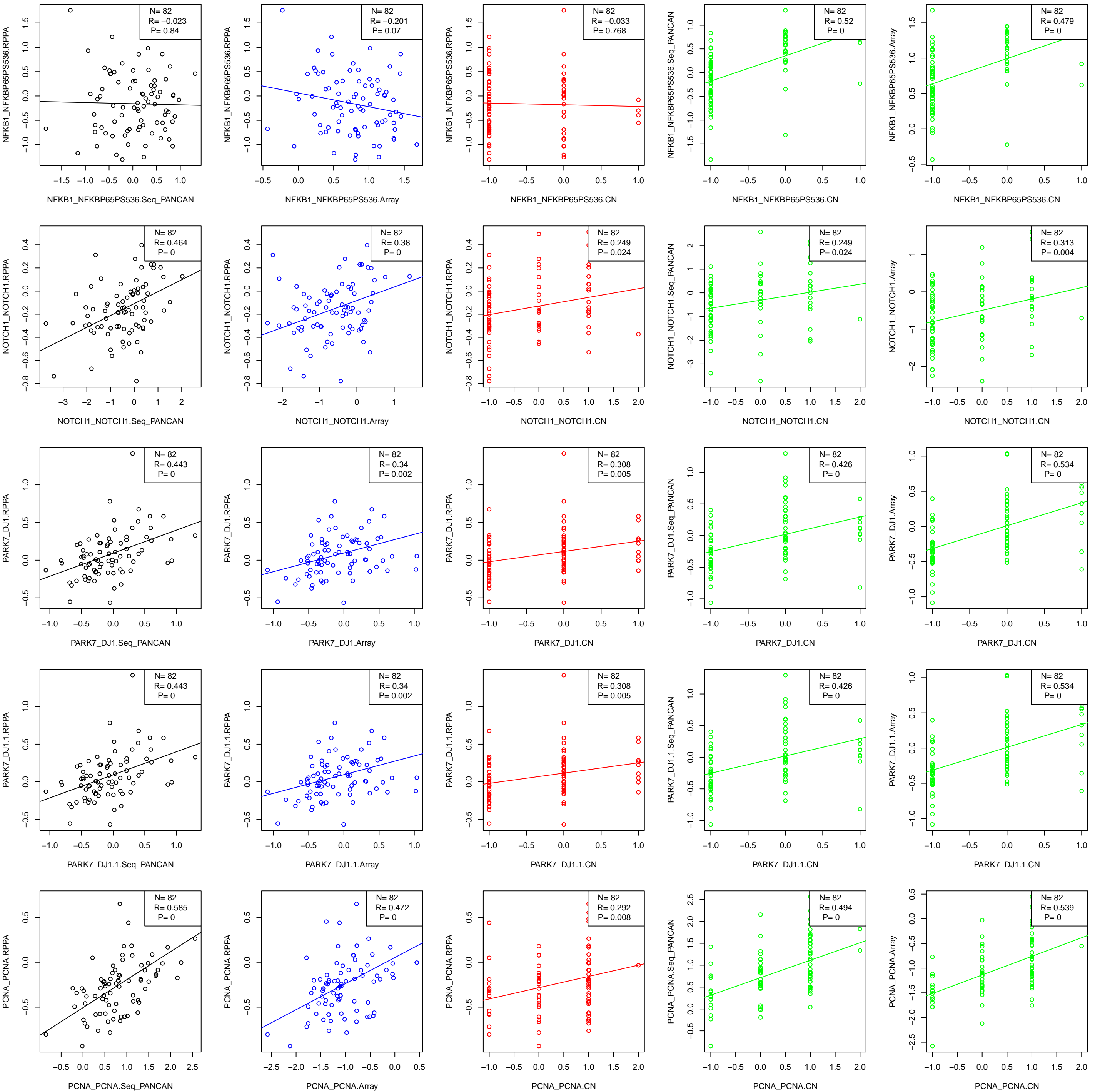

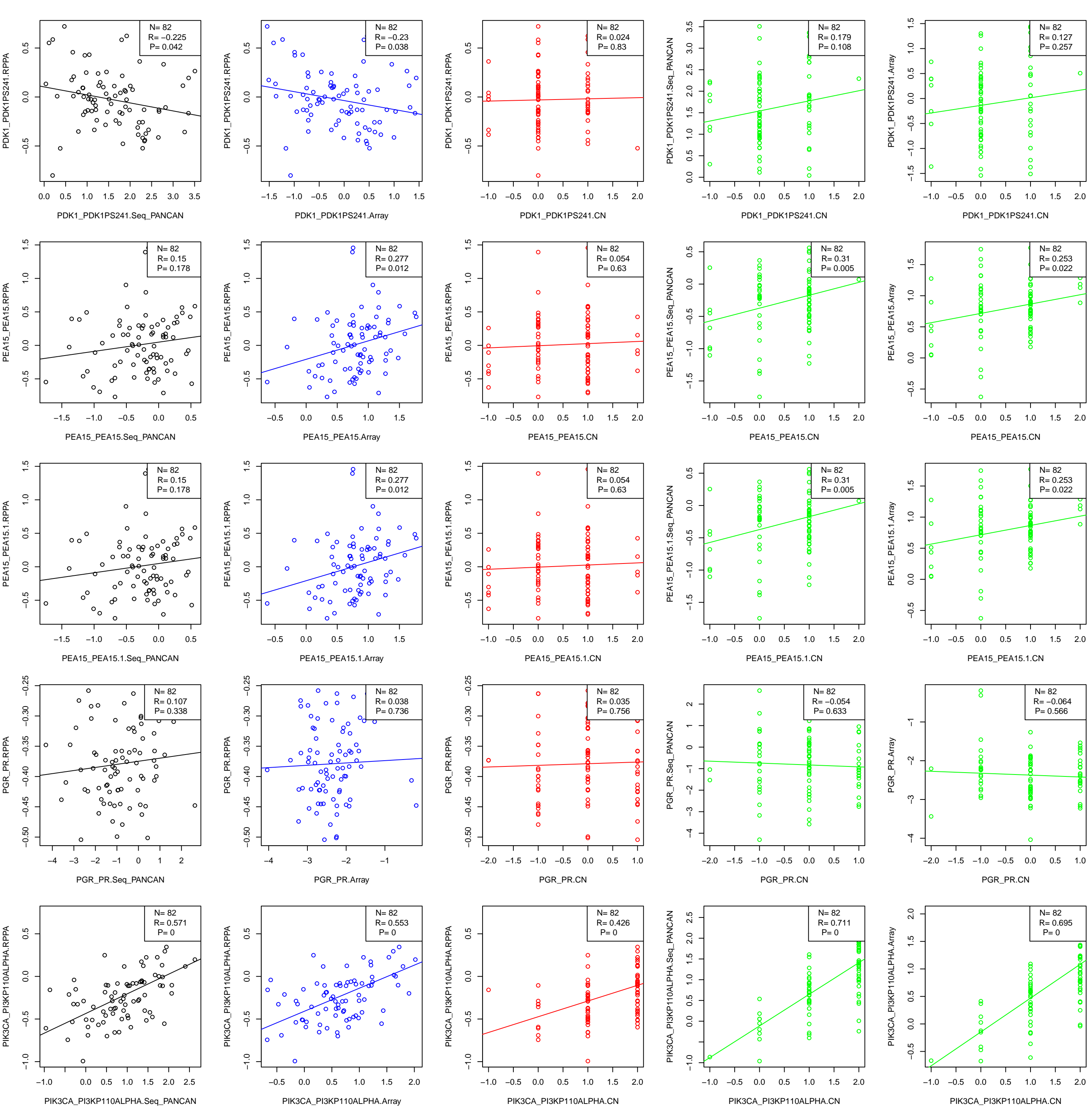

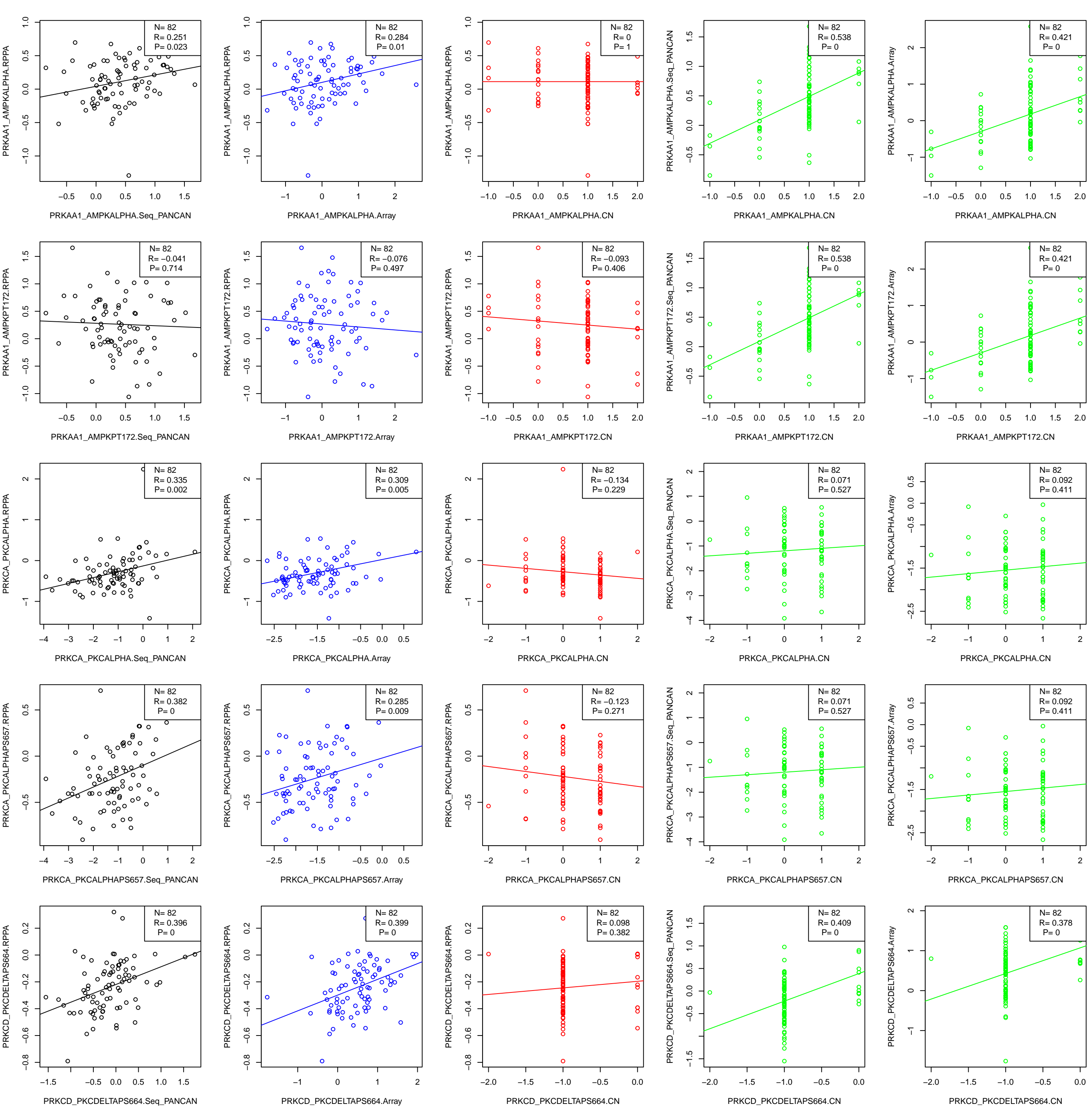

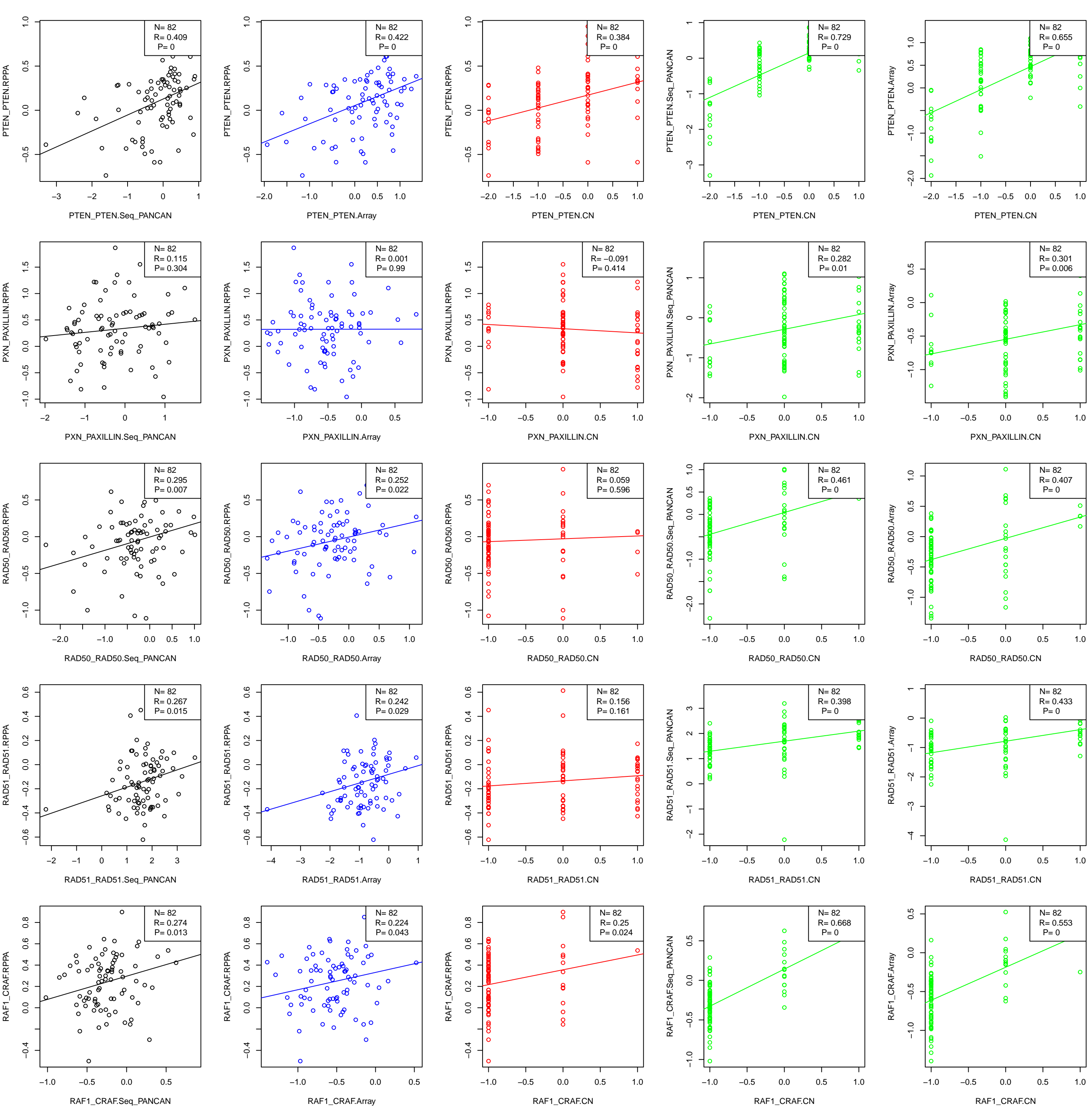

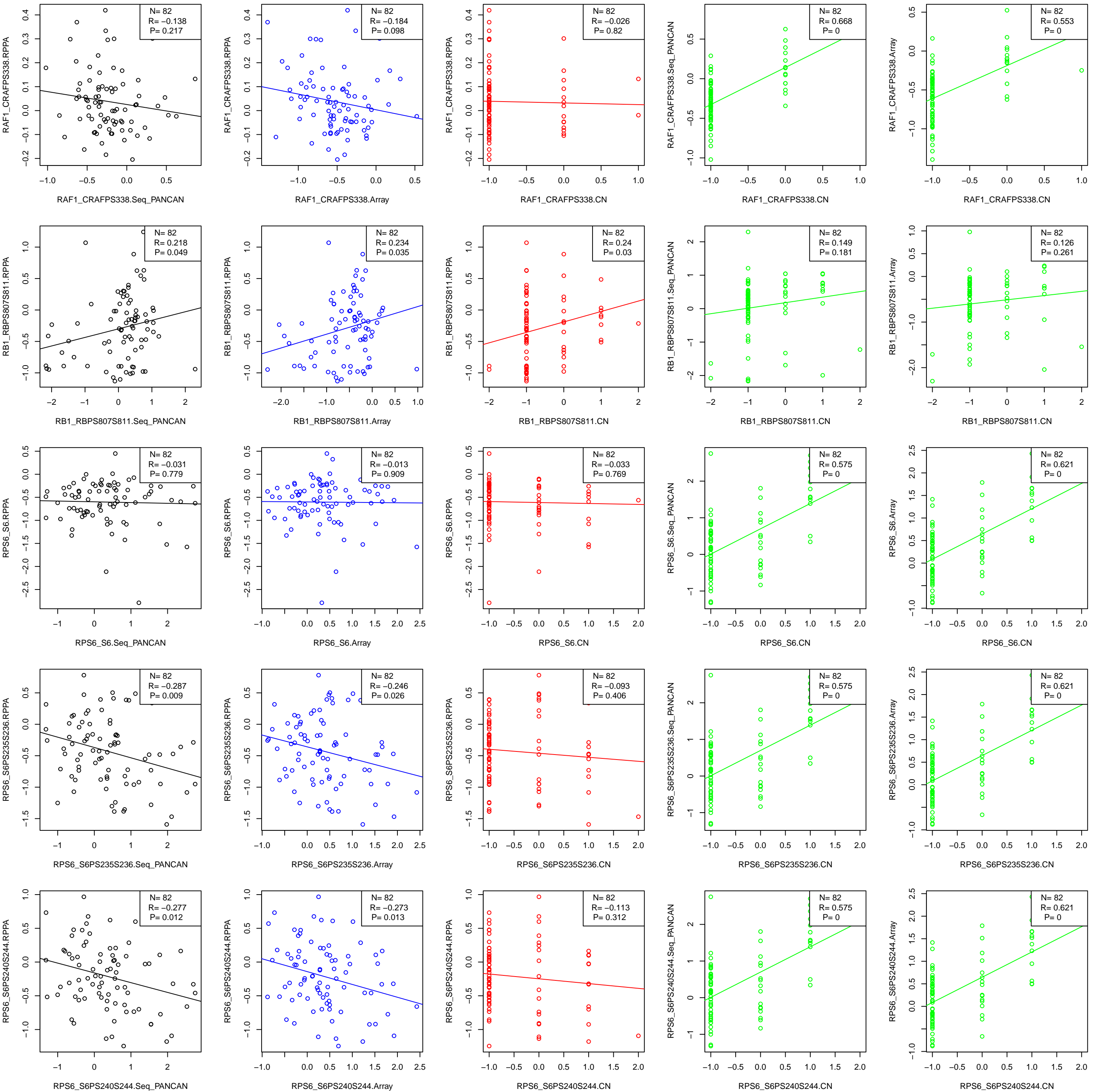

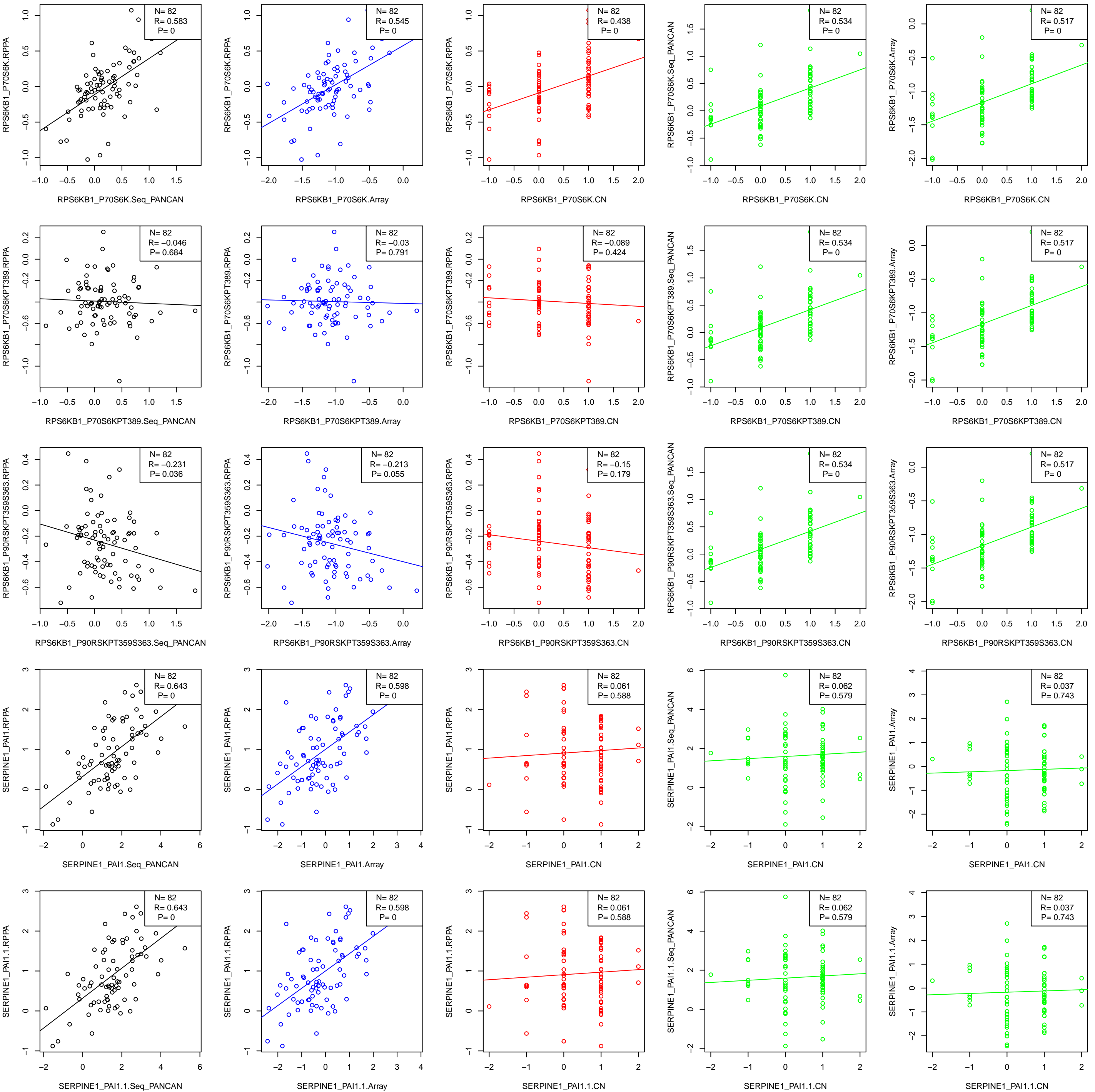

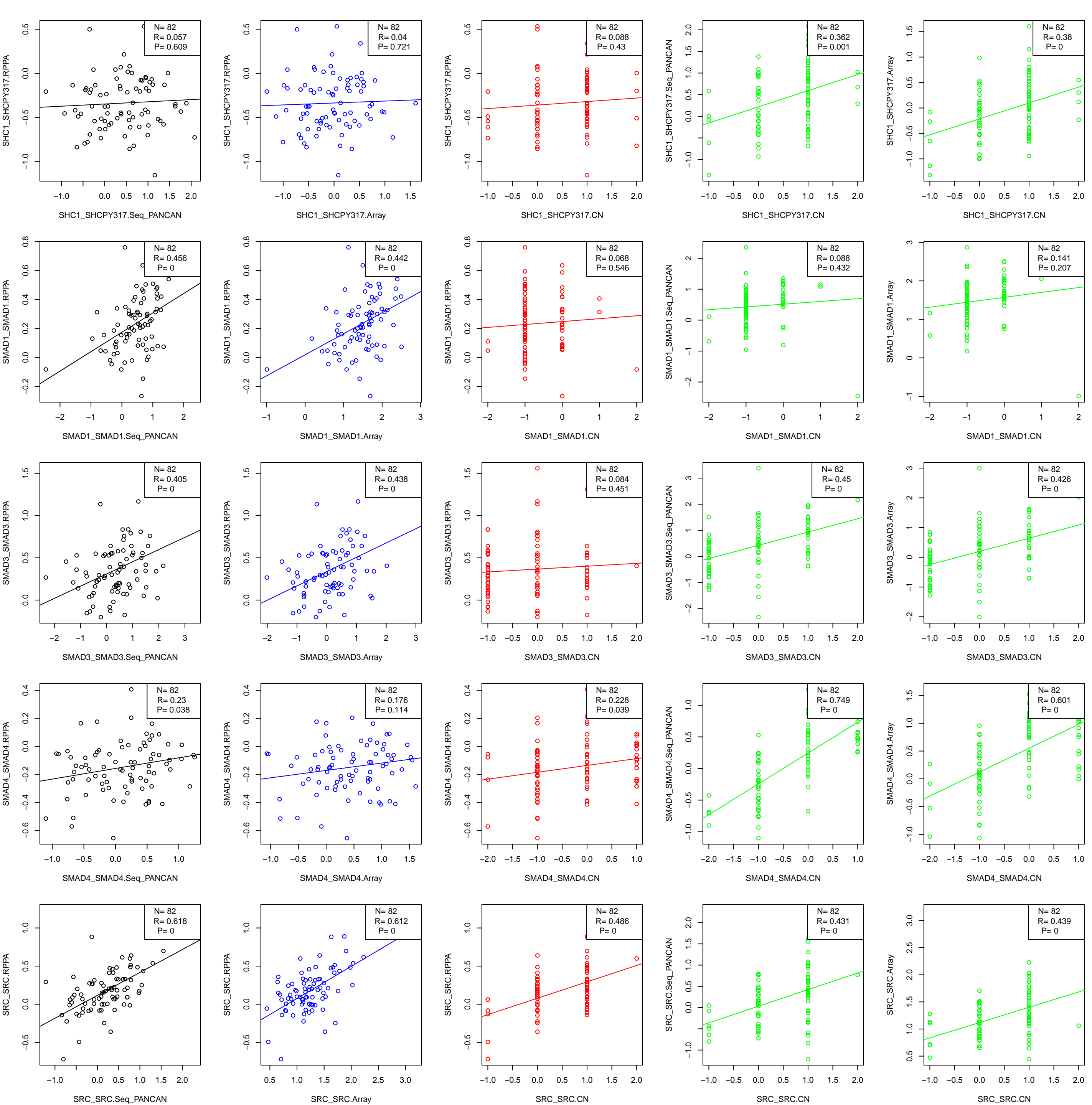

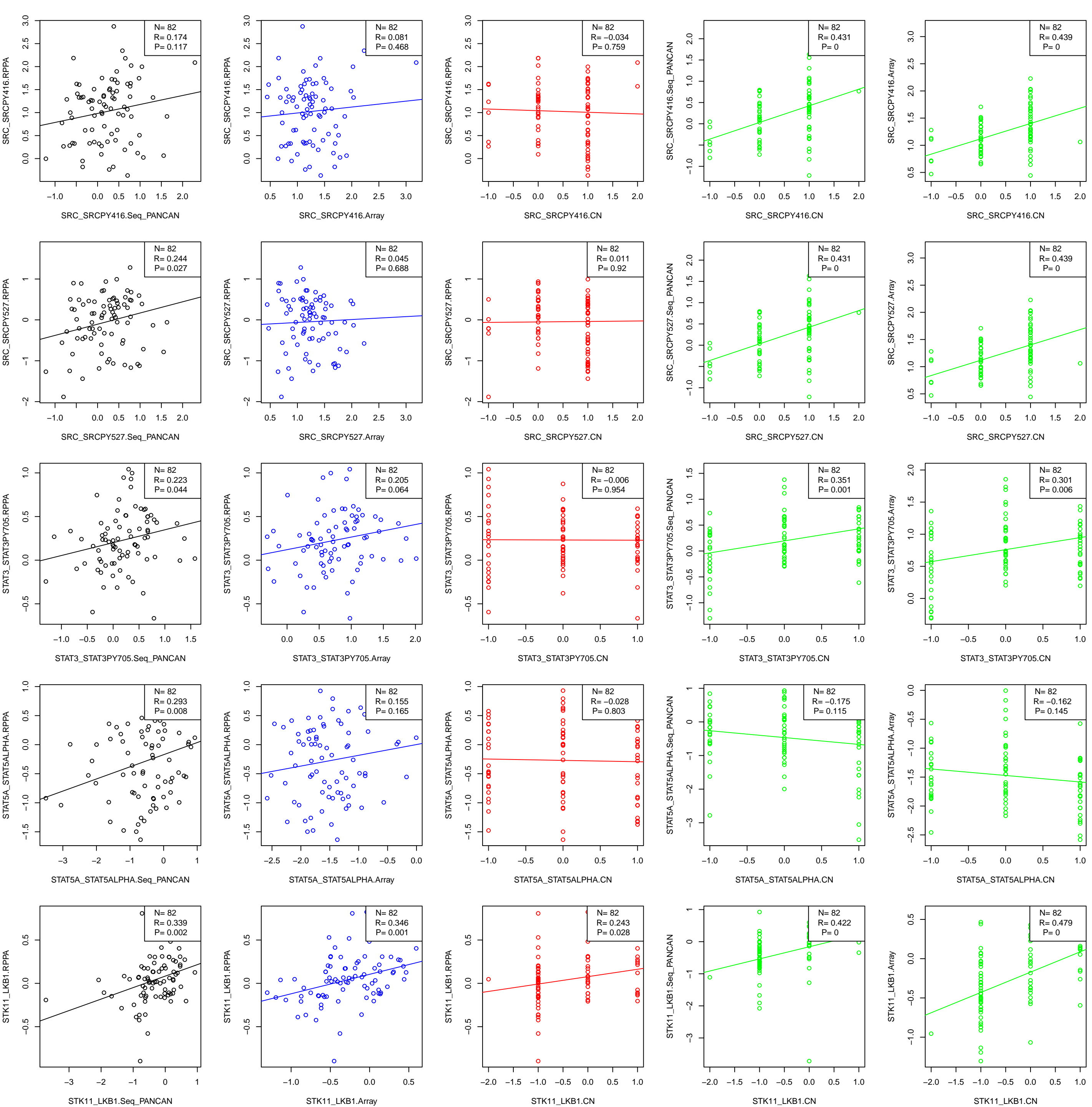

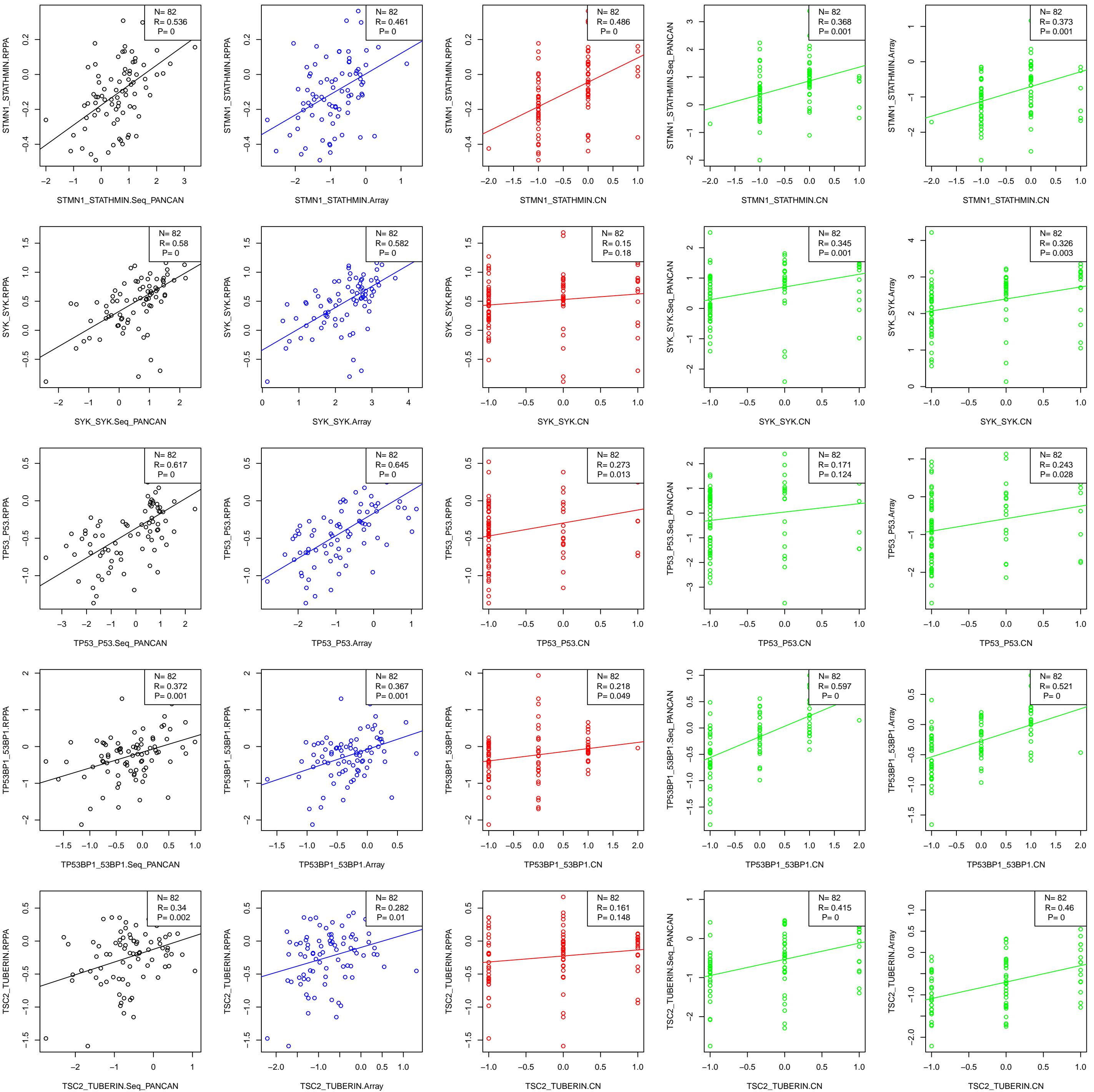

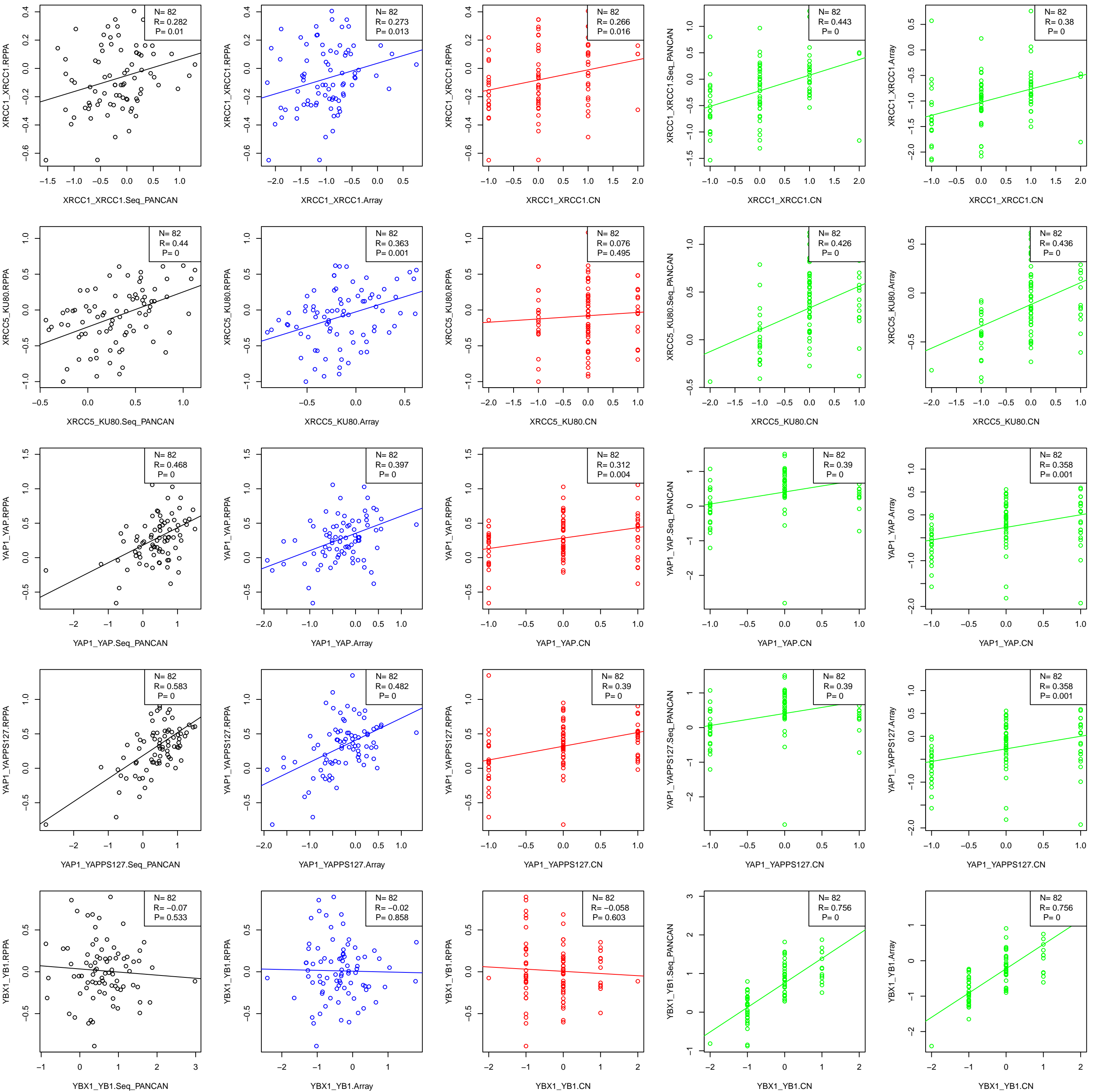

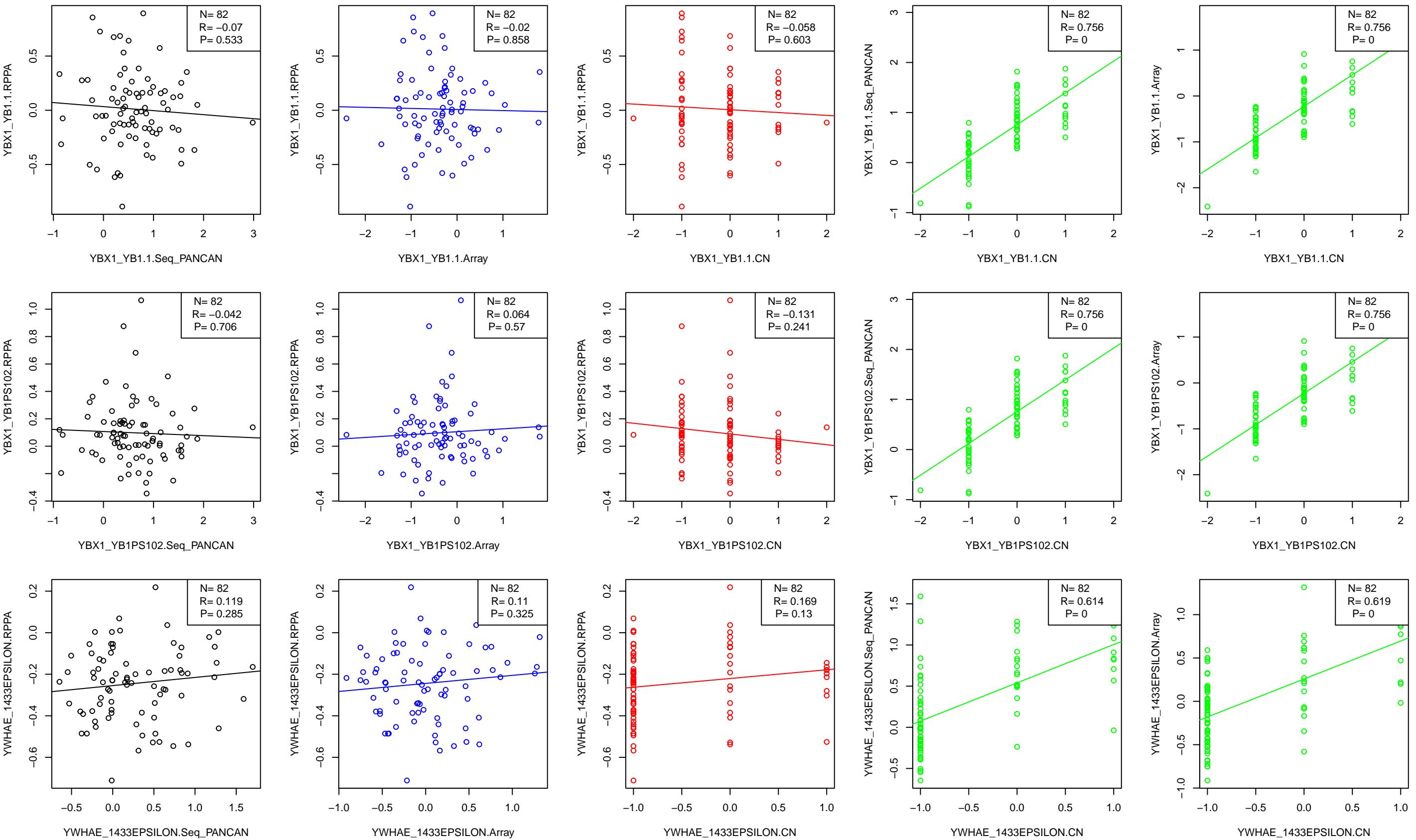

Supplement: Supplementary file 11 [file DataSheet5.PDF]

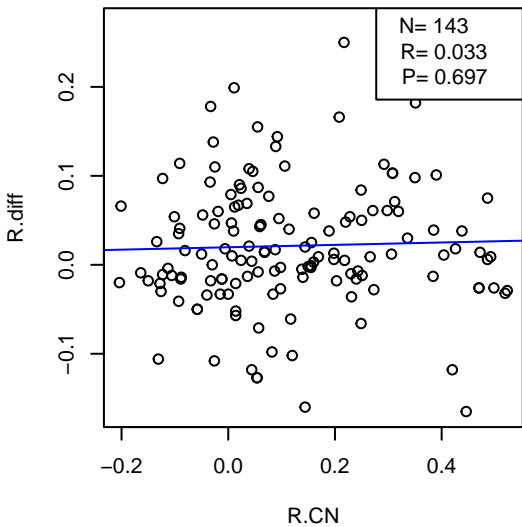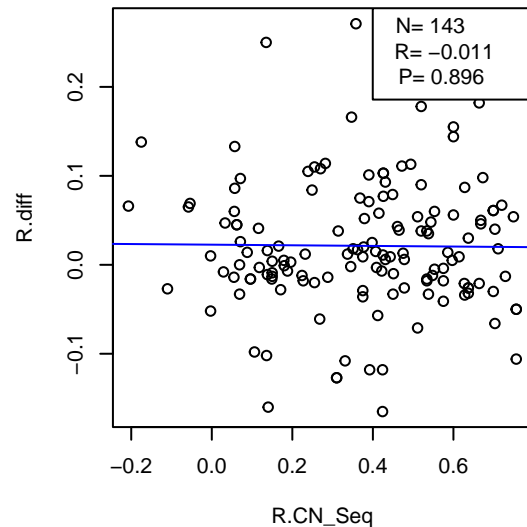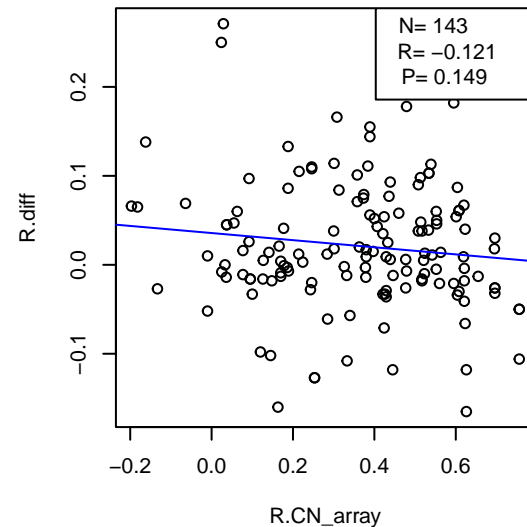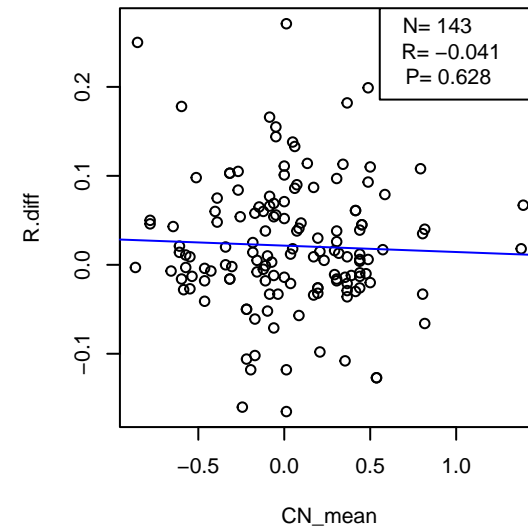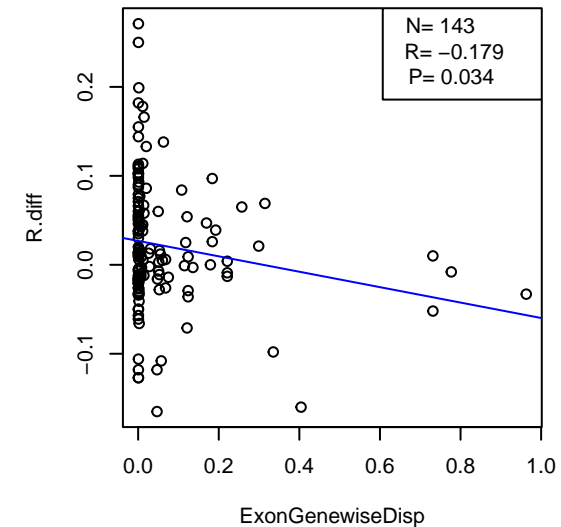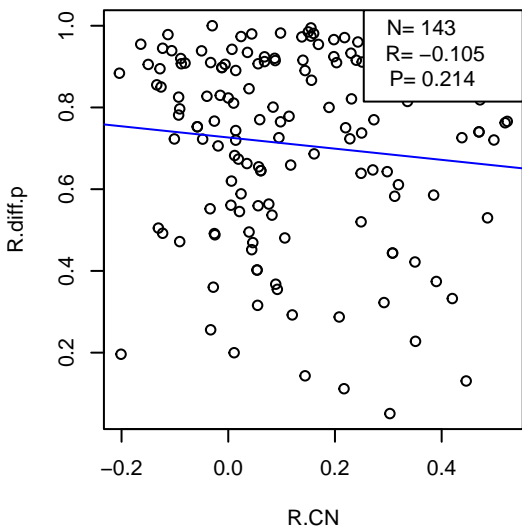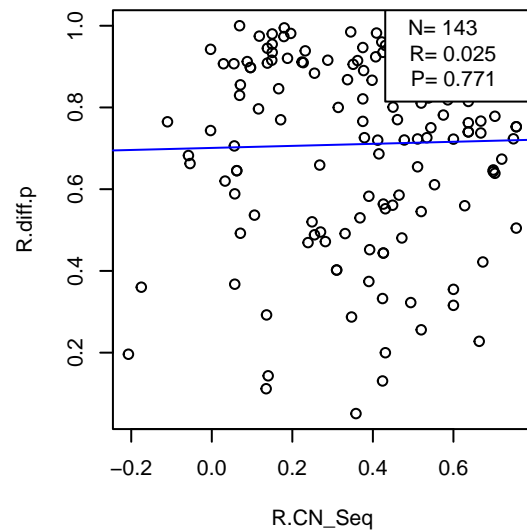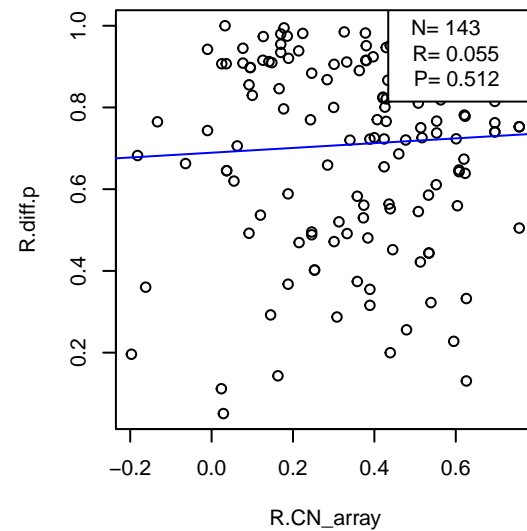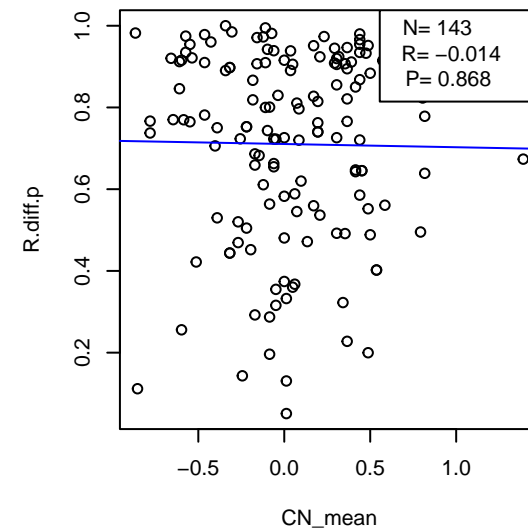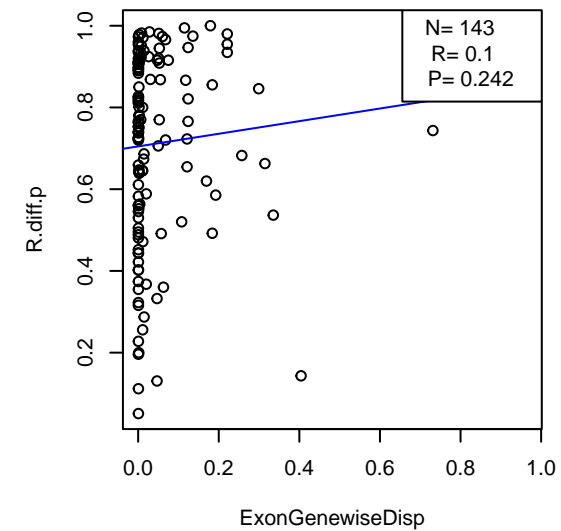

Supplement: Supplementary file 12 [file DataSheet12.PDF]

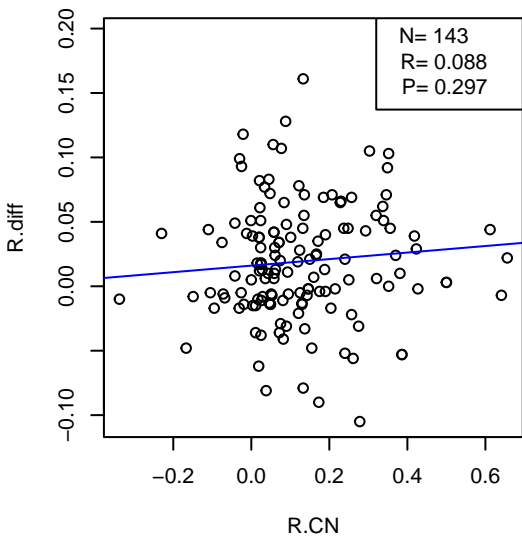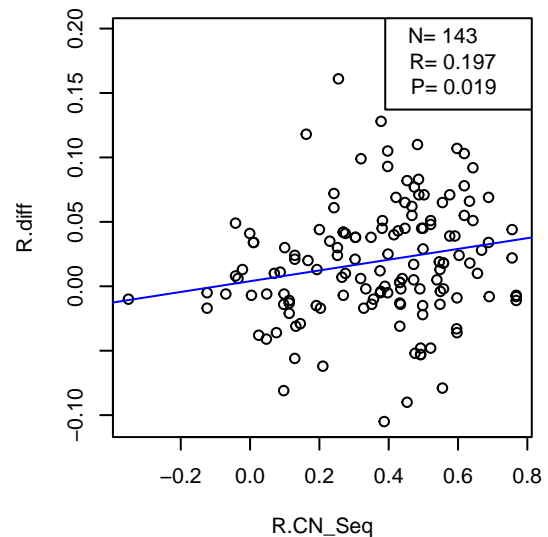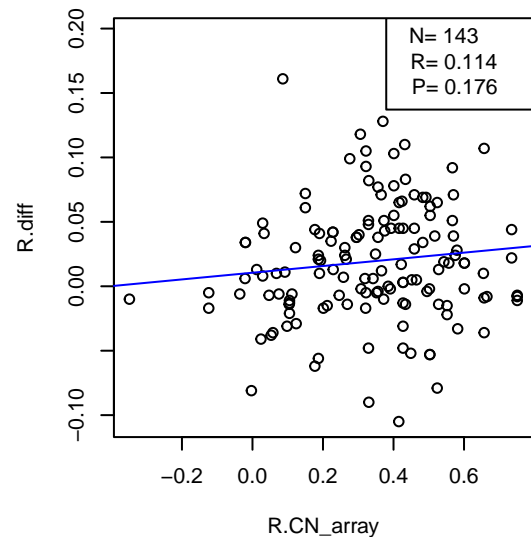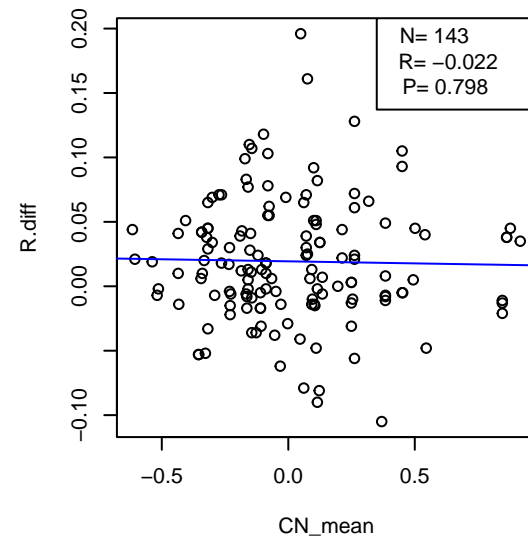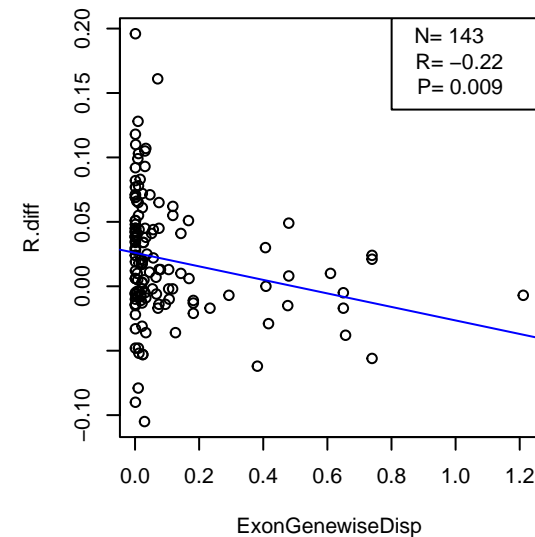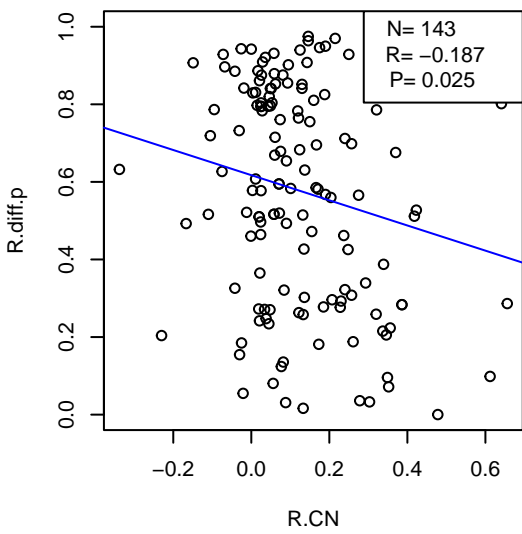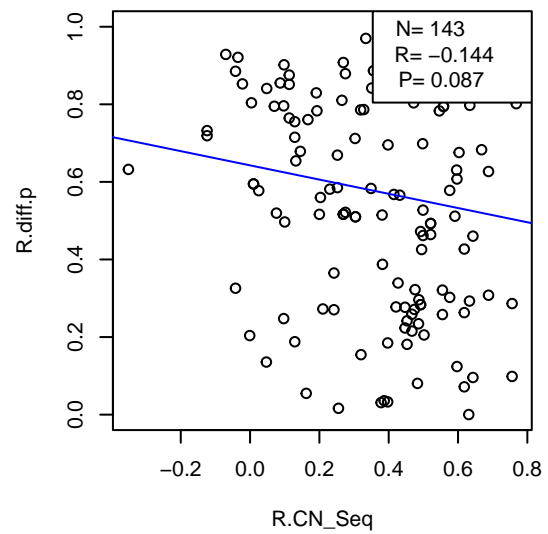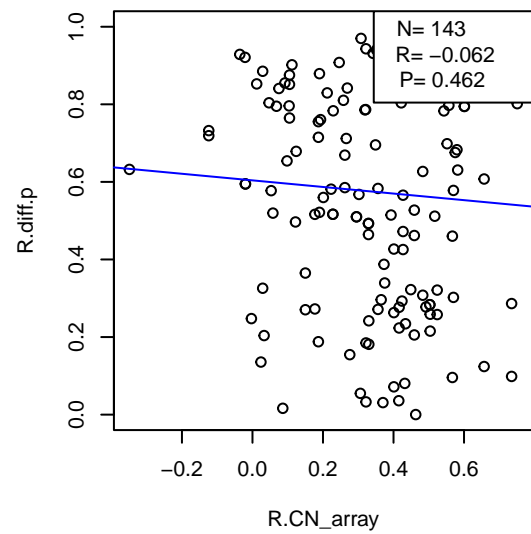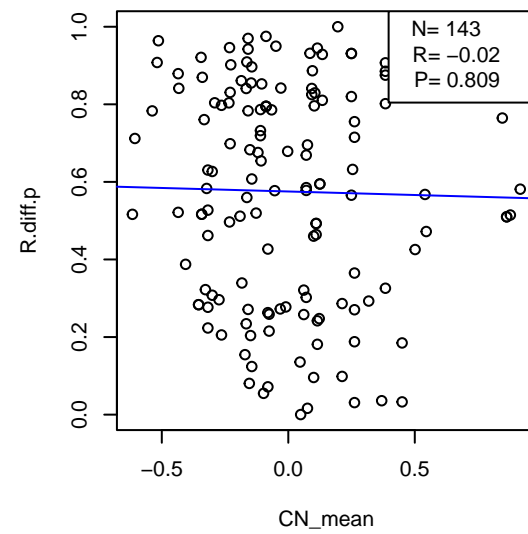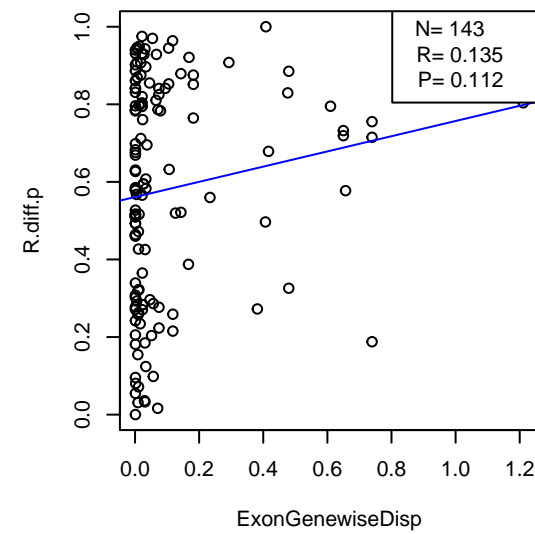

Supplement: Supplementary file 13 [file DataSheet8.PDF]

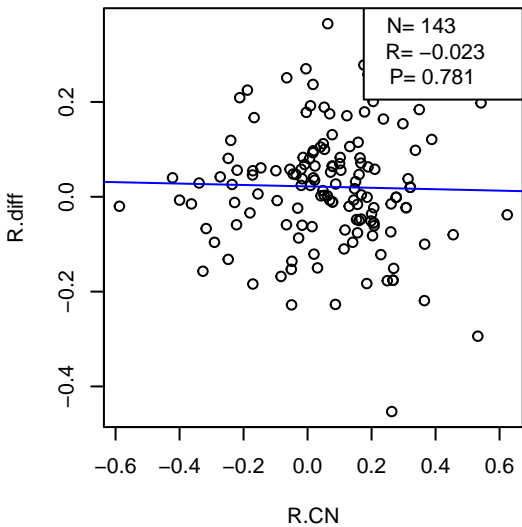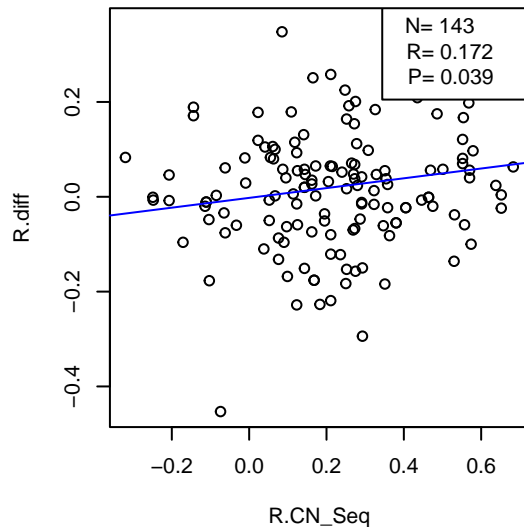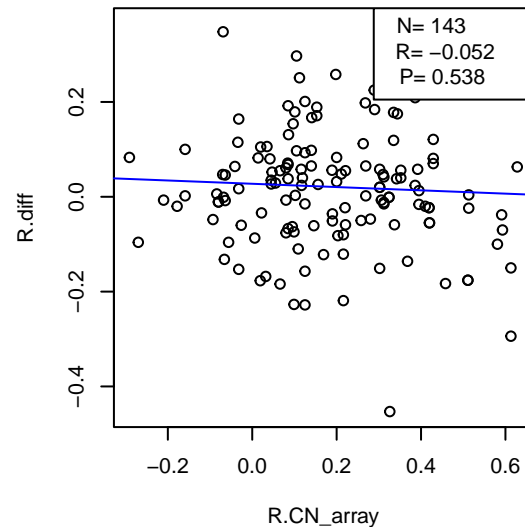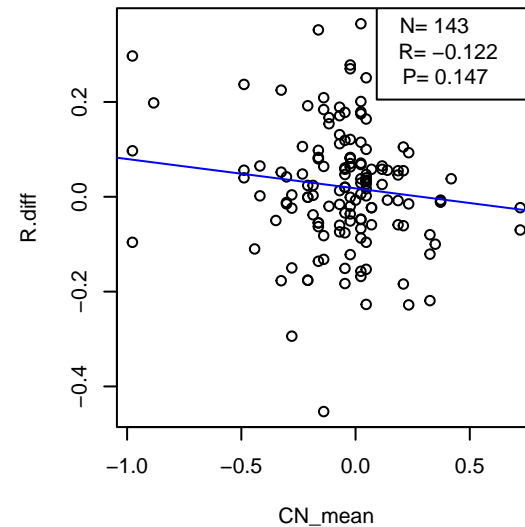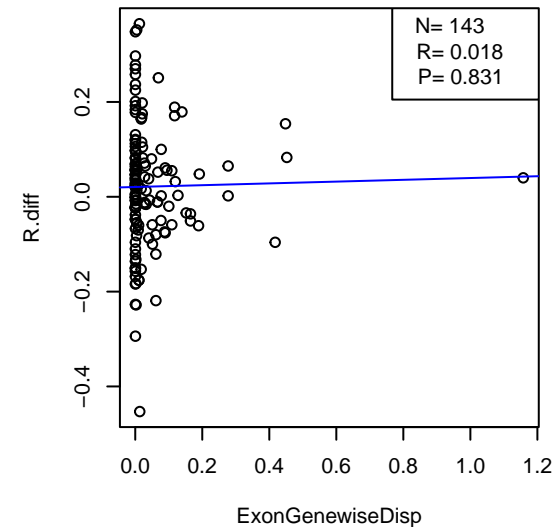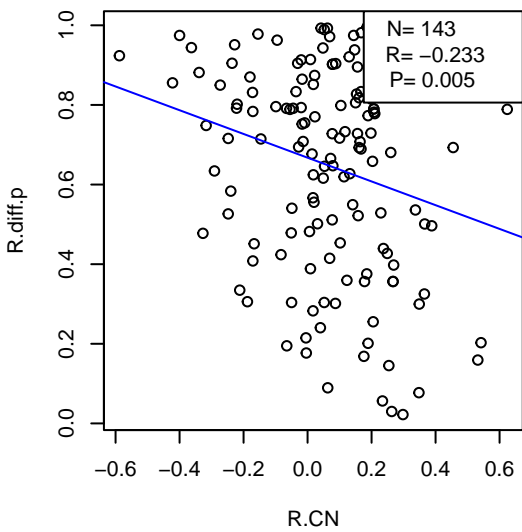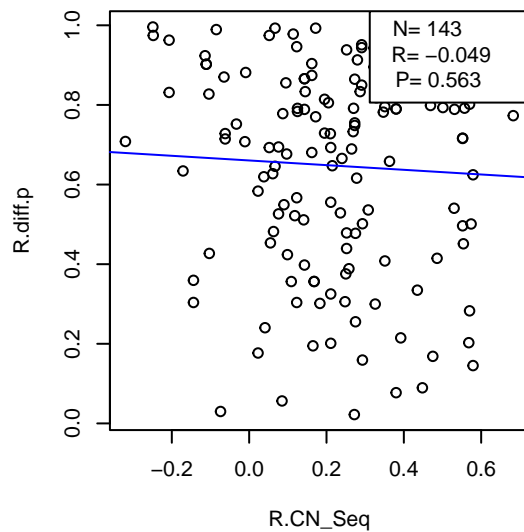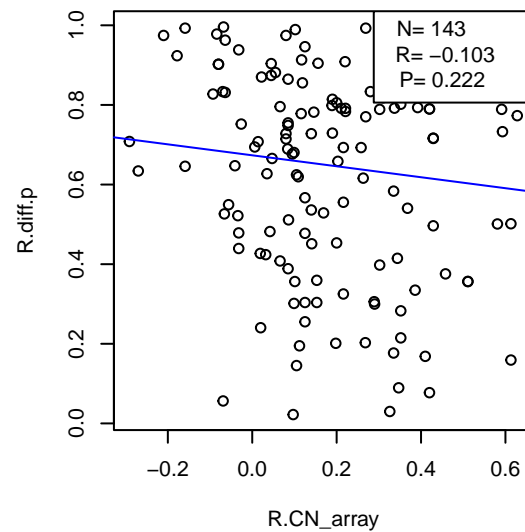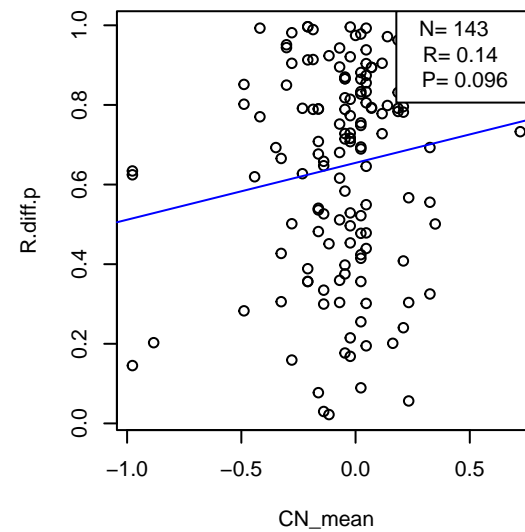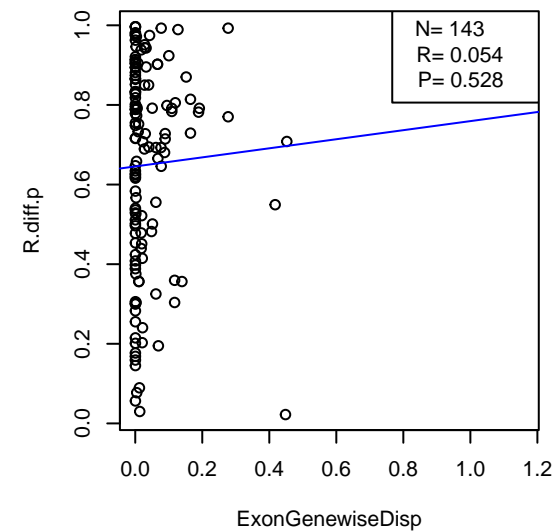

Supplement: Supplementary file 14 [file DataSheet10.PDF]

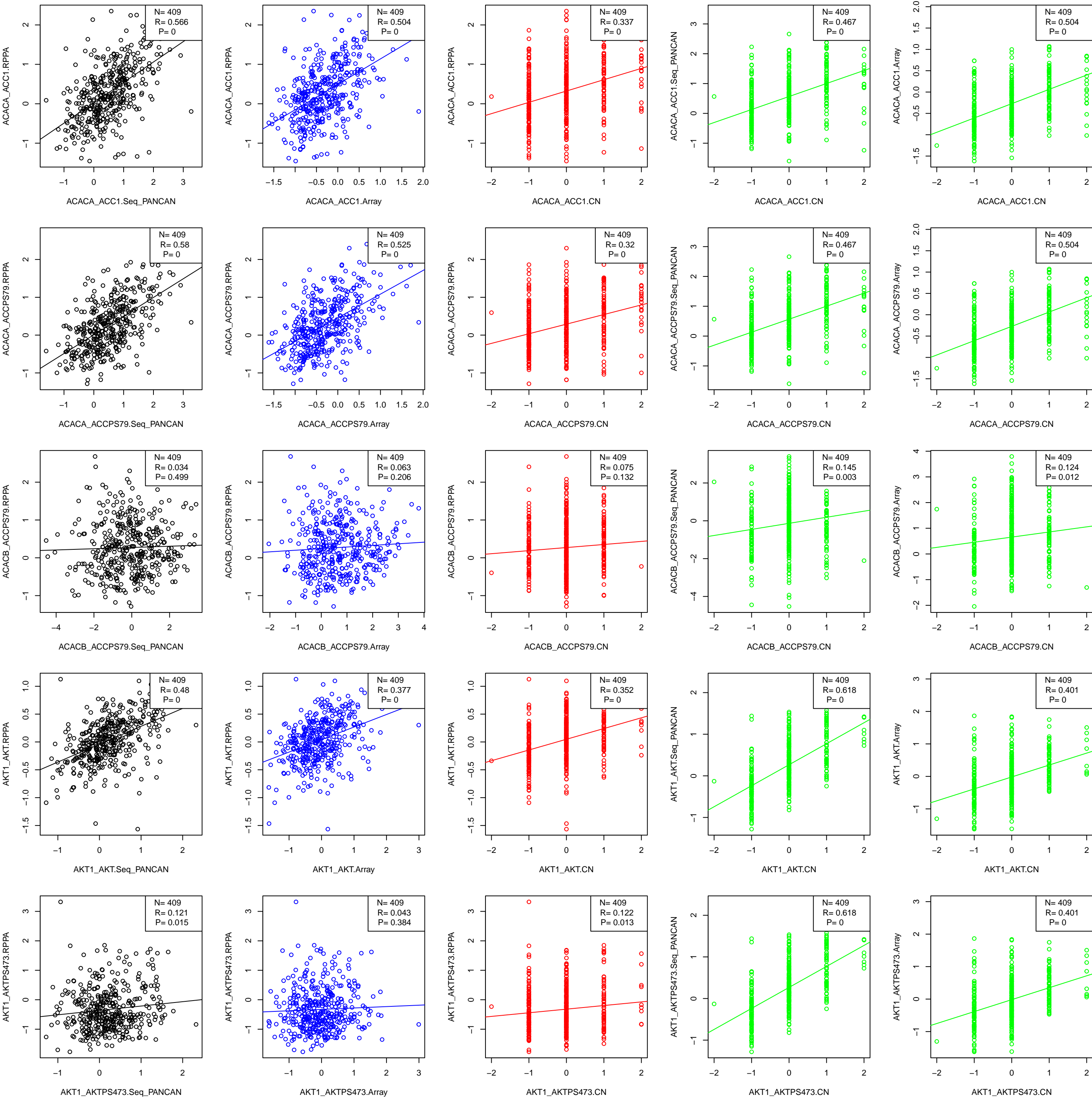

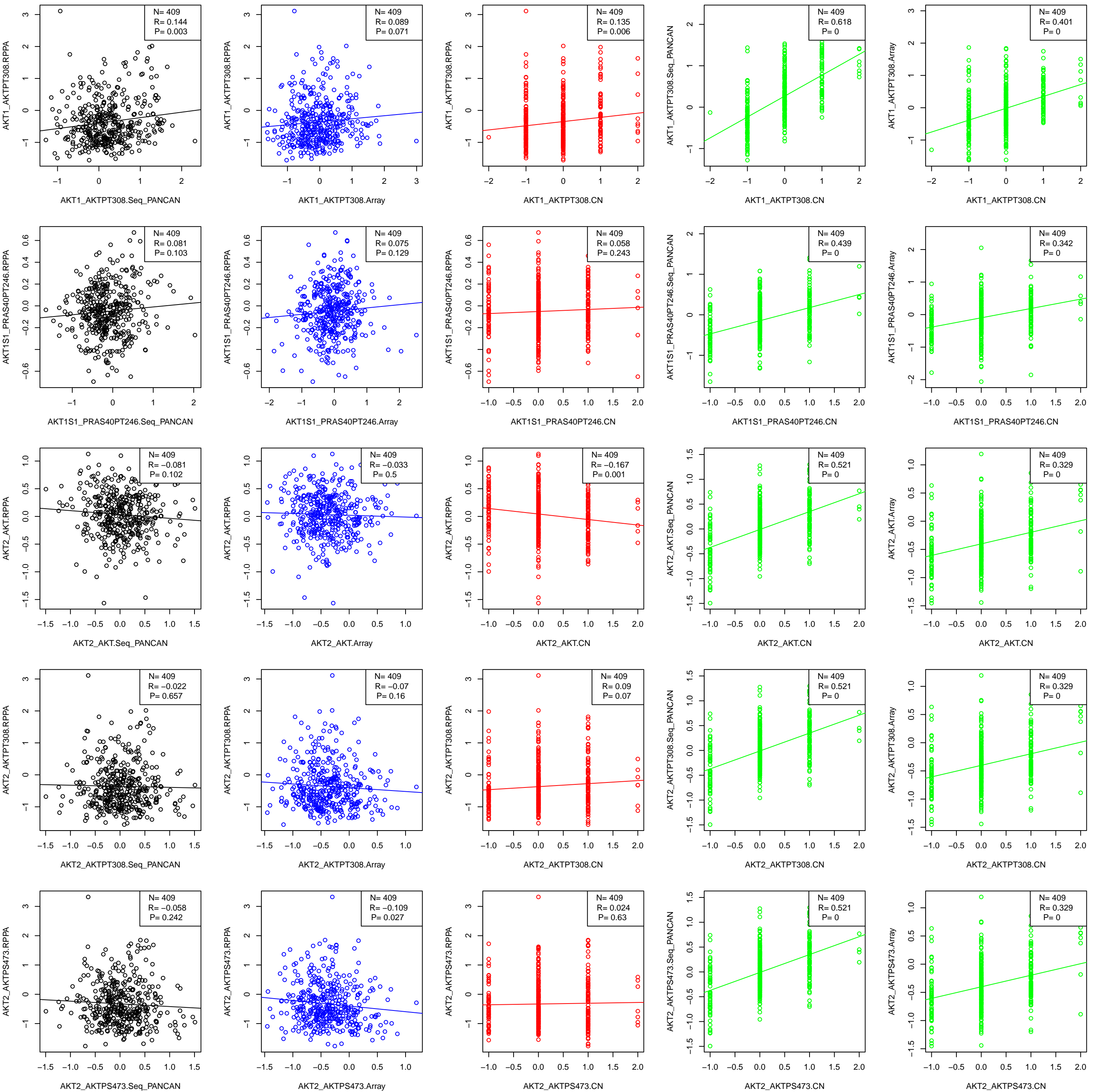

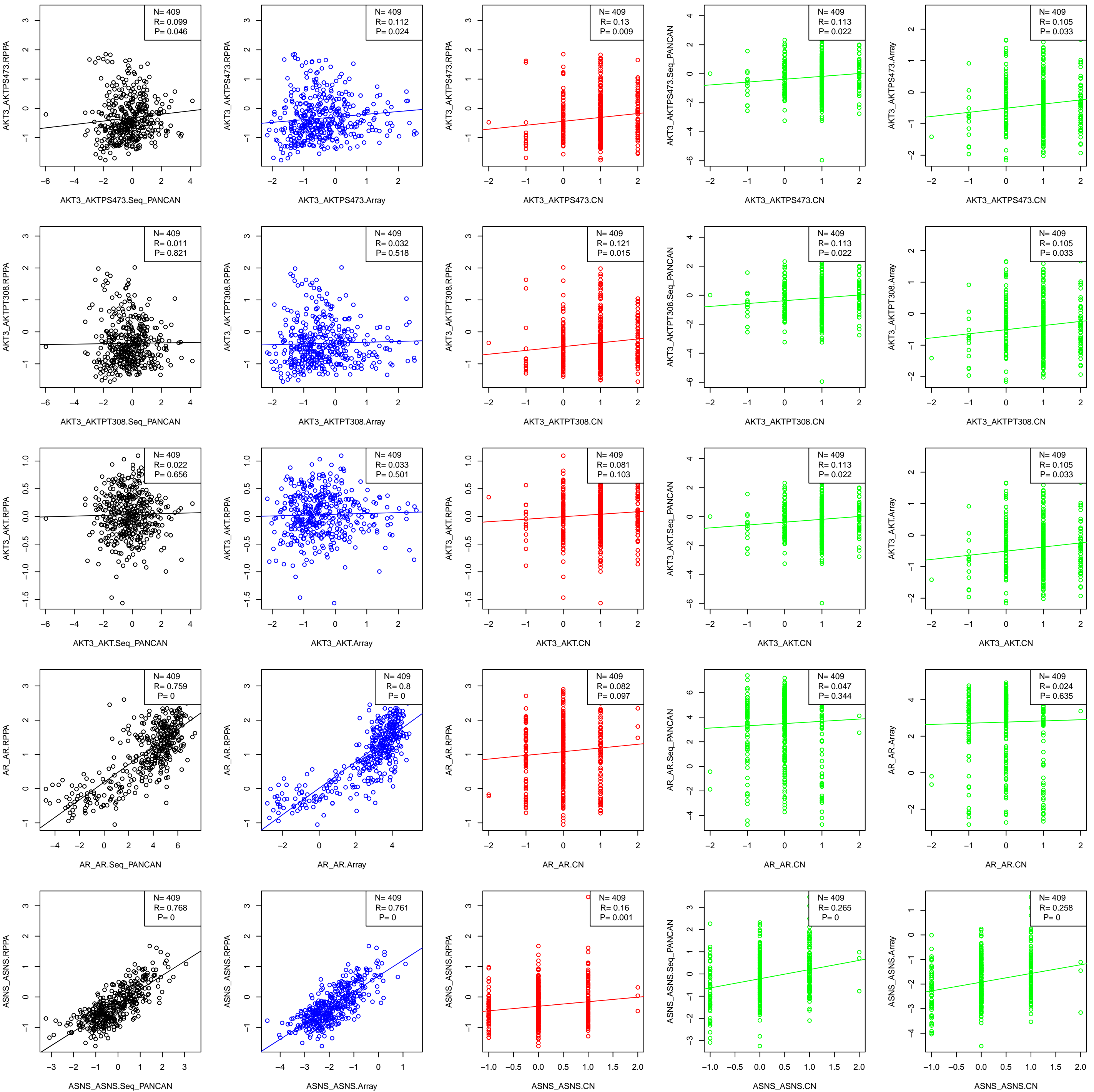

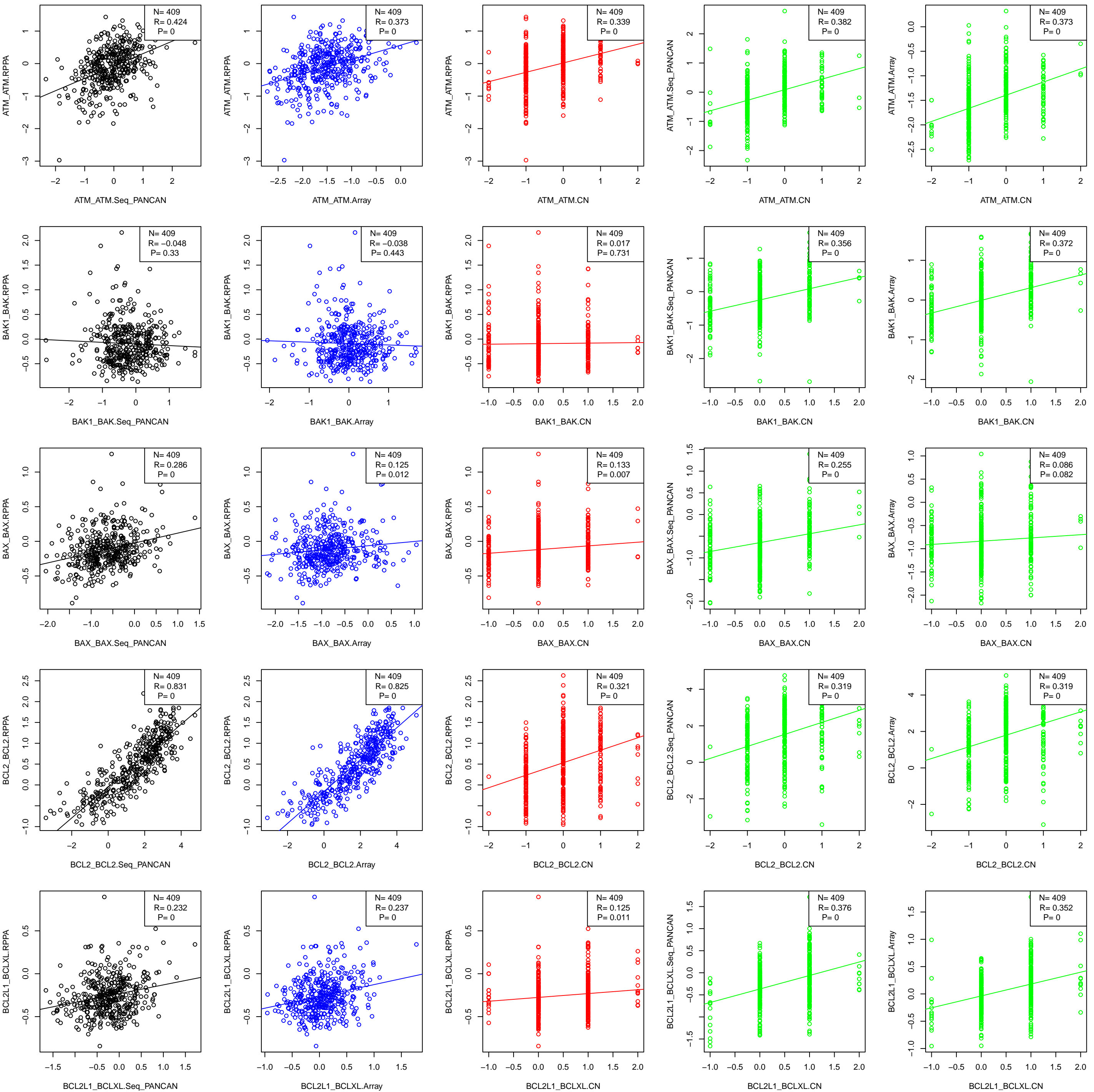

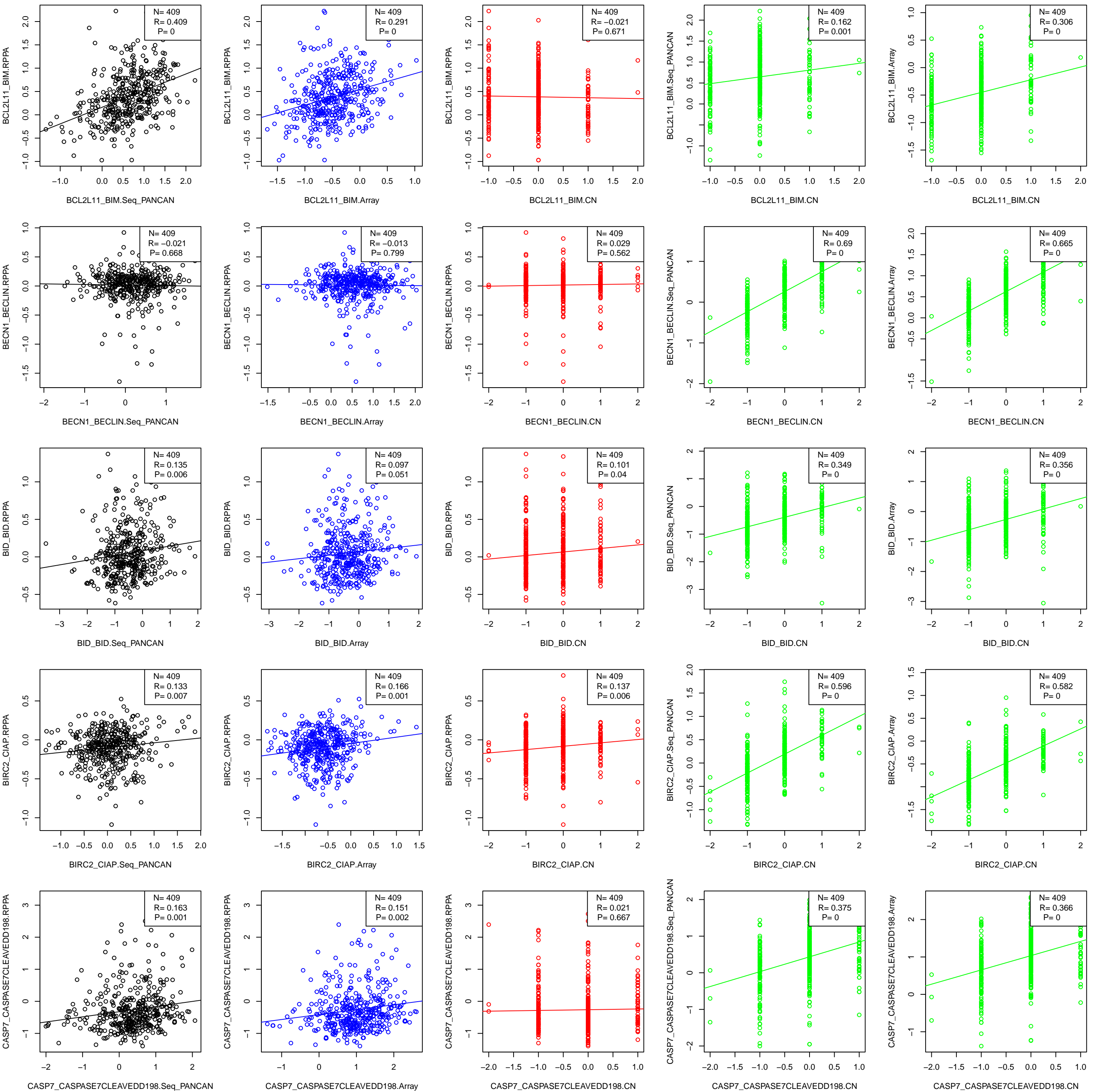

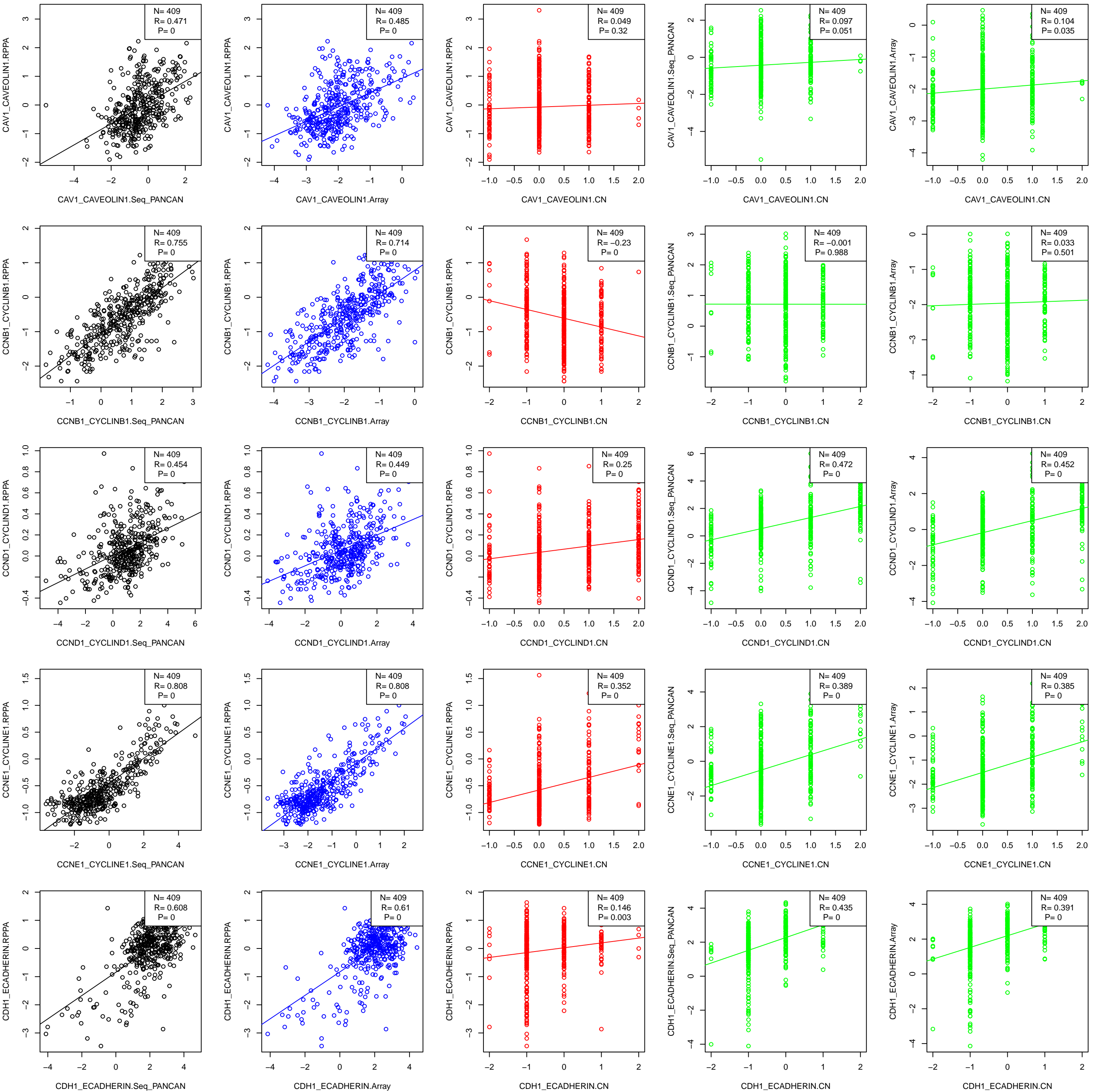

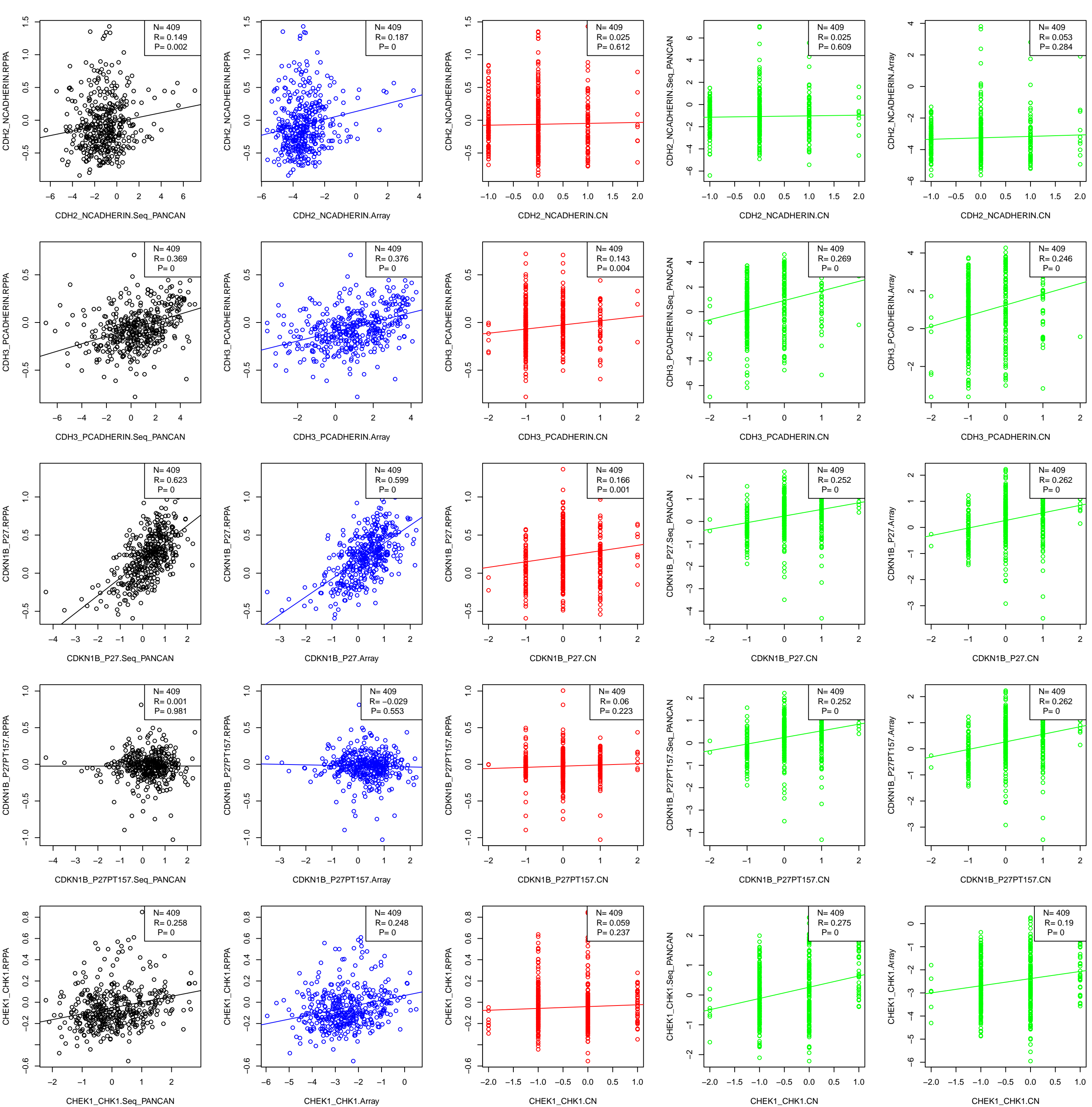

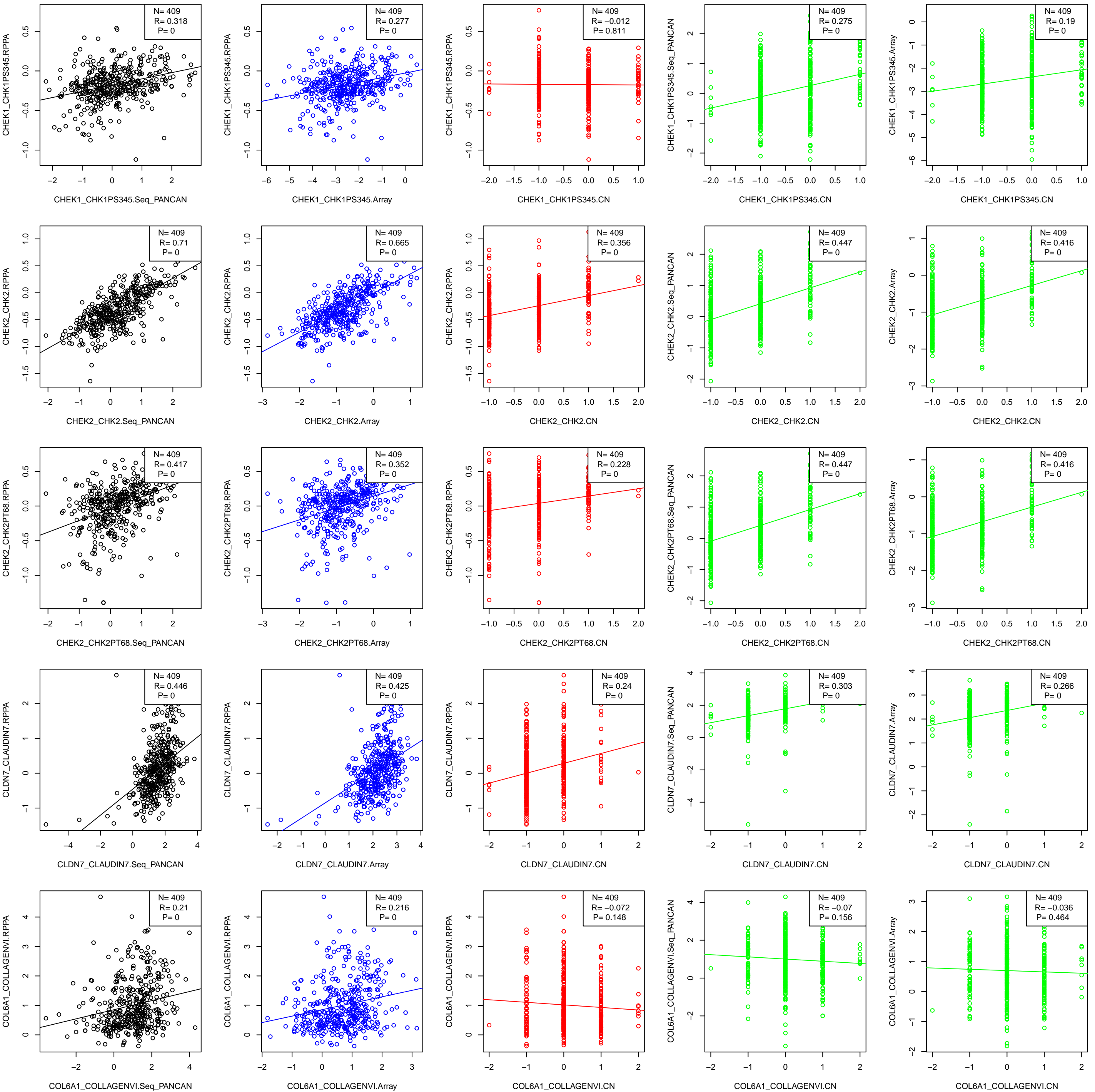

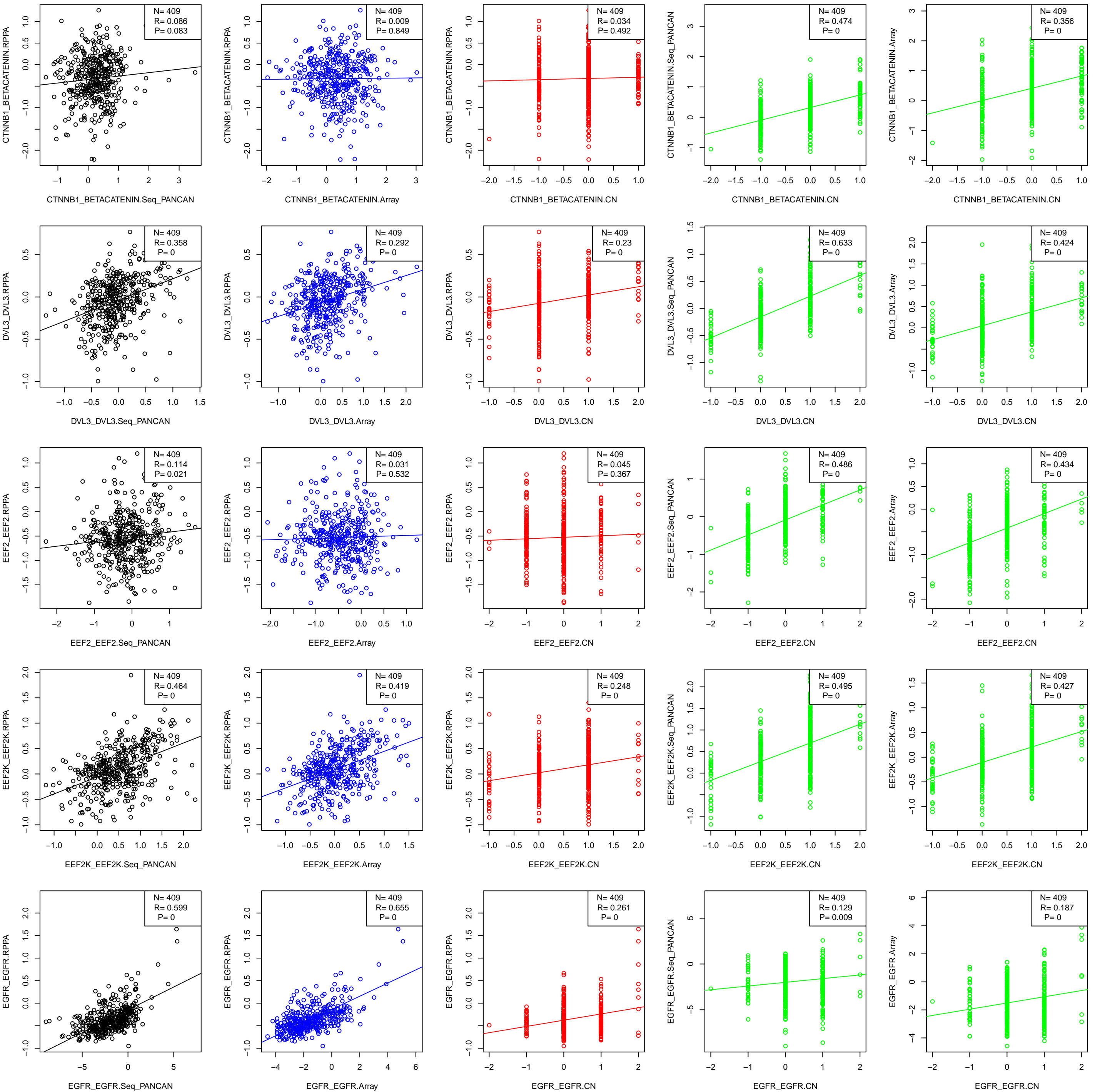

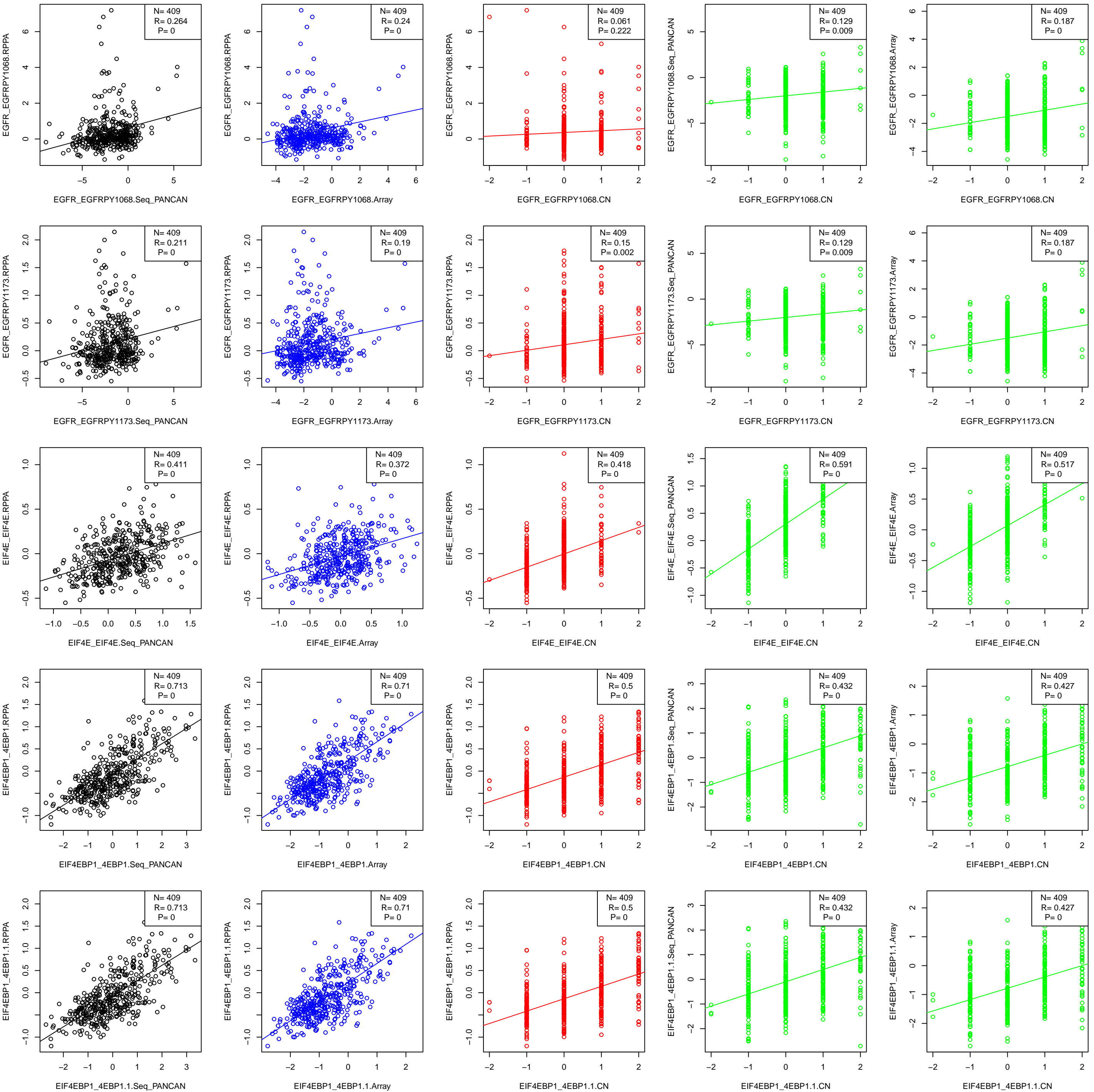

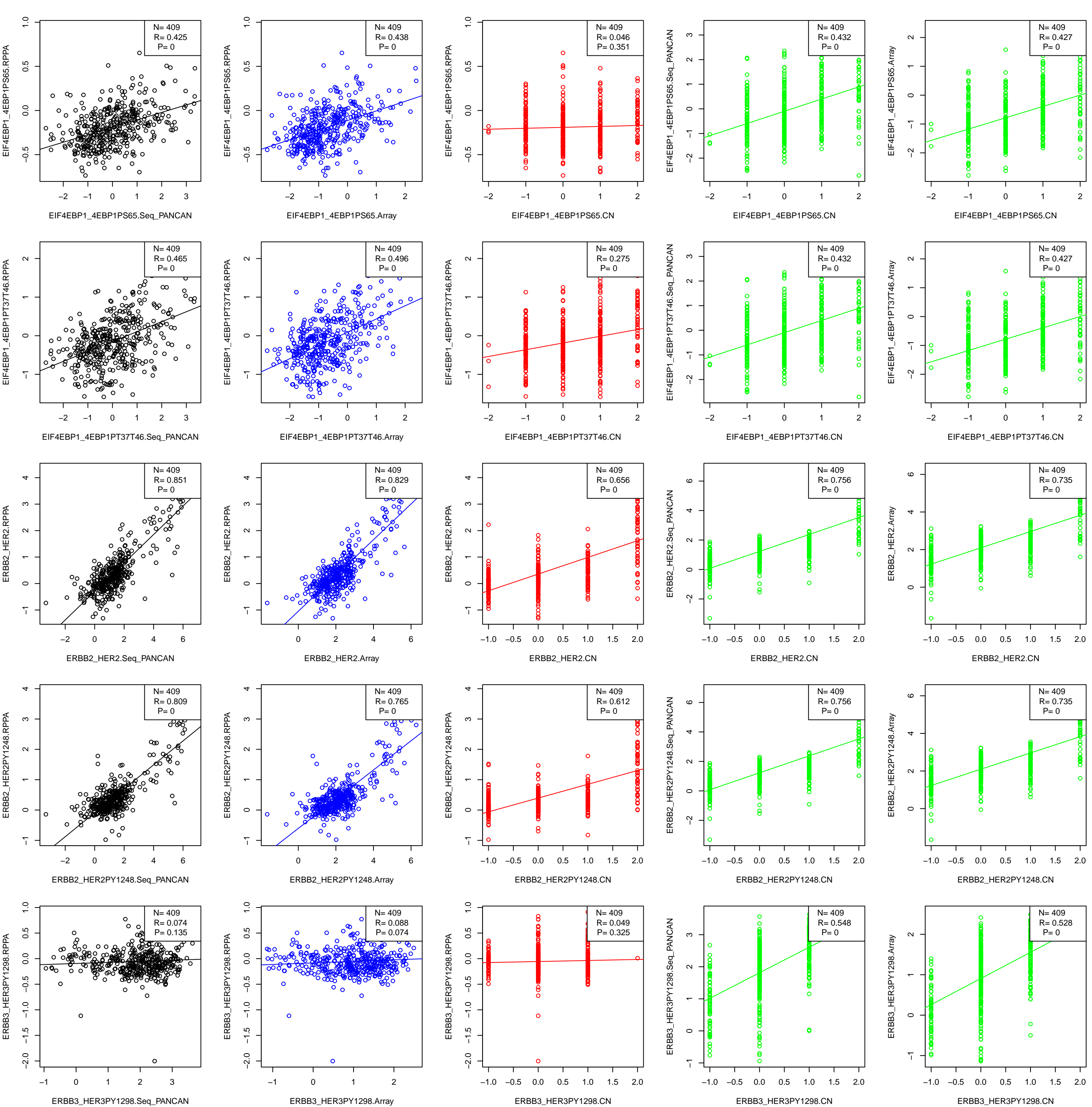

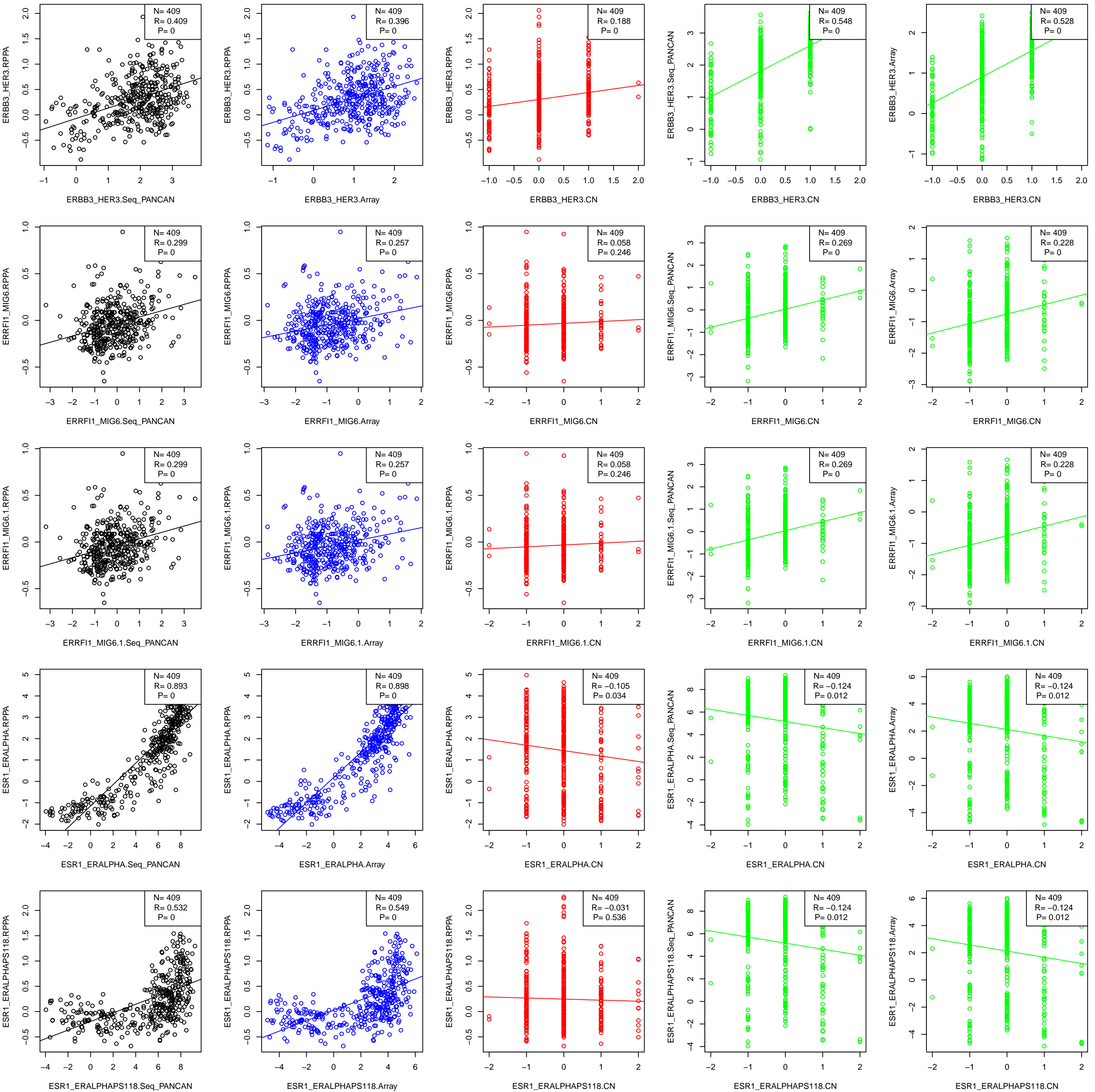

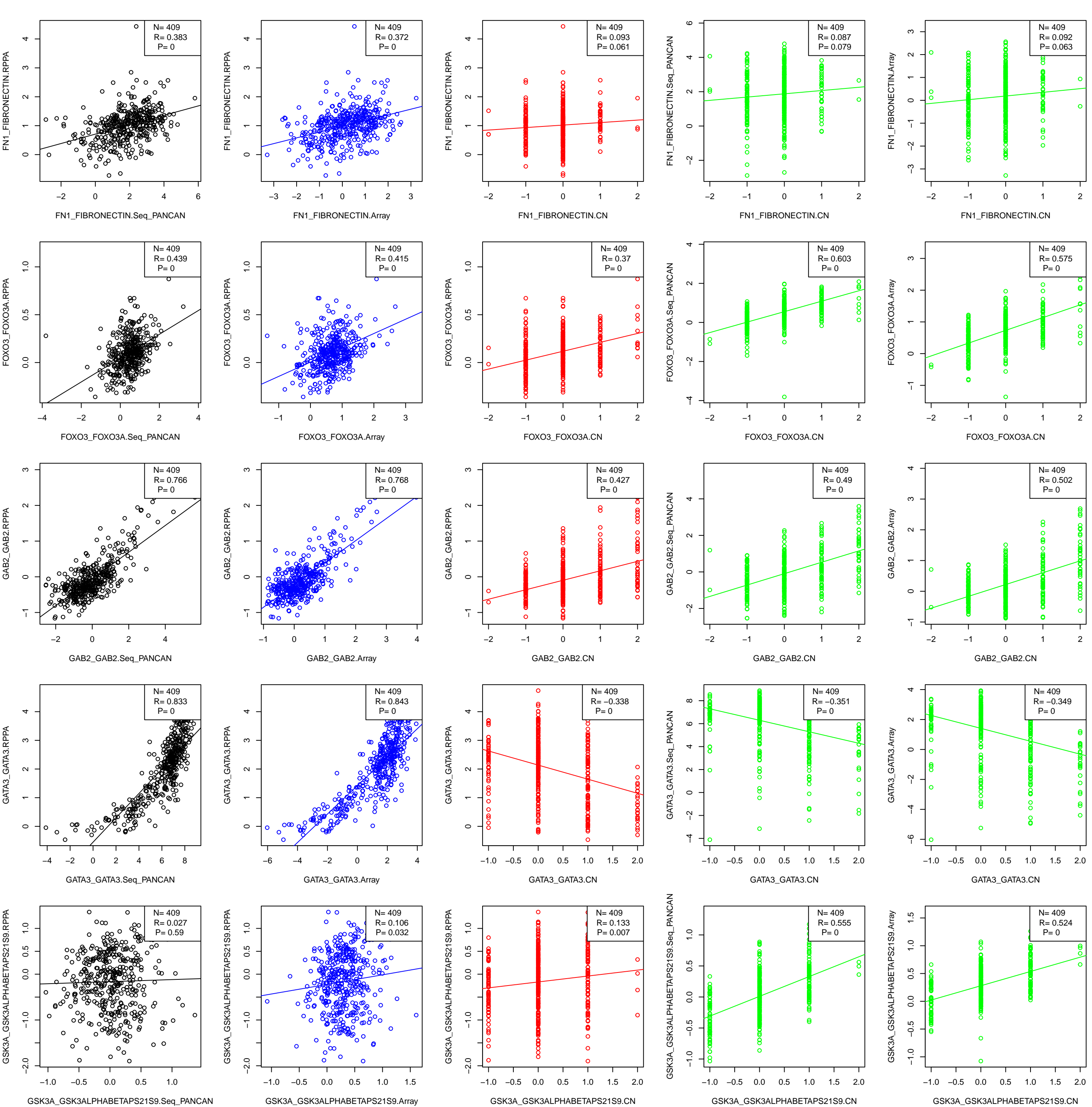

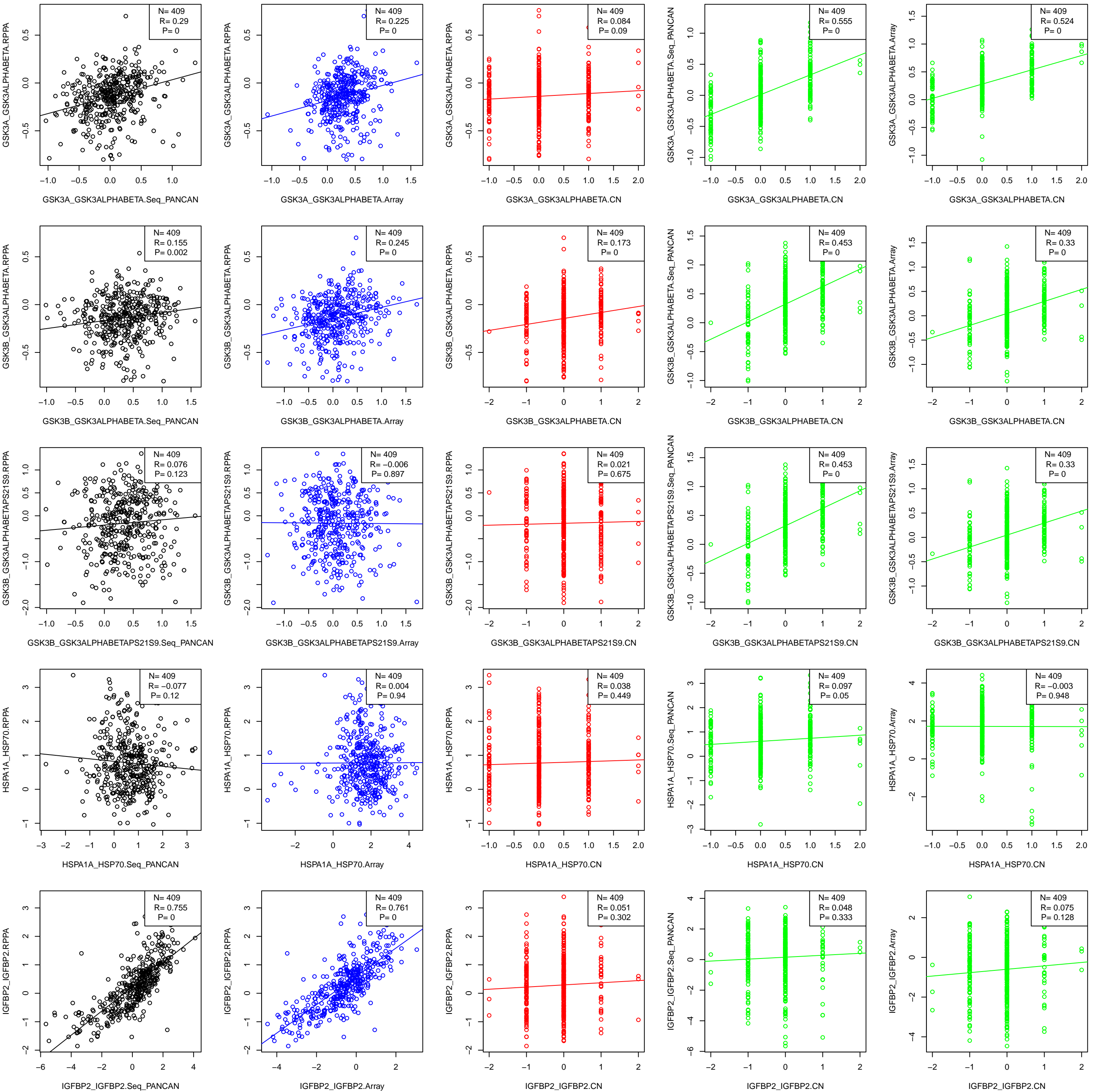

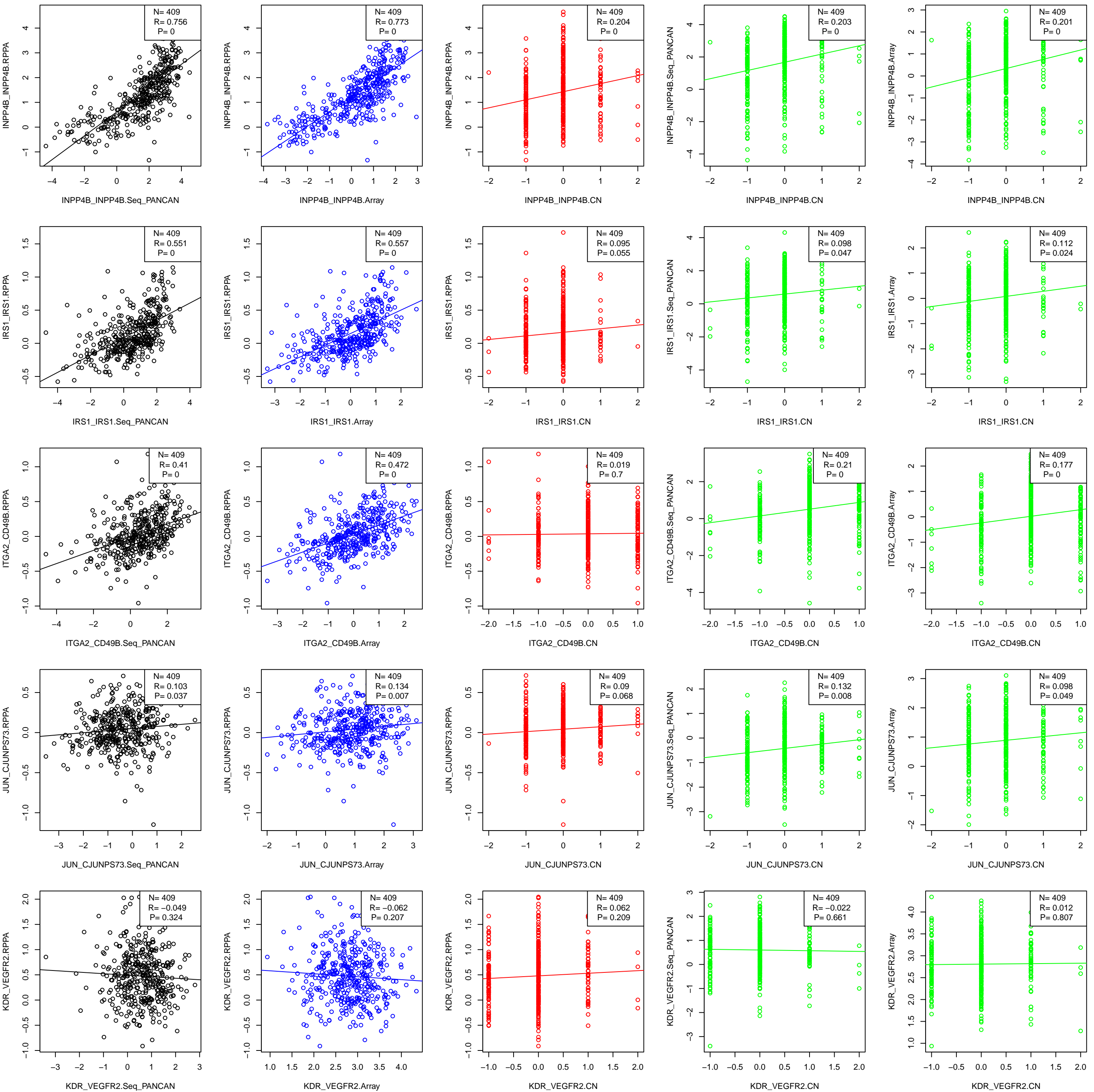

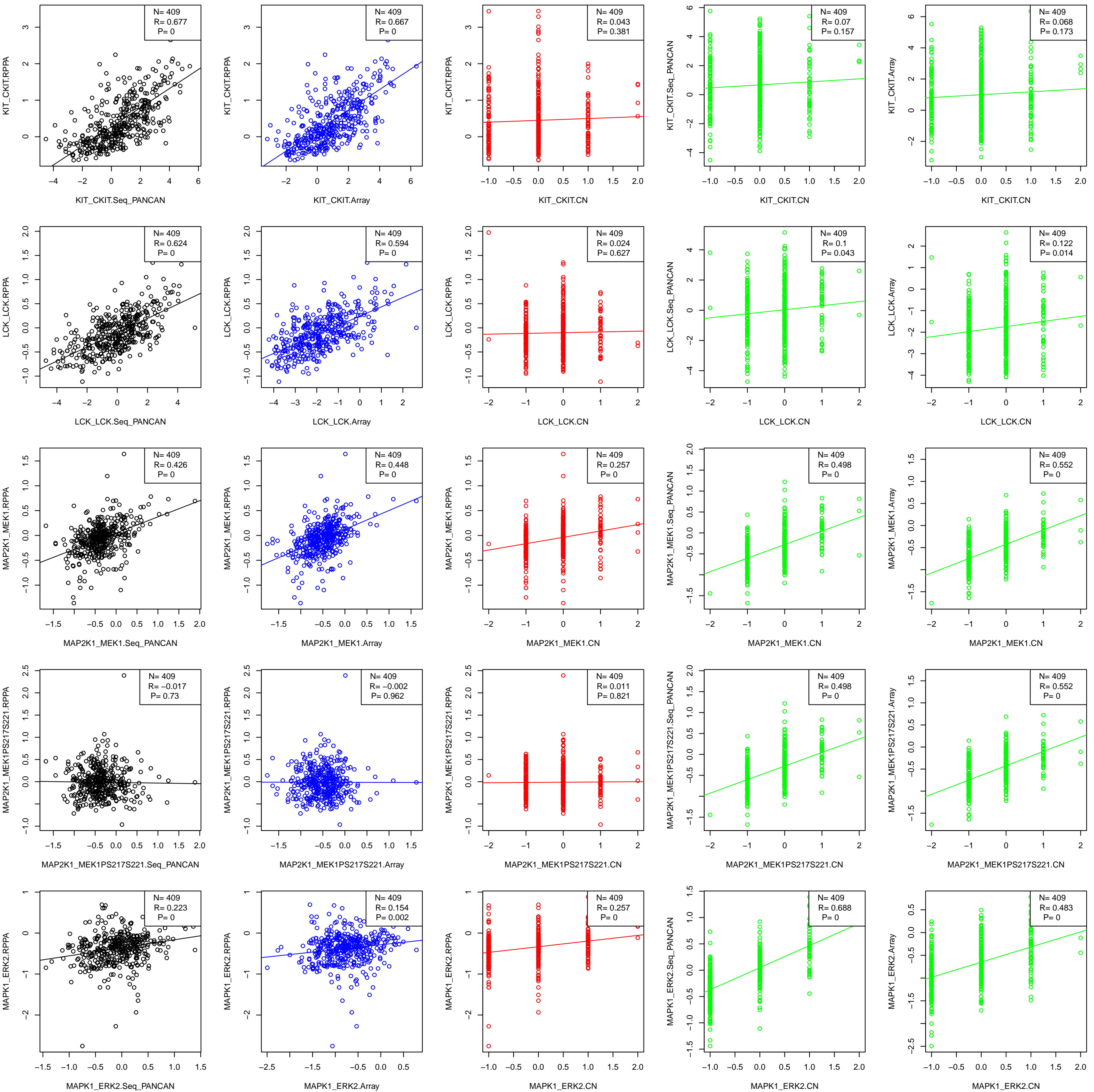

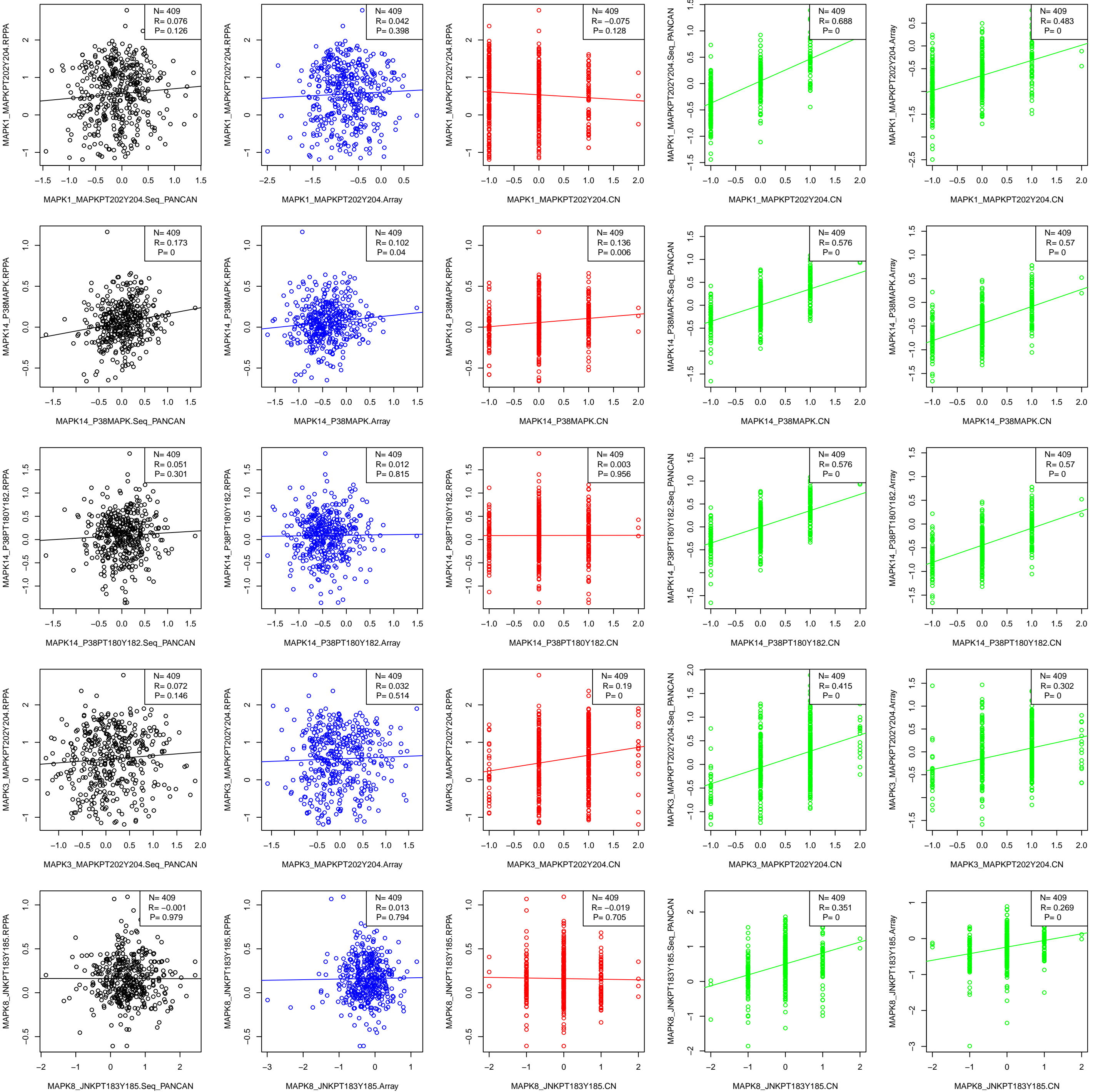

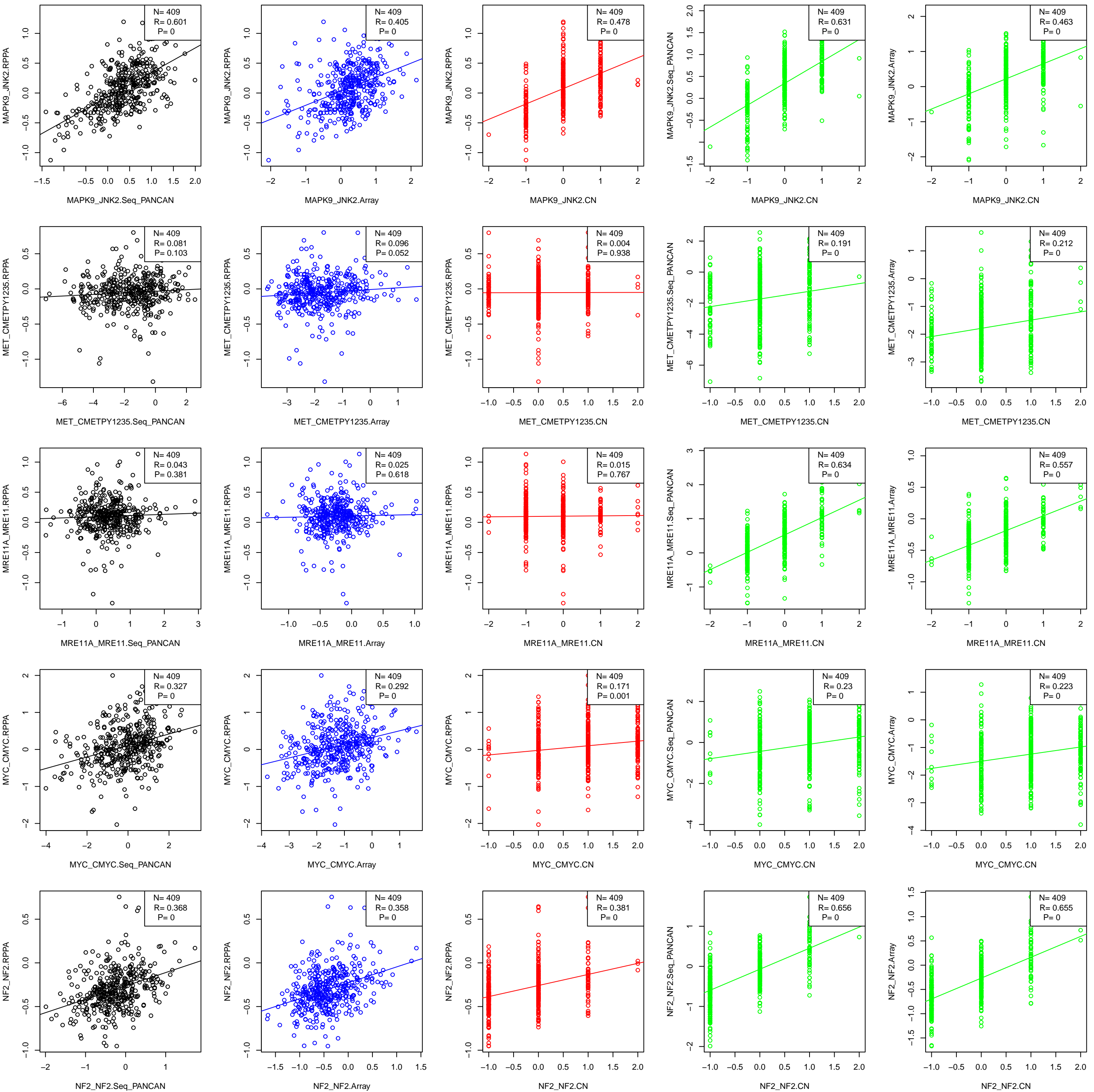

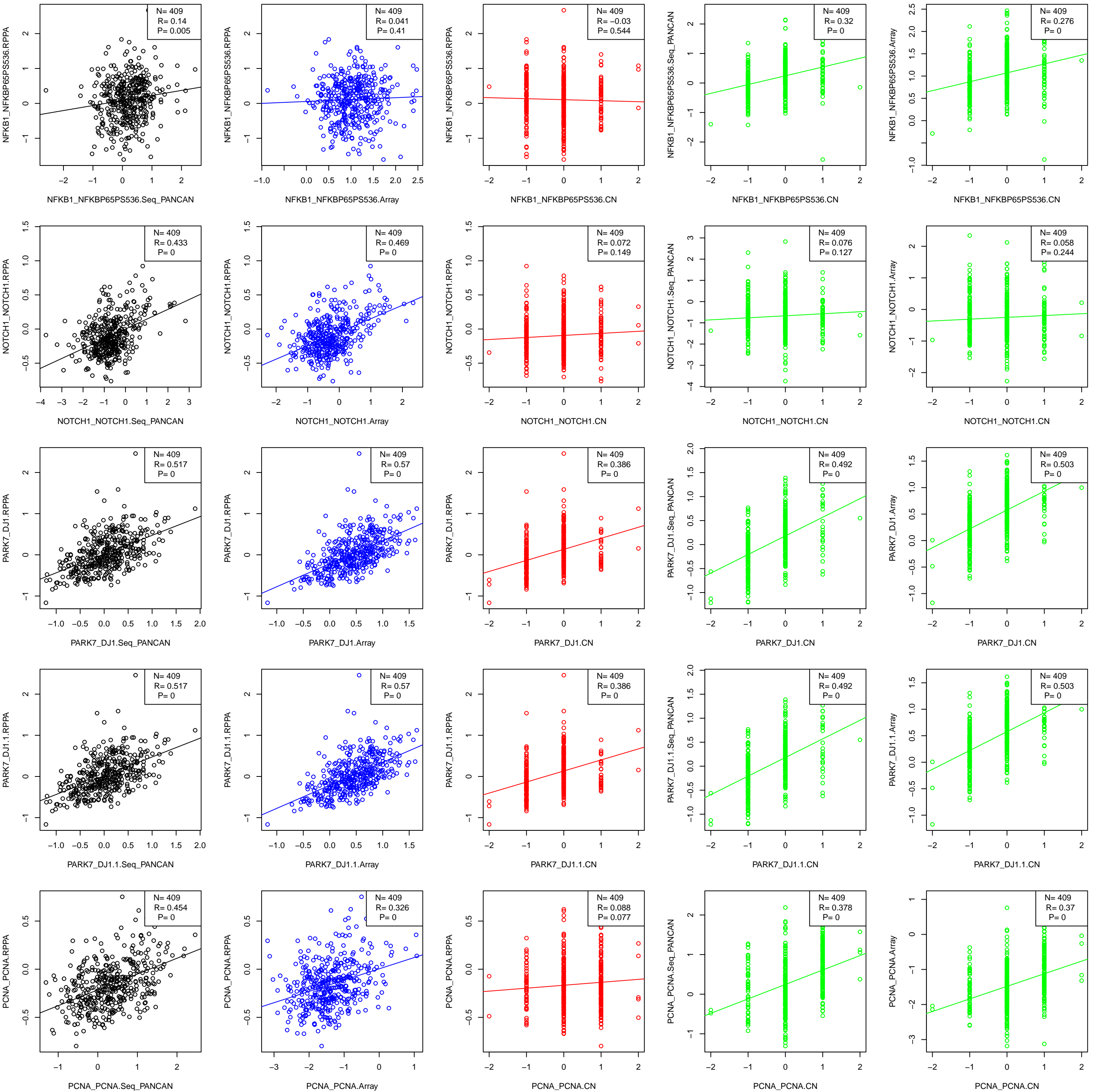

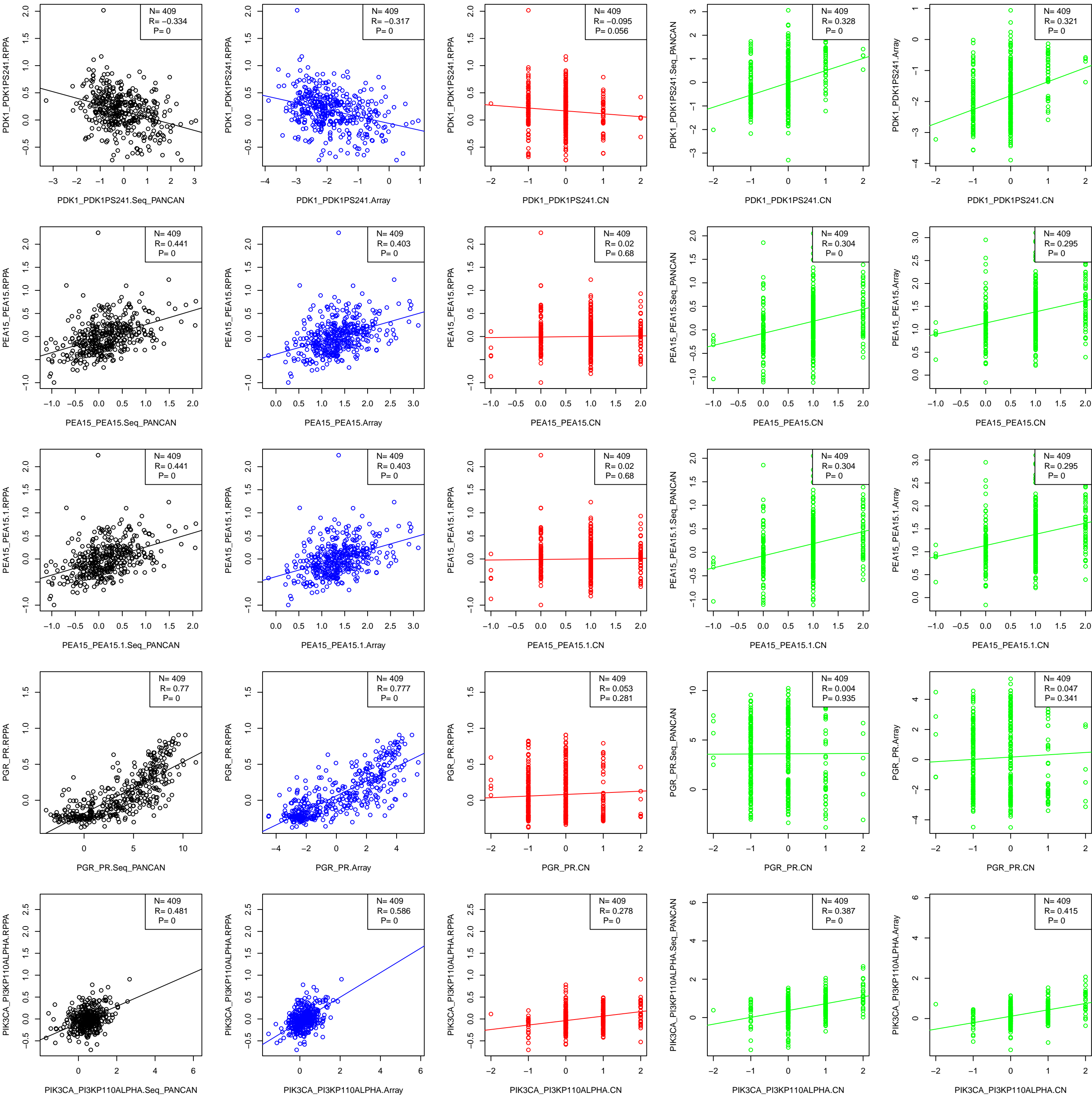

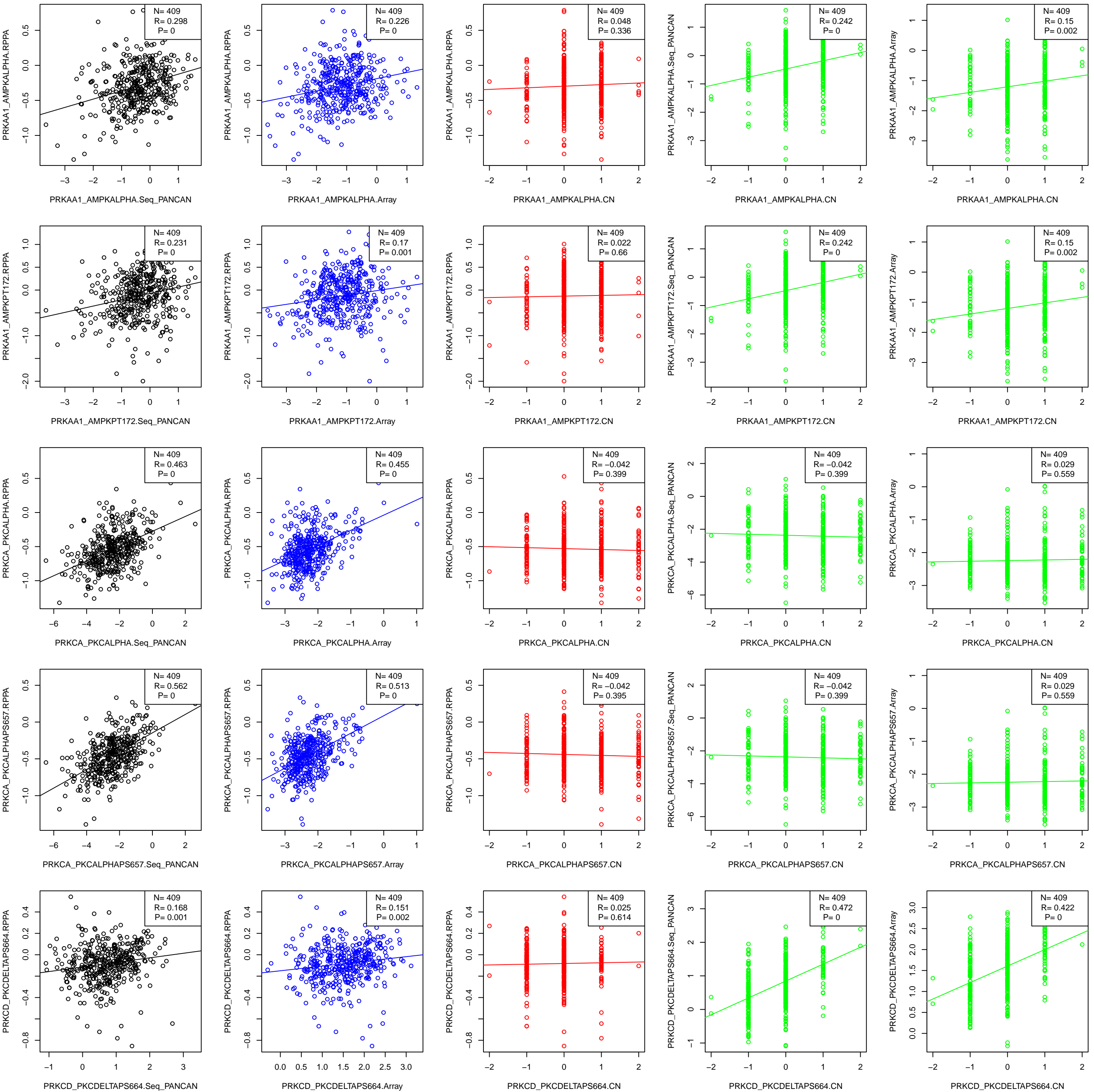

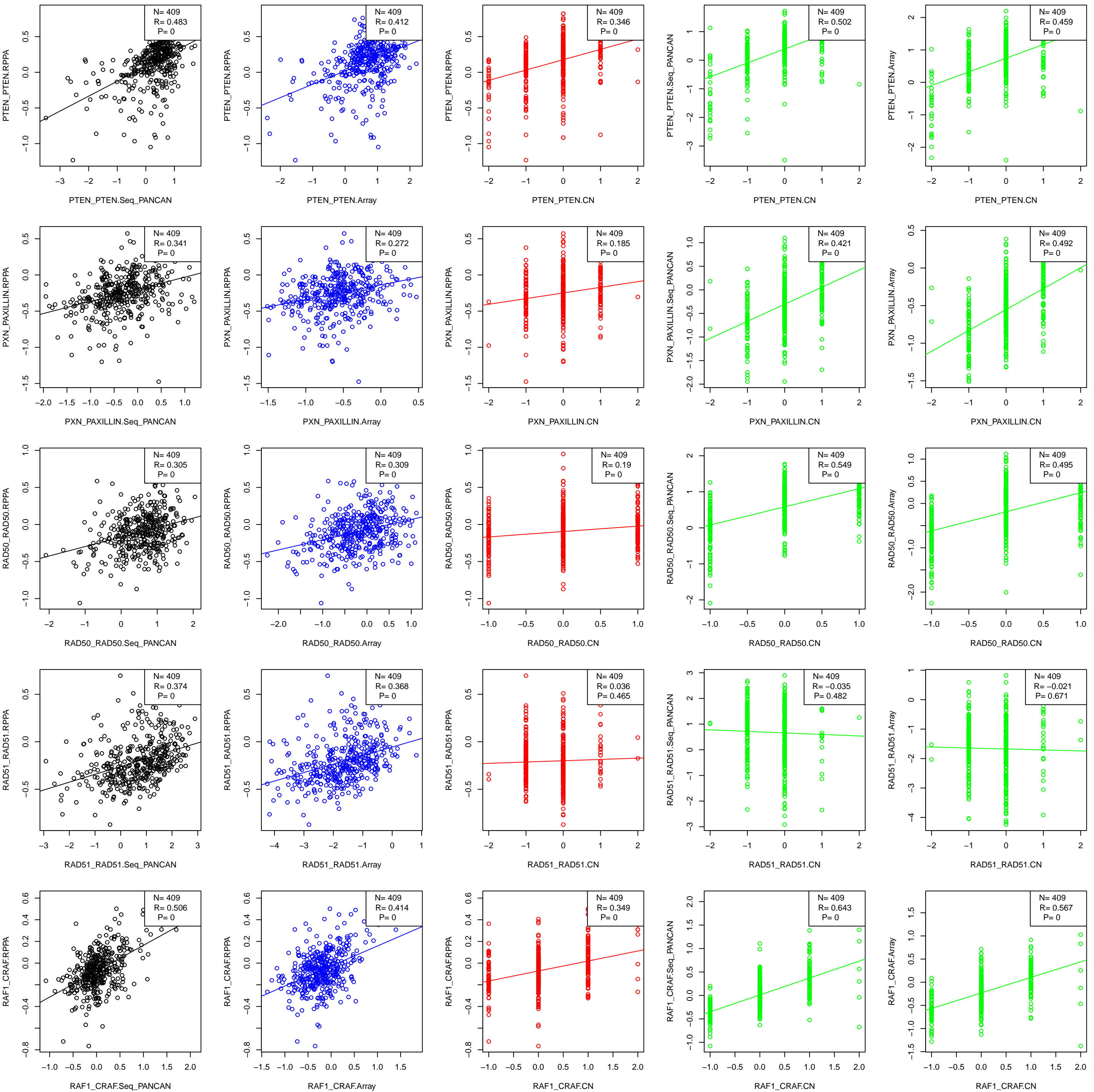

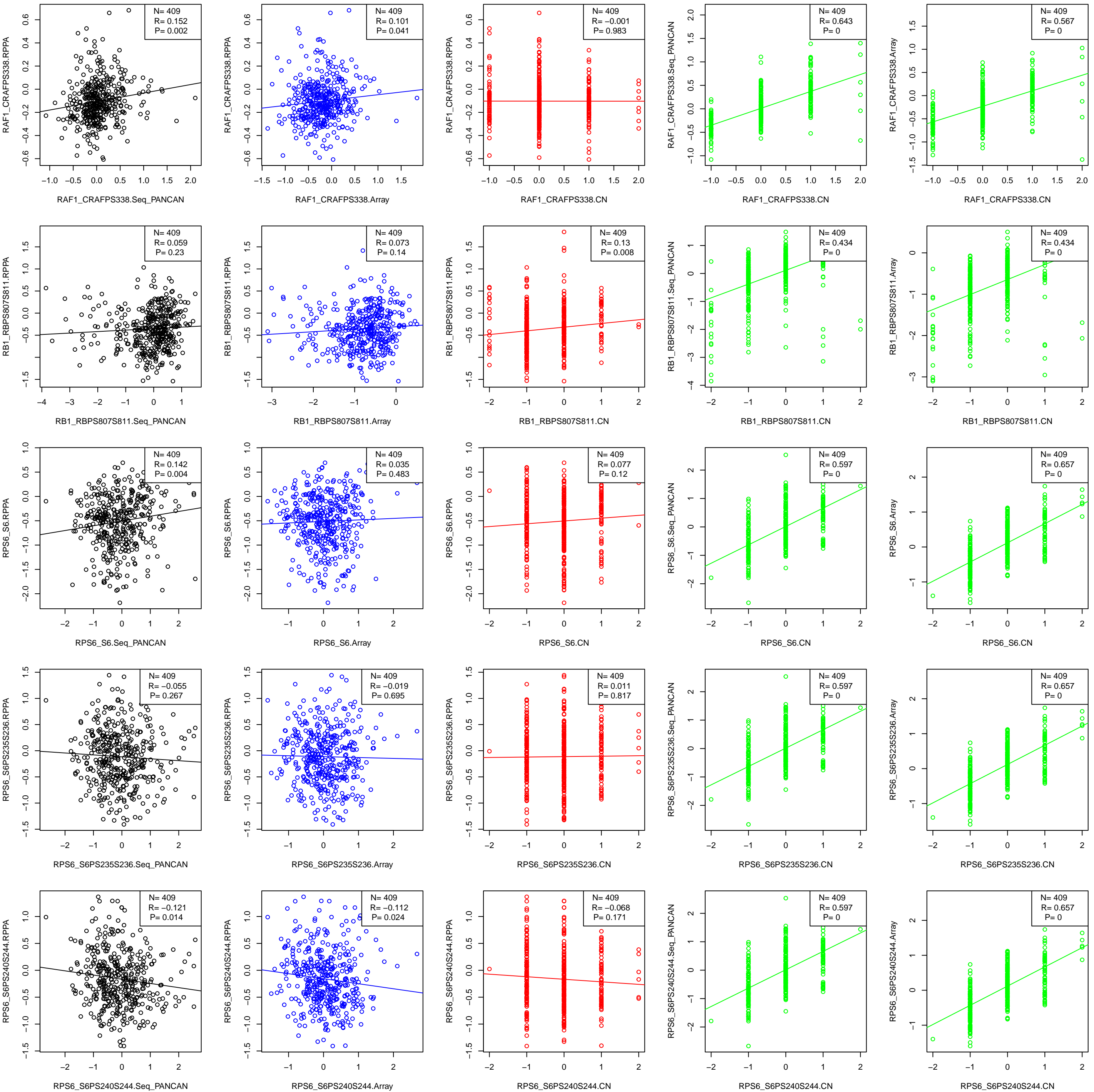

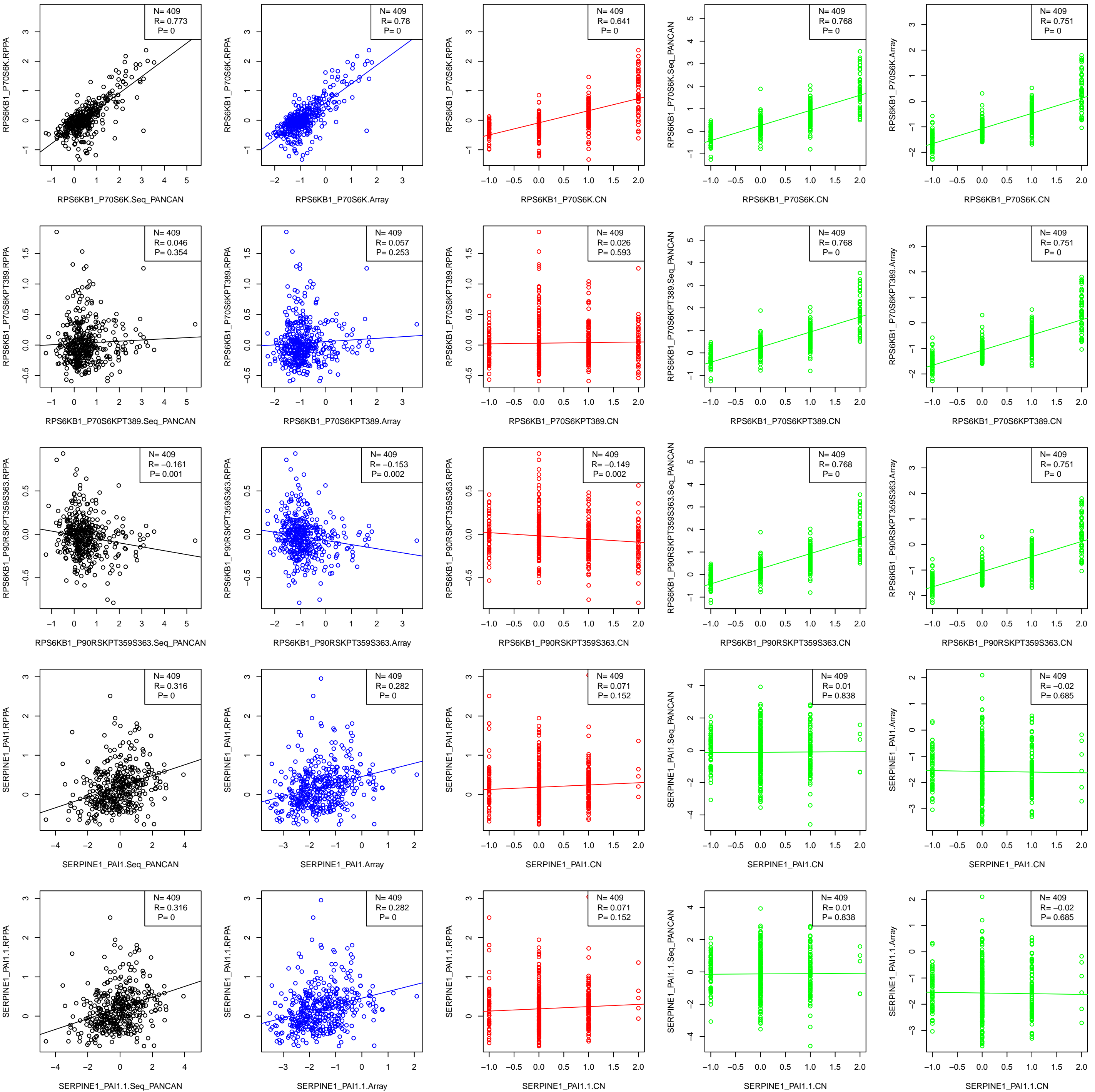

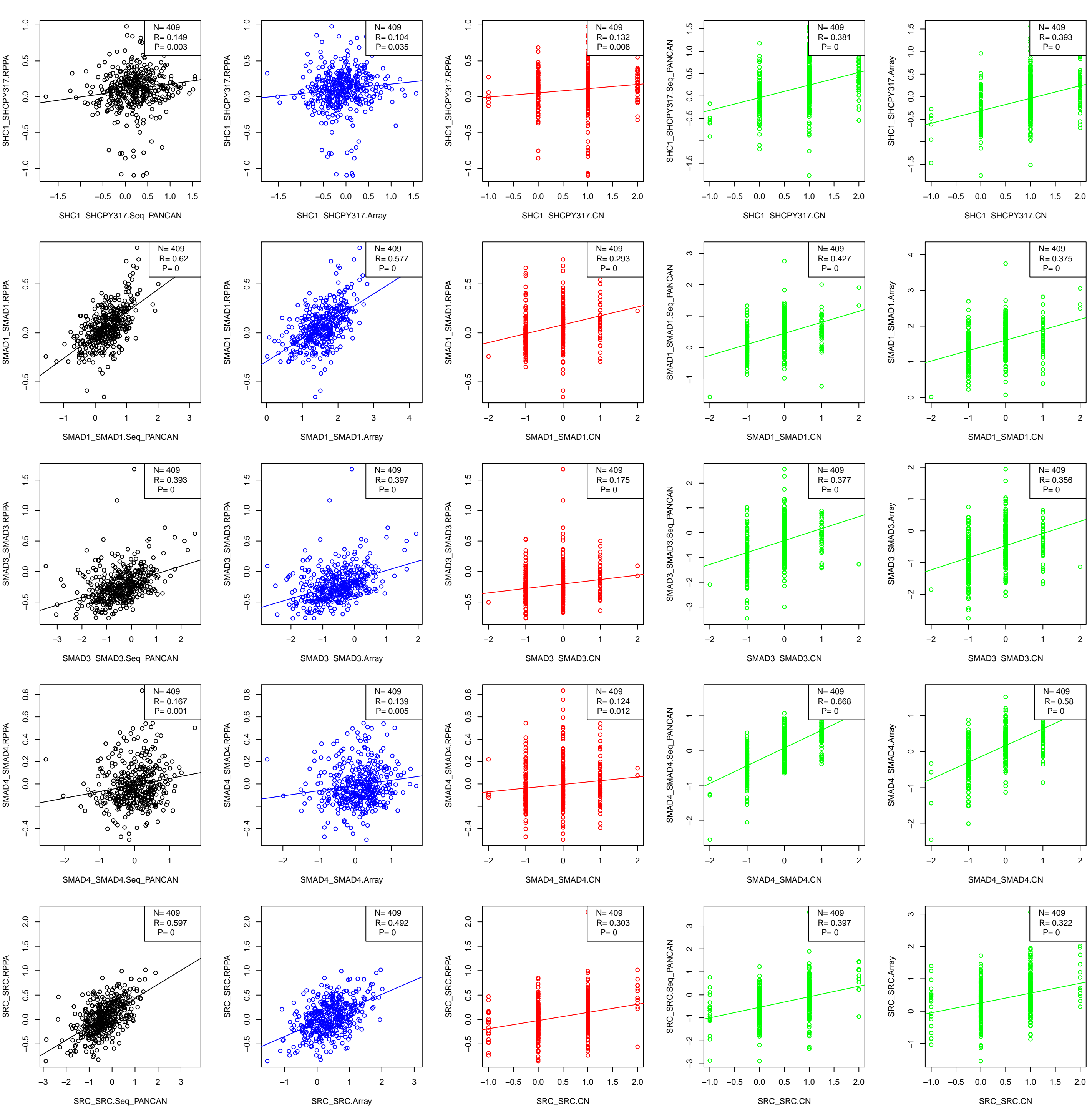

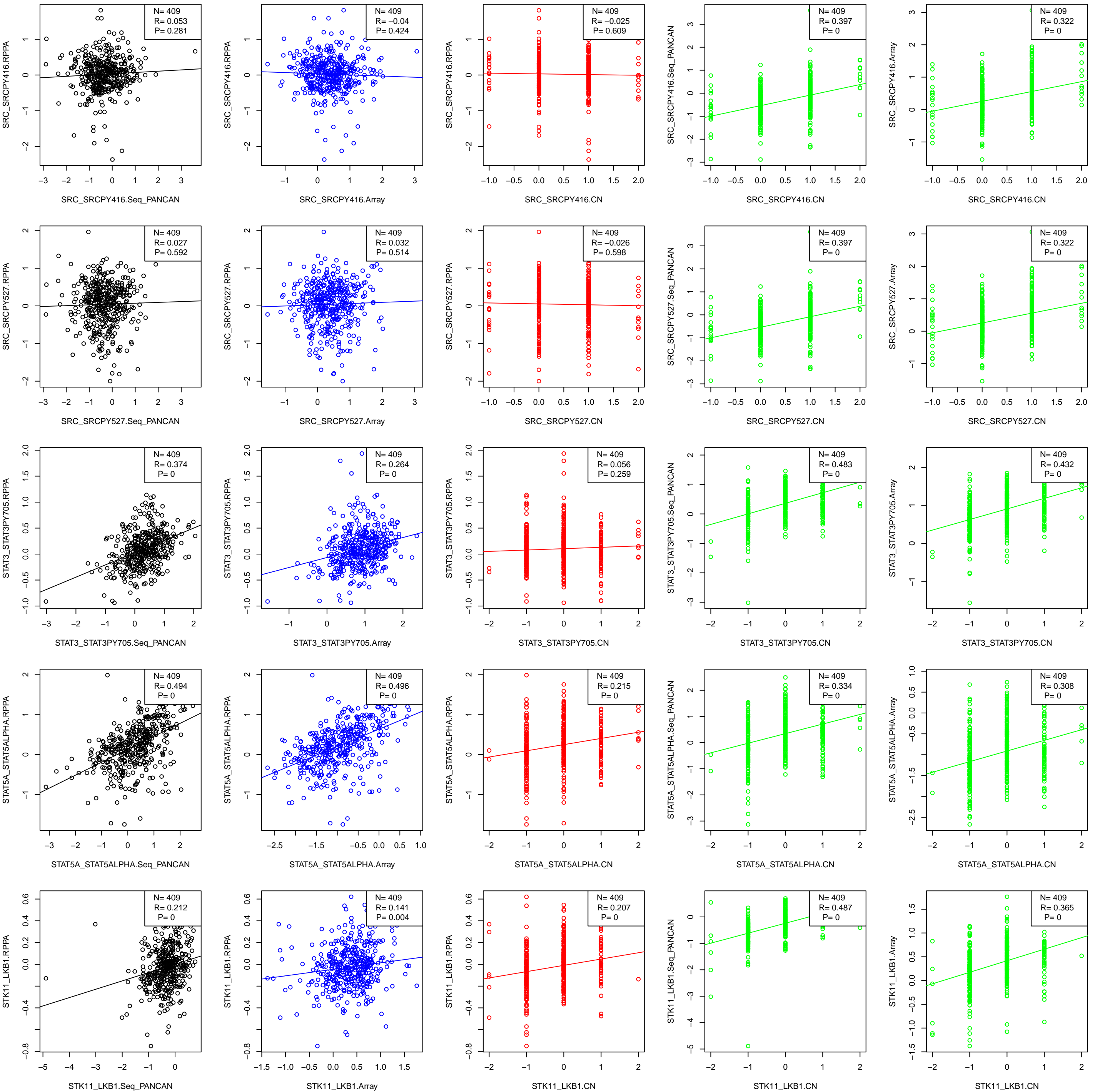

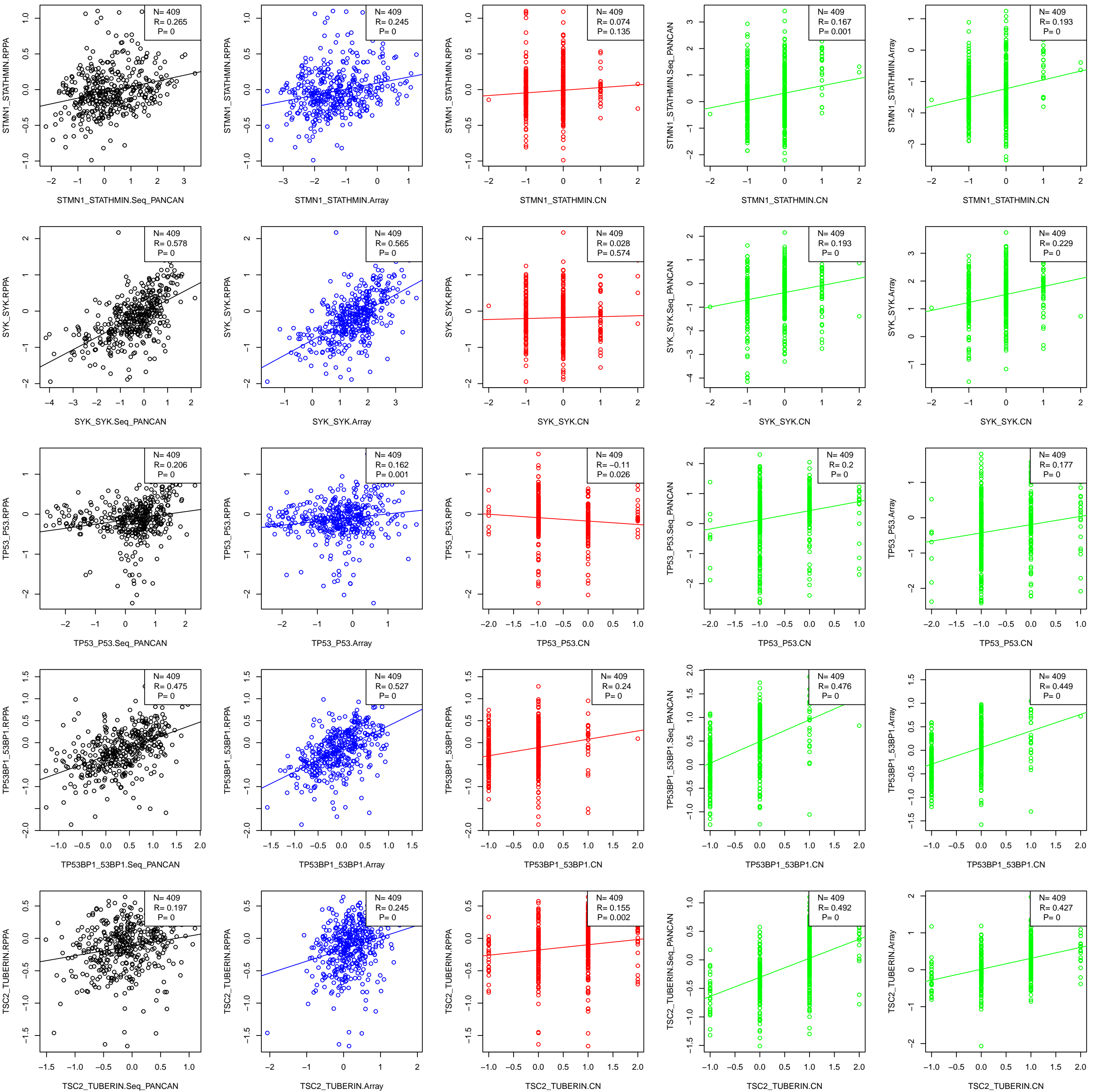

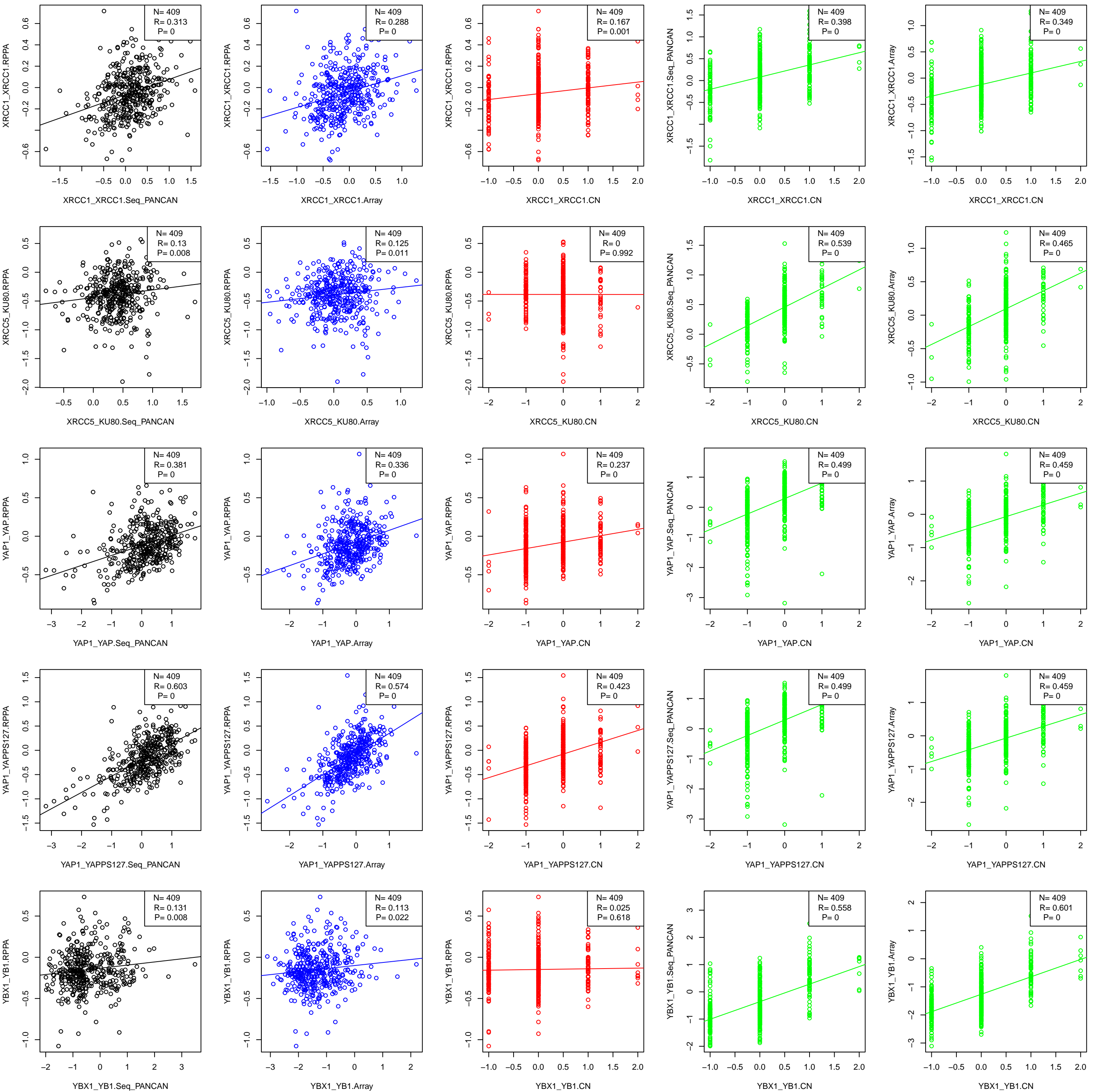

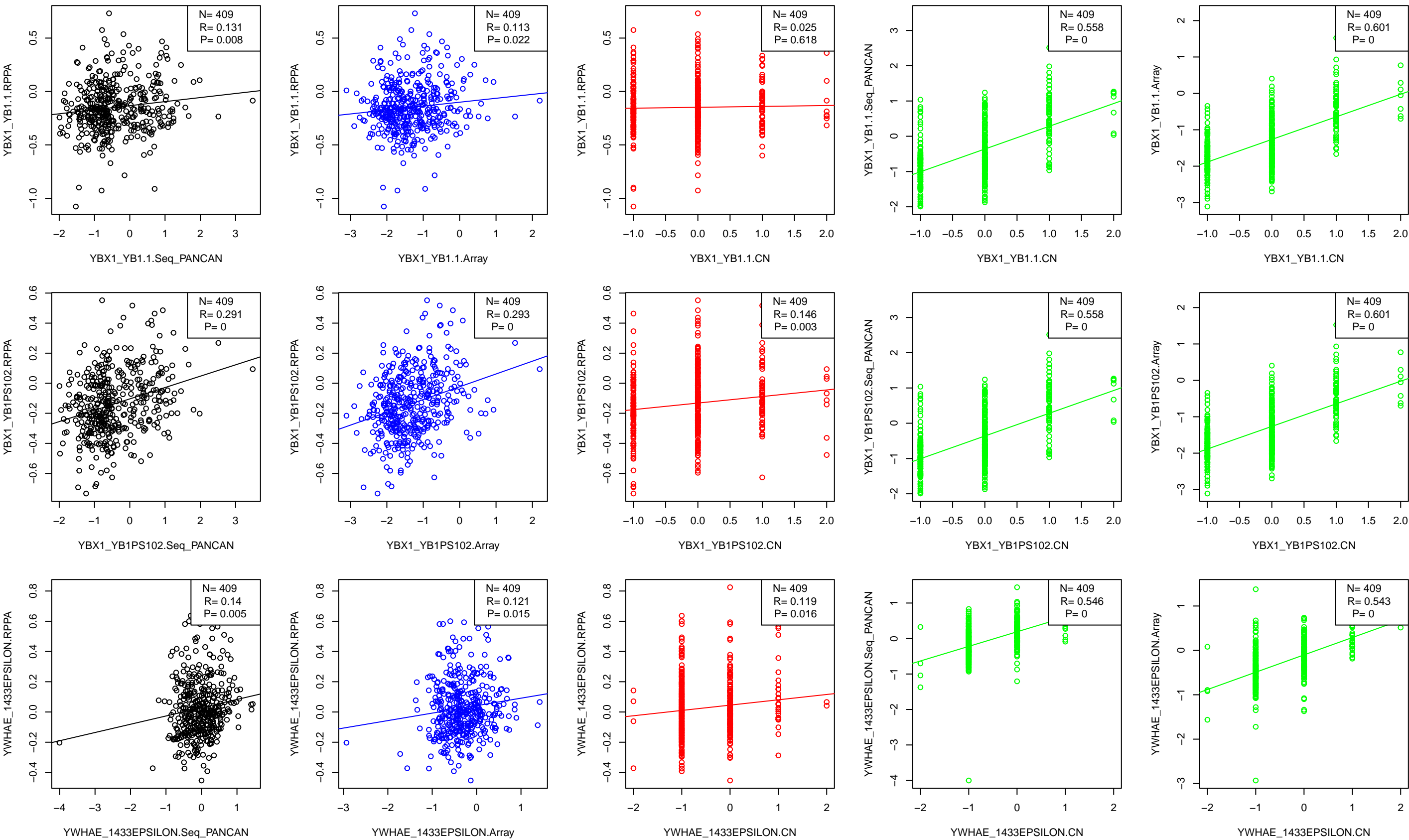

Supplement: Supplementary file 16 [file DataSheet15.PDF]
