# Supplementary material for: Comparison of RNA-Seq and microarray in the prediction of protein expression and survival prediction
Source: Front Genet. 2024 Feb 23;15:1342021. doi: 10.3389/fgene.2024.1342021 (PMC10920353; doi:10.3389/fgene.2024.1342021)
Supplement: Supplementary file 8 [file Table1.DOCX]

**Supplementary Table S1 Top 103 survival-related genes and their characteristics.**

| **Cancer** | **Gene** | **Chromosome location** | **Exon count** | **Nucleic acid count** | **Protein** | **Amino acid count** |
| --- | --- | --- | --- | --- | --- | --- |
| **BRCA**  **COAD KIRC**  **LUSC**  **OV**  **UCEC** | *ACACA* | 17q12 | 66 | 415234 | Acetyl-CoA carboxylase 1 | 2346 |
|  | *ACACB* | 12q24.11 | 59 | 221095 | Acetyl-CoA carboxylase 2 | 2458 |
|  | *AKT1* | 14q32.33 | 17 | 33627 | RAC-alpha serine/threonine-protein kinase | 480 |
|  | *AKT1S1* | 19q13.33 | 7 | 68300 | Proline-rich AKT1 substrate 1 | 256 |
|  | *AKT2* | 19q13.2 | 14 | 126319 | RAC-beta serine/threonine-protein kinase | 481 |
|  | *AKT3* | 1q43–q44 | 25 | 791276 | RAC-gamma serine/threonine-protein kinase | 479 |
|  | *AR* | Xq12 | 11 | 419344 | Androgen receptor | 920 |
|  | *ASNS* | 7q21.3 | 16 | 241113 | Asparagine synthetase [glutamine-hydrolyzing] | 561 |
|  | *ATM* | 11q22.3 | 67 | 312666 | Serine-protein kinase ATM | 3056 |
|  | *BAK1* | 6p21.31 | 9 | 76410 | Bcl-2 homologous antagonist/killer | 211 |
|  | *BAX* | 19q13.33 | 7 | 31413 | Apoptosis regulator BAX | 192 |
|  | *BCL2* | 18q21.33 | 4 | 302361 | Apoptosis regulator Bcl-2 | 239 |
|  | *BCL2L1* | 20q11.21 | 6 | 163888 | Bcl-2-like protein 1 | 233 |
|  | *BCL2L11* | 2q13 | 15 | 762588 | Bcl-2-like protein 11 | 198 |
|  | *BECN1* | 17q21.31 | 12 | 63150 | Beclin-1 | 450 |
|  | *BID* | 22q11.21 | 8 | 151632 | BH3-interacting domain death agonist | 195 |
|  | *BIRC2* | 11q22.2 | 9 | 146143 | Baculoviral IAP repeat-containing protein 2 | 618 |
|  | *CASP7* | 10q25.3 | 12 | 194715 | Caspase-7 | 303 |
|  | *CAV1* | 7q31.2 | 4 | 188289 | Caveolin-1 | 178 |
|  | *CCNB1* | 5q13.2 | 9 | 116343 | G2/mitotic-specific cyclin-B1 | 433 |
|  | *CCND1* | 11q13.3 | 5 | 20434 | G1/S-specific cyclin-D1 | 295 |
|  | *CCNE1* | 19q12 | 12 | 102225 | G1/S-specific cyclin-E1 | 410 |
|  | *CDH1* | 16q22.1 | 16 | 439872 | Cadherin-1 | 882 |
|  | *CDH2* | 18q12.1 | 19 | 462815 | Cadherin-2 | 906 |
|  | *CDH3* | 16q22.1 | 19 | 205314 | Cadherin-3 | 829 |
| **BRCA**  **KIRC**  **LUSC**  **OV** | *CDKN1B* | 12p13.1 | 3 | 179590 | Cyclin-dependent kinase inhibitor 1B | 198 |
|  | *CHEK1* | 11q24.2 | 17 | 181759 | Serine/threonine-protein kinase Chk1 | 476 |
|  | *CHEK2* | 22q12.1 | 21 | 782581 | Serine/threonine-protein kinase Chk2 | 543 |
|  | *CLDN7* | 17p13.1 | 5 | 51048 | Claudin-7 | 211 |
|  | *COL6A1* | 21q22.3 | 35 | 66563 | Collagen alpha-1(VI) chain | 1028 |
|  | *CTNNB1* | 3p22.1 | 21 | 773073 | Catenin beta-1 | 781 |
|  | *DVL3* | 3q27.1 | 15 | 48733 | Segment polarity protein dishevelled homolog DVL-3 | 716 |
|  | *EEF2* | 19p13.3 | 15 | 30263 | Elongation factor 2 | 858 |
|  | *EEF2K* | 16p12.2 | 18 | 172590 | Eukaryotic elongation factor 2 kinase | 725 |
|  | *EGFR* | 7p11.2 | 32 | 252485 | Epidermal growth factor receptor | 1210 |
|  | *EIF4E* | 4q23 | 9 | 216950 | Eukaryotic translation initiation factor 4E | 217 |
|  | *EIF4EBP1* | 8p11.23 | 3 | 109757 | Eukaryotic translation initiation factor 4E-binding | 118 |
|  | *ERBB2* | 17q12 | 35 | 76163 | Receptor tyrosine-protein kinase erbB-2 | 1255 |
|  | *ERBB3* | 12q13.2 | 30 | 67889 | Receptor tyrosine-protein kinase erbB-3 | 1342 |
|  | *ERRFI1* | 1p36.23 | 4 | 127943 | ERBB receptor feedback inhibitor 1 | 462 |
|  | *ESR1* | 6q25.1–q25.2 | 22 | 1010787 | Estrogen receptor | 595 |
|  | *FN1* | 2q35 | 47 | 161105 | Fibronectin | 2477 |
|  | *FOXO3* | 6q21 | 10 | 412606 | Forkhead box protein O3 | 673 |
|  | *GAB2* | 11q14.1 | 14 | 403615 | GRB2-associated-binding protein 2 | 676 |
|  | *GATA3* | 10p14 | 9 | 53830 | Trans-acting T-cell-specific transcription factor GATA-3 | 443 |
|  | *GSK3A* | 19q13.2 | 11 | 46083 | Glycogen synthase kinase-3 alpha | 483 |
|  | *GSK3B* | 3q13.33 | 12 | 328685 | Glycogen synthase kinase-3 beta | 420 |
|  | *HSPA1A* | 6p21.33 | 1 | 30081 | Heat shock 70 kDa protein 1A | 641 |
|  | *IGFBP2* | 2q35 | 6 | 635508 | Insulin-like growth factor-binding protein 2 | 325 |
|  | *INPP4B* | 4q31.21 | 50 | 974280 | Inositol polyphosphate 4-phosphatase type II | 924 |
|  | *IRS1* | 2q36.3 | 4 | 340500 | Insulin receptor substrate 1 | 1242 |
|  | *ITGA2* | 5q11.2 | 30 | 327210 | Integrin alpha-2 | 1181 |
|  | *JUN* | 1p32.1 | 1 | 186656 | Transcription factor Jun | 331 |
|  | *KDR* | 4q12 | 30 | 157686 | Vascular endothelial growth factor receptor 2 | 1356 |
|  | *KIT* | 4q12 | 21 | 215842 | Mast/stem cell growth factor receptor Kit | 976 |
|  | *LCK* | 1p35.2 | 13 | 126692 | Tyrosine-protein kinase Lck | 509 |
|  | *MAP2K1* | 15q22.31 | 12 | 161693 | Dual specificity mitogen-activated protein kinase kinase 1 | 393 |
|  | *MAPK1* | 22q11.22 | 9 | 220191 | Mitogen-activated protein kinase 1 | 360 |
|  | *MAPK14* | 6p21.31 | 23 | 201005 | Mitogen-activated protein kinase 14 | 360 |
|  | *MAPK3* | 16p11.2 | 10 | 93043 | Mitogen-activated protein kinase 3 | 379 |
|  | *MAPK8* | 10q11.22 | 16 | 368470 | Mitogen-activated protein kinase 8 | 427 |
|  | *MAPK9* | 5q35.3 | 18 | 73212 | Mitogen-activated protein kinase 9 | 424 |
|  | *MET* | 7q31.2 | 22 | 293954 | Hepatocyte growth factor receptor | 1390 |
|  | *MRE11A* | 11q21 | 22 | 389527 | Double-strand break repair protein | 708 |
|  | *MYC* | 8q24.21 | 3 | 432193 | Myc proto-oncogene protein | 454 |
|  | *NF2* | 22q12.2 | 20 | 177025 | Merlin | 595 |
|  | *NFKB1* | 4q24 | 27 | 342366 | Nuclear factor NF-kappa-B p105 subunit | 968 |
|  | *NOTCH1* | 9q34.3 | 34 | 107711 | Neurogenic locus notch homolog protein 1 | 2555 |
|  | *PARK7* | 1p36.23 | 7 | 90862 | Parkinson disease protein 7 | 189 |
|  | *PCNA* | 20p12.3 | 7 | 138771 | Proliferating cell nuclear antigen | 261 |
|  | *PDK1* | 2q31.1 | 28 | 255474 | [Pyruvate dehydrogenase (acetyl-transferring)] kinase isozyme 1, mitochondrial | 436 |
|  | *PEA15* | 1q23.2 | 4 | 115700 | Astrocytic phosphoprotein PEA-15 | 130 |
|  | *PGR* | 11q22.1 | 10 | 655813 | Progesterone receptor | 933 |
|  | *PIK3CA* | 3q26.32 | 22 | 249827 | Phosphatidylinositol 4,5-bisphosphate 3-kinase catalytic subunit alpha isoform | 1068 |
|  | *PRKAA1* | 5p13.1 | 12 | 20122485 | 5'-AMP-activated protein kinase catalytic subunit alpha-1 | 559 |
|  | *PRKCA* | 17q24.2 | 24 | 618220 | Protein kinase C alpha type | 672 |
|  | *PRKCD* | 3p21.1 | 22 | 54445 | Protein kinase C delta type | 676 |
|  | *PTEN* | 10q23.31 | 10 | 188936 | Phosphatidylinositol 3,4,5-trisphosphate 3-phosphatase and dual-specificity protein phosphatase PTEN | 403 |
|  | *PXN* | 12q24.23 | 19 | 86157 | Paxillin | 591 |
|  | *RAD50* | 5q31.1 | 25 | 169061 | DNA repair protein RAD50 | 1312 |
|  | *RAD51* | 15q15.1 | 14 | 167019 | DNA repair protein RAD51 homolog 1 | 339 |
|  | *RAF1* | 3p25.2 | 23 | 191324 | RAF proto-oncogene serine/threonine-protein kinase | 648 |
|  | *RB1* | 13q14.2 | 29 | 323154 | Retinoblastoma-associated protein | 928 |
|  | *RPS6* | 9p22.1 | 6 | 178323 | 40S ribosomal protein S6 | 249 |
|  | *RPS6KB1* | 17q23.1 | 19 | 164736 | Ribosomal protein S6 kinase beta-1 | 525 |
|  | *SERPINE1* | 7q22.1 | 9 | 38294 | Plasminogen activator inhibitor 1 | 402 |
|  | *SHC1* | 1q21.3 | 15 | 24970 | SHC-transforming protein 1 | 583 |
|  | *SMAD1* | 4q31.21 | 17 | 212624 | Mothers against decapentaplegic homolog 1 | 465 |
|  | *SMAD3* | 15q22.33 | 15 | 517597 | Mothers against decapentaplegic homolog 3 | 425 |
|  | *SMAD4* | 18q21.2 | 14 | 135886 | Mothers against decapentaplegic homolog 4 | 552 |
|  | *SRC* | 20q11.23 | 21 | 76194 | Proto-oncogene tyrosine-protein kinase Src | 536 |
|  | *STAT3* | 17q21.2 | 24 | 261170 | Signal transducer and activator of transcription 3 | 770 |
|  | *STAT5A* | 17q21.2 | 20 | 204382 | Signal transducer and activator of transcription 5A | 794 |
|  | *STK11* | 19p13.3 | 12 | 36004 | Serine/threonine-protein kinase STK11 | 433 |
|  | *STMN1* | 1p36.11 | 8 | 136579 | Stathmin | 149 |
|  | *SYK* | 9q22.2 | 15 | 234227 | Tyrosine-protein kinase SYK | 635 |
|  | *TP53* | 17p13.1 | 13 | 64745 | Cellular tumor antigen p53 | 393 |
|  | *TP53BP1* | 15q15.3 | 32 | 173436 | TP53-binding protein 1 | 1972 |
|  | *TSC2* | 16p13.3 | 45 | 108996 | Tuberin | 1807 |
|  | *XRCC1* | 19q13.31 | 17 | 75379 | DNA repair protein XRCC1 | 633 |
|  | *XRCC5* | 2q35 | 21 | 433175 | X-ray repair cross-complementing protein 5 | 732 |
|  | *YAP1* | 11q22.1 | 11 | 233802 | Transcriptional coactivator YAP1 | 504 |
|  | *YBX1* | 1p34.2 | 9 | 286088 | Y-box-binding protein 1 | 324 |

**BRCA,** Breast invasive carcinoma; **COAD,** Colon adenocarcinoma; **KIRC,** Kidney renal clear cell carcinoma; **LUSC,** Lung squamous cell carcinoma; **OV,** Ovarian serous cystadenocarcinoma; **UCEC,** Uterine Corpus Endometrial Carcinoma.
